# Efficient and risk-reduced genome editing using double nicks enhanced by bacterial recombination factors in multiple species

**DOI:** 10.1093/nar/gkaa195

**Published:** 2020-03-30

**Authors:** Xiaozhen He, Wenfeng Chen, Zhen Liu, Guirong Yu, Youbang Chen, Yi-Jun Cai, Ling Sun, Wanli Xu, Lili Zhong, Caixi Gao, Jishen Chen, Minjie Zhang, Shengxi Yang, Yizhou Yao, Zhiping Zhang, Fujun Ma, Chen-Chen Zhang, Hui-Ping Lu, Bin Yu, Tian-Lin Cheng, Juhui Qiu, Qing Sheng, Hai-Meng Zhou, Zhi-Rong Lv, Junjun Yan, Yongjian Zhou, Zilong Qiu, Zongbin Cui, Xi Zhang, Anming Meng, Qiang Sun, Yufeng Yang

**Affiliations:** 1 Institute of Life Sciences, Fuzhou University, Fuzhou, Fujian 350108, China; 2 Institute of Neuroscience, CAS Center for Excellence in Brain Science and Intelligence Technology, State Key Laboratory of Neuroscience, CAS Key Laboratory of Primate Neurobiology, Chinese Academy of Sciences, Shanghai 200031, China; 3 State Key Laboratory of Biomembrane and Membrane Engineering, Tsinghua-Peking Center for Life Sciences, School of Life Sciences, Tsinghua University, Beijing 100084, China; 4 College of Life Sciences, Zhejiang Sci-Tech University, Hangzhou, Zhejiang 310018, China; 5 Zhejiang Provincial Key Laboratory of Applied Enzymology, Yangtze Delta Region Institute of Tsinghua University, Jiaxing, Zhejiang 314006, China; 6 State Key Laboratory of Freshwater Ecology and Biotechnology, Institute of Hydrobiology, Chinese Academy of Sciences, Wuhan, Hubei 430072, China; 7 Department of Gastric Surgery, Union Hospital of Fujian Medical University, Fuzhou, Fujian 350001, China

## Abstract

Site-specific DNA double-strand breaks have been used to generate knock-in through the homology-dependent or -independent pathway. However, low efficiency and accompanying negative impacts such as undesirable indels or tumorigenic potential remain problematic. In this study, we present an enhanced reduced-risk genome editing strategy we named as NEO, which used either site-specific *trans* or *cis* double-nicking facilitated by four bacterial recombination factors (RecOFAR). In comparison to currently available approaches, NEO achieved higher knock-in (KI) germline transmission frequency (improving from zero to up to 10% efficiency with an average of 5-fold improvement for 8 loci) and ‘cleaner’ knock-in of long DNA fragments (up to 5.5 kb) into a variety of genome regions in zebrafish, mice and rats. Furthermore, NEO yielded up to 50% knock-in in monkey embryos and 20% relative integration efficiency in non-dividing primary human peripheral blood lymphocytes (hPBLCs). Remarkably, both on-target and off-target indels were effectively suppressed by NEO. NEO may also be used to introduce low-risk unrestricted point mutations effectively and precisely. Therefore, by balancing efficiency with safety and quality, the NEO method reported here shows substantial potential and improves the *in vivo* gene-editing strategies that have recently been developed.

## INTRODUCTION

Currently available precise defined genome editing methods such as knock-in (KI) and base editing are inadequate for biological research, despite the fact that a number of strategies have been employed to increase the efficiency and accuracy while reducing unwanted risks. Generally, site-specific DNA double-strand breaks (DSBs) are predominantly repaired by the non-homologous end joining (NHEJ) pathway or single-strand annealing (SSA) pathway, which are useful for knock-out in vertebrates (1,2). Although DSBs have been widely used for KI of large DNA fragments *in vivo* by NHEJ or homology-independent targeted integration (HITI) with acceptable efficiency (3–8), these strategies cannot be generally applied for a defined, precise and safe genome editing due to its error-prone nature and other limitations, such as frequent unwanted indel events, restricted targeting sites (mostly within the noncoding regions), incorporation of vector backbone, random insertion directions, and the risk of multi-insertions.

Nonetheless, more precise KIs through DSB-triggered homologous recombination (HR) have been achieved in many organisms. In general, there are two ways to enhance HR-KI: inhibiting NHEJ or directly promoting HR (9–15). There are two forms of NHEJs: classical NHEJs (c-NHEJs) and alternative NHEJs (alt-NHEJs; also known as backup NHEJ or microhomology-mediated end joining (MMEJ)) (16). c-NHEJ is mediated by LIG4 and the Ku70/80 heterodimer (also known as Xrcc6 and Xrcc5, respectively) (17). Conversely, alt-NHEJ is dependent on LIG3, PARP1 and DNA polymerase theta (Polθ; also known as POLQ) (16,18). Inhibition of c-NHEJ, through targeting LIG4, promotes alt-NHEJ and HR (19). In contrast, as an essential alt-NHEJ factor in mammalian cells, Polθ contains RAD51 binding motifs and blocks RAD51-mediated recombination. Recent studies have shown that the loss of Polθ resulted in a decreased rate of alt-NHEJ and an increased rate of HR (18,20). Moreover, co-injection of DSB-induced programmable nucleases and donor DNA to LIG4-deficient, Polθ-deficient or 53BP1-deficient embryos, could lead to a dramatic increase in HR targeting (14,19,21), which supported the working principle. However, the integrity of the entire genome might not be upheld in these gene-deficient embryos, as it may disrupt overall genetic background of a KI-modified individual. On the other hand, inhibitory small molecules cannot completely eliminate NHEJ, and this leaves the issue of heterogeneous mosaicism unresolved (12).

Attempts from other perspectives have been reported to directly enhance HR. Generally, DNA repair by HR is highly suppressed in G1 cells or non-dividing cells such as neurons and muscle cells, because end resection and the recruitment of BRCA2 to DNA breaks are inhibited in these cell types (22). Recently, Orthwein *et al.* have demonstrated that this suppression of HR is reversible through the manipulation of the BRCA1-PALB2 interaction and the activation of the DNA-end resection process (23). Additionally, Lin and colleagues increased the HR ratio in cultured human cells by synchronising cell growth and restricting the delivery timing of Cas9 ribonucleoprotein complexes in cellular late S and G2 phases (24). Song *et al.* have achieved significant improvement on the CRISPR/Cas9- and TALEN-mediated KI rates in rabbits by supplying HR enhancer (RS-1) (12). Charpentier and colleagues enhanced the HR efficiency by direct fusion of CtIP to Cas9 nuclease (13). Taken together, small molecules or genetic approaches that partially block NHEJs or directly enhance HR have been developed to boost HR in the milieu of DSB (8–12,19,21). However, further optimisation is desirable as these DSB-based KI approaches were accompanied by low efficiency and unwanted on-target NHEJ events, as well as high percentages of miscellaneous inaccurate genetic incorporations (25–34) (Supplementary Table S1). In addition, off-target events are prevalent for almost all current programmable nucleases and excessive DSBs may cause genomic instabilities, which tremendously increase the tumorigenic potential of the edited cell population and poses a risk of cancer (35–37).

Therefore, generating DSBs may not be an ideal approach to realize programmable ‘scarless’ and ‘clean’ genome editing, which might jeopardize phenotyping experiments, complicate subsequent genetic procedures and even sabotage therapeutic applications, such that alternative approaches should be explored.

We speculated that DNA nicks could be an alternative approach, as nicks have been shown to be more amenable for precise KI in mammalian cells, *Drosophila*, as well as in mice (38–41). Targeted nicking of the genome can be accomplished by CRISPR/Cas9 nickase and also by non-CRISPR gene-editing nickases, such as ZFNs and TALENs (42). In the case of CRISPR/Cas9 system, a pair of cooperative single-chain guide RNAs (sgRNAs) coupled with D10A Cas9 nickase (referred to as Cas9n thereafter) could be used to generate so-called off-set nicks (39,43,44). A multiple nicking approach could be employed to overcome the otherwise extremely low mutagenesis capacity of one single DNA nick (41,43–46). Although the off-target ratio could be reduced to minimum in such a setup (43,47,48), the efficacy of KI was far from satisfactory, especially for long DNA integration. Furthermore, the undesirable mosaicism with on-target and off-target indels remain prevalent (43).

We reasoned that on the basis of double nicks, exogenous supplementation of recombination factors, which are rate-limiting enzymes in the HR process, might further suppress NHEJ and shunt the repairing route towards HR. In this study, we first chose zebrafish to test our hypothesis. We demonstrated that ectopic supply of the codon-optimized bacterial RecA protein together with RecO, RecR and RecF factors, could enhance accurate HR-KI induced by *trans*-dual nicks (two cooperative nicks induced on the complementary strands) or *cis*-dual nicks (both nicks on a same strand) in zebrafish. We then found that this method could also remarkably elevate accurate HR-KI efficiency in mice, rats and unstimulated primary human PBLCs. The genome-editing strategy was named as NEO (Nickase-based homologous recombination Enhanced by recOfar factors). In addition, both on-target and off-target indels that are prevalent when conventional Cas9 strategies are adopted could be substantially reduced via NEO in multiple eukaryotic species. Therefore, the advantages of NEO over current CRISPR/Cas9-mediated KI strategies lie in two scenarios: a significant improvement of efficiency and the robust suppression of undesired indels.

## MATERIALS AND METHODS

### Animal handling

The zebrafish work was conducted under full animal care and use guidelines with prior approval by the local institutional animal care committee's approval (Institute of Hydrobiology, Chinese Academy of Sciences). The wild-type line AB was used.

Rats and mice were maintained in standard laboratory conditions in a 12 h light/12 h dark cycle. All animal procedures were performed according to the ethical guidelines of the Institute of Neuroscience, Shanghai Institutes for Biological Science, Chinese Academy Science.

### Targeting vector construction

All the circular plasmid donors (Supplementary Text S1–S15) were constructed using the same strategy (Supplementary Figure S1A). First, we used the primers (Supplementary Table S2) to amplify 5′ homology arms (5′ HA), insert fragment, 3′ HA and donor backbone, respectively. Then, the 5′ HA, insert fragment and 3′ HA were ligated together following two steps of overlap extension PCR. Finally, 5′ HA-insert fragment-3′ HA ligation product and donor backbone were ligated together by using In-Fusion HD Cloning Kit (Clonetech, 639649). Demethylated plasmids were derived from Trans110 Chemically Competent Cell (CD311, TransGen Biotech).

### Production of Cas9n mRNA, RecOFAR mRNAs, TagBFP mRNA and sgRNA

NLS-hSpCas9-NLS and NLS-hSpD10ACas9n-NLS cassettes from pX330 (Addgene Plasmid, #42230) and pX335 (Addgene Plasmid, #42235) were cloned into pSP73 vector (Promega) after SP6 promoter (Supplementary Text S16-S17). Cas9n and Cas9 mRNA were transcribed *in vitro* according to the Kit instructions. Specifically, these two plasmids were linearized and recovered as corresponding template. Transcriptions were carried out using mMESSAGE mMACHINE SP6 Transcription Kit (Ambion, USA, AM1340) and Poly(A) signal was added to the 3′ end of capped mRNAs by Poly(A) Tailing Kit (Ambion, USA, AM1350).

The RecA, RecF, RecO and RecR encoding sequences were cloned and optimized to vertebrate preference codons. RecOFAR ORFs were also ligated into pSP73 vector after SP6 promoter and transcribed as Cas9n with poly (A) tailing (Supplementary Text S18-S21). TagBFP ORF was amplified from pTagBFP-N (P0695, MiaoLingPlasmid) with SP6 promoter in the forward primer and transcribed with poly (A) tailing (Supplementary Text S22). sgRNA synthesis scheme is showed in Supplementary Figure S1B. Briefly, the sgRNA gene-specific primers and a sgRNA scaffold primer (Supplementary Table S3) extend directly via the 3′end complementary sequences by PCR amplify (Supplementary Figure S1B). Then, the PCR products which contain T7 promoter were purified via phenol/chloroform extraction, and transcriptions were carried out using MEGA shortscript T7 Transcription Kit (Ambion, USA, AM1354).

### *In vitro* DNA cleavage assay with Cas9 nuclease

For the fish *GFAP*, *Ndr2*, *lefty2* loci, mice *Slc6a4*, *Myc* locus and monkey *Oct4*, *CAMK2A* loci, primers flanking the sgRNA targeting sites in the genome were used to PCR amplify for Cas9 cleavage. For *CAMK2A*, 5′ HA and 3′ HA, which contains the sgRNA recognizing sites were cloned into vector and used as Cas9 cleavage templates. Briefly, 200 nM dsDNA, 100 nM sgRNA, 0.5 U Cas9 (V-Solid Biotech, E025), 2 μl 10× buffer were used to construct reaction mixture. The mixture was incubated at 37°C for 1 h before fragment analysis.

### Microinjection

In the fish experiments, one-cell-stage embryos were microinjected with ∼1 nl of solution containing: 100 ng/μl of RecA mRNA, 100 ng/μl of RecO mRNA, 100 ng/μl of RecR mRNA, 100 ng/μl of RecF mRNA, 50 ng/μl of sgRNA, 100 ng/μl Cas9n mRNA and 300 ng/μl of donor plasmid. The concentration is determined by NanoDrop2000 (ThermoFisher).

In the mice experiments, B6D2F1 (C57BL/6× DBA2) female mice and ICR mice were used as embryo donors and foster mothers, respectively. Superovulated female B6D2F1 mice (4–5 weeks old) were mated to B6D2F1 stud males. Fertilized embryos were collected from oviducts. The optimized concentrations of individual injection components are the same as in fish. Mouse embryos were immediately transferred to the oviducts of pseudopregnant SD or ICR females at 0.5 dpc after injection.

In the rat experiments, Sprague-Dawley (SD) female rats were used as embryo donors and foster mothers. Superovulated female SD rat (4 weeks old) were mated to SD stud males, and fertilized embryos were collected from oviducts. The optimized concentrations of individual injection components are the same as in fish. Rat embryos were immediately transferred to the oviducts of pseudopregnant SD or ICR females at 0.5 dpc after injection.

In the monkey experiments, the monkey fertilized eggs were constructed by injection of monkey sperm into monkey oocytes and cultured in HECM-9 medium. For RNAs and donor DNA injection, all the embryos were injected with the mixed RNAs and DNA donors in one cell stage by IM-300 microinjection system. The optimized concentrations of individual injection components are the same as in fish. Monkey embryos were cultured in KSOM or HECM-9 medium after injection until morula/blastocyst stage and harvested for genome extraction and analysis.

Notably, blinding was ensured for all microinjection and genotyping experiments.

### Chromatin immunoprecipitation (ChIP) assay for early zebrafish embryos

A translational fusion of HA-tag to the C terminus of RecA (RecA-HA), Myc-tag to RecOR (RecO-Myc, RecR-Myc) and Flag-tag to RecF (RecF-Flag) were used to do injection. Firstly, we co-injected tag-fused RecOFAR mRNAs together with the GFAP-gRNAs and Cas9n mRNA. Both *trans*- and *cis*-dual nicks were conducted. After 5 hours of injection, 500 embryos were collected for further ChIP assay. Before the DNA and proteins cross-linking procedure, the 500 embryos in PBS containing 20 mM Na-butyrate and protease inhibitors were transferred into a 5 ml syringe fitted with a 21 G needle. Then, the embryos were forced through the needle into a 1.5 ml tube to dissociate all cells. 1% formaldehyde was added and incubated for 8 min at room temperature. Then, 0.125 M glycine was added to quench excess formaldehyde and incubated on ice for 5 min. The embryos were centrifuged at 500 g for 10 min at 4°C and the supernatant was discarded. After further washed for twice with 500 μl ice-cold PBS containing protease inhibitors (49), the cells were used for ChIP assay with Pirece Agarose ChIP Kit (ThermoFisher Scientific, #26156). Further chromatin preparation and immunoprecipitation were conducted according to the Kit instructions. A rabbit monoclonal antibody of anti-HA-Tag (C29F4) (Cell Signal, #3724) or a mouse monoclonal antibody of anti-Myc-Tag (9B11) (Cell Signal, #2276) were used to do immunoprecipitation with 1:50 and 1:100 dilution, respectively. Two pairs of primers at the flank region of nicks were designed to detect ChIP and input DNA (Chip-f1: 5′-TGGTGTAGGGCAGTGGAGGTTAC-3′; Chip-r1: 5′-AGCCTAGTGGTTAAGTGCGCAAC-3′; Chip-f2: 5′-ATCACCATAAGAACCATGGTGC-3′; Chip-r2: 5′-GATCTGCGAAAGAGAGAATGTG-3′). The PCR was performed by using the DreamTaq Green PCR Master Mix (ThermoFisher Scientific, #K1081) under the following conditions: 1 cycle at 95°C for 4 min; 32 cycles for input DNA and 40 cycles for ChIP DNA at 95°C for 30 s, 60°C for 30 s and 72°C for 30 s.

### Identification of indels at fish *GFAP* and mouse *Slc6a4* on-target site by Next-generation sequencing

For fish *GFAP* locus, we co-injected the GFAP-gRNAs with Cas9n mRNA and donor plasmid together with or without RecOFAR mRNAs. After 5 and 10 h of injection, 30 embryos were collected for further PCR assay. For the mouse *Slc6a4* locus, we employed the tails from F_0_ generations as samples. Primers with different barcodes flanking fish *GFAP* and mouse *Slc6a4* on-target site sequences were designed to amplify the fragments of different samples. The two primers without barcodes were: NHEJ-fish-GFAP-F: 5′-GAACTCGGATCACCATAAGAACC-3′; NHEJ-fish-GFAP-R: 5′-AGGAGAGAAGCAGGGAAAGTTG-3′; NHEJ-mouse-Slc6a4-F: 5′-TCTTCTTTTAAAGGCTAGTGAGGCT-3′; NHEJ-mouse-Slc6a4-R: 5′-GGGCACCATAGTCTTTAGGACTG-3′. Then, the PCR products were purified and each sample with equal amount of DNA were pooled together and sent to do deep-sequencing for indels identification (Novogene, China). Briefly, the barcode and primer sequences were identified and the pooled paired-end reads were separated for each sample as FASTAQ files. Based on the paired-end reads’ overlaps, we joined the paired-end reads as tag sequences. Then, we employed the Burrows-Wheeler Aligner (BWA) to align the tag sequences with our target sequence. Next, we detected the CIGAR (Compact Idiosyncratic Gapped Alignment Report) character strings within the alignments to identify the complex variants and constructed the consensus sequences of each variant. Finally, we distinguished the complex variants which were located near the cleavage sites and calculated the variation frequency in each sample. The ratio data we showed is the percentage of reads with indels to the total reads. Each sample was repeated from two independently injection samples. Deep sequencing data were deposited in the NCBI Sequence Read Archive (SRA; http://www.ncbi.nlm.nih.gov/sra/) under accession numbers from SRR4431431 to SRR4431438 and SRR7125325.

### Genomic DNA preparation

Tail samples (or single larvae) from zebrafish of interest were placed into a 1.5 ml microcentrifuge tube. Add 200 μl Tail Buffer (10 mM Tris–HCl pH8.0, 10 mM EDTA, 200 mM NaCl, 0.5% SDS, 200 ng/μl Proteinase K) to the sample and incubate at 56°C overnight with mixing. The DNA were precipitated with two volume 100% Ethanol, washed with the 70% ethanol and dissolved with 50 μl TE buffer. For germline transmission screening, 30 embryos at 48 h were placed into a 1.5 ml microcentrifuge tube with 500 μl Tail Buffer, extracted as above and dissolved with 120 μl TE buffer.

For Rats and mice, Tail samples from animal of interest were placed into a 1.5 ml microcentrifuge tube. Add 200 μl Tail Buffer (50 mM Tris–HCl pH8.0, 100 mM EDTA, 100 mM NaCl, 1% SDS, 500 ng/μl Proteinase K) to the sample and incubate at 56°C overnight with mixing. The lysate samples were extracted twice with equal volume phenol–chloroform–isoamyl alcohol (PCI, 25:24:1). Finally, the DNA in extractions were precipitated with equal volume 100% isopropanol, washed with the 70% ethanol and dissolved with TE buffer.

For each monkey embryo, 0.1–0.5 μl of HECM-9 medium containing 1 morula/blastocyst was transferred to the wall near the bottom of a 0.2 ml thin wall PCR tube with cap. Thereafter, a REPLI-g Mini Kit (Qiagen, 150023) was used to do whole genome amplification according to the manufacturer's protocol. Briefly, 3.5 μl Buffer D2 and 2.5 μl PBS were gently added to each tube. Each PCR tube was incubated on ice for 10 min and 3.5 μl Stop Solution was added. Finally, 40 μl of REPLI-g solution was added to the mixture, which was incubated at 30°C for 16 h and at 65°C for 3 min. The amplified DNA was diluted 1:10 and 1 μl of diluted DNA was used for each PCR.

### Nest PCR

PCR was done in a 20 μl volume containing 50–100 ng genomic DNA. The primary PCR was performed by using the outside primer set (Supplementary Table S4) under the following conditions: 1 cycle at 95°C for 3 min; 10 cycles at 95°C for 20 s and 65°C (–0.5°C /cycle) for 2 min; 20 cycles at 95°C for 15 s and 60°C for 2 min; 1 cycle at 72°C for 10 min. The secondary and nested PCR was performed by using the primary PCR product as a template and inner primers (Supplementary Table S4) under the conditions: 1 cycle at 95°C for 4 min; 20 cycles at 95°C for 30 s, 64°C (–0.5°C /cycle) for 30 s and 72°C for 100 s; 20 cycles at 95°C for 30 s, 54°C (+0.5°C /cycle) for 30 s and 72°C for 100 s; 1 cycle at 72°C for 10 min. Full-length of KI-fragments (from upstream 5′ arm to downstream 3′ arm) were amplified and sequenced.

### Identification of off-target sites and T7 endonuclease I (T7EI) assay

In fish and mice experiment, we searched OTS against genome for matches to the 20 nt sgRNA sequences allowing for up to three mismatches followed by NRG PAM sequence using Cas-OFFinder (50). The selected OTS and on-target site were PCR amplified using tail genomic DNA as the templates. The PCR products were first subjected to the T7EI cleavage assay. The OTS that yielded typical pattern of cleavage bands were considered as candidates, and then the PCR products of the candidates were cloned and sequenced to confirm the off-target effects. Genomic DNA from targeted and control animals was extracted and PCR was performed using gene-specific primers (Supplementary Table S5) under the following conditions: 40× (98°C for 10 s, 60°C for 15 s, 72°C for 30 s). PCR products were then denatured at 95°C for 5 min and normally cooling to room temperature to anneal, and finally treated with T7EI nuclease (NEB, M0302S). Digested PCR products were separated on an ethidium-bromide-stained agarose gel (2%) for analysis. For sequencing, PCR products were cloned using the ZT4-Blunt Cloning Kit (ZOMANBIO, ZC205) or pEASY-Blunt Cloning Kit (TransGen Biotech, CB101), and mutations were identified by Sanger sequencing.

### Southern blotting

F_1_ or F_2_ animals were used for genome extraction and enzyme digestion. Then the samples were separated by agarose gel electrophoresis, and were transferred to positively charged nylon membrane by capillary siphoning. Following standard hybridization produces given in DIG High Prime DNA Labeling and Detection Starter Kit I (Roche, 11745832910), the membrane subsequently was baked at 80°C for 2 h in order to fix the DNA, and was hybridized with DIG-labeled probes. The PCR DIG Probe Synthesis Kit (Roche, Cat. No. 11636090910) and the primer pairs were employed for previous probes synthesis. Finally, the membrane combined with DIG-labeled probe was tested with DIG Nucleic Acid Detection Kit (Roche, 11175041910).

### Western blotting

Brain tissues from rat Drd1-p2A-ChR2-EYFP and Drd2-p2A-ChR2-EYFP F1 homozygous were lysed in 100 μl of a protein extraction buffer (10 mM Tris–HCl, pH8.0, 1 mM EDTA, 100 mM NaCl, 0.5% NP-40 supplemented with protease inhibitor cocktail and PMSF) on ice for 30 min. The lysate was then centrifuged at 12 000 rpm for 5 min. The supernatant was boiled with a loading buffer and was run on SDS-PAGE gels. A rabbit polyclonal antibody of anti-GFP (ThermoFisher, #A6455), a rabbit monoclonal antibody of anti-Drd1 (1H8L2) (ThermoFisher, #702593) and a rabbit polyclonal anti-Drd2 (Abcam, #ab21218) antibody were then used for western blotting analysis.

### Immunostaining analysis, image acquisition

Fish samples were perfused with 4% paraformaldehyde (PFA) in phosphate buffer. Fixed adult brain tissues were washed for 15 min in phosphate-buffered saline (PBS) containing 5% bovine serum albumin and 0.3% Triton X-100, and incubated with primary antibodies (in PBS with 3% BSA and 0.3% Triton X-100) overnight at 4°C and subsequently with corresponding secondary antibodies (Alexa Fluor-conjugated, Invitrogen, at 1:1000). Antibodies used: GFP antibody (Invitrogen, A11122), GFAP antibody (Millipore, ab5541).

Embryos at early stage were dechorionated and embedded in 0.2% agarose. Confocal images were obtained at an optical section thickness of 1–2 μm. A 405 nm (0.75 mW) laser was used to convert Dendra2 for 10–20 s. Fluorescence intensity was quantified with LAS AF lite software (Leica). Relative fluorescence intensity was calculated according to the fluorescence intensity in white region of interest (ROIs, *n* = 3) and blue ROI (*n* = 1) from unprocessed images for each sample. Three samples were quantified. Some images were processed with Photoshop.

Mice and rats were deeply anaesthetized with ketamine hydrochloride (5–10 mg/kg) and perfused with 4% PFA in phosphate buffer, and equilibrated in 30% sucrose. Fixed and equilibrated brain tissues were cut into 30 μm cortical sections with a Microm HM525 cryostat. Sections were washed for 5 min in phosphate-buffered saline (PBS) containing 5% bovine serum albumin and 0.3% Triton X-100, and incubated with primary antibodies (in PBS with 3% BSA and 0.3% Triton X-100) overnight at 4°C and subsequently with corresponding secondary antibodies (Alexa Fluor-conjugated, Invitrogen, at 1:1000). Antibodies used: 5-HTT (Immunostar, 24330), 5-HT (Immunostar, 20079), Drd1 (1H8L2) (ThermoFisher, #702593), Drd2 (Abcam, #ab21218) and GFP (ThermoFisher, #15379).

EdU staining process of hPBLCs was conducted according to the manufacturer's instruction (Life Technologies, Invitrogen™, C10044 and C10269). Human PBLCs were incubated with EdU for 48h, following the coloration of Alexa Fluor^®^ 594 azide for imaging (Life Technologies, Invitrogen™, C10270). Mito Tracker™ Red CMXRos kit (ThermoFisher, M7512) was used to stain the mitochondria of hPBLCs.

All experiments were imaged on a Lecia TCS SP5 confocal microscope. Confocal images were obtained at an optical section thickness of 1–2 μm.

### Human PBLCs isolation, culture, electroporation and image acquisition

Human PBLCs were isolated from either fresh whole blood or buffy coats obtained from the Union Hospital of Fujian Medical University Blood Center. A buffy coat is processed from a whole blood sample, which contains a mixture of lymphocytes, monocytes, some granulocytes and platelets. It was approved by the Medical Ethics Committee. PBLCs were isolated by Human peripheral blood lymphocyte separation fluid (Solarbio CAT.NO.P8610). Accordingly, we could get about 9 × 10^6^ cells in 25 ml fresh blood. The electroporation was conducted on Celetrix Electroporator (CTX-1500A-L, Celetrix LLC, Manassas VA, USA), which could achieve more than 50% mRNA or DNA delivery efficiency. We put about 2 × 10^6^ lymphocytes in a 20 μl tube for electroporation, the operation condition is 730 V/20 ms. Each 20 μl tube consumed 1.5 μg Cas9/Cas9n mRNA, 1 μg for each RecOFAR’s mRNA, 0.5 μg sgRNA, 1 μg Donor DNA and 1.5 μg BFP mRNA. After electroporation, hPBLCs were cultivated with 500 μl PEM-2 protective solution (post eletroporation medium, from Celetrix LLC) and 500 μl PRMI medium 1640 (Solarbio, Cat. No. 31800-500; add 10% FBS but not CD3, CD28 or IL-2), in a 24 well-plate. Images were acquired on a Leica TCS-SP5 confocal microscope in 36 h after electroporation through a whole area scanning. Results were obtained from at least 2000 BFP+ cells and three independent electroporation experiments. Every experimental replicate has been conducted with independent fresh preparation for DNA and RNA. Comparisons between groups were evaluated by Student's *t-*test.

### Indel detection by amplicon analysis (IDAA)

The experimental procedures for IDAA have been described by Lonowski *et al.* (51). IDAA PCR products were ranged from 100 to 500 bp and run on the instrument of ABI 3730XL (Shanghai Generay Biotech Co., Ltd). Standard Size is ROX 500 (Rhodamine B and Texas Red 500): 70, 80, 100, 120, 140, 160, 180, 200, 240, 280, 320, 360, 400, 450, 490, 500 bp. All primers concerned were listed in Supplementary Table S5. Data was applied to fragment analysis on ABI 3730XL Sequenator (ABI/Life Technologies, USA) using conditions recommended by the manufacturer. Raw data obtained were analysed using Peak Scanner Software V1.0 (http://resource.thermofisher.com/page/WE28396_2/). Total indel count ratio (shown in parenthesis) was calculated from peak areas of the summed indel peaks relative to the total peak area. When IDAA is used for knock-in mutagenesis analysis, the detection of the control group with mock treatment will help distinguish SNPs or any nonspecific bands amplified by PCR.

### Statistical analysis

Statistical analyses were performed with the GraphPad Prism 5 (GraphPad Software, Inc., CA, USA) software. For all *in vivo* analysis, sample sizes were chosen to ensure adequate power to detect a pre-specified effect size. Animals were excluded from analysis based on the absence of breeding ability. Animals were randomly selected to produce embryos for injection or breeding. Blinding was ensured for all microinjection and genotyping experiments. Comparisons between groups were evaluated by Student's *t-*test. *F*-test was used to assess variances. Chi-square with Fisher's exact test was used in the toxicity assay. *P*-values of less than 0.05 and 0.01 were considered statistically significant and very significant, respectively. Data are presented as means ± s.d.

## RESULTS

### Testing dual nicks to surrogate DSBs in eliciting HR-KI in zebrafish

**Figure 1.**
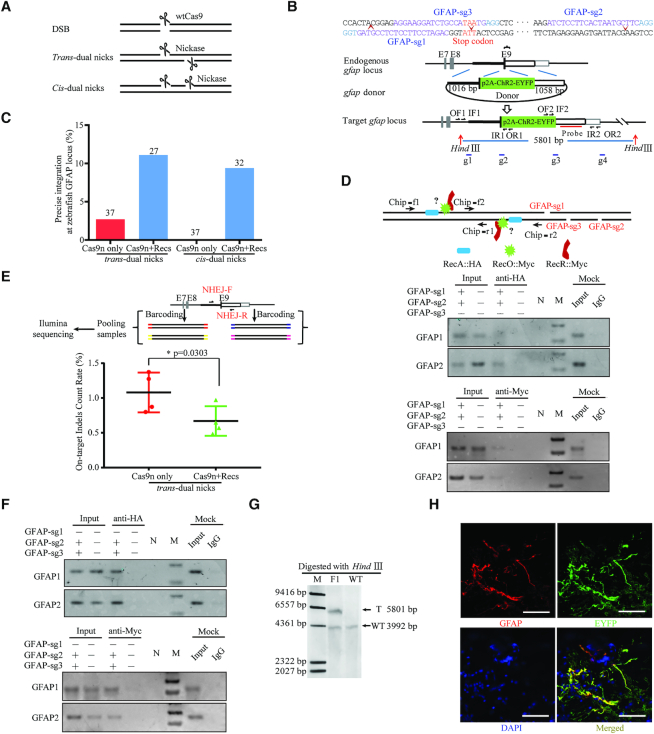
Design and optimisation of dual nicks-based gene KI at the *gfap* locus in zebrafish. (**A**) Schematic illustrating DNA break types. (**B**) Schematic overview of the strategy to generate a GFAP-p2A-ChR2-EYFP KI allele. The nested PCR primers used for knock-in identification are shown (Supplementary Table S4). (**C**) Germline transmission rate of ChR2-EYFP precise integration at the *gfap* locus with *trans-* and *cis-*dual nicks. (**D**) ChIP assay at the *gfap* locus subjected to *trans-*dual nicks processing. Each sample contained 500 embryos and three replicates were performed. (**E**) On-target indels analysis using Next-Gen-based multiplexed sequencing. The ratio data was presented as the percentage of reads with indels to the total reads according to the reference (62). Each sample contained 30 embryos and two replicates were performed. Comparisons between groups were analyzed with Student's *t-*test. * indicates *P* < 0.05. Error bars denote s.d. (**F**) ChIP assay at the *gfap* locus subjected to *cis-*dual nicks processing. Each sample contained 500 embryos and three replicates were performed. (**G**) Representative Southern blotting analysis of the GFAP-p2A-ChR2-EYFP targeted allele. T, KI target band. WT, wild type band. (**H**) ChR2-EYFP signals in the adult brain sections (hindbrain), nuclei in blue were stained with DAPI. Scale bar, 50 μm.

It has been demonstrated that the presence and polarity of the overhang structure is a critical determinant of DNA repair pathway choice, and D10A Cas9n-induced nicks are predominantly repaired by the HR machinery independent of the locus targeted (40,52). We speculated that two forms of dual nicks (*trans*-dual nicks or *cis*-dual nicks) could be a better way to initiate HR mediated KI over DSB (Figure 1A). We first chose zebrafish to explore the possibility of optimising nick mediated KI. We selected a locus of the glial fibrillary acidic protein (GFAP) gene and attempted to fuse a p2A-ChR2-EYFP reporter (∼1.7 kb) in-frame to the last codon, which could label GFAP-positive cells and enable optogenetic manipulation of glial cells in the future. A pair of sgRNAs (GFAP-sg1 and GFAP-sg2) coupled with Cas9n mRNA was applied to generate *trans*-dual nicks (Figure 1B). Previous study demonstrated that nicks on the transcribed strand might stimulate higher HR efficiency than nicks on the non-transcribed strand (40). However, *cis*-dual nicks have been shown to be incapable of inducing KI in mammals (43), hence we also wanted to revisit this possibility. To this end, we designed another sgRNA (GFAP-sg3) paired with GFAP-sg2 to induce *cis*-dual nicks on the transcribed strand of the *GFAP* gene (Figure 1A, B). The sgRNAs-mediated cleavage capacity was ensured by an *in vitr*o assay using recombinant wild-type Cas9 (wtCas9) protein prior to KI experiments (Supplementary Figure S2).

Then, we considered the features of the donor template DNA, which would affect the quality of KI experiments. First, given that ssDNA oligonucleotides would result in a large number of unintended mutations accompanying target modified alleles (3,25,27,53), we chose dsDNA as donor molecules. Second, although linear dsDNA donor templates with intact nuclease cleavage sites could improve integration efficiency (28,29,54,55), nuclease-blocking site mutations could minimize undesirable error-prone re-editing and thus increase the accuracy (56). As a result, we adopted the latter strategy for quality preference with a cost of reduced KI efficiency (28,29,54,55). Third, since linear donor dsDNA would increase the risk of random insertion, circular plasmids were chosen as the donor templates. Taken together, circular donor plasmids with CRISPR/Cas9 blocking mutation were applied in our study, with ∼1 kb upstream and downstream homology arms, respectively (Figure 1B).

We then tested whether the potential *GFAP* targeting events could be transmitted through the germ line using the dual nicks-based approach. We outcrossed founder fish (F_0_) with wild-type fish and collected the progeny embryos (F_1_). We pooled F_1_ embryos in two or three groups of 30 embryos each from individual founders and examined them by PCR and sequencing for correct integration of p2A-ChR2-EYFP (Supplementary Figures S3A and S3B). In line with previous reports, the germline transmission frequency was extremely low 2.7% (1/37) for *trans-*dual nicks and zero (0/37) for *cis*-dual nicks (‘Cas9n only’, Figure 1C and Supplementary Table S6).

### Optimized bacterial RecOFAR were recruited to dual nicks to suppress on-target indels in zebrafish

In the bacterial system, RecA and RecORF (RecO, RecR and RecF) act concomitantly to boost non-DSB-mediated recombination at the ss-dsDNA (single-stranded and double-stranded DNA) junctions (57–59). RecO could displace single-stranded binding (SSB) protein from ssDNA and interact with RecR to promote the loading of RecA onto the ssDNA that entails homology searching, which is stimulated by RecF (59,60). Given that unambiguous homologs of RecORF have yet to be clarified in eukaryotic cells and the catalyzing activity of RecORF proteins seem to be specific to RecA protein but not to its eukaryotic homologous RAD51 (57,60,61), we decided to introduce a cohort of all these 4 bacterial recombination factors to promote HR elicited by double nicks. Sequences of bacterial RecOFAR were codon-optimized and a nuclear localization signal (NLS) was added according to the vertebrate preference. The toxicity of the RecOFAR mRNAs, CRISPR/Cas9n ingredients and donor DNA were assessed systematically in zebrafish embryos. Statistical analysis showed that exogenous RecOFAR mRNAs up to 0.1 ng per embryo were nearly harmless to the embryos (Supplementary Table S7).

To determine whether prokaryote-derived recombination factors could be recruited to the DNA regions close to the nicks in eukaryotic cells, we carried out a chromatin immunoprecipitation (ChIP) assay. To facilitate the process, fusion proteins of RecA::HA, RecF::FLAG, RecO::Myc and RecR::Myc were constructed with the tags added in the C-terminus, respectively. We co-injected mRNAs encoding tag-fused RecOFAR proteins together with *GFAP*-targeted gRNAs (GFAP-sg1 & sg2) and Cas9n mRNA to the one-cell-stage zebrafish embryos. We found that DNA fragments near the *trans*-dual nicks could be enriched in both anti-HA and anti-Myc ChIP assay (Figure 1D). This indicated that RecA, RecOR were specifically localized to the region of induced DNA lesion. The engagement of RecF to DNA lesions could be rather transient that the recruitment might not be easy to capture by ChIP assay. Moreover, by using the deep-sequencing analysis method (62), we found that the percentage of indels at the on-target site was significantly reduced with the supplementation of RecOFAR (Figure 1E and Supplementary Excel S1). Collectively, it suggested that ectopic RecOFAR can suppress the NHEJ events by specifically binding to the DNA lesion in eukaryotic cells. Intriguingly, we found that RecA and RecOR could also be recruited to the DNA region near the *cis*-dual nicks elicited by GFAP-sg2 & sg3 (Figure 1F), indicating RecOFAR might also function in the context of *cis*-dual nicks.

### RecOFAR significantly elevated germline transmission frequency triggered by both *trans-* and *cis-* dual nicks in zebrafish

When RecOFAR mRNA was co-injected with donor plasmid and CRISPR/Cas9n components, the germline transmission frequency of *GFAP* KI fish was elevated to 11.1% (3/27) for *trans*-dual nicks and 9.4% (3/32) for *cis*-dual nicks (Figure 1C and Supplementary Table S6). Precise integrations were further verified in F_1_ fish by Southern blotting analysis and immunostaining (Figure 1G and H). The ratio of F_1_ fish carrying distinct KI-targeted events from individual NEO-generated founders ranged from 1.4% (1/74) to 12.1% (10/80) (Supplementary Table S8). These findings corroborated our hypothesis that bacterial RecOFAR could improve HR efficiency in eukaryotic cells. The feasibility of both *cis*- and *trans*-dual nicks thus renders the NEO system with great flexibility.

### NEO enabled more efficient and ‘clean’ KI in the exon region and all 4 Recs were indispensable for the efficacy

**Figure 2.**
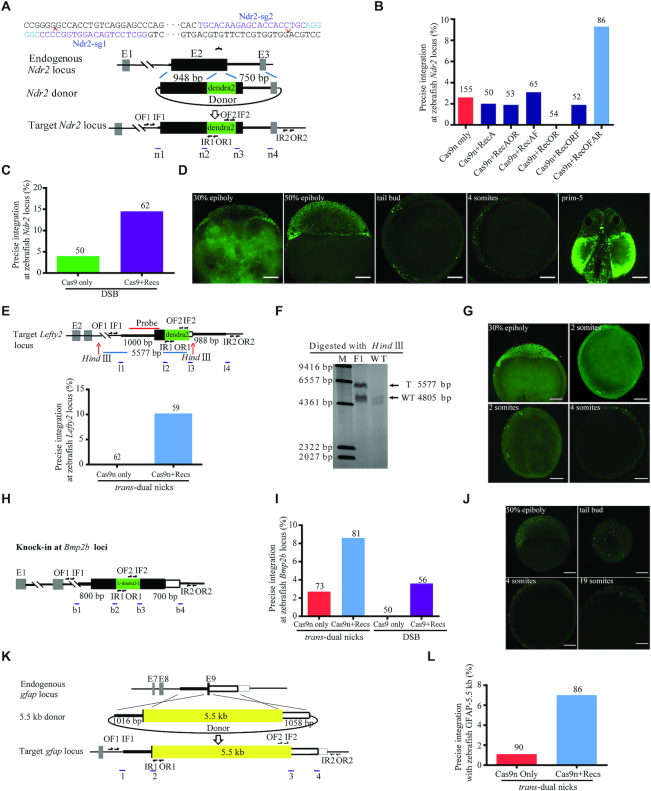
RecOFAR facilitate dual nicks-based efficient gene KI at multiple loci in zebrafish. (**A**) Schematic overview of the strategy to generate an Ndr2-linker-dendra2-V5 KI allele. The nested PCR primers used for knock-in identification are shown (Supplementary Table S4). (**B**) Germline transmission rate of linker-dendra2-V5 precise integration at the *Ndr2* locus with double-nicking approach. All four Rec factors are required for the enhancing effect. (**C**) Germline transmission rate of linker-dendra2 precise integration at the *Ndr2* locus with DSB approach. (**D**) Dendra2 fluorescence pattern in the Ndr2-linker-dendra2-V5 KI-positive F_2_ embryos. Scale bar, 100 μm. (**E**) Schematic overview of *Lefty2* target KI and the germline transmission rate of linker-dendra2 precise integration at the *Lefty2* locus. The nested PCR primers used for knock-in identification are shown (Supplementary Table S4). (**F**) Representative Southern blotting analysis of the *Lefty2* targeted allele. T, KI target band. WT, wild type band. (**G**) Dendra2 fluorescence pattern in the Lefty2-linker-dendra2-V5 KI-positive F_2_ embryos. Scale bar, 100 μm. (**H**) Schematic overview of the strategy to generate a Bmp2b-linker-dendra2-V5-linker allele. The nested PCR primers used for knock-in identification are shown (Supplementary Table S4). (**I**) Germline transmission rate of linker-Dendra2 precise integration at the *Bmp2b* loci with different strategies. (**J**) Dendra2 fluorescence pattern in the Bmp2b-linker-Dendra2-V5-linker KI-positive F_2_ embryos. Scale bar, 100 μm. (**K**) Schematic overview of the strategy to generate the *GFAP*-5.5kb KI allele. The nested PCR primers used for knock-in identification are shown (Supplementary Table S4). (**L**) Germline transmission rate of 5.5 kb precise integration at the *gfap* locus.

We then examined whether the NEO system could be adopted to integrate a long DNA fragment into a given exon region in the middle of a gene. To this end, we aimed to insert a Dendra2 (a GFP variant with the green-to-red photoconvertible property) fragment two amino acids downstream of the furin cleavage site between the pro- and mature domains of nodal-related 2 (Ndr2) (63) (Figure 2A). Notably, the targeting sites were in the exon II of *Ndr2* gene. Correct KI events were detected by PCR and sequencing (Supplementary Figure S3C and S3D). The germline transmission rate for Cas9n only group was 3.2% (3/93), the KI frequency was elevated to 9.3% (8/86) with the supply of RecOFAR mRNAs (Figure 2B and Supplementary Table S6). In order to test whether all four recombination factors are required, we set up parallel experiments with the addition of RecA, RecOR, RecAOR, RecAF and RecORF respectively. NEO (Cas9n+RecOFAR) achieved the highest KI efficiency up to 9.3% (8/86), with 3.1% (2/65) for the Cas9n+RecAF group, 2% (1/50) for the Cas9n+RecA group, 0% (0/54) for the Cas9n+RecOR group, 1.9% (1/52) for the Cas9n+RecORF group, 1.9% (1/53) for the Cas9n+RecAOR group and 2.6% (4/155) for the Cas9n only group, concluding that HR-KI could only be significantly enhanced if all four Rec factors were supplied (Figure 2B and Supplementary Table S6). We then tested whether the recombination factors might have an impact on KI that was mediated by wtCas9 (with Ndr2-sg1) as well. Interestingly, the germline transmission rate of successful KI was improved to ∼14% (9/62) in the wtCas9+RecOFAR group, when compared to 4% (2/50) with Cas9 only (Figure 2C and Supplementary Table S6). Dendra2 fluorescence in the early KI-positive embryos could be monitored (Figure 2D) and photo-converted (Supplementary Figure S4A and S4B), which displayed similar patterns to that of endogenous *Ndr2* (64).

Two potential off-target sites (OTS) of Ndr2-sg1 and 17 potential OTS of Ndr2-sg2 predicted by Cas-OFFinder (50) were first screened with the T7EI assay in some injected founder fish (the F_0_ individuals), which were then examined with the more sensitive and quantitative Indel Detection by Amplicon Analysis (IDAA) method (51) by capillary electrophoresis. Neither indels at the on-target sites, nor authentic mutations at OTS, were detected by the T7EI assay (Supplementary Figure S5 and Supplementary Table S5). However, IDAA detected obvious on-target indels in the Cas9n only group, which could be fully inhibited by the supplementation of RecOFAR factors (Supplementary Figure S6A and Supplementary Table S5). Moreover, IDAA identified one OT for Ndr2-sg1 that was generated by Cas9 and Cas9n at the Ndr2-sg1-OT1′, which was suppressed by adding RecOFAR. The indels count ratio was higher in wtCas9 than in Cas9n, which was consistent with previous reports (43) (Supplementary Figure S6B and Supplementary Table S5). IDAA also identified one OT for Ndr2-sg2 produced by Cas9n at the Ndr2-sg2-OT2′, which was also significantly reduced by RecOFAR factors (Supplementary Figure S6C and Supplementary Table S5).

### NEO enabled efficient HR-KI at various genomic regions in zebrafish

The applicability of the NEO system was further validated by targeting left-right determination factor 2 (*Lefty2*) gene in zebrafish. A similar *trans-*dual nicks strategy for *GFAP* editing was used to place a linker-dendra2-V5 cassette in-frame to the last codon of the *Lefty2* gene (Figure 2E), resulting in germline transmission frequency of 10.2% (6/59) for Cas9n+RecOFAR, while no KI was found for the Cas9n only group (0/62) (Figure 2E and Supplementary Table S6). The correct integrations in F_1_ were further confirmed by PCR, sequencing and Southern blotting analysis (Figure 2F, Supplementary Figure S3E and S3F). Dendra2 fluorescence in the early KI-positive embryos could be monitored and photo-converted (Figure 2G, Supplementary Figure S4C, D and Supplementary Movie S1). The fluorescence pattern of *Lefty2* KI fish was similar to the *Lefty2* mRNA expression pattern as reported (65) (http://zfin.org).

We further targeted the bone morphogenetic protein 2b (*Bmp2b*) gene with a linker-dendra2-V5-linker cassette in-frame to the potential cleavage site (REKR) within an exon region (Figure 2H). Correct KI events were detected by PCR and sequencing (Supplementary Figure S3G and S3H). The germline transmission rate for *Bmp2b* was 8.6% (7/81) in the Cas9n NEO group, compared to 2.6% (2/78) in the Cas9n only group. We did not get any positive KI fish in the offspring of the wtCas9 only group (0/50), while the KI efficiency could be enhanced to 3.6% (2/56) by adding the RecOFAR mRNAs to wtCas9 (Figure 2I and Supplementary Table S6). Dendra2 fluorescence in the early KI-positive embryos could be monitored (Figure 2J).

### NEO system enabled KI of >5.5-kb-long DNA cassettes into the zebrafish genome

Furthermore, we set out to achieve KI of an even larger DNA fragment in zebrafish. A DNA cassette over 5.5-kb-long (p2AV1-NpHR3.0-EYFP-p2AV2-ChR2-mCherry-IRES-WGA-Cre) was used as the donor DNA template for *GFAP* targeting (Figure 2K), and we achieved 7.0% (6/86) germline transmission frequency via NEO, in comparison to 1.1% (1/90) for the Cas9n only group (Figure 2L and Supplementary Table S6). Correct KI events were detected by PCR and sequencing (Supplementary Figure S3I and S3J).

### NEO system facilitated more efficient and ‘clean’ genome editing in mice

**Figure 3.**
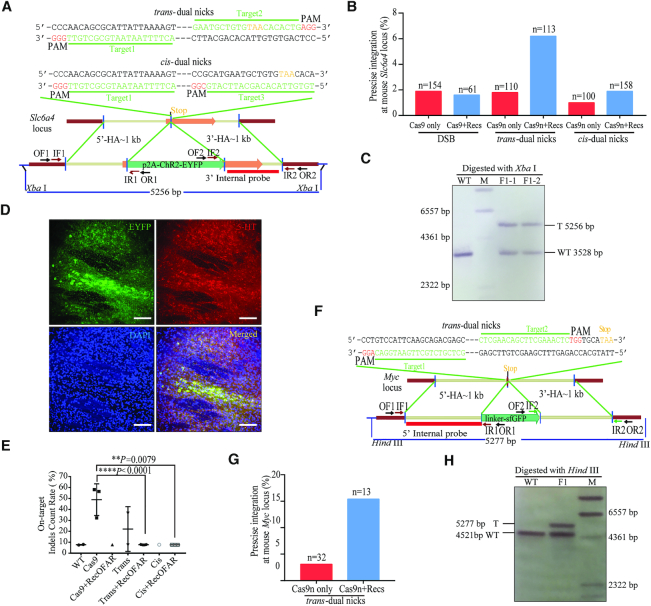
*Trans*- or *cis*-dual nicks with RecOFAR facilitate more efficient and ‘clean’ genome editing in mice. (**A**) Schematic overview of the strategy to generate a Slc6a4-p2A-ChR2-EYFP KI allele. The nested PCR primers used for knock-in identification are shown (Supplementary Table S4). Two targets are shown with green. The restriction endonuclease used for Southern blotting is XbaI. A 3′ internal probe on 3′ homology arm is shown. (**B**) Germline transmission rate of p2A-ChR2-EYFP precise integration at the *Slc6a4* locus with different strategies. (**C**) Southern blotting analysis of the *Slc6a4* KI-targeted allele and WT mouse. T, KI target band. WT, wild type band. (**D**) Immunostaining brain sections of *Slc6a4* KI F_1_ mice with anti-Serotonin (5-Hydroxytryptamine, 5-HT). ChR2-EYFP is in green and cells marked with anti-5HT are in red. DAPI in blue. Scale bar, 100 μm. (**E**) On-target indels analysis using Next-Gen-based multiplexed sequencing at the *Slc6a4* locus with KI-positive alleles. The tails DNA from F_0_ generations were used and amplified with the on-target primers marked with different barcodes. Mock, control group with mock treatment. Comparisons between groups were analyzed with Student's *t-*test. ** indicates *P* < 0.01, **** indicates *P* < 0.0001. Error bars denote s.d. (**F**) Schematic overview of the strategy to generate a Myc-linker-sfGFP KI allele. The nested PCR primers used for knock-in identification are shown (Supplementary Table S4). (**G**) Germline transmission rate of linker-sfGFP precise integration at the *Myc* locus with and without RecOFAR. (**H**) Southern blotting analysis of the *Myc* targeted allele and WT mouse. T, KI target band. WT, wild type band.

Next, we sought to extend this strategy to mammalian systems. To assess potential toxicity of ectopic supply of RecOFAR, we injected the engineered RecOFAR mRNAs into mouse embryos. At the working concentration of 100 ng/μl of each Rec's mRNA, no obvious toxicity was detected based on the ratio of developmental abnormality (Supplementary Figure S7A–G). We chose a locus within the mouse solute carrier family 6, member 4 (*Slc6a4*) gene for targeting: a p2A-ChR2-EYFP cassette was to be fused into the C-terminus of Slc6a4. We designed two sgRNAs for Cas9n to produce *trans*-dual nicks (Figure 3A). Donor plasmid with disrupted corresponding Cas9 cleavage sites was supplied as the exogenous HR template (Figure 3A). Nest PCR was used to screen the correct KI events in postnatal mice produced through direct zygote microinjection (Figure 3A). In the mice group injected with two sgRNAs and Cas9n, 2 out of 110 (1.8%) KI-positive individuals were detected (‘Cas9n only’, Figure 3B and Supplementary Table S6). By contrast, with the NEO system, we detected targeted insertion in 6.2% (7/113) of pups (Figure 3B and Supplementary Table S6). We further examined whether our NEO system could be compatible with *cis*-dual nicks on the non-transcribed strand of *Slc6a4* gene in mice (Figure 3A). The NEO system achieved the efficiency of KI as high as 1.9% (3/158), with 1% (1/100) for the group of Cas9n/sgRNAs alone (Figure 3B and Supplementary Table S6). In contrast, 1.9% (3/154) KI efficiency was detected in the group injected with a single sgRNA plus wtCas9, with 1.6% (1/61) for the wtCas9+RecOFAR group, inferior to that of NEO with *trans*-dual nicks (Figure 3B and Supplementary Table S6). PCR and sequencing results demonstrated high percentage of positive KI integration in the F_1_ generation animals (Supplementary Table S8), as was further verified by Southern blotting (Figure 3C) and immunostaining analysis (Figure 3D and Supplementary Figures S8, S9A–E). Deep-sequencing revealed an extraordinarily high percentage of undesired on-target indels in the *Slc6a4* KI-positive founders generated by wtCas9 or Cas9n only (35∼60%), which was completely prevented by RecOFAR, as the indels ratio was reduced to be the same as the background level of control animals (∼10%) (Figure 3E, Supplementary Figure S7H and Supplementary Excel S2). We also screened 11 potential OTS of *Slc6a4*-sg1 and 26 potential OTS of *Slc6a4*-sg2 in some *Slc6a4* KI-positive founder mice with the T7EI assay and IDAA assay (Supplementary Table S5). While T7EI failed to detect on-target indels or authentic mutations at OTS (Supplementary Figures S10 and S11), IDAA assay identified one OT at the *Slc6a4*–sg2-OT1′ locus in two KI-positive individuals of wtCas9 only group, with indels count ratios 20% higher than WT background, which was reduced by adding RecOFAR. On the other hand, the indels count ratio in the *trans* or *trans*+NEO group was on the same level with WT background (Supplementary Figure S6D). Also, we succeeded in applying our method to a second mouse locus. A linker-sfGFP cassette was inserted into mouse locus Myelocytomatosis oncogene (*Myc*) (Figure 3F). NEO achieved the KI efficiency up to 15.4%, with 3.1% for the Cas9n only group (Figure 3G, H, Supplementary Figure S9F and Supplementary Table S6). Thus, NEO is an efficient strategy for precise targeted integration in mice.

### NEO significantly improved accurate HR-KI in rats

**Figure 4.**
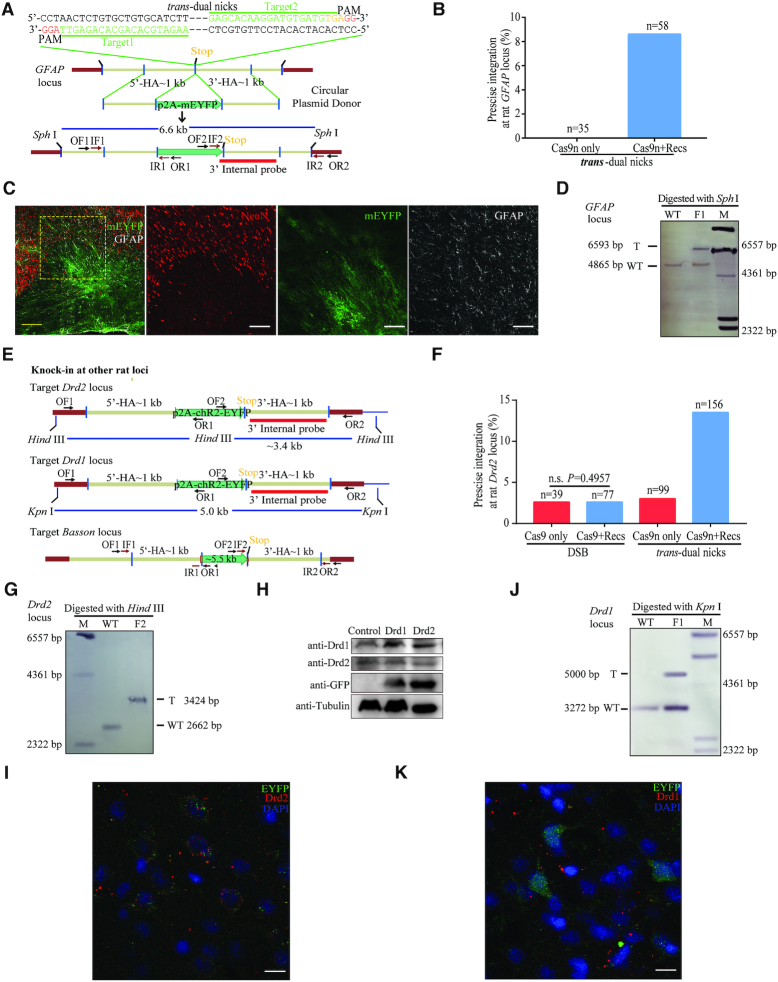
*Trans*-dual nicks with RecOFAR facilitates efficient gene KI in rats. (**A**) Schematic overview of the strategy to generate a GFAP-p2A-mEYFP KI allele. The nested PCR primers used for knock-in identification are shown (Supplementary Table S4). Two targets are shown with green. The restriction endonuclease used for Southern blotting is SphI. A 3′ internal probe on 3′ homology arm is shown. (**B**) Germline transmission rate of p2A-mEYFP precise integration at the *gfap* locus without and with RecOFAR. (**C**) Immunostaining brain sections of *gfap* KI-positive F_1_ rats. Neurons marked with NeuN are in red, mEYFP is in green and glial cells marked with anti-GFAP are in white. Scale bar in left panel is 200 μm. The region in the box is magnified in the right panels. Scale bar, 100 μm. (**D**) Southern blotting analysis of the *gfap* targeted allele and WT. T, KI target band, 6.6 kb. (**E**) Schematic overview of the strategy to generate the Drd2-p2A-ChR2-EYFP, Drd1-p2A-ChR2-EYFP and *Bassoon*-5.5 kb KI alleles. The restriction endonuclease HindIII and KpnI were used for Southern blotting at the *Drd2* and *Drd1* loci respectively. Two 3′ internal probes on 3′ homology arms are shown at the *Drd2* and *Drd1* loci. The PCR primers used for knock-in identification are shown (Supplementary Table S4). (**F**) Germline transmission rate of p2A-ChR2-EYFP precise integration at the *Drd2* locus with different strategies. (**G**) Southern blotting analysis of the Drd2-p2A-ChR2-EYFP targeted allele. (**H**) Western blotting analysis of *Drd1* and *Drd2* targeted allele in F_1_ generation. Brain tissues from Drd1-p2A-ChR2-EYFP and Drd2-p2A-ChR2-EYFP F_1_ homozygous were used as samples. (**I**) Immunostaining brain sections of *Drd2* KI-positive F_1_ rats. EYFP is in green and cells marked with anti-Drd2 are in red. DAPI in blue. Scale bar, 10 μm. (**J**) Southern blotting analysis of the Drd1-p2A-ChR2-EYFP targeted allele and WT. T, KI target band, 5.0 kb. (**K**) Immunostaining brain sections of *Drd1* KI-positive F_1_ rats. EYFP is in green and cells marked with anti-Drd1 are in red. DAPI in blue. Scale bar, 10 μm.

With the success of NEO-based genome editing in mice, we extended this strategy to rats. A similar *trans*-dual nicks strategy was used to place a fluorescent protein p2A-mEYFP in-frame to the last codon of *GFAP* (Glial fibrillary acidic protein), coding for a glia-specific protein (Figure 4A). In the rat group injected with the donor plasmid, two sgRNAs and Cas9n, KI events were not detected (Figure 4B and Supplementary Table S6). By contrast, NEO achieved a marked KI efficiency as high as 8.6% (5/58) in parallel experiments (Figure 4B and Supplementary Table S6). Furthermore, we obtained F_1_ generation from F_0_ KI-positive rats via NEO (Supplementary Table S8). Precise integrations were further confirmed by immunostaining (Figure 4C, Supplementary Figure S12A–C) and Southern blotting (Figure 4D). To further validate the applicability of our methods in rats, we employed this approach to insert a p2A-ChR2::EYFP into another locus, the *Drd2* (Dopamine receptor D2) locus with a C-terminal fusion (Figure 4E). While Cas9n only achieved with low efficiency of 3.0% (3/99), NEO resulted in 13.5% (21/156) of F_0_ pups with precise integration (Figure 4F and Supplementary Table S6). On the other hand, the wtCas9 only group resulted in a low efficiency of 2.6% (1/39) and the efficiency remained at the same level when added with RecOFAR (Figure 4F and Supplementary Table S6). Sequencing, southern blotting, western blotting and immunostaining indicated the precise integration (Figure 4G–I, Supplementary Figure S12D–F). We also screened 10 potential OTS of *Drd2*-sg1 and 9 potential OTS of *Drd2*-sg2 in KI-positive founder rats using IDAA, as we did in mice (Supplementary Table S5). IDAA identified one OT (*Drd2*-sg2-OT3 locus) with ∼20% higher indel count ratio in the wtCas9 only groups compared to the mock-treated groups, which was reduced by adding RecOFAR, while the indels count ratio for this potential OT locus in the Cas9n or Cas9n+RecOFAR group was at the same level with the mock groups (Supplementary Figure S6E). In addition, when applied to the *Drd1* (Dopamine receptor D1) locus, our NEO system also performed efficiently with an integration rate of 10.2% (10/98) (Figure 4E, H, J, K, Supplementary Figure S12G–I and Supplementary Table S6).

### NEO system enabled KI of >5.5-kb-long DNA cassettes into the rat genome

To test whether our NEO system could achieve KI of even larger DNA fragment in mammals. We chose a locus within the rat gene, *Bassoon* (coding for a presynaptic cytomatrix protein) for targeting: a fragment over 5.5-kb-long (the same as *GFAP*-5.5kb KI fragment in zebrafish) was aimed to be knocked into the C-terminus of *Bassoon* (Figure 4E). We found that NEO achieved a KI efficiency of 6.7% (2/30) (Supplementary Table S6).

### NEO strategy could induce accurate and efficient KI in the embryos of non-human primates

**Figure 5.**
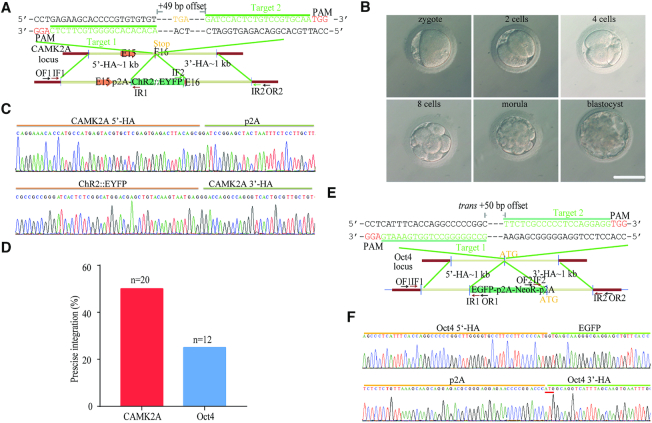
*Trans*-dual nicks with RecOFAR enable precise and efficient genome knock-in in monkey embryos. (**A**) Schematic overview of strategy to generate a *CAMK2A*-p2A- ChR2::EYFP knock-in allele. The nested PCR primers used for knock-in identification are shown (Supplementary Table S4). Two targets are shown with green. The offset distance between two targets is 49 bp. (**B**) Representative pictures of monkey embryos at distinct stages (Scale bar = 100 μm). The embryos at the morula/blastocyst stage were collected for genome extraction and analysis. (**C**) Successful HR of *CAMK2A*-p2A- ChR2::EYFP was confirmed by Sanger sequencing of the PCR amplicon. (**D**) Efficiencies of p2A-ChR2-EYFP precise integration at the *CAMK2A* locus and EGFP-p2A-NeoR-p2A precise integration at the *Oct4* locus in monkey embryos. (**E**) Schematic overview of strategy to generate an EGFP-p2A-NeoR-p2A-*Oct4* knock-in allele. The nested PCR primers used for knock-in identification are shown (Supplementary Table S4). Two targets are shown with green. The offset distance between two targets is 50 bp. (**F**) Successful HR of EGFP-p2A-NeoR-p2A-Oct4 was confirmed by Sanger sequencing of the PCR amplicon.

Furthermore, we aimed to evaluate whether NEO could be applied to non-human primates. We used a donor plasmid designed to introduce the p2A-ChR2::EYFP cassette into the *CAMK2A* locus (in-frame right before the stop codon) of *Macaca fascicularis* (Figure 5A). Genomic DNA of morulas/blastocysts developed from the injected zygotes was extracted, and PCR screening identified 10 out of 20 embryo showing correct recombination via NEO, while no on-target NHEJ indels were detected in all of the 10 KI-positive embryos (Figure 5B–D, Supplementary Figure S13 and Supplementary Table S6). Following this initial success, we knocked EGFP-p2A-NeoR-p2A fragment in the monkey *Oct4* gene and found that 3 out of 12 embryos had accurate KI integration via NEO (Figure 5E, F, Supplementary Figure S14 and Supplementary Table S6). Overall, we concluded that our NEO strategy could induce accurate and efficient KI in the embryos of non-human primates by one-step direct microinjection.

### NEO system achieved integration efficiency up to 20% in unstimulated primary human PBLCs

**Figure 6.**
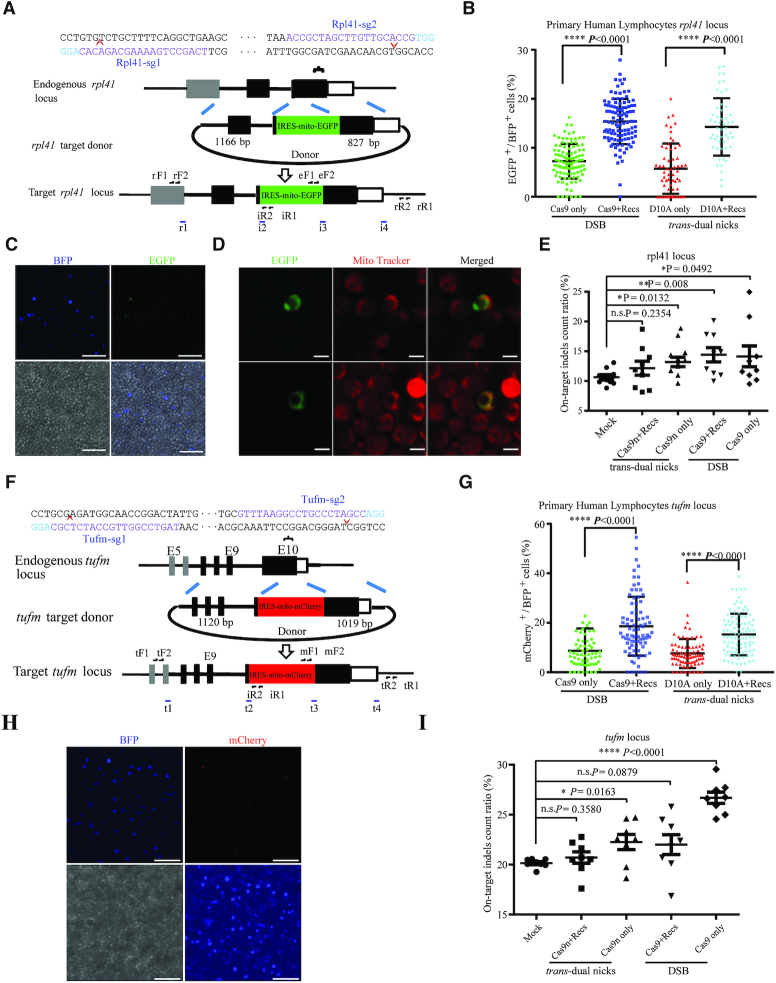
NEO achieved efficient integration in non-dividing primary human PBLCs. (**A**) Schematic overview of the strategy to generate the Rpl41-IRES-mito-EGFP cells. rF1, rF2, iR1, rR2, i2, i3, i4, eF1 and eF2 are the nest PCR primers (Supplementary Table S4). (**B**) Relative integration efficiencies (EGFP^+^ / BFP^+^) with IRES-mito-EGFP precise integration at the *Rpl41* locus. Results were obtained from at least 2000 BFP^+^ cells and three independent electroporation experiments. The input data points calculated from each randomly selected image field were shown as dots. ****P < 0.0001, Student's *t*-test. (**C**) Mito-EGFP signals in the *Rpl41*-KI positive hPBLCs. Cells in blue were positively transfected with TagBFP that served as an indicator. Scale bar, 50 μm. (**D**) Live staining of hPBLCs with mito Tracker Red. Green, *Rpl41*-KI positive hPBLCs. MitoTracker Red fluorescence dye specifically labelled mitochondria in red. Scale bar, 5 μm. (**E**) On-target indel count ratio detected by IDAA at the *Rpl41* locus. Total indel count ratio was calculated as the summed peak areas of all indel peaks relative to the total peak area. Results were obtained from at least three samples from independent electroporation experiments, each sample was tested three times. Mock, control group with mock treatment. n.s., no significant difference. *P < 0.05, **P < 0.01, Student's *t-*test. Error bars are s.d. (**F**) Schematic overview of the strategy to generate a TUFm-IRES-mito-mCherry KI cell. The nested PCR primers used for knock-in identification are shown (Supplementary Table S4). (**G**) Relative efficiencies of IRES-mito-mCherry precise integration at the *TUFm* locus. Results were obtained from at least 2000 BFP^+^ cells and three independent electroporation experiments. The input data points calculated from each randomly selected image field were shown as dots. ****P < 0.0001, Student's *t-*test. (**H**) Mito-mCherry signals in *TUFm*-KI positive hPBLCs. Cells in blue were positively transfected with TagBFP mRNA. Scale bar, 50 μm. (**I**) On-target indels count ratio detected by IDAA at the *TUFm* locus. Results were obtained from at least three samples from independent electroporation experiment, each sample was tested three times. Mock, control group with mock treatment. n.s., no significant difference, *P < 0.05, ****P < 0.0001, Student's *t-*test. Error bars denote s.d.

Lymphocyte genome engineering holds great promise for immune-therapies and cell-based therapies, however, genetic manipulation of unstimulated primary human lymphocytes has been inefficient (66–68). Moreover, the KI efficiency of long DNA sequence insertion for unstimulated human lymphocytes has yet to be determined as the low dividing ability poses an extra challenge for HR based genome editing (7,23). Given that recombination factors are ectopically provided, we therefore set out to examine if our NEO system could be applied in unstimulated primary hPBLCs, which were isolated from fresh whole blood and cultivated without any cytokines or stimulators. EdU staining confirmed that the unstimulated hPBLCs harboured very low dividing ability (∼0.16%) in 48h after separation (Supplementary Figure S15). Immediately after separation, the cells were supplied with wtCas9 or Cas9n mRNA, sgRNAs targeting the human *Rpl41* (ribosomal protein L41) locus, donor DNA with or without RecOFAR mRNA via electroporation (66,67) with co-delivered TagBFP mRNA as an internal reference. We monitored KI-positive events (indicated by the reporting GFP fluorescence) among the transfection-positive cells with the four types of recipes (wtCas9, wtCas9+RecOFAR, Cas9n and Cas9n+RecOFAR) respectively (Figure 6A). We found that the relative efficiency (EGFP^+^ / BFP^+^) could be improved from 6.7% to 14.9% with the addition of RecOFAR to Cas9n, and 7.3% to 15.4% with the addition of RecOFAR to wtCas9 (Figure 6B). The specific GFP signal in the KI-positive cells was co-localized with mito-Tracker Red staining as expected and the correct integrations were further confirmed by PCR and sequencing analysis (Figure 6C, D and Supplementary Figure S16A–D). In sharp contrast to the wtCas9, wtCas9+RecOFAR or Cas9n only groups that all resulted in obvious undesired on-target indels, NEO system did not generate discernible on-target indels according to IDAA (Figure 6E and Supplementary Figure S16E). IDAA detected one authentic OT out of 5 potential OTS for Rpl41-sgRNA2 (Rpl41-sg2-OT4 locus) in the Cas9n only group, which was eliminated by adding RecOFAR (Supplementary Figure S6F and Supplementary Table S5). The capacity of NEO system was further supported by accurate KI at the *TUFm* (Tu translation elongation factor, mitochondrial) locus. Cas9n gained 7.6% relative integration efficiency (mCherry^+^ / BFP^+^), while addition of RecOFAR to Cas9n achieved up to 15.2% relative integration efficiency, exhibiting two-fold improvement when compared to those without RecOFAR (Figure 6F–H and Supplementary Figure S17A–D). Similarly, the relative integration efficiency of successful KI was improved to 19% in the wtCas9+RecOFAR group, when compared to 8.7% with Cas9 only. IDAA detected obvious on-target indels in the wtCas9 or Cas9n only groups, which were fully inhibited by the supplementation of RecOFAR factors (Figure 6I and Supplementary Figure S17E).

## DISCUSSION

In an attempt to seek a potential replacement of DSBs to develop both efficient and low-risk genome editing approaches, we first showed that nicks mediated HR was generally low in zebrafish, mice and extremely low in rats (close to zero within the detecting range), indicating that the very low efficiency was, indeed, an obstacle for a broader application of nicking approach. We then illustrated that the ectopic supply of the primary recombinase proteins (RecA, RecO, RecR and RecF), which participate in HR of *E.coli*, could not only significantly improve HR-KI medicated by both *trans-* and *cis-*dual nicks in zebrafish, mammals and primary human cells but also have the conserved function of reducing the ratio of indels at both the on-target site and the potential off-target sites. The strength of our strategy, termed NEO, hinges upon two crucial elements, double nicks and recombination boosting factors RecOFAR, which may suppress error-prone DNA repair pathways and increase HR efficiency. The range of distance between *trans*-dual nicks was 46–99 bp, and 52–63 bp for *cis*-dual nicks in our study (Supplementary Table S6). In order to minimize the on-target indels while maintain efficiency at the same time, we recommend 50–150 bp as the optimal distance for the *trans*-dual nicks, and 50–100 bp for the *cis*-dual nicks.

At the six loci (n = 6) we compared the efficiency side by side, we found that KI rates with NEO was substantially higher than wtCas9 or Cas9n. It seems that low KI efficiency is more common than what has been expected, which has been reported by a number of previous publications (28,29,54,55). In line with this, all 8 loci that were investigated in this study seemed very resistant to KI editing by the conventional wtCas9- or Cas9n-mediated strategies, with a 1.9% of KI efficiency at average and even close to zero for some loci (*n* = 3). By contrast, our technology NEO, with *trans* dual nicks performed steadily and robustly at distinct loci among multiple species, with a 5-fold improvement (an average KI efficiency of 10.3%, Supplementary Table S6). For those loci with zero KI efficiencies (n = 3 loci, notably, in a relatively random gene selection process), the improvement is really outstanding (an average of 7.5% KI efficiency via NEO). Objectively, if an approach could obtain a KI-positive inheritable from only 10 F_0_ founders or less with simple reagents preparation, this proposed strategy should be attractive to most investigators, as well as the benefits of reducing undesirable risks via NEO.

Utilizing bacterial RecA to enhance wtCas9-mediated gene editing has been reported recently (69,70). However, in our study, we found that ectopic supplement of RecA only did not enhance nicks-mediated HR for germ-line transmission (Figure 2B and Supplementary Table S6). Clues from previous studies implicated that mediator proteins are needed to initiate recombination, otherwise RecA nucleation and RecA filament extension at the ss-dsDNA junction would be extremely inefficient (57–59,71,72). In *E. coli*, RecA is loaded through two major pathways: RecBCD and RecFOR. During double-strand break repair, the double-stranded ends are processed by the RecBCD helicase-nuclease pathway. The second loading pathway in *E. coli* is the RecFOR pathway, wherein RecO and RecR form a complex that is required for RecA loading onto single-stranded DNA binding protein (SSB)-coated ssDNA and subsequent RecA filament extension. The third component, RecF, can stimulate RecA nucleation, particularly on gapped DNA substrates (72). Indeed, it is amazing that the prokaryotic recombinant factors mixture works effectively in eukaryotes, probably due to a highly conserved mechanism for DNA repair. Although not clearly defined, the RecOFAR proteins have exact or potential structural or functional counterparts in both unicellular and multicellular eukaryotes, namely, Rad51 for RecA (73), Rad52 for RecO, yeast Rad55/57 likely for RecF/R, and probably human Rad51 paralogs (RAD51B, C and D) for RecF/R (61).

Interestingly, although this cocktail of bacterial recombinases, RecOFAR, could significantly improve the efficiency of nicks-mediated KI in all cases among all four species, we observed their positive effects on wtCas9 only in fish and hPBLCs. These results indicated that nicks and DSB may have distinct DNA repair mechanisms, as the overhang configuration at the DNA lesion site is a critical determinant of repair pathway choice (40,52). To this end, RecABCD, other than RecOFAR, might be more suitable to DSBs that are generated by wtCas9.

Through deep-sequencing, we analyzed the on-target NHEJ frequency in mouse *Slc6a4* KI positive F_0_ founders generated by 6 different strategies (*trans* nicks, *trans*+RecOFAR, *cis*, *cis*+RecOFAR, wtCas9, wtCas9+RecOFAR) (Figure 3E, Supplementary Figure S7H and Supplementary Excel S2). When compared to wild-type control, wtCas9-DSB induced more than 50% reads with indels at the on-target site, while NEO system resulted in minimal indels (the same level as background). In addition, we demonstrated that the ectopic RecOFAR could be enriched to the DNA damage region, which was in line with previous reports (57–59). Collectively, we speculate that the RecOFAR-ssDNA complex could facilitate RecA-filament formation, long-range homology search and accelerate DNA strand exchange, three essential steps of recombinational repair. At the off-target site, DNA lesions might be repaired with endogenous homologous template, such as the sister chromatid, homologous chromosome in the S- and G2-phases of the cell cycle. However, further studies are required to elucidate the detail mechanisms.

On the other hand, Paquet and colleagues previously introduced the ‘CORRECT’ method to establish ‘scarless’ homozygous and heterozygous mutations in an iPS cell line by implementation of consecutive re-guide or re-Cas steps (56). The requirement of two-steps and proper cut-to-mutation distance would be a restriction of the ‘CORRECT’ method for genome editing towards one-cell-stage embryos which divide rapidly. Our strategy NEO appears superior to ‘CORRECT’ from an *in vivo* perspective and exhibits higher efficiency and lower risk in unwanted indels as well.

However, there are several limitations of NEO technology. First, finding a proper pair of nicks may restrict its broad application on the entire genome. To this end, NEO, could be easily applicable to non-CRISPR gene-editing nucleases, such as ZFNs and TALENs (42); in addition, the feasibility of both *cis*- and *trans*-dual nicks in NEO system renders greater flexibility. Second, compared to CORRECT, defined homozygous and heterozygous mutations cannot be achieved through NEO. Third, the multiple RecOFAR components may result in increased difficulty for delivery *in vivo*. Fortunately, the amino acid length of individual RecOFAR is short enough to be applicable in various polycistronic technologies. Fourth, circular donor DNA with blocking cutting sites was used in this study to reduce toxicity and increase accuracy, the efficiency may be somehow sacrificed. Finally, there was low toxicity that was observed with the ectopic supply of RecOFAR given that the hatching rates and blastocyst rates were similar or higher in the RecOFAR supplemented groups than in the control group, and the founder animals or their F_1_ offspring were all healthy. We speculate that context (ssDNA–dsDNA junctions)-dependent activity of RecOFAR adopted in our NEO system and the endogenous presence of their functional counterparts, may forestall immoderate genotoxic stress after a transient implantation. In addition, the cocktail of RecFOR mediators may help to avoid inappropriate RecA recruitment on transiently-exposed ssDNA during DNA replication, thereby limiting RecA to recombination-mediated DNA repair. Nevertheless, potential negative effects of permanent or prolonged implantation of RecOFAR should be cautiously investigated in the future.

Traditional methods used DSB-mediated HR for introducing precise point mutations, and the application is limited by off-target activity and inefficiency (25–34). In contrast, base editing is a new genome-editing approach that enables the direct, irreversible conversion of one specific DNA base pair at a targeted genomic locus without double-stranded DNA cleavage (74–76). While the improved base editing methods could achieve high editing efficiency in the cultured human cells, a variety of animals, plants and even human embryos, base editor can only induce transitions but not transversions (76–81). On the contrary, NEO can be used to generate unrestricted base conversion and single-base indel (insertion/deletion) precisely and effectively via the HR-mediated approach, considering that an average of 10% KI efficiency within the zebrafish exon regions via NEO, as well as with reduced frequency of undesirable indels, given that one specific type of base editor (the cytosine base editor) was reported to generate substantial unwanted, and potentially hazardous ‘off-target’ genetic changes (82,83).

## CONCLUSION

With high efficiency and accuracy, limited undesirable indels, low toxicity, unrestricted targeting genomic regions, great flexibility of using either *trans*- or *cis*-dual nicks, easy manipulation and the conserved function of RecOFAR, the NEO method reported here adds substantially to a variety of new approaches recently developed for enhancing KI (6,9–11,21,24). Additionally, the method reported here is expected to revive the application of DNA nicks for genome editing, especially in the face of pressing demand of developing efficient and safe *in vivo* genome editing approaches. We anticipate that a favourable combination of our strategy with the advantages of others may further optimize ‘scarless’ and precise genome editing technology and lead to a broader implication. And most importantly, a more thorough mechanistic understanding of DNA repairing processes is imperative.

## DATA AVAILABILITY

Deep sequencing data were deposited in the NCBI Sequence Read Archive (SRA; http://www.ncbi.nlm.nih.gov/sra/) under accession numbers from SRR4431431 to SRR4431438, and SRR7125325.

## Supplementary Material

gkaa195_Supplemental_Files
