# Supplementary material for: Efficient and risk-reduced genome editing using double nicks enhanced by bacterial recombination factors in multiple species
Source: Nucleic Acids Res. 2020 Mar 30;48(10):e57. doi: 10.1093/nar/gkaa195 (PMC7261186; doi:10.1093/nar/gkaa195)
Supplement: gkaa195_Supplemental_Files [file gkaa195_supplemental_files.zip › corrected Final Supplementary V7 NAR-02028-Met-K-2019-R1.pdf]

Supplementary Materials for "Efficient and risk-reduced genome editing using double nicks enhanced by bacterial recombination factors in multiple Species".

Authors : Xiaozhen He[1]*, Wenfeng Chen[1]*, Zhen Liu[2]*, Guirong Yu[1#], Youbang Chen[1#], Yi-Jun Cai[2#], Ling Sun[1#], Wanli Xu[1], Lili Zhong[1], Caixi Cao[1], Jishen Chen[1], Minjie Zhang[1], Shengxi Yang[1], Yizhou Yao[1], Zhiping Zhang[1], Fujun Ma[1], Chen-Chen Zhang[2], Hui-Ping Lu[2], Bin Yu[2], Tian-Lin Cheng[2], Juhui Qiu[3], Qing Sheng[4], Hai-Meng Zhou[4,5], Zhi-Rong Lv[5], Junjun Yan[6], Yongjian Zhou[7], Zilong Qiu[2], Zongbin Cui[6], Xi Zhang[1], Anming Meng[3], Qiang Sun[2 †], Yufeng Yang[1 †]

Supplementary Materials includes seventeen figures, eight tables, twenty-two texts, two excels, and one movie.

Supplementary Figure S1. Schemes for donor cloning and sgRNA synthesis.
Supplementary Figure S2. sgRNAs-mediated cleavage of target loci with recombinant Cas9 protein *in vitro*.
Supplementary Figure S3. PCR and sequencing results of KI events in zebrafish.
Supplementary Figure S4. Photo-conversion experiment with *Ndr2* and *Lefty2* KI-positive embryos.
Supplementary Figure S5. Detection of off-target indels from the zebrafish *Ndr2* locus by T7EI cleavage assay.
Supplementary Figure S6. On-target indels and off-target events were reduced significantly through NEO.
Supplementary Figure S7. Negligible toxicity of ectopic RecOFAR in mouse embryos and Next-Gen-based multiplexed sequencing indicated reduced on-target indels at the mouse *Slc6a4* locus through NEO.
Supplementary Figure S8. Immunostaining of WT and Slc6a4-p2A-ChR2-EYFP F_1 mice with anti-5HT.
Supplementary Figure S9. Immunostaining of WT and Slc6a4-p2A-ChR2-EYFP F_1 mice with anti-5HTT.
Supplementary Figure S10. Assessment of NEO (*trans*-dual nicks)-mediated on-target and off-target cleavage of one *Slc6a4* KI founder by T7EI assay.
Supplementary Figure S11. Assessment of NEO (*cis*-dual nicks)-mediated on-target and off-target cleavage of one *Slc6a4* KI positive founder by T7EI assay.
Supplementary Figure S12. Immunostaining of KI positive rats.
Supplementary Figure S13. PCR genotyping in individual monkey embryos with p2A-ChR2::EYFP knock-in at the *CAMK2A* locus.
Supplementary Figure S14. PCR genotyping in individual monkey embryos with knock-in EGFP-2A-NeoR-2A at the *Oct4* locus.
Supplementary Figure S15. The unstimulated primary human PBLCs shows low dividing ability.
Supplementary Figure S16. Genotype, phenotype and on-target indel analyses of gene KI at the human *Rpl41* locus.
Supplementary Figure S17. Genotype, phenotype and on-target indel analyses of gene KI at the human *TUFm* locus.

Supplementary Table S1. Summary of DNA integration studies using TALENs and CRISPR/Cas9 in zebrafish.

Supplementary Table S2. Primers used for donor DNA construction.
Supplementary Table S3. Oligonucleotides used for generating templates for *in vitro* transcription of sgRNAs.
Supplementary Table S4. Primers used for nest PCR.
Supplementary Table S5. Primers used for off-target analysis.
Supplementary Table S6. NEO system enables highly efficient homologous recombination in zebrafish and mammals – Summary Table
Supplementary Table S7. Toxicity assessment of RecOFAR mRNAs, CRISPR/Cas9 ingredients and donor DNA in zebrafish embryos.
Supplementary Table S8. The mosaicism of germline transmission in the founder fish, mice and rats produced by NEO system.

Supplementary Text S1. Sequence of the zebrafish *gfap* targeting construct *p2A-ChR2-EYFP*.
Supplementary Text S2. Sequence of the zebrafish *Lefty2* targeting construct *linker-Dendra2*.
Supplementary Text S3. Sequence of the zebrafish *Ndr2* targeting construct *linker-Dendra2*.
Supplementary Text S4. Sequence of the zebrafish *Bmp2b* targeting construct *linker-Dendra2-linker*.
Supplementary Text S5. Sequence of the zebrafish *gfap* targeting construct *p2AV1-NpHR3.0-EYFP-p2AV2-hChR2-mCherry-IRES-WGA-Cre*.
Supplementary Text S6. Sequence of the mouse *Slc6a4* targeting construct *p2A-ChR2-EYFP*.
Supplementary Text S7. Sequence of the mouse *Myc* targeting construct *linker-sfGFP*.
Supplementary Text S8. Sequence of the rat *GFAP* targeting construct *p2A-mEYFP*.
Supplementary Text S9. Sequence of the rat *Drd1* targeting construct *p2A-ChR2-EYFP*.
Supplementary Text S10. Sequence of the rat *Drd2* targeting construct *p2A-ChR2-EYFP*.
Supplementary Text S11. Sequence of the rat *Bassoon* targeting construct *p2A-NpHR-EYFP-p2A-ChR2-mCherry-IRES-WGA-Cre* donor plasmid (*Bassoon*-5.5 kb).
Supplementary Text S12. Sequence of monkey *CAMK2A* targeting construct *p2A-ChR2::EYFP* donor plasmid.
Supplementary Text S13. Sequence of monkey *Oct4* targeting construct *EGFP-p2A-NeoR-p2A* donor plasmid.
Supplementary Text S14. Sequence of the human *Rpl41* targeting construct *hRpl41-IRES-mito-EGFP*.
Supplementary Text S15. Sequence of the human *EFTu* targeting construct *hEFTu-IRES-mito-mCherry*.
Supplementary Text S16. Sequence of Cas9n transcript template.
Supplementary Text S17. Sequence of Cas9 transcript template.
Supplementary Text S18. Sequence of RecA transcript template
Supplementary Text S19. Sequence of RecF transcript template.
Supplementary Text S20. Sequence of RecO transcript template.
Supplementary Text S21. Sequence of RecR transcript template.
Supplementary Text S22. Sequence for TagBFP transcript template.

Supplementary Excel S1. *GFAP* On-target deep-sequencing.
Supplementary Excel S2. *Slc6a4* On-target deep-sequencing.

Supplementary Movie S1. Representative heart beating movie of *Lefty2-linker-Dendra2* positive fish at 36 h post-fertilization.

Supplementary Figure S1

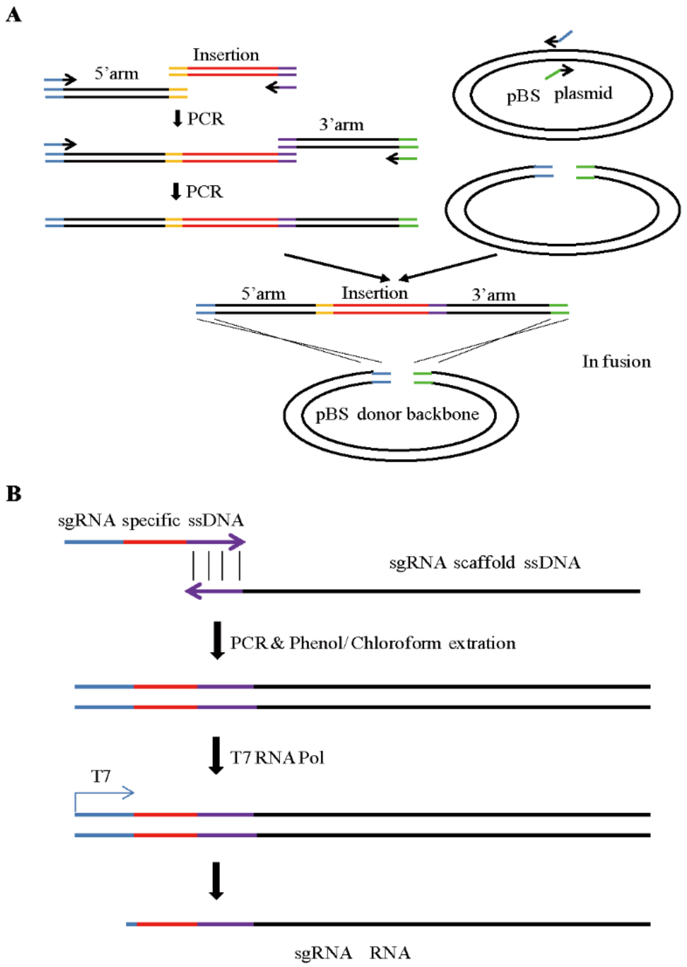

Supplementary Figure S1. Schemes for donor cloning and sgRNA synthesis. (A) Overlap-extension PCR strategy and In-Fusion cloning enzyme were used to clone 5'HA-Insertion-3'HA into pBluescript backbone. (B) sgRNA transcription templates were produced by direct extension of gene-specific primers and a sgRNA scaffold primer.

Supplementary Figure S2

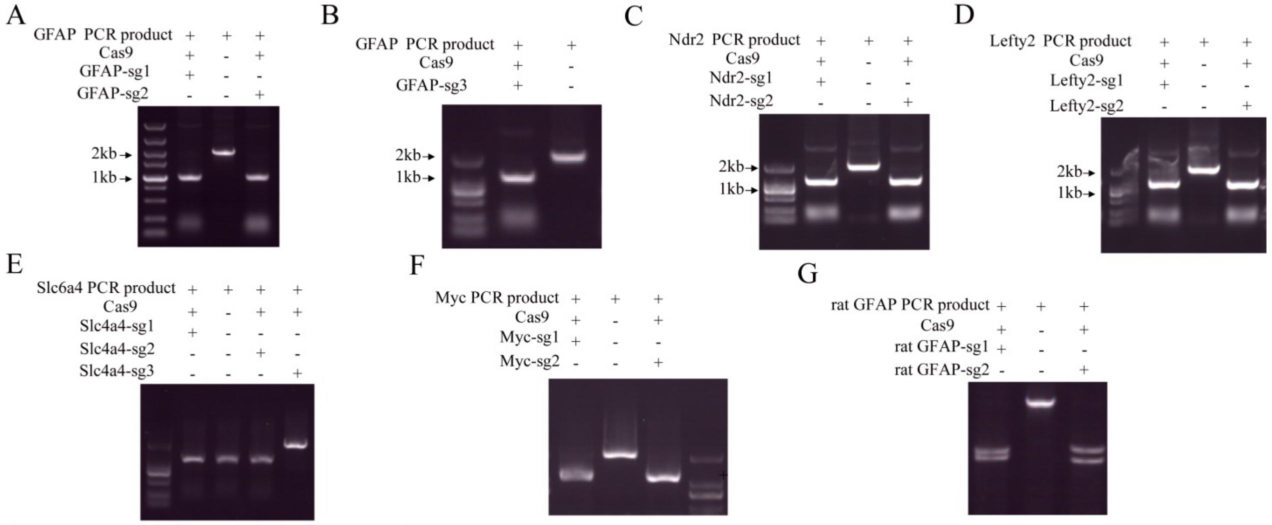

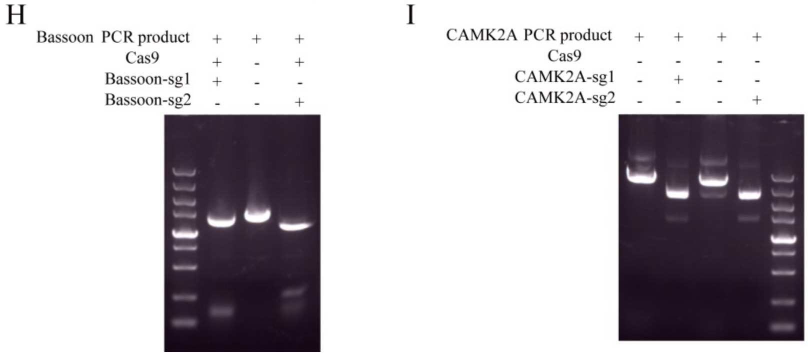

J

	sgRNA1	sgRNA2
Drd2	5/10	3/14
Drd1	3/9	15/18
Oct4	4/5	4/6

Supplementary Figure S2. SgRNAs-mediated cleavage of target loci with recombinant Cas9 protein *in vitro* or *in vivo*. For fish *gfap* (A, B), fish *Ndr2* (C), fish *Lefty2* (D), mouse *Slc6a4* (E), mouse *Myc* (F), rat *gfap* (G), rat *Bassoon* (H) locus, Monkey *CAMK2A* (I) locus, PCR products containing sgRNA targeting sites were used as the substrate for the *in vitro* cleavage assay. (J) For rat *Drd2*, *Drd1* and Monkey *Oct4* loci, Cas9 mRNA and sgRNAs were injected into embryos. Fragments flanking the target site from each embryo were amplified by PCR, and were further used to identify indels by sequencing. Ratio of embryos with indels versus total embryos is shown.

Supplementary Figure S3

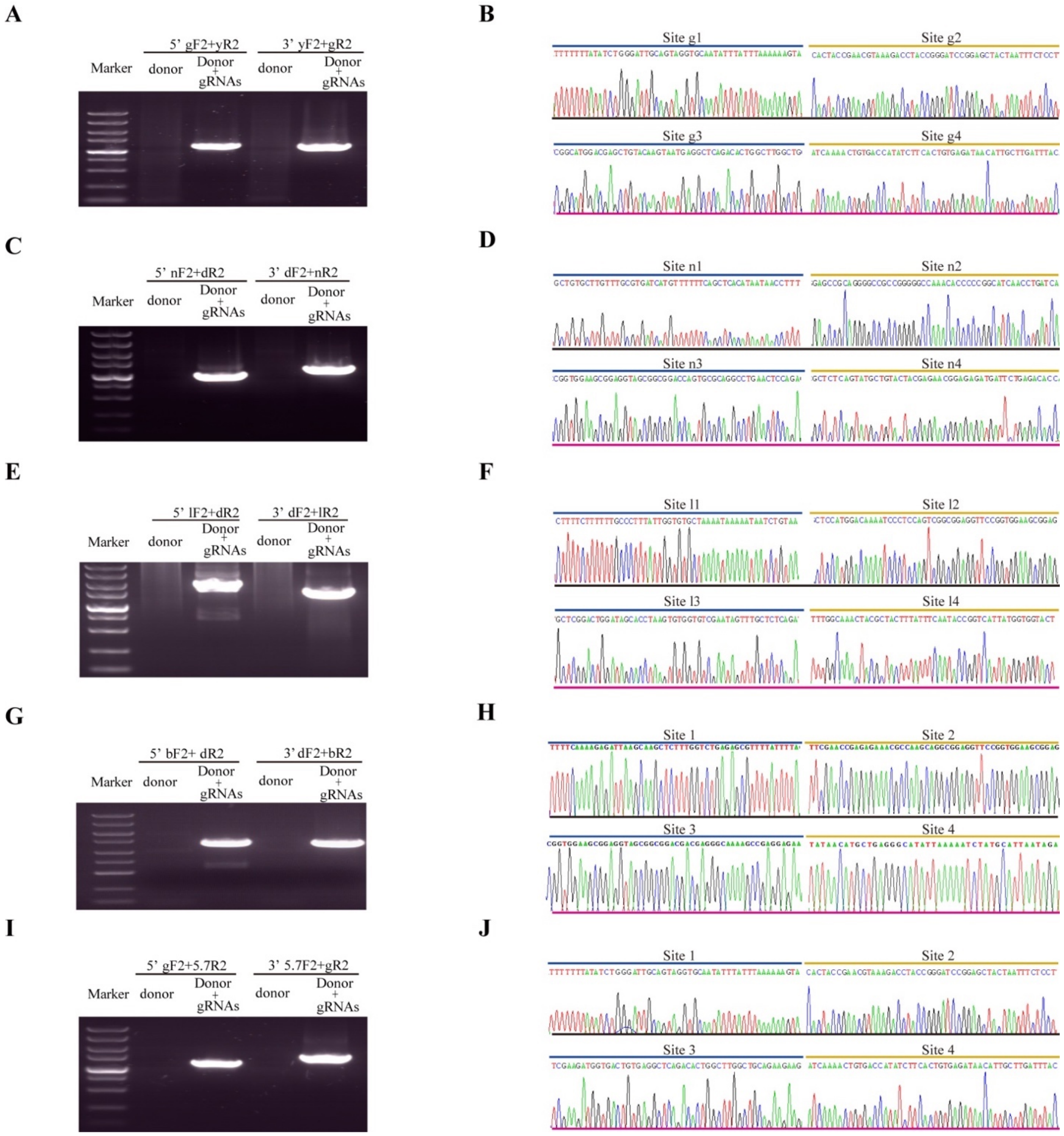

Supplementary Figure S3. PCR and sequencing results of KI events in zebrafish. (A) PCR analysis of the 5′ and 3′ integration junctions at the *gfap* locus in the p2A-ChR2-EYFP KI positive F_1 fish. The primers used are shown in **Figure 1B** and **Supplementary Table S4**. (B) Representative sequencing results of the *gfap* locus in EYFP-positive zebrafish. (C) PCR analysis of the 5′ and 3′ integration junctions at the *Ndr2* locus in linker-dendra2-V5 KI-positive F_1 fish. The primers used are shown in **Figure 2A** and **Supplementary Table S4**. (D) Representative sequencing results of the *Ndr2* locus in dendra2-positive zebrafish. (E) PCR analysis of the 5′and 3′integration junctions at the *Lefty2* locus in the linker-dendra2-V5 KI positive F_1 fish. The primers used are shown in **Figure 2E** and **Supplementary Table S4.** (F) Representative sequencing results of the *Lefty2* locus in dendra2-positive zebrafish. (G) PCR analysis of the 5′ and 3′ integration junctions at the *Bmp2b* locus in the linker-dendra2-V5-linker KI-positive F_1fish. The primers used are shown in **Figure 2H** and **Supplementary Table S4**. (H) Representative sequencing results of the *Bmp2b* locus in dendra2-positive zebrafish. (I) PCR analysis of the 5′ and 3′ integration junctions at the *gfap* locus in the 5.5 kb KI-positive F_1

fish. The primers used are shown in **Figure 2K** and **Supplementary Table S4**. (J) Representative sequencing results of zebrafish with 5.5 kb KI at the *gfap* locus.

Supplementary Figure S4

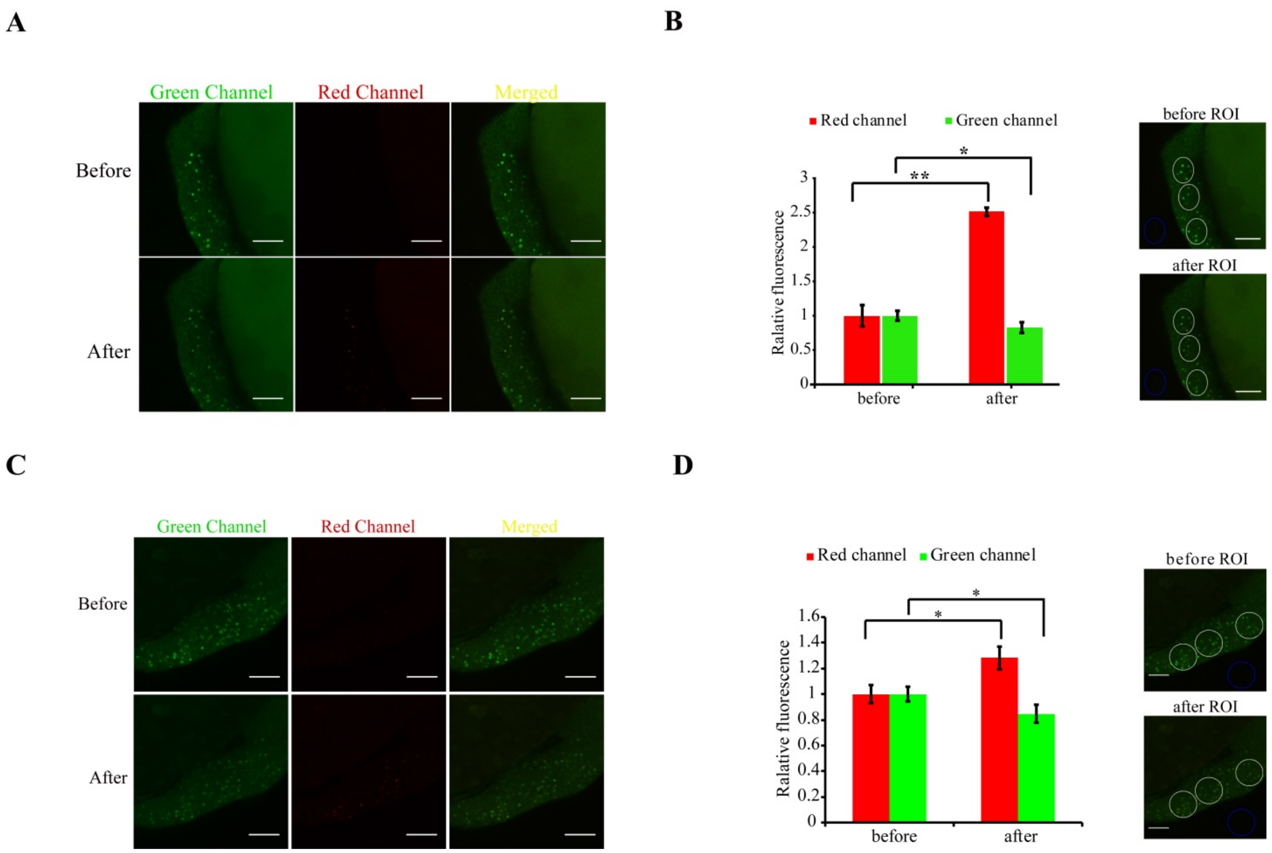

Supplementary Figure S4. Photo-conversion experiment with *Ndr2* and *Lefty2* KI-positive embryos. (A) Photo-conversion experiment with *Ndr2* KI-positive embryos from F_2 generation. Scale bar, 50 µm. (B) Relative fluorescence intensity before or after photo-conversion. Vertical bars represent means. *P<0.05. Error bars are s.d. Scale bar, 50 µm. (C) Photo-conversion experiment with *Lefty2* KI-positive embryos from F_2 generation. Scale bar, 50 µm. (D) Relative fluorescence intensity before or after photo-conversion. Vertical bars represent means. **P<0.01. *P<0.05. Error bars are s.d. Scale bar, 50 µm. Confocal images were obtained at an optical section thickness of 1-2µm. A 405nm (0.75mW) laser was used to convert Dendra2 from green to red fluorescence for 10-20s. Fluorescence intensity was quantified with LAS AF lite software (Leica).

Supplementary Figure S5

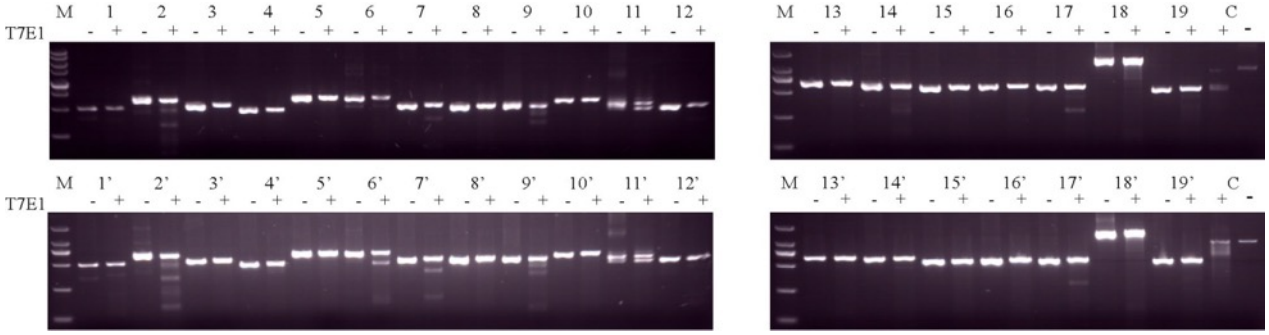

Supplementary Figure S5. Detection of NEO-mediated off-target events of *Ndr2* by T7EI cleavage assay. PCR products of potential sgRNA targeting sites from Ndr2-linker-dendra2-V5 target KI (Cas9n+NEO) founder fish were subjected to T7EI cleavage assay. Lane number 1-2 were for potential off-target sites of Ndr2-sgRNA1. Lane number 3-19 were for potential off-target sites of Ndr2-sgRNA2. Lane number 1'-19' referred to another founder. The OTS that yielded typical bands pattern of cleavage were considered as candidates, then the PCR products of the candidates were cloned and sequenced to identify the off-target events and single nucleotide polymorphisms (SNPs) were excluded.

Supplementary Figure S6

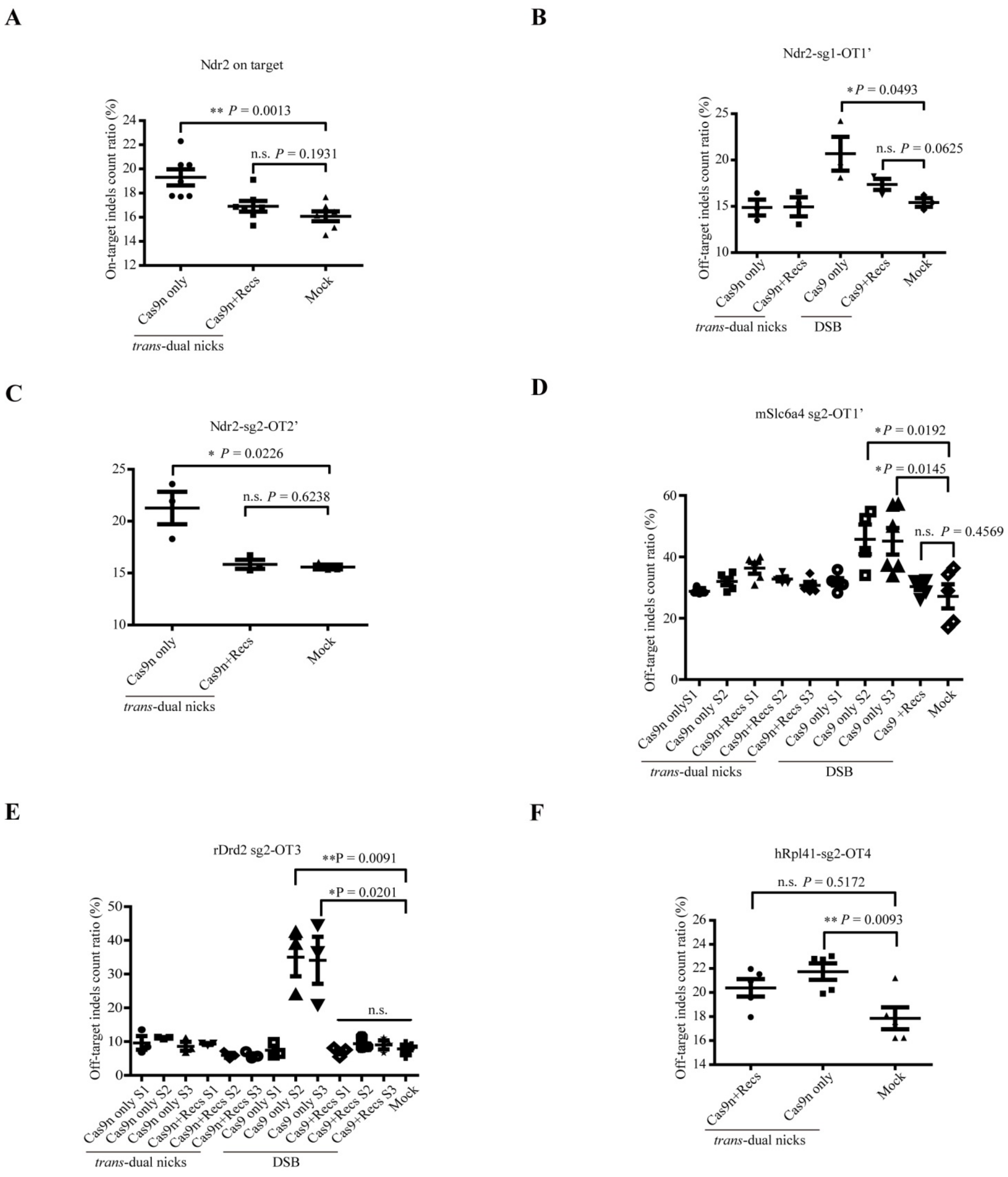

Supplementary Figure S6. On-target indels and off-target events were reduced significantly through NEO. Detection of on-target indels at the zebrafish *Ndr2* locus and representative off-target detection results of zebrafish *Ndr2*, mouse *Slc6a4*, rat *Drd2* and human *Rpl41* by IDAA. (A) Ndr2-on-target detection. (B) Ndr2-sg1-OT1' detection. (C) Ndr2-sg2-OT2' detection. (D) mSlc6a4-sg2-OT1' detection. Tails of KI-positive F_0 individuals with different injection recipes were subjected to analysis. Total indel count ratio was calculated from peak areas of the summed indel peaks relative to the total peak area. Each sample was tested more than three times. (E) rDrd2-sg2-OT3 detection. Tails of F_0 individuals with different injection recipes (all KI-positive individuals and some non-KI-positive individuals) were subjected to analysis. (F) Rpl41-sg2-OT4

detection. Results were obtained from at least three samples from independent electroporation experiments, each sample was tested more than three times. Mock, control group with mock treatment. All results were presented with Mean±S.D. Comparisons between groups were evaluated by Student's *t*-test. n.s., no significant difference. *P < 0.05, **P < 0.01.

Supplementary Figure S7

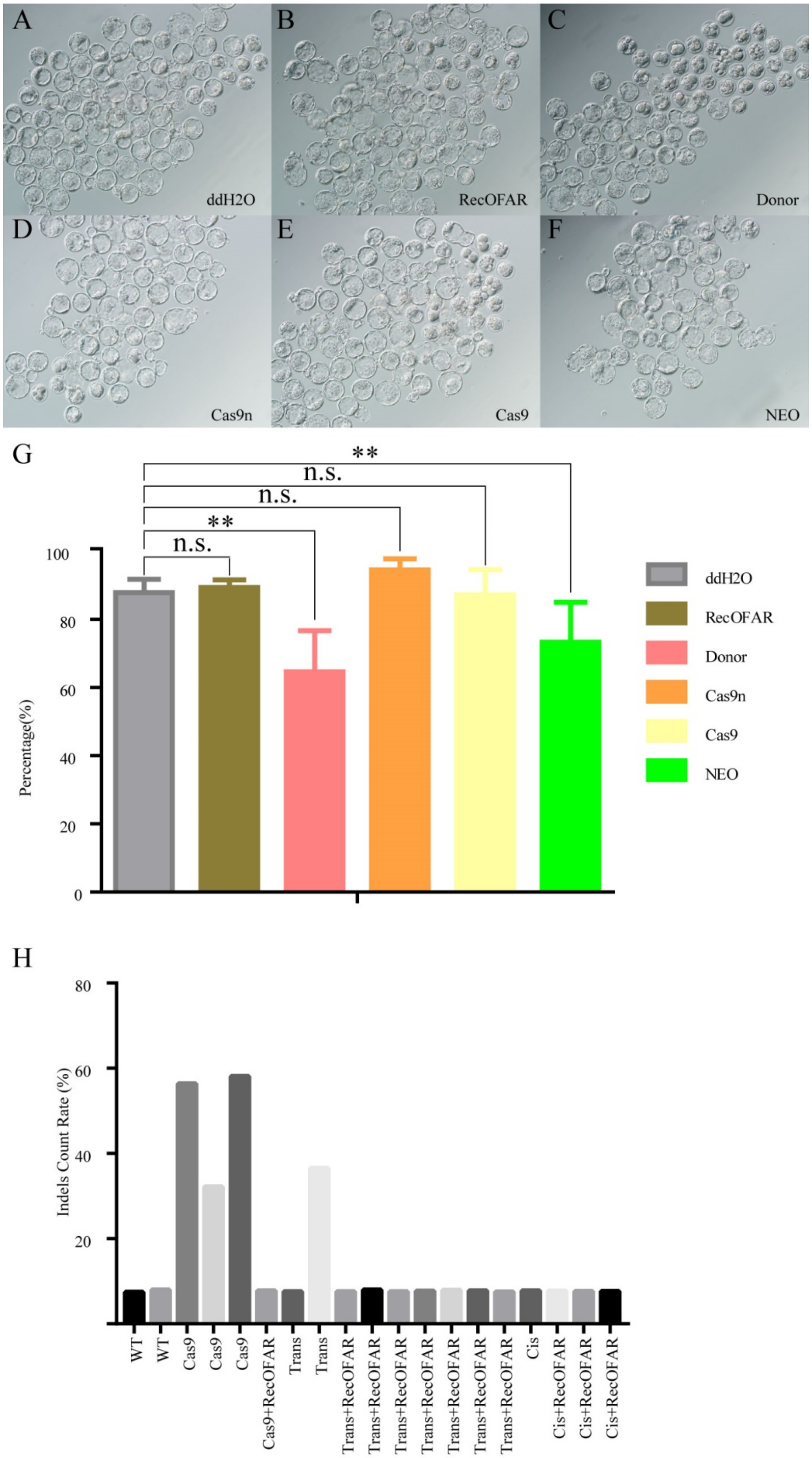

Supplementary Figure S7. Negligible toxicity of ectopic RecOFAR in mouse embryos and Next-Gen-based multiplexed sequencing indicated reduced on-target indels at the mouse *Slc6a4* locus through NEO.

(A-F) The viability of embryos developed to blastocyst stage after injection of different recipes was examined. Representative images of developing embryos are shown. In the NEO injection group, the donor of Slc6a4-p2A-chR2-EYFP, Slc6a4-sgRNA1 and Slc6a4-sgRNA2 were used. (G) Percentage of embryos that developed to blastocyst stage. Two independent microinjection experiments were conducted. Chi-square with Fisher's test was performed. n.s., no significant difference. **, indicates significant difference. Error bars are s.e.m. (H) On-target indels analysis using Next-Gen-based multiplexed sequencing at the *Slc6a4* locus. Results indicated a significant reduction of on-target indels through NEO. Tails of KI-positive F_0 individuals with different injection recipes were subjected to analysis.

Supplementary Figure S8

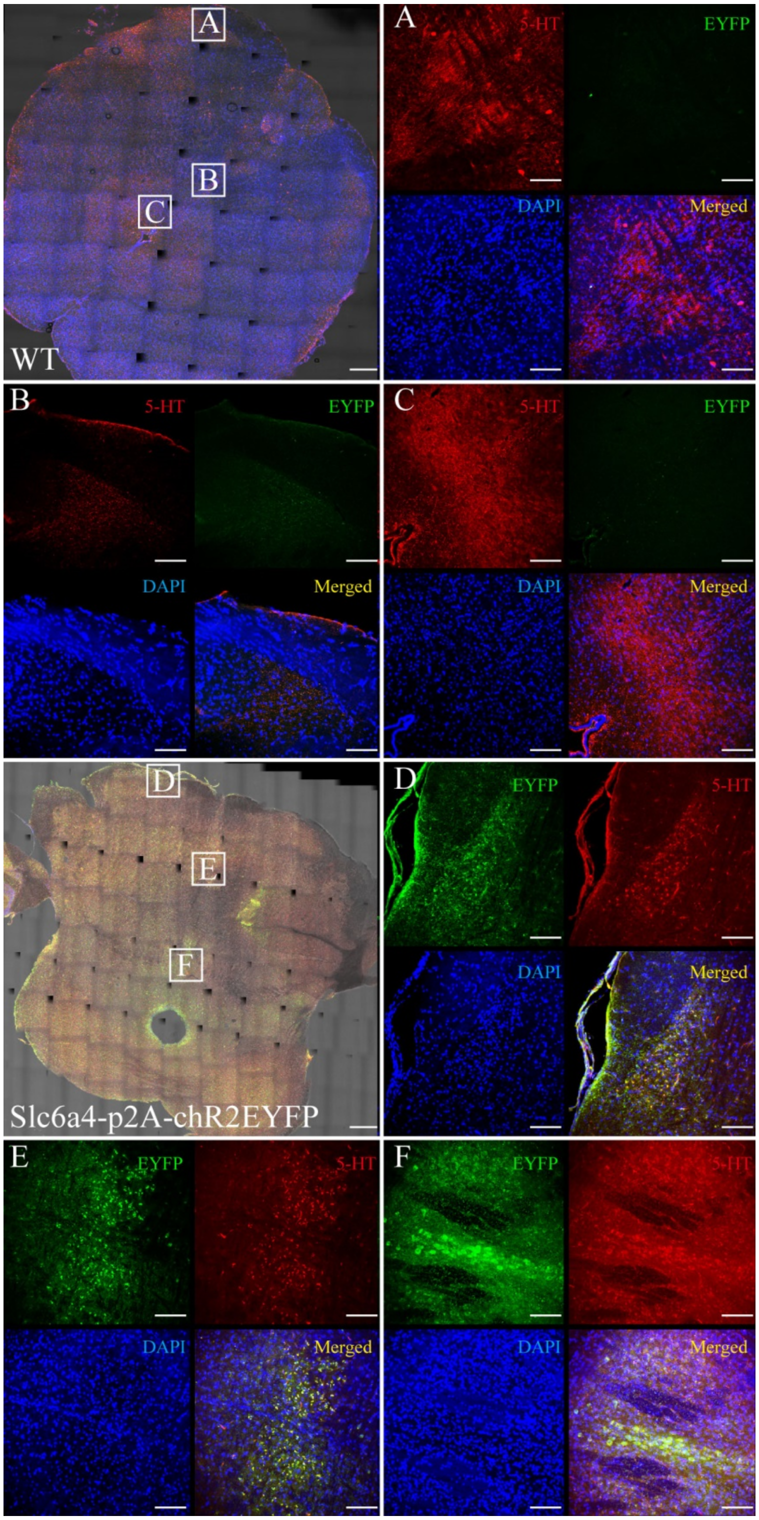

Supplementary Figure S8. Immunostaining of WT and Slc6a4-p2A-ChR2-EYFP F_1 mice with anti-5HT. Representative whole mount brain sections are shown (Scale bar=500 µm). The regions indicated in the boxes of whole mount sections are magnified in the other panels (Scale bar=100 µm). ChR2-EYFP is in green and cells marked with anti-Serotonin (5-Hydroxytryptamine, 5-HT) are in red. Nuclei in blue were stained with DAPI. (A-C) WT. (D-F) Slc6a4-p2A-ChR2-EYFP F_1 mice.

Supplementary Figure S9

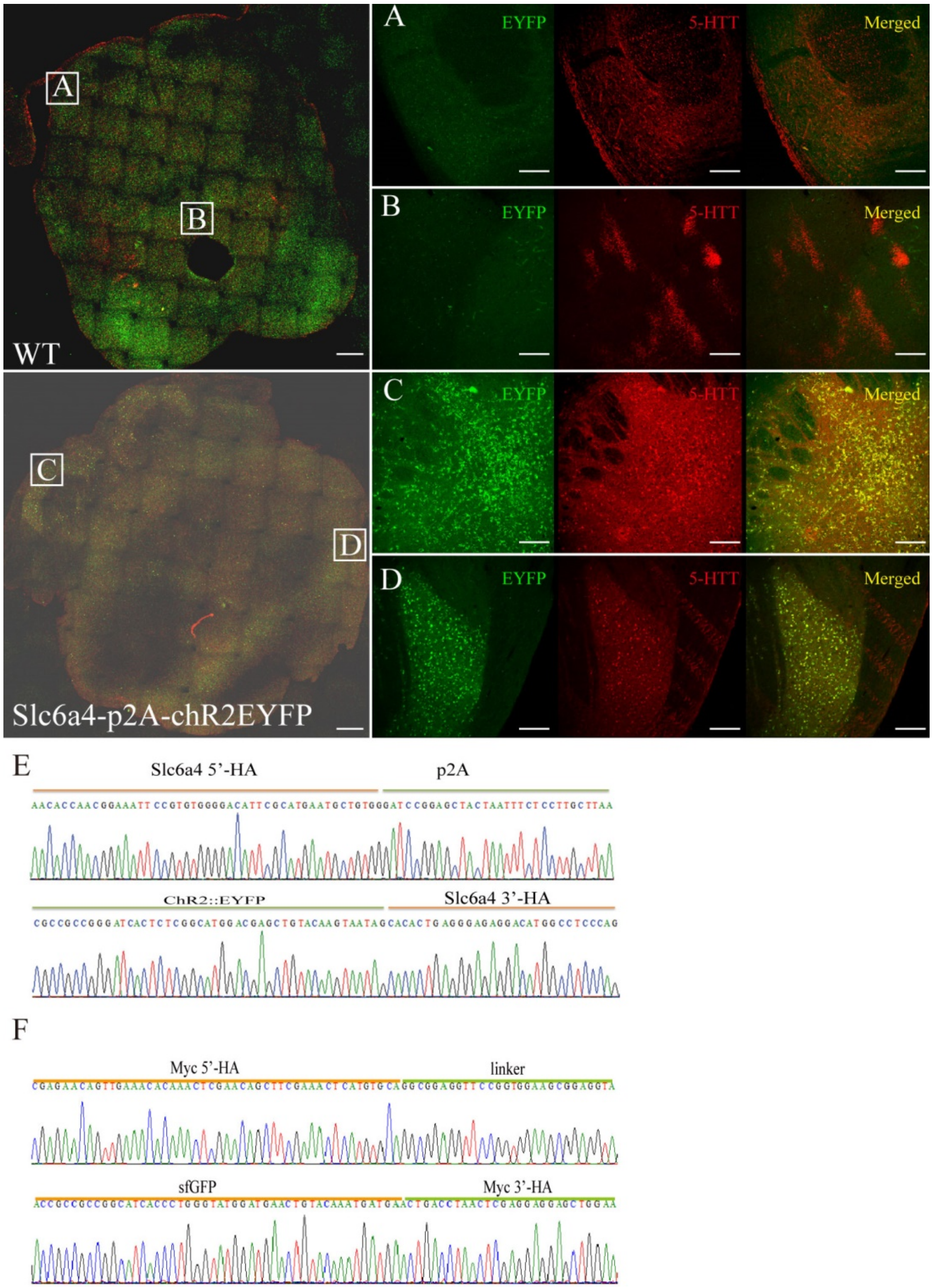

Supplementary Figure S9. Immunostaining of WT and Slc6a4-p2A-ChR2-EYFP F_1 mice with anti-5HTT. Representative whole mount brain sections are shown in the left panels (Scale bar=500 µm). The regions indicated in the boxes of whole mount sections are magnified in the right panels (Scale bar=100 µm). ChR2-EYFP is in green and cells marked with anti-5HTT (SLC6A4) are in red. (A, B) WT, (C, D) Slc6a4-p2A-ChR2-EYFP F_1 mice. (E, F) Representative sequencing results of mice with p2A-ChR2-EYFP and sfGFP KI at the *Slc6a4* and *Myc* loci.

Supplementary Figure S10

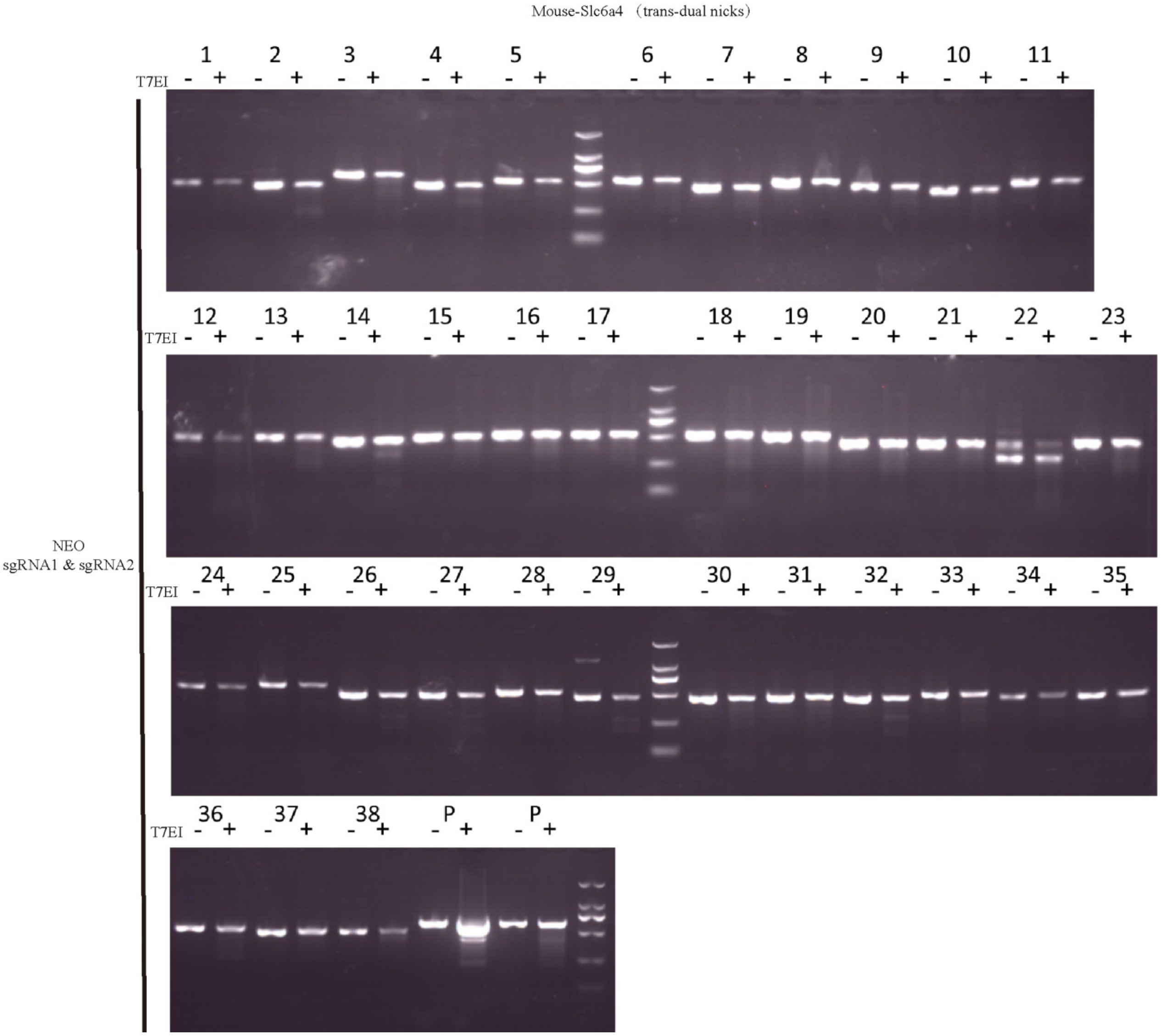

Supplementary Figure S10. Assessment of NEO (*trans*-dual nicks)-mediated on-target and off-target cleavage of one *Slc6a4* KI positive founder by T7EI assay. PCR products containing potential sgRNA targeting sites were subjected to T7EI cleavage assay. Lane number 1 is for the on-target site. Lane number 2-12 are for the potential off-target sites of sgRNA1. Lane number 13-38 are for potential off-target sites of sgRNA2. The OTS that yielded typical bands pattern of cleavage were considered as candidates, then the PCR products of the candidates were cloned and sequenced to identify the off-target events. All of the candidates were found with single nucleotide polymorphisms (SNPs) but not with true off-target cleavage.

Supplementary Figure S11

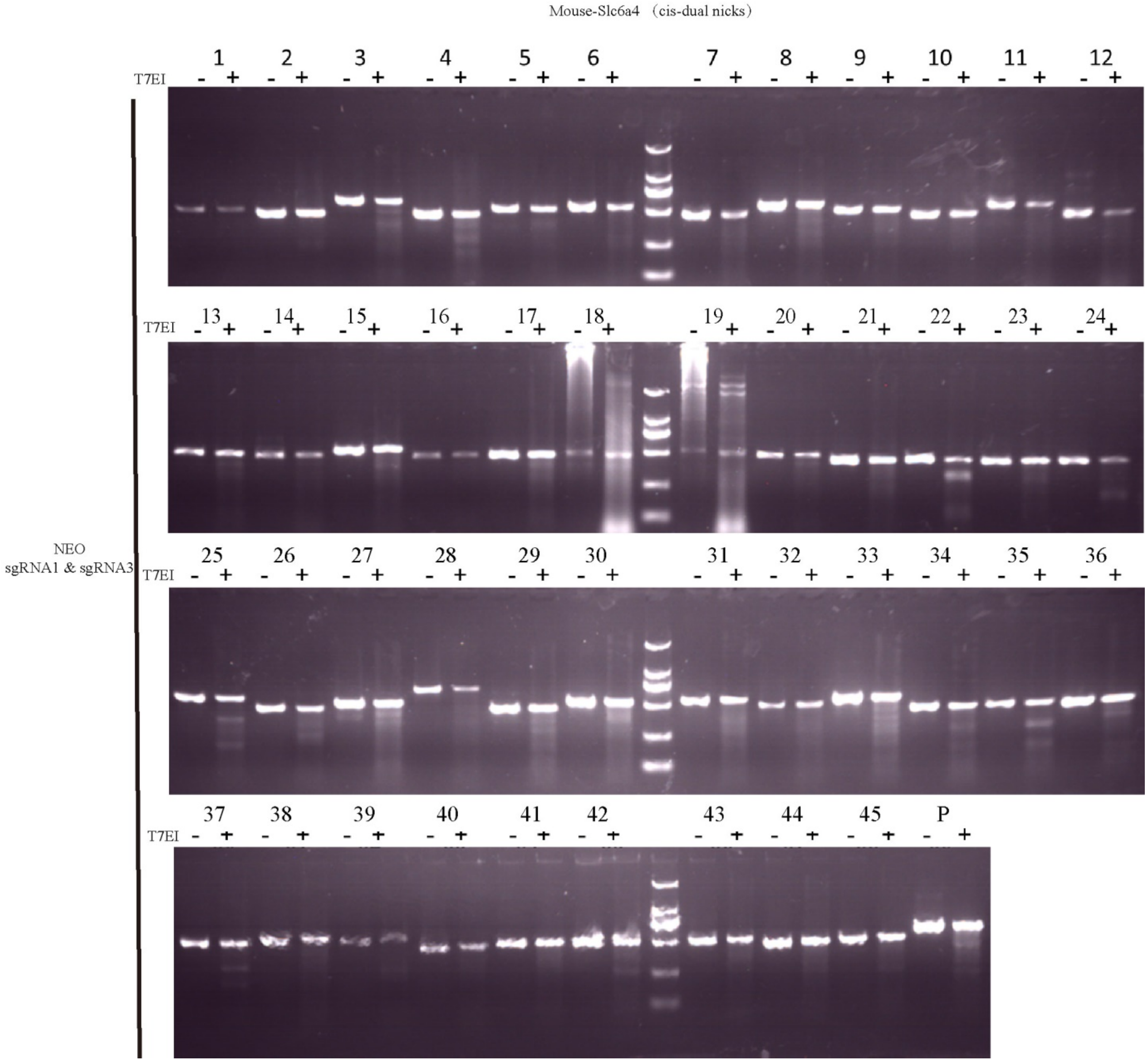

Supplementary Figure S11. Assessment of NEO (*cis*-dual nicks)-mediated on-target and off-target cleavage of one *Slc6a4* KI positive founder by T7EI assay. PCR products containing potential sgRNA target sites were subjected to T7EI cleavage assay. Lane number 1 is for the on-target site. Lane number 2-12 are for the potential off-target sites of sgRNA1. Lane number 13-45 are for the potential off-target sites of sgRNA3. The OTS that yielded typical bands pattern of cleavage were considered as candidates, then the PCR products of the candidates were cloned and sequenced to identify the off-target events. All of the candidates were found with single nucleotide polymorphisms (SNPs) but not with true off-target cleavage.

Supplementary Figure S12

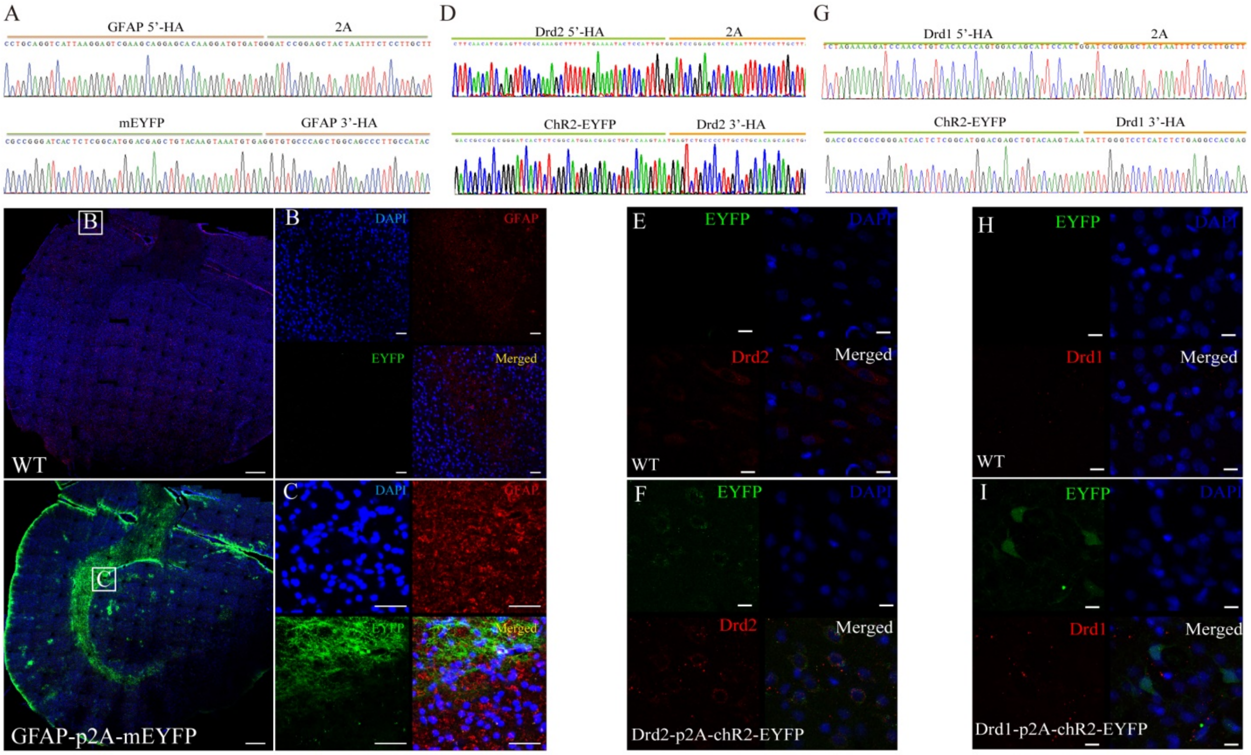

Supplementary Figure S12. Immunostaining of KI-positive rats. (A) Representative sequencing results of rat with p2A-mEYFP KI at the *gfap* locus. (B-C) Immunostaining of GFAP-p2A-mEYFP F_1 rats. The whole mount brain sections are shown in the left panels (Scale bar=500 µm). The regions indicated in the boxes of whole mount sections are magnified in the right panels. Neurons marked with NeuN are in red, mEYFP is in green and glial cells marked with anti-GFAP are in white. Scale bar, 50 µm. (D) Representative sequencing results of rats with p2A-chR2-EYFP KI at the *Drd2* loci. (E-F) Immunostaining with anti-GFP and anti-Drd2 of WT, *Drd2*-p2A-chR2-EYFP F_1 rats. Scale bar, 10 µm. (G) Representative sequencing results of rats with p2A-chR2-EYFP KI at the *Drd1* loci. (H-I) Immunostaining with anti-GFP and anti-Drd1 of WT and *Drd1*-p2A-chR2-EYFP F_1 rats. Scale bar, 10 µm.

Supplementary Figure S13

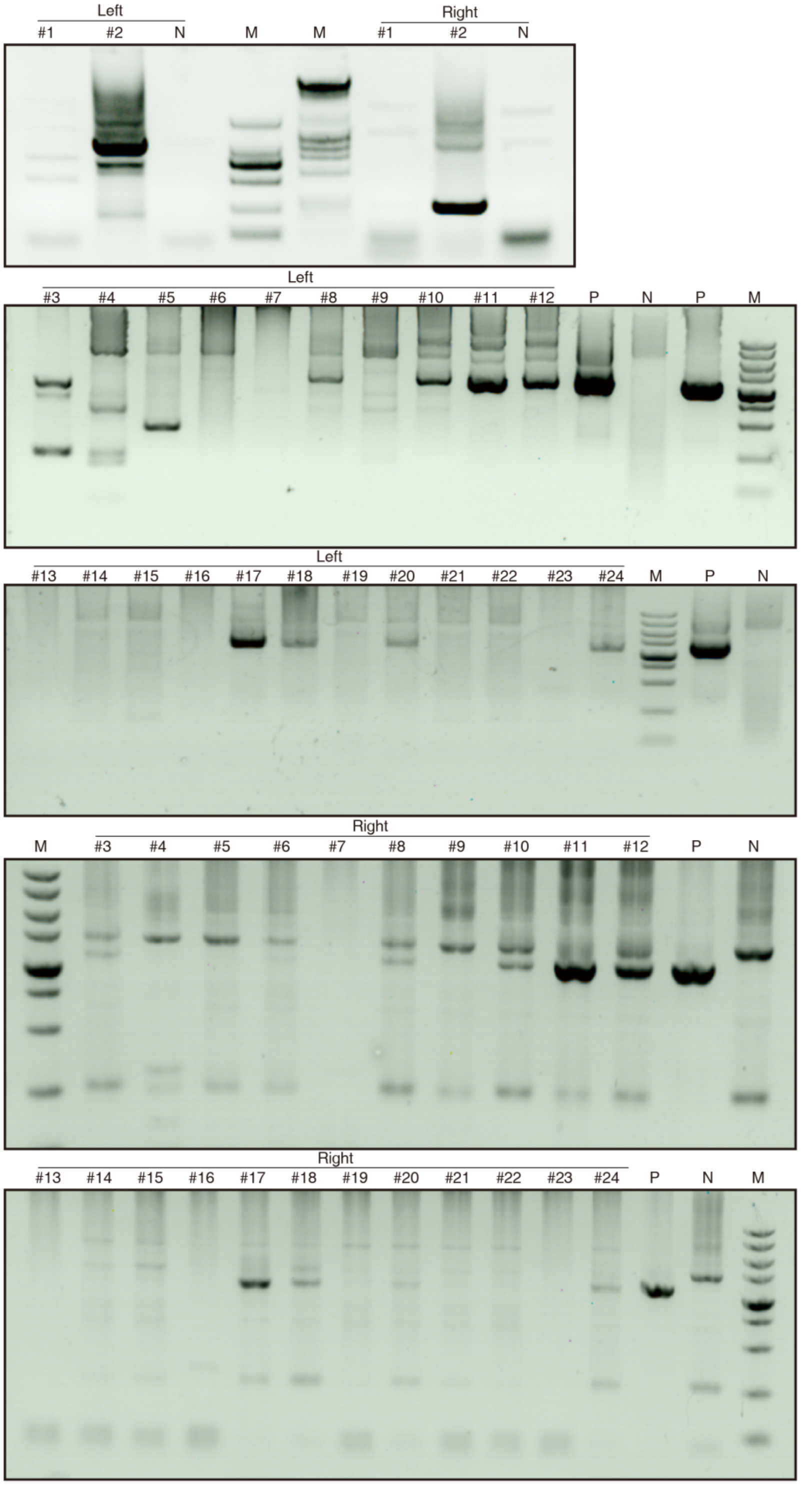

Supplementary Figure S13. PCR genotyping in individual monkey embryos with p2A-ChR2::EYFP knock-in at the *CAMK2A* locus. Left (or right) indicates the PCR products corresponding to the junctional region between the 5' (or 3') arm and the host genome. The PCR products with approximately 1.0 kb were amplified in 10 samples (#2, #3, #8, #10, #11, #12, #17, #18, #20, #24) and were identified by sequencing. The genomic DNA extraction of 4 samples (#7, #13, #16, #23) was failed. Thus, the percent of positive was calculated as 10/20. P, positive control is used to indicate the right position; N, negative control; M, marker.

Supplementary Figure S14

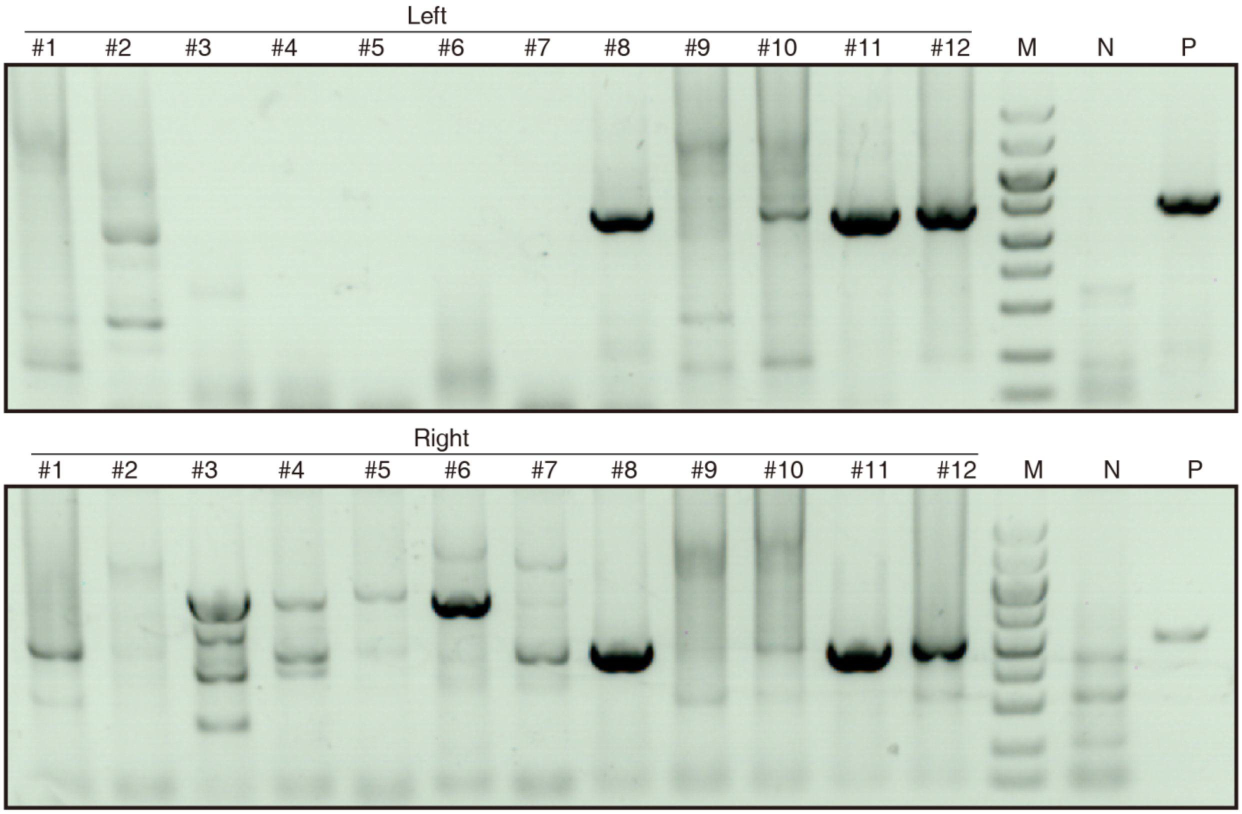

Supplementary Figure S14. PCR genotyping in individual monkey embryos with knock-in EGFP-2A-NeorR-2A at the *Oct4* locus. Left (or right) indicates the PCR products corresponding to the junctional region between the 5' (or 3') arm and the host genome. The PCR products with approximately 1.0 kb were amplified in 3 samples (#8, #11, #12). P, positive control is used to indicate the right position; N, negative control; M, marker.

Supplementary Figure S15

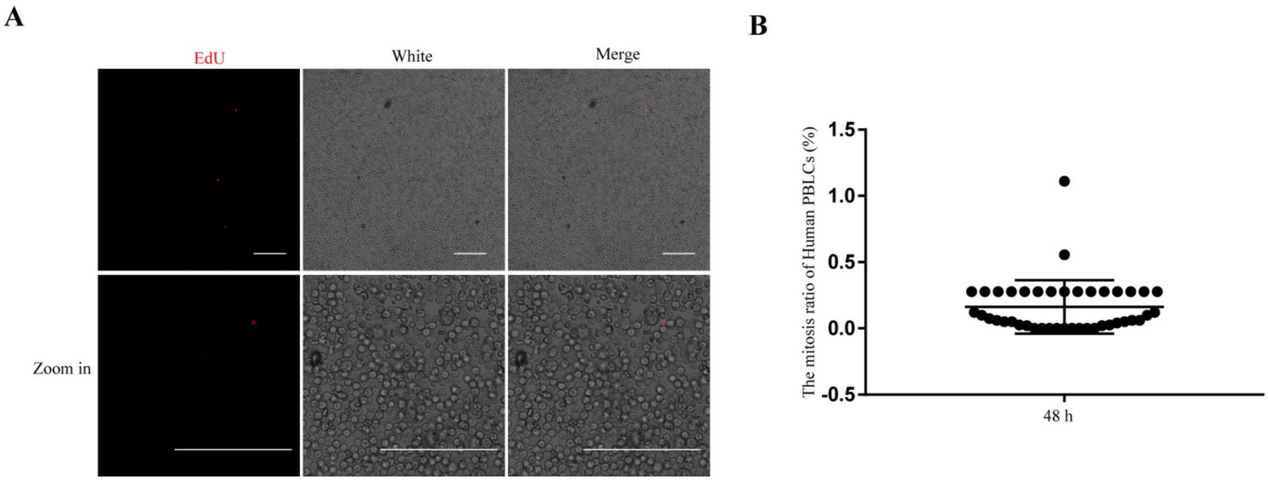

Supplementary Figure S15. The unstimulated primary human PBLCs shows low dividing ability. (A) EdU staining results of the Human PBLCs at 48h after electroporation. Human PBLCs were incubated with EdU for 48h. EdU specifically labels S phase cells. Scale bar in left panel is 100 µm. (B) Ratio of human PBLCs in S phase (mean 0.16%).

Supplementary Figure S16

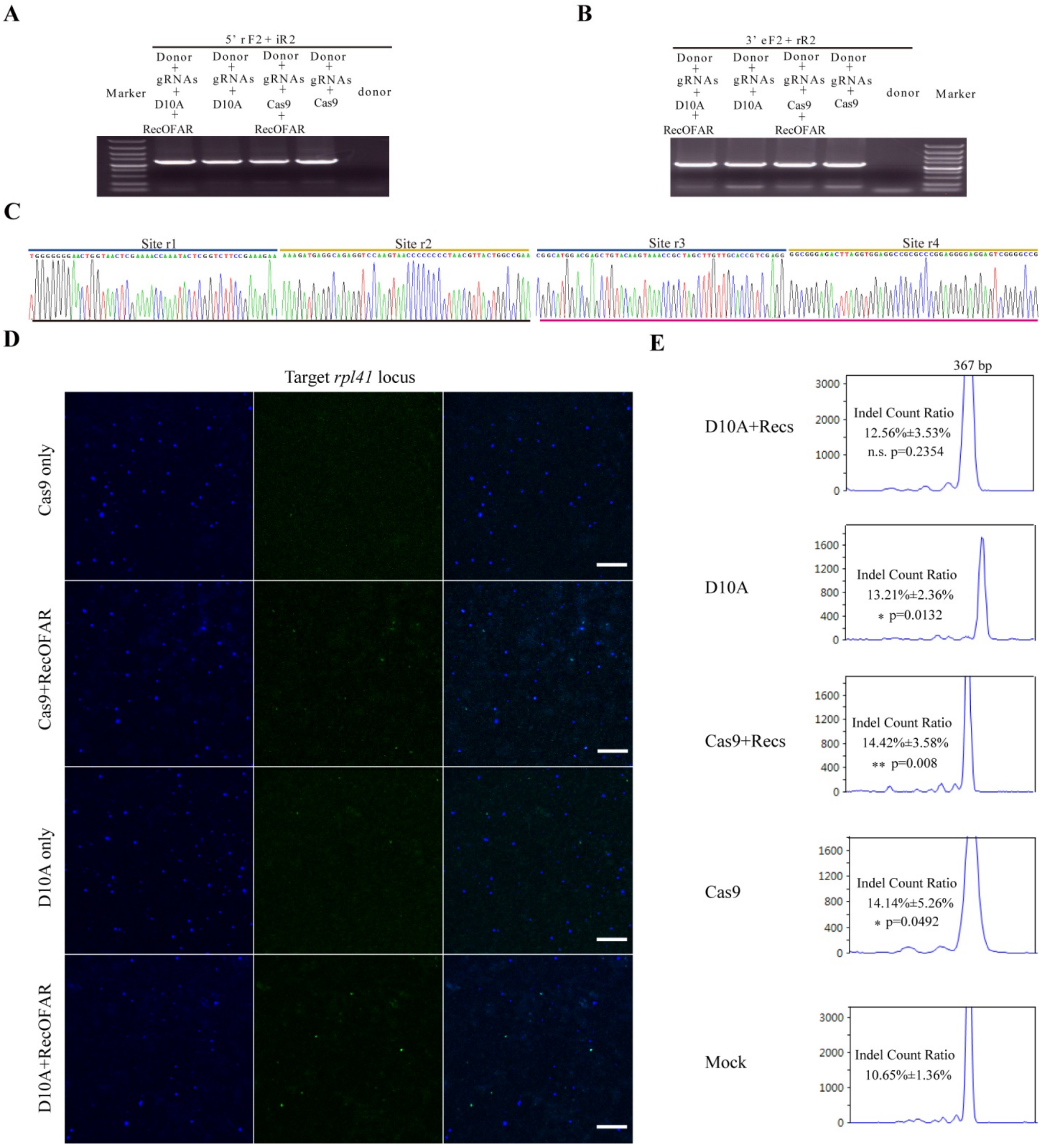

Supplementary Figure S16. Genotype, phenotype and on-target indel analyses of gene KI at the human *Rpl41* locus. (A) PCR analysis of the 5′ junctions at the *Rpl41* locus. The primers used are shown in Fig 6A. (B) PCR analysis of the 3′ junctions at the *Rpl41* locus. The primers used are shown in Fig 6A. (C) Representative sequencing results of primary human PBLCs cells with IRES-mito-EGFP KI at the *Rpl41* locus. (D) EGFP fluorescence signal in the Rpl41-IRES-mito-EGFP KI-positive cell. Blue, TagBFP transfection positive cells. Scale bar, 100 μm. (E) On-target analysis by IDAA at the *Rpl41* locus. In sharp contrast to the wtCas9, wtCas9+RecOFAR or Cas9n only groups that all resulted in obvious undesired on-target indels, NEO system did not generate discernible on-target indels. Total indel count ratio was calculated from peak areas of the summed indel peaks relative to the total peak area. Mock, control group with mock treatment. Results were

presented with Mean±S.D. Comparisons between groups and mock were evaluated by Student's *t*-test. n.s., no significant difference. *$P < 0.05$, **$P < 0.01$.

Supplementary Figure S17

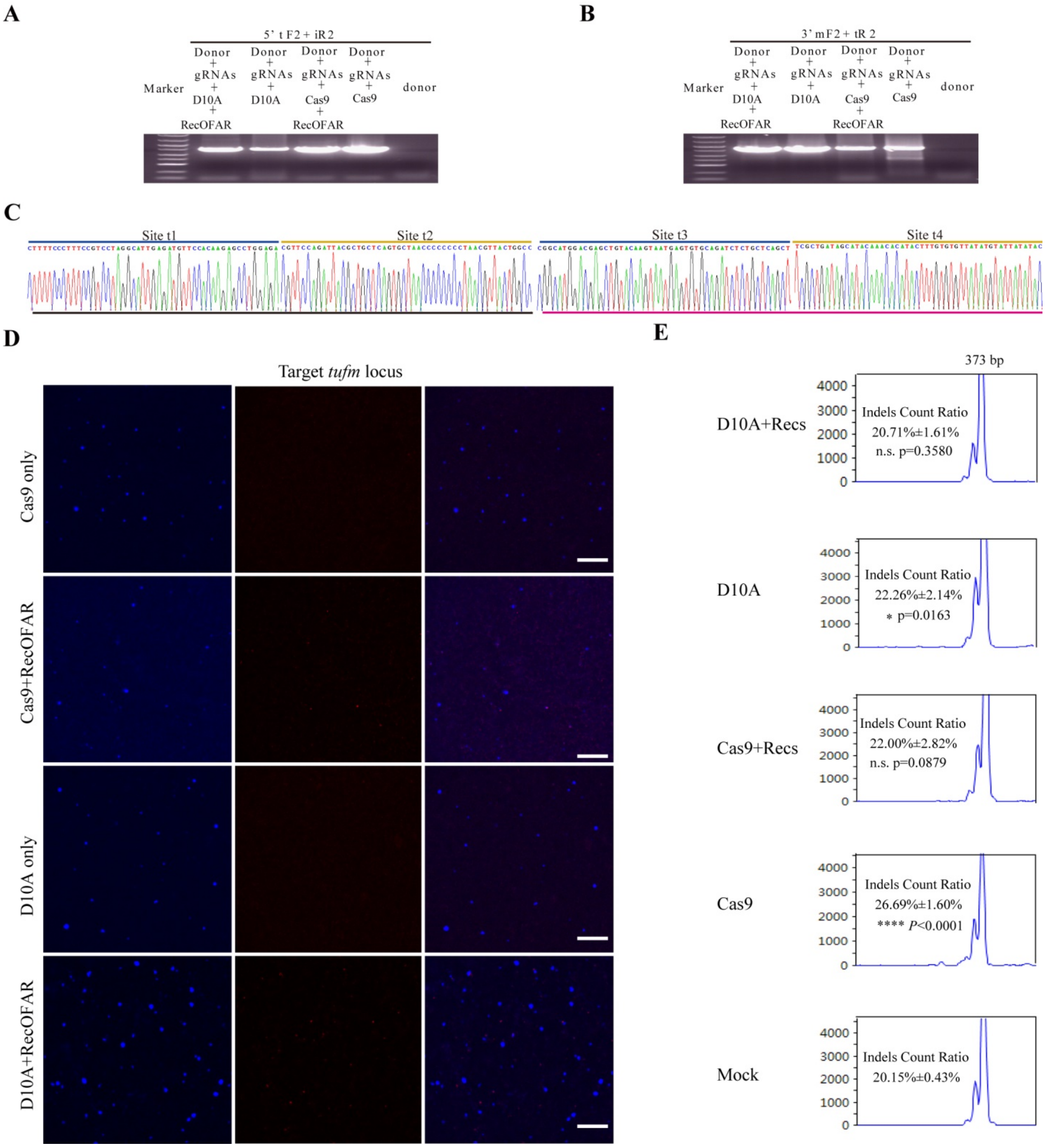

Supplementary Figure S17. Genotype, phenotype and on-target indel analyses of gene KI at the human *TUFm* locus. (A) PCR analysis of the 5′ junctions at the *TUFm* locus. The primers used are shown in Fig 6E. (B) PCR analysis of the 3′ junctions at the *TUFm* locus. The primers used are shown in Fig 6E. (C) Representative sequencing results of primary human PBLCs cells with IRES-mito-mCherry KI at the *TUFm* locus. (D) mCherry fluorescence signal in the TUFm-IRES-mito-mCherry KI-positive cell. Scale bar, 100 μm. Blue, TagBFP transfection positive cells. (E) On-target analysis by IDAA at the *TUFm* locus. IDAA detected obvious on-target indels in the wtCas9 or Cas9n only groups, which were fully inhibited by the supplementation of RecOFAR factors (Cas9+Recs and D10A+Recs). Total indel count ratio was calculated from peak areas of the summed indel peaks relative to the total peak area. Mock, control group with mock treatment. Results were

presented with mean±S.D. Comparisons between groups and mock were evaluated by Student's *t*-test. n.s., no significant difference. *$P < 0.05$, ****$P < 0.0001$.

Supplementary Table S1 Summary of DNA integration studies using TALENs and CRISPR/Cas9 in zebrafish

	Integration mechanism	Donor type	Insertion	Germline transmission rate	Disadvantage(s)	Advantage(s)	Reported in Refs
**Targeting system**							
TALENs	NHEJ/HR	ssDNA	LoxP	~10%	Short fragment insertion/ unwanted indels	LoxP knockin	V.M. Bedell et al.(2012)
TALENs CRISPR/Cas9	NHEJ	plasmid	Gal4	~10%	Disruption of endogenous gene	Large fragment insertion/easy donor design	Thomas O. Auer et al. (2013)
CRISPR/Cas9	NHEJ	plasmid	Gal4/RFP	~12%	Disruption of promoter/plasmid backbone insertion	Large fragment insertion/easy donor design	Kimura, Y et al.(2014)
CRISPR/Cas9	NHEJ	plasmid	EGFP	~12% (after GFP pre-screen)	Plasmid backbone insertion/ unwanted indels	Large fragment insertion	Li, et al.(2015)
TALENs	HR	Lineari zed DNA	EGFP	~1.5%	Disruption of endogenous gene	Large fragment insertion	Zu. et al.(2013)
CRISPR/Cas9	HR	plasmid	Single base	~11%	Short fragment insertion/ unwanted indels	Correction of mismatches/targe t mutation	Irion. et al.(2014)
TALENs	HR	Lineari zed DNA	sfGFP/ tdTomato	~11% (33% incorrect)	Frequent unwanted insertion	Large fragment insertion	Shin. et al.(2014)
CRISPR/Cas9	NHEJ/HR	plasmid	EGFP	~15% (40% incorrect)	Frequent unwanted indels	Large fragment insertion	Yu Hisano. et al. (2015)

Supplementary Table S2 Primers used for donor DNA construction

Primer name	Direction (5'-3')
zGFAP-2A-ChR2-EYFP-pBS-F	TCCCCGCGGTGGAGCTCCAGCTTTTGT
zGFAP-2A-ChR2-EYFP-pBS-R	CGGGGTACCCAATTCGCCCTATAGTGA
zGFAP-5'HA-F	CGGGGTACCGGTGCAATATTTATTTAAAAAAG
zGFAP-5'HA-R	GATCCCGGTAGGTCTTTACGTTCGGTAGTGGACTCTTTAATG
zGFAP-2A-ChR2-EYFP-F	CGTAAAGACCTACCGGGATCCGGAGCTACTAATTTC
zGFAP-2A-ChR2-EYFP-R	CTTCTTCTGCAGCCAAGCCAGTGTCTGAGCCTCATTACTTGTACAGCTCGTC
zGFAP-3'HA-F	GGCTTGGCTGCAGAAGAAGATCTCCTTGTGATTACGTTCAGGAAGCTGTGTTTG
zGFAP-3'HA-R	TCCCCGCGGGTGAAGATATGGTCACAG
zNdr2-pBS-F	GCCTGTGATCCGCGGTGGAGCTCCAGCTTTTG
zNdr2-pBS-R	CTGAAAAAAGGTACCCAATTCGCCCTATA
zNdr2-5'HA-F	CGAATTGGGTACCTTTTTCAGCTCACATAATAAC
zNdr2-5'HA-R	GGGGGTGTTTGGCCCCCGGCGGCCCCTG
zNdr2-dendra2-F	CGGGGGCCAAACACCCCCGGCATCAAC
zNdr2-dendea2-R	CAGGCCTGCGCACTGGTCCGCCGCTACCTCCGCTTCCACCGGAACCTCCGCCGGTGCTATCCAGTCCGAG
zNdr2-3'HA-F	GACCAGTGCGCAGGCCTGAACTCCAGAGGACCCCTCTCCATAAATCCACAACATGCAGGAGGGTGGACATG
zNdr2-3'HA-R	CCACCGCGGATCACAGGCTCTCGTAGTACAGCATAC
zBmp2b-5'HA-F	CGAATTGGGTACCAAGCGAGCTCTTTGGTCTGAG
zBmp2b-5'HA-R	TGCTTGGCGTTTCTCTCGGTTCGAATGCAAGACGGCTG
zBmp2b-dendra2-F	CGAGAGAAACGCCAAGCAGGCGGAGGTTCCGGTGGAAG
zBmp2b-dendra2-R	GCCCTCGTCGTCCGCCGCTACCTCCGCTTCCACCGGAACCTCCGCCGGTGCTATCCAGTCCGAG
zBmp2b-3'HA-F	CGACGAGGGCAAAAGCCGAGGAGAAAGCACCACCAGCGCTCGAACTGTAGGCGACATGCTCTCTATGTCGACTTCAGTGATGTC
zBmp2b-3'HA-R	CTGGAGCTCCACCGCGGATGCATAGATTTTTAATATGCC
zLefty2-pBS-F	CCTACGCGGTACCCAATTCGCCCTATAG
zLefty2-pBS-R	CCGGTCATCCGCGGTGGAGCTCCAGC
zLefty2-5'HA-F	CTCCACAATCATATTAGGGAATTCTGCCACCTCGATTTCAGTATAGTCACCTTTCTTCACCAGGTACATCATGGGC
zLefty2-5'HA-R	GAATTGGGTACCGCGTAGGTAAATCGAG
zLefty2-dendra2-F	CGACACCACACTTAGGTGCTATCCAGTCCGAG
zLefty2-dendea2-R	CCCTAATATGATTGTGGAGAAGTGTGGATGCTCCATGGACAAAATCCCTCCAGTCGGCGGAGGTTCCGGTGGAAG
zLefty2-3'HA-F	CTCCACCGCGGATGACCGGTATTGAAATAAAG
zLefty2-3'HA-R	GCACCTAAGTGTGGTGTCGAATAGTTTG
zGFAP-5.7kb-pBS-F	GATGGTGACTGTGAGGCTCAGACACTGGC
zGFAP-5.7kb-pBS-R	CTCTGTCATAGGACCAGGATTTTCTTC
zGFAP-5.7kb-F	CCTGGTCCTATGACAGAGACCCTGCCTC
zGFAP-5.7kb-R	CTGAGCCTCACAGTCACCATCTTCGAGC
Mouse-Slc6a4-5'HA-F	CGAATTGGGTACCATGCTCTGTTCTTAGATTAG
Mouse-Slc6a4-5'HA-R	GAATTTCCGTTGGTGTTTCAGGAGTGATACTTTTAATAATGCGCTGTTGCGAAGAAAAGTGCAGTGTTAC
Mouse-Slc6a4-2AEYFP-F	CTGAAACACCAACGGAAATTCCGTGTGGGGACATTCGCATGAATGCTGTGGGATCCGGAGCTACTAATTTC
Mouse-Slc6a4-2AEYFP-R	CCTCAGTGTGCTATTACTTGTACAGCTCGTC
Mouse-Slc6a4-3'HA-F	CAAGTAATAGCACACTGAGGGAGAGGAC
Mouse-Slc6a4-3'HA-R	CCACCGCGGAGTCCGATTCTGCGATTG
Mouse-Slc6a4-pBS-F	GAATCGGACTCCGCGGTGGAGCTCCAGC
Mouse-Slc6a4-pBS-R	GAACAGAGCATGGTACCCAATTCGCCCTATAG
Mouse-Myc-5'HA-F	CTCCCCCAACACCAGGACGTTTG
Mouse-Myc-5'HA-R	TCCGCTTCCACCGGAACCTCCGCCTGCACATGAGTTTCGAAGCTGTTCGAG

Mouse-Myc-sfGFP-F	GGTTCCGGTGGAAGCGGAGGTAGCGGCGGAATGCGTAAAGGCGAGGAGCT
Mouse-Myc-sfGFP-R	TCCTCGAGTTAGGTCAGTTCATCATTTGTACAGTTCATCCAT
Mouse-Myc-3'HA-F	ACTGACCTAACTCGAGGAGGAG
Mouse-Myc-3'HA-R	ACCCTCTGAACCTTTGTATGTC
Mouse-Myc-pBS-F	TACAAAGGTTCAGAGGGTCAGCTTTTGTTCCCTTTAGTGAGGGTTA
Mouse-Myc-pBS-R	GTCCTGGTGTTGGGGGAGCCAATTCGCCCTATAGTGAGTCG
Rat-GFAP-5'HA-F	CACTATAGGGCGAATTGGGTCCCCAGAGTTCTATTGCTTCATTC
Rat-GFAP-5'HA-R	ATTAGTAGCTCCGGATCCCATCACATCCTTGTGCTCCTGCTTC
Rat-GFAP-2AEYFP-F	GAGCACAAGGATGTGATGGGATCCGGAGCTACTAATTTCTCCTTGCTT
Rat-GFAP-2AEYFP-R	CTGCCAGCTGGGCACACCTCACATTTACTTGTACAGCTCGTCCATGCCGAGA
Rat-GFAP-3'HA-F	GGTGTGCCCAGCTGGCAGCCCT
Rat-GFAP-3'HA-R	TAAAGGGAACAAAAGCTGAGGGTTTGTTGTGGAGGGTTTAG
Rat-GFAP-pBS-F	CAGCTTTTGTTCCCTTTAGTGAGGGTTA
Rat-GFAP-pBS-R	CCAATTCGCCCTATAGTGAGTCG
Rat-Drd1-5'HA-F	CACTATAGGGCGAATTGGGTCCATTCTGAACCTCTGCGTG
Rat-Drd1-5'HA-R	ATTAGTAGCTCCGGATCCAGTGGAATGCTGTCCACTGTGTGT
Rat-Drd1-2AEYFP-F	GGATCCGGAGCTACTAATTTCTC
Rat-Drd1-2AEYFP-R	TTACTTGTACAGCTCGTCCATGC
Rat-Drd1-3'HA-F	GACGAGCTGTACAAGTAAATATTGGGTCCTCATCTCTGAGGC
Rat-Drd1-3'HA-R	TAAAGGGAACAAAAGCTGGACCAATGATGAGCCCAGCTA
Rat-Drd1-pBS-F	CAGCTTTTGTTCCCTTTAGTGAGGGTTA
Rat-Drd1-pBS-R	CCAATTCGCCCTATAGTGAGTCG
Rat-Drd2-5'HA-F	CACTATAGGGCGAATTGGCATATCTGAGGTGTGGCAGGATG
Rat-Drd2-5'HA-R	ATTAGTAGCTCCGGATCCACAATGGAGTATTTTCATAAAAGCTTTGCGGAAC TCGATGTTGAAGGTG
Rat-Drd2-2AEYFP-F	GGATCCGGAGCTACTAATTTCTC
Rat-Drd2-2AEYFP-R	TTACTTGTACAGCTCGTCCATGC
Rat-Drd2-3'HA-F	GACGAGCTGTACAAGTAATGAGTCTGCCCCTTGCCTGC
Rat-Drd2-3'HA-R	TAAAGGGAACAAAAGCTGCAAACTGCAGCTTCCCCTCCTC
Rat-Drd2-pBS-F	CAGCTTTTGTTCCCTTTAGTGAGGGTTA
Rat-Drd2-pBS-R	CCAATTCGCCCTATAGTGAGTCG
Rat-Bassoon-5'HA-F	CACTATAGGGCGAATTGGGGTGAGCTGTGCCCAGAACT
Rat-Bassoon-5'HA-R	ATTAGTAGCTCCGGATCCGGAAAATTTTTTGCCAAAAGCAGAGACA
Rat-Bassoon-5.5kb-F	GGATCCGGAGCTACTAATTTCTCCT
Rat-Bassoon-5.5kb-R	TGGACACAATCACCAGAATCAGTCACCATCTTCGAGCAGTC
Rat-Bassoon-3'HA-F	TTCTGGTGATTGTGTCCAGAAGG
Rat-Bassoon-3'HA-R	TAAAGGGAACAAAAGCTGGTGCAAGGGACAGCTTAAACAAC
Rat-Bassoon-pBS-F	CAGCTTTTGTTCCCTTTAGTGAGGGTTA
Rat-Bassoon-pBS-R	CCAATTCGCCCTATAGTGAGTCG
Human-Rpl41-5’HA-F	CACTATAGGGCGAATTGGCCAAATACTCGGTCTTCCGAAAG
Human-Rpl41-5’HA-R	TTTGCGCTTCAGCCTGAAAAGCAGACAtAAAAATGGAATACACCTCAAACCA GAGT
Human-Rpl41-IRES-F	TTCAGGCTGAAGCGCAAAAGAAGAAAGATGAGGCAGAGGTCCAAGTAACC CCCCCCTAACGTTACTGGCCG
Human-Rpl41-MitoCox8-R	CAACGAATGGATCTTGGCGCG
Human-Rpl41-5’EGFP-F	GCCAAGATCCATTCGTTGGTGAGCAAGGGCGAGGAGCTG
Human-Rpl41-5’EGFP-R	CAACAAGCTAGCGGTTTACTTGTACAGCTCGTCCATGCC
Human-Rpl41-3’HA-F	TAAACCGCTAGCTTGTTGCGTcGAGGCCACAGGAGCAGAAAC
Human-Rpl41-3’HA-R	TAAAGGGAACAAAAGCTGGCGGCCTCCACCTAAGTCTC
Human-TUFm-F	GCACTGAGCAGCGTAATCTGGAAC
Human-TUFm-R	CTGAGCCTGCGCCAGAGCATC
Human-TUFm-IRES-F	GATTACGCTGCTCAGTGCTAACCCCCCCCTAACGTTAC
Human-TUFm-IRES-R	GCTCTGGCGCAGGCTCAGCATGGTTGTGGCCATATTATCATCG
Monkey-CAMK2A-5'HA-F	TGTGCAGGATACCCGCTTTAGGTGGG
Monkey-CAMK2A-5'HA-R	ATTAGTAGCTCCGGATCCGCTGTAAGTCTCACTCGAGCACGTACTCATGGCA TGGTGTTTCCTGCTTG
Monkey-CAMK2A-2AEYFP-F	GGATCCGGAGCTACTAATTTCTCCTTGCTT

Monkey-CAMK2A-2AEYFP-R	CCCTGGCCTGGTCCCTCATTACTTGTACAGCTCGTCCATGCCGAGA
Monkey-CAMK2A-3'HA-F	TGAGGGACCAGGCCAGGGTCACT
Monkey-CAMK2A-3'HA-R	GCCCCAATAACCACAGGTGACAAGGT
Monkey-CAMK2A-pBS-F	ACCTGTGGTTATTGGGGCCAGCTTTTGTTCCCTTTAGTGAGGGTTA
Monkey-CAMK2A-pBS-R	AAGCGGGTATCCTGCACACCAATTCGCCCTATAGTGAGTCG
Monkey-Oct4-5'HA-F	GATGGGGTGGCTGGATTTGGCCA
Monkey-Oct4-5'HA-R	CTCGCCCTTGCTCACCATGGGGAAGGAAGGCACCCCAAG
Monkey-Oct4-EGFP-F	ATGGTGAGCAAGGGCGAGGAGCTG
Monkey-Oct4-EGFP-R	AAGCAAGGAGAAATTAGTAGCTCCGGATCCCTTGTACAGCTCGTCCATGCCGAG
Monkey-Oct4-NeroR-F	TACTAATTTCTCCTTGCTTAAGCAAGCTGGTGATGTTGAAGAAAATCCTGGTCCTATGATTGAACAAGATGGATTGCACGCA
Monkey-Oct4-NeroR-R	GGGTCCGGGGTTCTCCTCCACGTCTCCTGCTTGCTTTAACAGAGAGAAGTTCGTGGCTCCGGAGCCGAAGAACTCGTCAAGAAGGCGAT
Monkey-Oct4-3'HA-F	GGAGGAGAACCCCGGACCCATGGCAGGTCATTTAGCAAGTGAATTTGCATTTAGTCCTCCTCCAGGAGGTGGAGGTGATG
Monkey-Oct4-3'HA-R	GCCTTGAAGGACTGGAAGATACATC
Monkey-Oct4-pBS-F	CTTCCAGTCCTTCAAGGCCAGCTTTTGTTCCCTTTAGTGAGGGTTA
Monkey-Oct4-pBS-R	AAATCCAGCCACCCCATCCCAATTCGCCCTATAGTGAGTCG

Supplementary Table S3 Oligonucleotides used for making templates for *in vitro* transcription of sgRNAs

ssDNA name	Direction (5'-3')
zGFAP-sg1-F	GAAATTAATACGACTCACTATAGGcagatccttcctctccgtagGTTTTAGAGCTAGAAATAGC
zGFAP-sg2-F	GAAATTAATACGACTCACTATAGGatctccttcactaatgcttcGTTTTAGAGCTAGAAATAGC
zGFAP-sg3-F	GAAATTAATACGACTCACTATAGGaggaaggatctgccataatgGTTTTAGAGCTAGAAATAGC
zNdr2-sg1-F	GAAATTAATACGACTCACTATAGGggctcctgacaggtggccccGTTTTAGAGCTAGAAATAGC
zNdr2-sg2-F	GAAATTAATACGACTCACTATAGGtgcacaagagcaccacctgcGTTTTAGAGCTAGAAATAGC
zLefty2-sg1-F	GAAATTAATACGACTCACTATAGGatccctccagtctgacagtgGTTTTAGAGCTAGAAATAGC
zLefty2-sg2-F	GAAATTAATACGACTCACTATAGGatccctccagtctgacagtgGTTTTAGAGCTAGAAATAGC
zBmp2b-sg1-F	GAAATTAATACGACTCACTATAGGgtcgagcctgccgcttttctGTTTTAGAGCTAGAAATAGC
zBmp2b-sg2-F	GAAATTAATACGACTCACTATAGGtaggcgacatgctctctatgGTTTTAGAGCTAGAAATAGC
Mouse-Slc6a4-sg1-F	GAAATTAATACGACTCACTATAGGacttttaataatgcgctgttTTTTAGAGCTAGAAATAGC
Mouse-Slc6a4-sg2-F	GAAATTAATACGACTCACTATAGGgaatgctgtgtaacacactgGTTTTAGAGCTAGAAATAGC
Mouse-Slc6a4-sg3-F	GAAATTAATACGACTCACTATAGGtgtgttacacagcattcatgGTTTTAGAGCTAGAAATAGC
Mouse-Myc-sg1-F	GAAATTAATACGACTCACTATAGGgctcgtctgcttgaatggacGTTTTAGAGCTAGAAATAGC
Mouse-Myc-sg2-F	GAAATTAATACGACTCACTATAGGctcgaacagcttcgaaactcGTTTTAGAGCTAGAAATAGC
Rat-GFAP-sg1-F	GAAATTAATACGACTCACTATAGGgagcacaaggatgtgatgtgGTTTTAGAGCTAGAAATAGC
Rat-GFAP-sg2-F	GAAATTAATACGACTCACTATAGGaagatgcacagcacagagttGTTTTAGAGCTAGAAATAGC
Rat-Drd1-sg1-F	GAAATTAATACGACTCACTATAGGatgaggacccaatattcaagGTTTTAGAGCTAGAAATAGC
Rat-Drd1-sg2-F	GAAATTAATACGACTCACTATAGGctgaggccacgagttcccttGTTTTAGAGCTAGAAATAGC
Rat-Drd2-sg1-F	GAAATTAATACGACTCACTATAGGtcccacctccctgcctatgcGTTTTAGAGCTAGAAATAGC
Rat-Drd2-sg2-F	GAAATTAATACGACTCACTATAGGgcagtgcaagatcttcatgaGTTTTAGAGCTAGAAATAGC
Rat-Bassoon-sg1-F	GAAATTAATACGACTCACTATAGGgcaaaaaattttcctcattcGTTTTAGAGCTAGAAATAGC
Rat-Bassoon-sg2-F	GAAATTAATACGACTCACTATAGGcagggccataggtcacggtaGTTTTAGAGCTAGAAATAGC
hRpl41-sg1	GAAATTAATACGACTCACTATAGGtcagcctgaaaagcagacacGTTTTAGAGCTAGAAATAGC
hRpl41-sg2	GAAATTAATACGACTCACTATAGGaccgctagcttgttgcaccgGTTTTAGAGCTAGAAATAGC
hTUFm-sg1	GAAATTAATACGACTCACTATAGGtagtccggttgccatctcgcGTTTTAGAGCTAGAAATAGC
hTUFm-sg2	GAAATTAATACGACTCACTATAGGgtttaaggcctgccctagccGTTTTAGAGCTAGAAATAGC
Monkey-CAMK2A-sg1-F	GAAATTAATACGACTCACTATAGGacacacacggggtgcttctcGTTTTAGAGCTAGAAATAGC
Monkey-CAMK2A-sg2-F	GAAATTAATACGACTCACTATAGGgatccactctgtccgtgcaaGTTTTAGAGCTAGAAATAGC

Monkey-Oct4-sg1-F	GAAATTAATACGACTCACTATAGGgccgggggcctggtgaaatgGTTTTAGAGCTAGAAATAGC
Monkey-Oct4-sg2-F	GAAATTAATACGACTCACTATAGGttctcgccccctccaggaggGTTTTAGAGCTAGAAATAGC
sg scaffold-R	TTGTGAAAAGCACCGACTCGGTGCCACTTTTTCAAGTTGATAACGGACTAGCCTTATTTTAACTTGCTATTTCTAGCTCTAAAAC

Supplementary Table S4 Primers used for nest PCR

Primer name	Direction (5'-3')
Zebrafish-GFAP-OF1	ACTAGGAGGCGCTGTTCATCAACC
Zebrafish-GFAP-OR1	AATCCATCCGGCACAGTAACATTG
Zebrafish-GFAP-IF1	TGACAATAAACATTACCAAACCGTGC
Zebrafish-GFAP-IR1	TTAGTAACGAACAAAAGTTCGCGTCC
Zebrafish-GFAP-OF2	TACCAACTTGTTGCTTGAAGGCAC
Zebrafish-GFAP-OR2	ATCACATGGTCCTGCTGGAGTTCG
Zebrafish-GFAP-IF2	TTTTCAGGTTCATAAGTGTGACGAGG
Zebrafish-GFAP-IR2	TCGGCATGGACGAGCTGTACAAGT
Zebrafish-Ndr2-OF1	TTGTTTGGTCACATAAAATCGTCTGC
Zebrafish-Ndr2-OR1	AAATCGCACAGGTAGTGTCCTCCG
Zebrafish-Ndr2-IF1	ACATTTGCTGTGCTTGTTTGCGTG
Zebrafish-Ndr2-IR1	TGAAGCGCACGTTCTGGAAGAAG
Zebrafish-Ndr2-OF2	CTCAAGTGTTCCTGCGGTGCATC
Zebrafish-Ndr2-OR2	GGAGGACACTACCTGTGCGATTTC
Zebrafish-Ndr2-IF2	TGTATCACAGGCATCCGCACTCC
Zebrafish-Ndr2-IR2	CGATTTCAAGACCACCTACAAGGC
Zebrafish-Lefty2-OF1	GAAGTGGGTCCAAATAGAGCCCAT
Zebrafish-Lefty2-OR1	TGAAGCGCACGTTCTGGAAGAAG
Zebrafish-Lefty2-IF1	TGCCTTCTGCTGGTGTGCTAAAAT
Zebrafish-Lefty2-IR1	TTGACGCGCATATCTTCCTTGATC
Zebrafish-Lefty2-OF2	TGCATTCATCTGACCACTAGAGGACTC
Zebrafish-Lefty2-OR2	CGATTTCAAGACCACCTACAAGGC
Zebrafish-Lefty2-IF2	GAGATAAATAGGCCACCACAGACCTG
Zebrafish-Lefty2-IR2	AACAAGGTGAAGCTGTACGAGCACG
Zebrafish-Bmp2b-OF1	AGCTGAGGTGCGCAGTCAAGTAGC
Zebrafish-Bmp2b-OR1	AAATCGCACAGGTAGTGTCCTCCG
Zebrafish-Bmp2b-IF1	CCTCAAGACGGATTGACTGGGAG
Zebrafish-Bmp2b-IR1	TTATCCTCGAAGGTCATGGTGCG
Zebrafish-Bmp2b-OF2	ATACCCAATTCCTAACCCCACCAC
Zebrafish-Bmp2b-OR2	CGATTTCAAGACCACCTACAAGGC
Zebrafish-Bmp2b-IF2	TTGTGCCTTAAAAGGAAGTGTCGC
Zebrafish-Bmp2b-IR2	TACAAGGCCAAGAAGGTGGTCCAG
Mouse-Slc6a4-OF1	CGCTATAAGCTGGGGACTGC
Mouse-Slc6a4-OR1	CAGACAAAGCGCCGCCATAG
Mouse-Slc6a4-IF1	GGGGACTGCTTGTGTAAGTC
Mouse-Slc6a4-IR1	GGATTTTCTTCAACATCACCAGC
Mouse-Slc6a4-OF2	ATCACATGGTCCTGCTGGAGTTCG
Mouse-Slc6a4-OR2	GGACTCGAAGTTATAGAGTGCCAG
Mouse-Slc6a4-IF2	TCGGCATGGACGAGCTGTACAAGT
Mouse-Slc6a4-IR2	AGGTAAGATTTGCCTCCATGCTTTC
Mouse-Myc-OF1	GGGGGTGAATTACCTGCTTCTT
Mouse-Myc-OR1	GTTCACGTCGCCATCCAGC
Mouse-Myc-IF1	AGCTGGGACAGCCTTTATAGC
Mouse-Myc-IR1	CTTTACGCATTCCGCCGCTAC
Mouse-Myc-OF2	CTGGCCGATCACTACCAGCA
Mouse-Myc-OR2	CCTCTTTCGTGGCACTCTGTC
Mouse-Myc-IF2	CAGAGCGTGCTGTCCAAGG
Mouse-Myc-IR2	AGACCCCTAACCAGCGGAAG
Rat-GFAP-OF1	TAGCCTATGCTCAAGGTGAGGTC
Rat-GFAP-OR1	TTAGTAACGAACAAAAGTTCGCGTCC
Rat-GFAP-IF1	CACCACTTTTGTGTCGCTAGGTGG
Rat-GFAP-IR1	ACAAAGCGCCGCCATAGTCCAT
Rat-GFAP-OF2	GGCCGACAAGCAGAAGAACG
Rat-GFAP-OR2	GAAATCGGAGGTCAAGCACAGG

Rat-GFAP-IF2	AAAGACCCCAACGAGAAGCG
Rat-GFAP-IR2	CGTTGGAGGCGGATTTAACC
Rat-Drd1-OF1	ACTCTCCTGGGCAATACCCTTGTC
Rat-Drd1-OR1	TGTGGCAAGGTAGAGCATAGAGG
Rat-Drd1-OF2	TCGGCATGGACGAGCTGTACAAGT
Rat-Drd1-OR2	CGAGCCGACTTGGTTAGACCTG
Rat-Drd2-OF1	TTATTCAGGCAGTTGGGAAAGGAGAG
Rat-Drd2-OR1	TGTGGCAAGGTAGAGCATAGAGGGAT
Rat-Drd2-OF2	AAGGACGACGGCAACTACAAGACC
Rat-Drd2-OR2	TGGAGCTGTGAATGAACCTGGGAG
Rat-Bassoon-OF1	GCCTCTGACAGCAAAAAGGGC
Rat-Bassoon-OR1	TTCAGCACGAACTCGAACAACTCC
Rat-Bassoon-IF1	GCAGGACTGGTGAGTTAGCC
Rat-Bassoon-IR1	CAGGGTCTCTGTCATAGGACCAG
Rat-Bassoon-OF2	GCTGGAGGTTGGACAAATGTGAAC
Rat-Bassoon-OR2	CAGTCAAAGCCCCTCTCCAAG
Rat-Bassoon-IF2	TATCCGCAATCTTGACTCTGAAACC
Rat-Bassoon-IR2	TACAGACCAATTCCCCGAGGTC
rF1	CAACTCCCAAGAAGCAATGCGA
iR1	GGTTGTGGCCATATTATCATCG
rF2	CAGCGTCCTCATACTAAATCTG
iR2	GGAATGCTCGTCAAGAAGACAG
rR1	TGAAAGAGGATGTGAGGGTCAGAGC
eF1	AAGAACGGCATCAAGGTGAACTTC
rR2	TCTTCTAGCTTGGCAGGTTCCGTG
eF2	ACATGGTCCTGCTGGAGTTCGTG
tF1	GACATAGCAAGAACATCCGCACTG
iR1	GGTTGTGGCCATATTATCATCG
tF2	TGCCTTCCCTCTGTCTCACATTCG
iR2	GGAATGCTCGTCAAGAAGACAG
tR1	ACAAGTTACACCGAGTCTGCCTGTC
mF1	GCTGAAGGTGACCAAGGGTGG
tR2	AGAAACGTGAAGACAATGAGGGCC
mF2	GGTCAAGACCACCTACAAGGC
Monkey-CAMK2A-OF1	TTCACTTGGCTTGCACGATGTTT
Monkey-CAMK2A-OR2	GCCCTGACTTGCTGTTCCTGAG
Monkey-CAMK2A-IF1	TCACTCACATAGCAACCACCAGG
Monkey-CAMK2A-IR1	CGCCATAGTCCATAGGACCAGGA
Monkey-CAMK2A-IF2	ACATGGTCCTGCTGGAGTTCGT
Monkey-CAMK2A-IR2	CGCCTGTGTCAGTACACCTTGG
Monkey-Oct4-OF1	TCAGTCCAAAGCCTGGTCCCTTG
Monkey-Oct4-OR1	TCAAGGACGACGGCAACTACAAG

Monkey-Oct4-IF1	GTCTGGCAGTCTACTCTTGAAGATG
Monkey-Oct4-IR1	ACAAGTTCAGCGTGTCCGGCGA
Monkey-Oct4-OF2	CGATGCCTGCTTGCCGAATATCAT
Monkey-Oct4-OR2	GCACACAGCCAGGCACTTAGGAA
Monkey-Oct4-IF2	CATCGCCTTCTATCGCCTTCTTGAC
Monkey-Oct4-IR2	CCATTATCATTCAAGGCTCAGCAGTG

Supplementary Table S5 Primers used for off-target analysis

Primer name	Direction (5'-3')
Ndr2-sg1-OT1-f	GCGTCTGATCCGGTGTCCAGAG
Ndr2-sg1-OT1-r	TACAGGCAACGACAACAGCCTCTG
Ndr2-sg1-OT2-f	AGCCGGAAAGCCTCCACTTCCT
Ndr2-sg1-OT2-r	AACCAGCAGCAGTGCAGAACACAG
Ndr2-sg2-OT1-f	ACCATGCGCTATGGTGCAAATC
Ndr2-sg2-OT1-r	AGCAGGTGGCAATGTGAAGTCTG
Ndr2-sg2-OT2-f	TCAGATGTGGGTGTATGCTGCAAC
Ndr2-sg2-OT2-r	AATTGGAGAAACGCTCGTTCCTG
Ndr2-sg2-OT3-f	AGTGTTCTTGACAGGCAGTGACCG
Ndr2-sg2-OT3-r	CAAATCCGTGTAAACCAACATGACC
Ndr2-sg2-OT4-f	TTCTCTGATAAACAGTCCACCGTGG
Ndr2-sg2-OT4-r	AATGTGGTGTGATCGGGCTTGAC
Ndr2-sg2-OT5-f	CATGAATGATTGAAGCATGTATTGCAC
Ndr2-sg2-OT5-r	CACTGAATTACAGTAAAGGCCCAGATG
Ndr2-sg2-OT6-f	TCTAAAGGGTTGACCCCTTGGCTC
Ndr2-sg2-OT6-r	AGCAAGCTGTTCCTGTGAAGAGGG
Ndr2-sg2-OT7-f	ACGCATCCCTATAGCGACAGATGTG
Ndr2-sg2-OT7-r	ATAAACACTCTATCACATGCGCACG
Ndr2-sg2-OT8-f	ACACATTTCTTGGATGATGAAAGGG
Ndr2-sg2-OT8-r	GCCAAGTGTTTGCGTTCTTAAAGAG
Ndr2-sg2-OT9-f	AACCAACTTGCGTTATTCAGCAAAC
Ndr2-sg2-OT9-r	ACAGTGGATGGTGCAGCGCACTAG
Ndr2-sg2-OT10-f	ATGCTTCCTTTCCGCATCCAAAG
Ndr2-sg2-OT10-r	AACTGAAACATGGTCACCCTGTCC
Ndr2-sg2-OT11-f	CATGCTGCCTGTTACTTTTAGGACC
Ndr2-sg2-OT11-r	ACTATTTTGGGTATCGCCTTTGTGG
Ndr2-sg2-OT12-f	AAATGTTGCTCCTCGCTTCCTGG
Ndr2-sg2-OT12-r	ACCTGTGGATACTCATCCCACGG
Ndr2-sg2-OT13-f	ACCTAGCATATGACCCCACTCCAG
Ndr2-sg2-OT13-r	ACTTCCTTGGTGTAAGACAGGCATG
Ndr2-sg2-OT14-f	CACGTCTTTGATTCTCTAACACACACG
Ndr2-sg2-OT14-r	TGTACTTTCAACATACTACTAATACAG
Ndr2-sg2-OT15-f	AGCGCAGGTTGTGTCCTTAATGAG
Ndr2-sg2-OT15-r	CTCAATGGGGTCAGGAACTGTTGC
Ndr2-sg2-OT16-f	TGAGCTGGTGACAGGAATTTACTGG
Ndr2-sg2-OT16-r	AGTGTCCTGAGTGGGGTCTGGATG
Ndr2-sg2-OT17-f	ACTTCTTTGCTGAATCCGAGTTGC
Ndr2-sg2-OT17-r	TGGATGTGATGAACTTCTCCAGGC
Ndr2-sg1-OfT1'-f	AGCTGACCGGCAGCAAAATTGacacagagccatgctgctgc
Ndr2-sg1-OfT1'-r	TTCTCCCAGTGGGTTCGGGCAG
Ndr2-sg2-OfT1'-f	AGCTGACCGGCAGCAAAATTGcccgcagtgttcttgacagg
Ndr2-sg2-OfT1'-r	TCGAGATGTGCTCGTTTGTGG
Ndr2-sg2-OfT2'-f	AGCTGACCGGCAGCAAAATTGcttccctctaaagtcacagg
Ndr2-sg2-OfT2'-r	TCAGAGTTCACGCTGGGCAG
Ndr2-sg2-OfT3'-f	AGCTGACCGGCAGCAAAATTGccagcatgactttgcttgag
Ndr2-sg2-OfT3'-r	CATGCCGAACAGTACAACAC
Ndr2-sg2-OfT4'-f	AGCTGACCGGCAGCAAAATTGacagagccatgctgctgctg
Ndr2-sg2-OfT4'-r	AGCTCTTCTCCCAGTGGGTTC
Ndr2-sg2-OfT5'-f	AGCTGACCGGCAGCAAAATTGgctgtcaatacagatgacacc
Ndr2-sg2-OfT5'-r	CCTTGGTGTAAGACAGGCATG
Mouse-Slc6a4-sg123-OnT-f	CGACGCTTGTACTTCTCCACTCTG
Mouse-Slc6a4-sg123-OnT-r	GTGTCACCAGCTAATGTGGCAGTA
Mouse-Slc6a4-sg1-OfT1-f	TGGTCATCCCATTGTCTGTTGAAGA
Mouse-Slc6a4-sg1-OfT1-r	ACTGCTAAGAAACCTGAACCAGAGT
Mouse-Slc6a4-sg1-OfT2-f	AGAAGGCTGTAATCTGACCTGAGAC
Mouse-Slc6a4-sg1-OfT2-r	CACAAACGCTCATACAATTCCTCCC
Mouse-Slc6a4-sg1-OfT3-f	TGTGGAATAGTGGTCAGGCTTATCA

Mouse-Slc6a4-sg1-OfT3-r	AGATCACTGGCTGTACACTGTATGC
Mouse-Slc6a4-sg1-OfT4-f	CTCCTCCTTCCCACACACCTATCA
Mouse-Slc6a4-sg1-OfT4-r	GTGACAAGCCTGACAACCTGAGT
Mouse-Slc6a4-sg1-OfT5-f	CTGCTTCTTCCTAAGGAGGCTGATG
Mouse-Slc6a4-sg1-OfT5-r	ACTCACTTCCACACATGAACTTCTG
Mouse-Slc6a4-sg1-OfT6-f	TCTGTGCTAACCATCCAATTCCTCA
Mouse-Slc6a4-sg1-OfT6-r	GCTTAAGGGTTGGCTCAGTGACA
Mouse-Slc6a4-sg1-OfT7-f	CAGGAAACCAGGCAGTCTCTGATAT
Mouse-Slc6a4-sg1-OfT7-r	GATAGGTAACCCACAGCAACAAGGA
Mouse-Slc6a4-sg1-OfT8-f	GAGAGGTAACGCCCACCCTTTATG
Mouse-Slc6a4-sg1-OfT8-r	ATGGCAGTGGCTGAACCTTTACG
Mouse-Slc6a4-sg1-OfT9-f	AGTGGTGGATATGGAGCAGGAAGT
Mouse-Slc6a4-sg1-OfT9-r	AACGCACCCTGGCAAAGAAGTAA
Mouse-Slc6a4-sg1-OfT10-f	GGAGCATCTCAAGGACATGACAGAA
Mouse-Slc6a4-sg1-OfT10-r	TTGGTTGGTGGTTCAGTCTCTTCTC
Mouse-Slc6a4-sg1-OfT11-f	GACTTGACAGAGACCTGGGCTAAG
Mouse-Slc6a4-sg1-OfT11-r	GGCTGCAATCCTTCCTCCTATTCTT
Mouse-Slc6a4-sg2-OfT1-f	TCCAGAGGGTAAGTTATCTGCAAGA
Mouse-Slc6a4-sg2-OfT1-r	CTAGCCTGGCGGATTAAAGAAAGC
Mouse-Slc6a4-sg2-OfT2-f	GGAAGAATGAAGAAGTCTGGGAGGT
Mouse-Slc6a4-sg2-OfT2-r	GAATGCCACTGAACCACATTGCTT
Mouse-Slc6a4-sg2-OfT3-f	TCAAGATCACAGTGAGGTGGAGGT
Mouse-Slc6a4-sg2-OfT3-r	TGCTGGAACAATGAATGGGTCTGA
Mouse-Slc6a4-sg2-OfT4-f	CCTAAACAGGCATCATGGTCTCACA
Mouse-Slc6a4-sg2-OfT4-r	CACAGGCTCAGGATCAAAGGAATCA
Mouse-Slc6a4-sg2-OfT5-f	AACTGGTTCCAAGTGAGCTTCCTT
Mouse-Slc6a4-sg2-OfT5-r	ATGCTAGAGGTAGAGCTGCCACT
Mouse-Slc6a4-sg2-OfT6-f	CCAACTGTCATTGCCACCAGACT
Mouse-Slc6a4-sg2-OfT6-r	GAGAGAGGAAGTTATGCCTGCCATT
Mouse-Slc6a4-sg2-OfT7-f	ACACAGGCAAAGCTCTCCTTTAGTT
Mouse-Slc6a4-sg2-OfT7-r	TGGAAGCAAGCAGGTAAGCACAT
Mouse-Slc6a4-sg2-OfT8-f	AATGATGCAGGGATCTCTGTGTTCA
Mouse-Slc6a4-sg2-OfT8-r	TCATAGGGCCTTTGTGTCGATTGG
Mouse-Slc6a4-sg2-OfT9-f	CTGGGCACATCTGTCTTTCTTGGT
Mouse-Slc6a4-sg2-OfT9-r	TCCTTACTGTTCTCATTGCTGTGGC
Mouse-Slc6a4-sg2-OfT10-f	TGCTAGGCATGTGACAGTGCTAAC
Mouse-Slc6a4-sg2-OfT10-r	ACATAGGTCATAGGCTGGAGAGAGT
Mouse-Slc6a4-sg2-OfT11-f	TCTTGGGCTCCTCTCTTGTCACT
Mouse-Slc6a4-sg2-OfT11-r	AGGGAATGGATGGTAGCTCTCAGAT
Mouse-Slc6a4-sg2-OfT12-f	GCCATAGTGGAGGAGGCAGATGT
Mouse-Slc6a4-sg2-OfT12-r	CTGACACCTTCACACAGACATCCAT
Mouse-Slc6a4-sg2-OfT13-f	GTTACCACCCATGCCCACATTCA
Mouse-Slc6a4-sg2-OfT13-r	ATCTTCAAACTCAGGCTGCCAGG
Mouse-Slc6a4-sg2-OfT14-f	TCTGTGTCTCCTGCTCTGGAAGG
Mouse-Slc6a4-sg2-OfT14-r	AGGGTAGCTGCAACAAAGAAATGGA
Mouse-Slc6a4-sg2-OfT15-f	AGGTAGGCTGTCTGTAGGTTGGAA
Mouse-Slc6a4-sg2-OfT15-r	GCCTGAAGAAGTCCTTAGTCCTGTG
Mouse-Slc6a4-sg2-OfT16-f	GCCAGTGTCTGTTCAATGATCTCCT
Mouse-Slc6a4-sg2-OfT16-r	GCCAGAGGGAATACCAGCTCAGATA
Mouse-Slc6a4-sg2-OfT17-f	GCTCACTCCTGGCACAGATGAAG
Mouse-Slc6a4-sg2-OfT17-r	CCACTCTTACATCCTTGCTCCAACA
Mouse-Slc6a4-sg2-OfT18-f	GGCAGGTCCTACAGATCCAAAGC
Mouse-Slc6a4-sg2-OfT18-r	TGAGGATGAAGCAACTTAACACAGC
Mouse-Slc6a4-sg2-OfT19-f	GGTGAGCCTATGGCAGAAGGATATG
Mouse-Slc6a4-sg2-OfT19-r	AAGGCATCACTCACAGCTTCCAAT
Mouse-Slc6a4-sg2-OfT20-f	TCAGTGTGACATTGATGGCAGACA
Mouse-Slc6a4-sg2-OfT20-r	TGTTCATTCCCACCTCTACCCTACT
Mouse-Slc6a4-sg2-OfT21-f	GAGGGTGGAAGGAACTGGGAAGA
Mouse-Slc6a4-sg2-OfT21-r	AAAGAGAGGCAAAGGTGCTGTGT
Mouse-Slc6a4-sg2-OfT22-f	ACAGGTGGATTTCTGAGTTTGAGGT
Mouse-Slc6a4-sg2-OfT22-r	CGCAGCATAGGAAGATAGCACAATT

Mouse-Slc6a4-sg2-OfT23-f	TCAGGACAACGATGGGAGTGATCT
Mouse-Slc6a4-sg2-OfT24-r	ACCGTGATGGATGACTGTGAGGAT
Mouse-Slc6a4-sg2-OfT24-f	CACTGGAGAGCCTGGTAGACTAACT
Mouse-Slc6a4-sg2-OfT24-r	TTGAATATGTCACTGTTCGGAGCCA
Mouse-Slc6a4-sg2-OfT25-f	ACTGAACTCCATTGCTTTGCCATCT
Mouse-Slc6a4-sg2-OfT25-r	GCTCCCAACATCTCATGTGTGAACT
Mouse-Slc6a4-sg2-OfT26-f	TTGGAAGTTTCTACTCCCACTGTGA
Mouse-Slc6a4-sg2-OfT26-r	GAGGTATGGCTTCATTGGAGGTGAG
Mouse-Slc6a4-sg3-OfT1-f	AACAATCACCAGAACTGAGCATCAA
Mouse-Slc6a4-sg3-OfT1-r	CCCATACTGACCTTGAACTCCTGAT
Mouse-Slc6a4-sg3-OfT2-f	GCCACAGGATAGGACAGCAGAAG
Mouse-Slc6a4-sg3-OfT2-r	AGCCATCTCTCCAGCCTCTCATT
Mouse-Slc6a4-sg3-OfT3-f	CAAGCACTTAGGCACGACAGGAA
Mouse-Slc6a4-sg3-OfT3-r	CCTCATTGACAGGTGACCAAGAACA
Mouse-Slc6a4-sg3-OfT4-f	AGGGAATCTGAGGACAGCCTAGTG
Mouse-Slc6a4-sg3-OfT4-r	TGCCTTTAACACACTGAGCCATCTT
Mouse-Slc6a4-sg3-OfT5-f	TAGGCATCAGGTCAGAGAACTTGGA
Mouse-Slc6a4-sg3-OfT5-r	GGGTCATCTGGAACGGTAGCATTT
Mouse-Slc6a4-sg3-OfT6-f	TCCATCCATCCATCCATCCATCCA
Mouse-Slc6a4-sg3-OfT6-r	CCCGTAATTTGTGGTAGGCACTTTG
Mouse-Slc6a4-sg3-OfT7-f	TGTGTATGTGTGTCTGTGTCTGTGT
Mouse-Slc6a4-sg3-OfT7-r	TTGAGACCTCGCATAACCACTTCC
Mouse-Slc6a4-sg3-OfT8-f	TCAAGAAACATTCCTCAGGCAACAA
Mouse-Slc6a4-sg3-OfT8-r	GGTCAGCCTATGTGTTTGGAGTGT
Mouse-Slc6a4-sg3-OfT9-f	ATCGAATCTAGGGCCTTGCACATG
Mouse-Slc6a4-sg3-OfT9-r	AAGCAGCCTTTATGGAGCATTGAGT
Mouse-Slc6a4-sg3-OfT10-f	AGTCTGTGAGAGGCATAGCTTGCTA
Mouse-Slc6a4-sg3-OfT10-r	TTCAACACCAACAACAGCCAACTTC
Mouse-Slc6a4-sg3-OfT11-f	GTACAACCTGCTCGTGTGTTCAAAG
Mouse-Slc6a4-sg3-OfT11-r	CTGTGTTTCCAAGGCTCCTTCCA
Mouse-Slc6a4-sg3-OfT12-f	CACCTCAATCCCTCTGTTCTTCTCC
Mouse-Slc6a4-sg3-OfT12-r	GTGGTATGCACATGTTTGCTGGTT
Mouse-Slc6a4-sg3-OfT13-f	CTCCTCTGGCAACAAGCACTGAT
Mouse-Slc6a4-sg3-OfT13-r	GCAGGAACCGTGGAATTAAACAAGA
Mouse-Slc6a4-sg3-OfT14-f	GGCCTAGTGGTTATACATGCAAGTG
Mouse-Slc6a4-sg3-OfT14-r	CGGCACCTGACTGGGAATTTCTT
Mouse-Slc6a4-sg3-OfT15-f	TCTTGCTGCATGAGTCACTGCTATT
Mouse-Slc6a4-sg3-OfT15-r	CCCAAATCTCTGCATTTGTCCAGAC
Mouse-Slc6a4-sg3-OfT16-f	AGGCCAGGAAGTTCTCTGCTGTATA
Mouse-Slc6a4-sg3-OfT16-r	GGCTAACATAGTGGCTCAGTGGTT
Mouse-Slc6a4-sg3-OfT17-f	CCAGCTTAAGTTTGGCCTCTGATGT
Mouse-Slc6a4-sg3-OfT17-r	GCTTAGGGAGATGGCTTAGAGGGTA
Mouse-Slc6a4-sg3-OfT18-f	CCCACCAAGGTAGGCTACAATACAA
Mouse-Slc6a4-sg3-OfT18-r	CTTAGTCTGGGCATCTTCTGTGTCA
Mouse-Slc6a4-sg3-OfT19-f	TTCTATCCCTAGCTCTGGCCTTTGA
Mouse-Slc6a4-sg3-OfT19-r	TGGTCCATTCTAGCAGTTCTGTTCC
Mouse-Slc6a4-sg3-OfT20-f	AAACATGGCCGAAATTTGAGAACGT
Mouse-Slc6a4-sg3-OfT20-r	ACACACCTGTAATCCCAGCACTG
Mouse-Slc6a4-sg3-OfT21-f	TCACTGCTGGGAACTCTCAACCT
Mouse-Slc6a4-sg3-OfT21-r	TCCTGGTGATGCTGGCATAGACA
Mouse-Slc6a4-sg3-OfT22-f	GTTCTCTTGTCACAGGTGGACTCAG
Mouse-Slc6a4-sg3-OfT22-r	GAGCTGTCATGTGGGTGATGGTTAT
Mouse-Slc6a4-sg3-OfT23-f	TATAGTCACAGGTGGAAGCGAGGT
Mouse-Slc6a4-sg3-OfT23-r	TTCTCTTTGCTAGGGATCGTGTCAG
Mouse-Slc6a4-sg3-OfT24-f	AGTGATGGTCCTACACTGTGAATGT
Mouse-Slc6a4-sg3-OfT24-r	TTATAGGATGCCATGTCCAGATTGC
Mouse-Slc6a4-sg3-OfT25-f	AGGGAATAGAGGGAAGCCATAGAGG
Mouse-Slc6a4-sg3-OfT25-r	AAGCAGTTCTGTGACATAAGCAACC
Mouse-Slc6a4-sg3-OfT26-f	AGATTCCTGTCCTGACTTCCATTGT
Mouse-Slc6a4-sg3-OfT26-r	GCTGCTCAATAGCTTCAGCATGAA
Mouse-Slc6a4-sg3-OfT27-f	TGTCTGTCTAGCATGAGTTCTCACA

Mouse-Slc6a4-sg3-OfT27-r	GAAATCTTCCTACCTCTGCCTCCAA
Mouse-Slc6a4-sg3-OfT28-f	CAGAAGAAGGCATCAGATGGTTGTG
Mouse-Slc6a4-sg3-OfT28-r	GCAGGATTCAAACTCAGGTGGAAAG
Mouse-Slc6a4-sg3-OfT29-f	TTCCATCTCCCAAGCACTGAAACTC
Mouse-Slc6a4-sg3-OfT29-r	AGCAGGCAAGCGTTAACTCATTCT
Mouse-Slc6a4-sg3-OfT30-f	GGTGTTTGTTGGAGCATAGGTCTGT
Mouse-Slc6a4-sg3-OfT30-r	CTTGCCAGTCCTTCCAGGTTCATC
Mouse-Slc6a4-sg3-OfT31-f	ATCAGTTCTAAGACCCACGCCAAG
Mouse-Slc6a4-sg3-OfT31-r	CCAGCAAGACAGCTCAGCAAGTA
Mouse-Slc6a4-sg3-OfT32-f	CTGGCAAGTGGCTCATCAGACAA
Mouse-Slc6a4-sg3-OfT32-r	CCCACAGCTCTTCAGGATCAGTC
Mouse-Slc6a4-sg3-OfT33-f	GTGGCAATGCTTACTTGACTATGGC
Mouse-Slc6a4-sg3-OfT33-r	ATGACTGTGGAATGTGCTCTCCTG
Mouse-Slc6a4-sg1-OfT1'-f	AGCTGACCGGCAGCAAAATTGctcctccttcccacacacctatcag
Mouse-Slc6a4-sg1-OfT1'-r	GGAAAAGCTGCCTCTCAAAGGAAAC
Mouse-Slc6a4-sg1-OfT2'-f	AGCTGACCGGCAGCAAAATTGctgcttcttcctaaggaggctgatg
Mouse-Slc6a4-sg1-OfT2'-r	GTCACCAATTCTAATTCATCATAGCTG
Mouse-Slc6a4-sg1-OfT3'-f	AGCTGACCGGCAGCAAAATTGagaaggctgtaatctgacctgagac
Mouse-Slc6a4-sg1-OfT3'-r	GCCAGTTAGTAGGAGGTGGGAATTTC
Mouse-Slc6a4-sg2-OfT1'-f	AGCTGACCGGCAGCAAAATTGtacagctcctcagtcaattcgtgcat
Mouse-Slc6a4-sg2-OfT1'-r	TGCTGGAACAATGAATGGGTCTGA
Mouse-Slc6a4-sg2-OfT2'-f	AGCTGACCGGCAGCAAAATTGaactggttccaagtgagcttcctt
Mouse-Slc6a4-sg2-OfT2'-r	CAGTACACCAAATGCCAGCTATATTAC
Mouse-Slc6a4-sg2-OfT3'-f	AGCTGACCGGCAGCAAAATTGgatgagggatggagcagagactga
Mouse-Slc6a4-sg2-OfT3'-r	TTGGTTGGTGGTTCAGTCTCTTCTC
Mouse-Slc6a4-sg3-OfT1'-f	AGCTGACCGGCAGCAAAATTGtagggagatggctcagcaggtcaa
Mouse-Slc6a4-sg3-OfT1'-r	CCCATACTGACCTTGAACTCCTGAT
Mouse-Slc6a4-sg3-OfT2'-f	AGCTGACCGGCAGCAAAATTGggtaaagaacgttcctgagtttgatg
Mouse-Slc6a4-sg3-OfT2'-r	CTGTGTTTCCAAGGCTCCTTCCA
Mouse-Slc6a4-sg3-OfT3'-f	AGCTGACCGGCAGCAAAATTGcatatactccctctactctgaacacC
Mouse-Slc6a4-sg3-OfT3'-r	GCAGCATAGGAAGATAGCACAATT
Mouse-Slc6a4-sg3-OfT4'-f	AGCTGACCGGCAGCAAAATTGtcaggacaacgatgggagtgatct
Mouse-Slc6a4-sg3-OfT4'-r	GATAATCTGGCCACACTTTGATCAGC
Mouse-Slc6a4-sg3-OfT5'-f	AGCTGACCGGCAGCAAAATTGactgaactccattgctttgccatct
Mouse-Slc6a4-sg3-OfT5'-r	GTTAAGCTGCTTTCTTTGAGGCTTCC
Mouse-Slc6a4-sg3-OfT6'-f	AGCTGACCGGCAGCAAAATTGgcctggaattggtgtctcctctg
Mouse-Slc6a4-sg3-OfT6'-r	CTTTTTCAGTCGGTGCCAATGCTG
Rat-Drd2-sg1-OfT1-f	AGCTGACCGGCAGCAAAATTGgcccagacttcctttagggacaga
Rat-Drd2-sg1-OfT1-r	GGCCCTGCTGTTTCTCTGATGTG
Rat-Drd2-sg1-OfT2-f	AGCTGACCGGCAGCAAAATTGccatcagctccttctacattcttatc
Rat-Drd2-sg1-OfT2-r	GTTAAGGTCAGGTTGGGGCAAAGTC
Rat-Drd2-sg1-OfT3-f	AGCTGACCGGCAGCAAAATTGctctagtcagggacatgagctcag
Rat-Drd2-sg1-OfT3-r	GCAGTTAGGGATGGTAACTCTTGC
Rat-Drd2-sg2-OfT1-f	AGCTGACCGGCAGCAAAATTGcagggatgatatcaacttggcacag
Rat-Drd2-sg2-OfT1-r	CAGTGGGGATTCTGGCAGCAAAC
Rat-Drd2-sg2-OfT2-f	AGCTGACCGGCAGCAAAATTGggtgtgagaaggaaaactggactg
Rat-Drd2-sg2-OfT2-r	GTATTTCCTTGGGGATAGATAGCTGC
Rat-Drd2-sg2-OfT3-f	AGCTGACCGGCAGCAAAATTGcgaagttcatattaatgtgcattggt
Rat-Drd2-sg2-OfT3-r	GTACACATACATACATGCAGGCAAAAC
Human-Rpl41-OnT-f	AGCTGACCGGCAGCAAAATTGgagtgttttctccctggagccagg
Human-Rpl41-OnT-r	CAGAGGGCGATGAAGTTCTAGATCC
Human-Rpl41-sg1-OfT1-f	AGCTGACCGGCAGCAAAATTGcagagagggacaagaggtggtag
Human-Rpl41-sg1-OfT1-r	TGCCTAAAGTCGCATAAGCAG
Human-Rpl41-sg2-OfT1-f	AGCTGACCGGCAGCAAAATTGtctcagaggtgatcggcgatc
Human-Rpl41-sg2-OfT1-r	GGAAACCTCTGTGCCATGAGAGC
Human-Rpl41-sg2-OfT2-f	AGCTGACCGGCAGCAAAATTGatcttgctctcggccttagcg
Human-Rpl41-sg2-OfT2-r	GCATGCAGTCCCACAACTTGTAC
Human-Rpl41-sg2-OfT3-f	AGCTGACCGGCAGCAAAATTGcggcgatcagagggtgatgaagt
Human-Rpl41-sg2-OfT3-r	TACTCAGGCTCTCGACCTTAGC
Human-Rpl41-sg2-OfT4-f	AGCTGACCGGCAGCAAAATTGcctagaagagcgtccaaggtgg
Human-Rpl41-sg2-OfT4-r	GAGGGCGGTGAAGTTCTGGAT

Human-Rpl41-sg2-OfT5-f	AGCTGACCGGCAGCAAAATTGgttatgagcgaggtgggtctc
Human-Rpl41-sg2-OfT5-r	CCTCTGTGCCATGACAGCCAAG
Human-TUFm-OnT-f	AGCTGACCGGCAGCAAAATTGaaggaatgaaggcaccctggagg
Human-TUFm-OnT-r	GGGAAATGTCCATCTAGCTGCC

Supplementary Table S6 NEO system enables highly efficient homologous recombination in zebrafish and mammals – Summary Table

	**Donor DNA**	**Recipes**	**Nature of DNA lesions (distance between nicks, bp)**	**Number of fish screened. (without pre-selection)**	**Founder with HR mediated KI**	**Rate of germline transmission**
**Species**						
Zebrafish	GFAP-p2A-ChR2-EYFP	Cas9n only	*Trans*-dual nicks (74)	37	1	2.7%
		Cas9n+NEO	*Trans*-dual nicks (74)	27	3	11.1%
		Cas9n only	*Cis*-dual nicks (52)	37	0	0
		Cas9n+NEO	*Cis*-dual nicks (52)	32	3	9.4%
	Ndr2-linker-dendra2- V5	Cas9 only	Ndr2-Sg1 (DSB)	50	2	4%
		Cas9+RecOFAR	Ndr2-Sg1 (DSB)	62	9	14.5%
		Cas9n only	*Trans*-dual nicks (55)	155	4	2.6%
		Cas9n+RecA	*Trans*-dual nicks (55)	50	1	2%
		Cas9n+RecORF	*Trans*-dual nicks (55)	52	1	1.9%
		Cas9n+RecOR	*Trans*-dual nicks (55)	54	0	0
		Cas9n+RecAOR	*Trans*-dual nicks (55)	53	1	1.9%
		Cas9n+RecAF	*Trans*-dual nicks (55)	65	2	3.1%
		Cas9n+NEO	*Trans*-dual nicks (55)	86	8	9.3%
	Lefty2-linker-dendra2-V5	Cas9n only	*Trans*-dual nicks (99)	62	0	0
		Cas9n+NEO	*Trans*-dual nicks (99)	59	6	10.2%
	BMP2b-linker-dendra2-V5	Cas9n only	*Trans*-dual nicks (77)	78	2	2.6%
		Cas9n+NEO	*Trans*-dual nicks (77)	81	7	8.6%
		Cas9 only	BMP2b-Sg1 (DSB)	50	0	0
		Cas9+RecOFAR	BMP2b-Sg1 (DSB)	56	2	3.6%
	GFAP-5.5kb	Cas9n only	*Trans*-dual nicks (74)	90	1	1.1%
		Cas9n+NEO	*Trans*-dual nicks (74)	86	6	7.0%
		**Recipes**	**Nature of DNA lesions (distance between nicks, bp)**	**Transferred embryos after injection**	**Postnatal**	**Knock-in (F_0)**
Mice	Slc6a4-p2A-ChR2-EYFP	Cas9n+NEO	*Trans*-dual nicks (80)	296	113	7 (6.2%)
		Cas9n+NEO	*Cis*-dual nicks (63)	351	158	3 (1.9%)
		Cas9n only	*Trans*-dual nicks (80)	296	110	2 (1.8%)
		Cas9n only	*Cis*-dual nicks (63)	216	100	1 (1.0%)
		Cas9+RecOFAR	DSB	175	61	1 (1.6%)
		Cas9 only	DSB	349	154	3 (1.9%)

	Myc-sfGFP	Cas9n+NEO	*Trans*-dual nicks (93)	180	13	2 (15.4%)
		Cas9n only	*Trans*-dual nicks (93)	135	32	1 (3.1%)
Rat	GFAP-p2A-mEYFP	Cas9n+NEO	*Trans*-dual nicks (75)	212	58	5 (8.6%)
		Cas9n only	*Trans*-dual nicks (75)	146	35	0
	Drd2-p2A-chR2::EYFP	Cas9n+NEO	*Trans*-dual nicks (72)	775	156	21(13.5%)
		Cas9n only	*Trans*-dual nicks (72)	274	99	3(3.0%)
		Cas9+RecOFAR	DSB	241	77	2(2.6%)
		Cas9 only	DSB	184	39	1(2.6%)
	Drd1-p2A-chR2::EYFP	Cas9n+NEO	*Trans*-dual nicks (42)	323	98	10 (10.2%)
	Bassoon-5.5 kb	Cas9n+NEO	*Trans*-dual nicks (99)	103	30	2 (6.7%)
Monkey	CAMK2A-p2A-ChR2：：EYFP	Cas9n+NEO	*Trans*-dual nicks	20	10	50%
	EGFP-2a1-NeoR-2a2-OCT4	Cas9n+NEO	*Trans*-dual nicks	11	3	27.3%

Supplementary Table S7 Toxicity assessment of RecOFAR mRNAs, CRISPR/Cas9 ingredients and donor DNA in zebrafish embryos[a]

	Embryos injected	Normal embryos	Percentages
**Recipes**			
RecOFAR mRNAs (100 ng/µl each)	122	109	89.34%[n.s.]
GFAP-p2A-ChR2-EYFP donor DNA (300 ng/µl)	134	74	55.22%[**]
Cas9 mRNA (100 ng/µl)	129	100	77.52%[**]
ddH$_2$O	104	96	92.31%

Chi-square with Fisher's test was performed. Pairwise comparisons were made between RecOFAR mRNA, GFAP donor plasmid, Cas9 mRNA and ddH2O alone, respectively.

[a] Two independent microinjection experiments were conducted and evaluated together.

[n.s.] no significant difference.

[**] indicates significant difference.

Supplementary Table S8 The mosaicism of germline transmission in the founder fish, mice and rats produced by NEO system

	F_1 individuals evaluated	Positive individuals	% of germline with a homologous recombination event
**Fish GFAP-p2A-ChR2-EYFP target KI (Cas9n+NEO, *cis*-dual nicks) Fish**			
#♂6	50	3	6.0
#♂10	80	10	12.1
#♂29	74	1	1.4
**Ndr2-linker-dendra2-V5 target KI founder (Cas9n+NEO, *trans*-dual nicks)**			
#♂2	65	2	3.1
#♂8	81	3	3.7
#♀4	122	3	2.5
#♀8	120	2	1.7
**Fish GFAP-5.5kb target KI (Cas9n+NEO, *trans*-dual nicks)**			
#♀30	86	4	4.7
**Mouse Slc6a4-p2A-ChR2-EYFP target KI (Cas9n+NEO, *trans*-dual nicks)**			
♀	6	1	16.7
♂	21	11	52.4
**Mouse Slc6a4-p2A-ChR2-EYFP target KI (Cas9n+NEO, *cis*-dual nicks)**			
♂	17	12	70.6
♂	18	1	5.6
**Mouse Myc-linker-sfGFP target KI (Cas9n+NEO, *trans*-dual nicks)**			
♂	26	7	26.9
**Rat GFAP-p2A-mEYFP target KI (Cas9n+NEO, *trans*-dual nicks)**			
♂	25	2	8.0
♂	28	1	3.6
**Rat Drd1-p2A-ChR2-EYFP target KI (Cas9n+NEO, *trans*-dual nicks)**			
♂	28	7	25.0
♂	17	5	29.4
♂	14	4	28.6
♂	12	4	33.0

Supplementary Text S1

Plasmid backbone

CTAAATTGTAAGCGTTAATATTTTGTTAAAATTCGCGTTAAATTTTTGTTAAATCAGCTCATTTTTTAACCAATAGGC
CGAAATCGGCAAAATCCCTTATAAATCAAAAGAATAGACCGAGATAGGGTTGAGTGTTGTTCCAGTTTGGAACAAGAG
TCCACTATTAAAGAACGTGGACTCCAACGTCAAAGGGCGAAAAACCGTCTATCAGGGCGATGGCCCACTACGTGAACC
ATCACCCTAATCAAGTTTTTTGGGGTCGAGGTGCCGTAAAGCACTAAATCGGAACCCTAAAGGGAGCCCCCGATTTAG
AGCTTGACGGGGAAAGCCGGCGAACGTGGCGAGAAAGGAAGGGAAGAAAGCGAAAGGAGCGGGCGCTAGGGCGCTGGC
AAGTGTAGCGGTCACGCTGCGCGTAACCACCACACCCGCCGCGCTTAATGCGCCGCTACAGGGCGCGTCCCATTCGCC
ATTCAGGCTGCGCAACTGTTGGGAAGGGCGATCGGTGCGGGCCTCTTCGCTATTACGCCAGCTGGCGAAAGGGGGATG
TGCTGCAAGGCGATTAAGTTGGGTAACGCCAGGGTTTTCCCAGTCACGACGTTGTAAAACGACGGCCAGTGAGCGCGC

Kpn I **5' arm**

GTAATACGACTCACTATAGGGCGAATTGGGTACCGGTGCAATATTTATTTAAAAAAGTATGTTTATTGTAAGATTTTT
ATGAACATCTATTAACATAAACAATGAACAATATATGTATTATATTACTTTATAATCTTTGTCAAATGTTAGTTGAAA

***gfap* 8rd intron sequence**

TACAGCTATTCATTATTTAAGTTAGCTTCAATCCATTAAATAATATCAACATACAGCAAAATCCTCTGAGCAAAATTT
ACTCACTTCAGAGAGTAAATGTATCTCTTTAAATAAGGGCAAACAGGTTGGTAATAATTAAAGCTGCTGGTTTTGGAG
TTGCTTAATGATCCTCTGCTGAGATTTCAAAAGAGATTCCATGTACTATATTTCAGCTGATTTCATCTAAGGATGTGA
CTGATTTCAGTTTTAGTTTTGTGTTTCTCTTCTGTGTTTCTGTTTATTAACAACAACAATCAGTGCTCAATCATCCCC
CAATAAATCCTTTCATTTTTTCATAAACATGAGTAACTTTAACACTTTAAAAAGAACCAAATACTGTCTAAACTGAGT
AAAATTTACTCAGAGGATTTTGCTGTGTAACTTTTTTACTTAATGATTTAGTTCATATTAACTAATTTACTCTATATT
CCAGTCAATAAATTTGTATTCCTGTCTACAATGTAATATTCTCTTTCACTGTAAAAGAATTCAGGATCCCACACAACT
CAAATAAATTAACTTTTTTACTAATTTTTTTAGGGTTTTTTCACCTTTATTTGGTGTAGGGCAGTGGAGGTTACAGAC
TGGATAGTATTGGGAGCAGAGAGGGGGGAAGGGTTGGCAAAGGACCTTAAGCCGAGAATCGAACTCGGATCACCATAA
GAACCATGGTGCTATGTGTTGCGCACTTAACCACTAGGCTATAGGCGCCGACCGATTAAGTTAACTTCAATTAAAGTG
GATTAAACATAAAACAATTAAGTTGTCCCCAAAAACATCTCAAGAATTGTGTTGATTCTGCTCATTCTTCATTTTACA

PAM 1 ***gfap* 9rd exon sequence** **In-frame silent mutation**

CATTCTCTCTTTCGCAG**ATCATTAAAGAGTCCACTA^CC(T*).GAA(E*).CGT(R*).AAA(K*).GAC(D*).CT**

P2A

A(L*).CCG(P*).GGATCCGGAGCTACTAATTTCTCCTTGCTTAAGCAAGCTGGTGATGTTGAAGAAAATCCTGGTC
CT**ATGGACTATGGCGGCGCTTTGTCTGCCGTCGGACGCGAACTTTTGTTCGTTACTAATCCTGTGGTGGTGAACGGGT**

ChR2-EYFP

CCGTCCTGGTCCCTGAGGATCAATGTTACTGTGCCGGATGGATTGAATCTCGCGGCACGAACGGCGCTCAGACCGCGT
CAAATGTCCTGCAGTGGCTTGCAGCAGGATTCAGCATTTTGCTGCTGATGTTCTATGCCTACCAAACCTGGAAATCTA
CATGCGGCTGGGAGGAGATCTATGTGTGCGCCATTGAAATGGTTAAGGTGATTCTCGAGTTCTTTTTTGAGTTTAAGA
ATCCCTCTATGCTCTACCTTGCCACAGGACACCGGGTGCAGTGGCTGCGCTATGCAGAGTGGCTGCTCACTTGTCCTG
TCATCCTTATCCGCCTGAGCAACCTCACCGGCCTGAGCAACGACTACAGCAGGAGAACCATGGGACTCCTTGTCTCAG
ACATCGGGACTATCGTGTGGGGGGCTACCAGCGCCATGGCAACCGGCTATGTTAAAGTCATCTTCTTTTGTCTTGGAT
TGTGCTATGGCGCGAACACATTTTTTCACGCCGCCAAAGCATATATCGAGGGTTATCATACTGTGCCAAAGGGTCGGT
GCCGCCAGGTCGTGACCGGCATGGCATGGCTGTTTTTCGTGAGCTGGGGTATGTTCCCAATTCTCTTCATTTTGGGGC
CCGAAGGTTTTGGCGTCCTGAGCGTCTATGGCTCCACCGTAGGTCACACGATTATTGATCTGATGAGTAAAAATTGTT
GGGGGTTGTTGGGACACTACCTGCGCGTCCTGATCCACGAGCACATATTGATTCACGGAGATATCCGCAAAACCACCA
AACTGAACATCGGCGGAACGGAGATCGAGGTCGAGACTCTCGTCGAAGACGAAGCCGAGGCCGGAGCCGTGCCAGCGG
CCGCCACCATGGTGAGCAAGGGCGAGGAGCTGTTCACCGGGGTGGTGCCCATCCTGGTCGAGCTGGACGGCGACGTAA
ACGGCCACAAGTTCAGCGTGTCCGGCGAGGGCGAGGGCGATGCCACCTACGGCAAGCTGACCCTGAAGTTCATCTGCA
CCACCGGCAAGCTGCCCGTGCCCTGGCCCACCCTCGTGACCACCTTCGGCTACGGCCTGCAGTGCTTCGCCCGCTACC
CCGACCACATGAAGCAGCACGACTTCTTCAAGTCCGCCATGCCCGAAGGCTACGTCCAGGAGCGCACCATCTTCTTCA
AGGACGACGGCAACTACAAGACCCGCGCCGAGGTGAAGTTCGAGGGCGACACCCTGGTGAACCGCATCGAGCTGAAGG

GCATCGACTTCAAGGAGGACGGCAACATCCTGGGGCACAAGCTGGAGTACAACTACAACAGCCACAACGTCTATATCA

TGGCCGACAAGCAGAAGAACGGCATCAAGGTGAACTTCAAGATCCGCCACAACATCGAGGACGGCAGCGTGCAGCTCG

CCGACCACTACCAGCAGAACACCCCCATCGGCGACGGCCCCGTGCTGCTGCCCGACAACCACTACCTGAGCTACCAGT

Stop codon sgRNA2 target Mutation

CCGCCCTGAGCAAAGACCCCAACGAGAAGCGCGATCACATGGTCCTGCTGGAGTTCGTGACCGCCGCCGGGATCACTC

PAM 2 3’ arm

TCGGCATGGACGAGCTGTACAAGTAATGAGGCTCAGACACTGGCTTGGCTGCAGAAGAAGATCTCCTTGTGATTACGT

TCAGGAAGCTGTGTTTGTCAAACCTTGTATTTTCTCACCAACTTTCCCTGCTTCTCTCCTGGTATATATAGTTCATGG

AAACTGTATTCACTGTTTTTAGCAACTACCTATATGTTTTGATCTCCTTTTCTTCCTGGGCTTCTCTTTTTATCTGTC

TGTTTCTTCTGTGGCAGAGTCCTTGCAAGATAGTAGACTAGTTTATCGCAGAATGTGCTATAAAAAGTCATGGTTGGA

CAGATGAAAGTAAACAGTAGGACAAGATGTAGCTAGTGGTCAGAGCGGGTGGGAAATATAGTGTTTTTTTTTATCAAG

CATAAATTGTAGAGAACAAGTTCTTGAGCTTTATTGTAGTCTTTAAGAATGCCAGAAGAAAAAAAGAGCAAGTCTGGA

GCTGGTAGTTTTTAAATCTGAACAGTAGGTGTTAAAGTAAGGCAGAAGGGAATTTTAACAGCAAGATGTTTGTAAGAG

TTTTAAAGGAATGATGGATTTGGTCAGCGGTGCTATTTGAACATTTTACTCCACATTTTTTTTGTCCTCCTCCACTGT

GTGTGTGCGCAGTGTTTTTGCACAGTAGCTAACTCATCTCTTGGACCCAGGTTAAAATGCTTTAAATTGCTCGTTGAC

ACATTACTGACCTGTAGAAGACATTAAAATCTTAACATTAAGCATCTTACTGAATGAATTATGGTATAAATATCTGAC

ACAGTGTTCCCCATCATGTGACCAGATCATTTGATTCAGATTTATACTGATAGCTTTCAATTGTCACGCTACATTTCC

TTGGTGAAATTGAGCAATTTTTCCATTTCCATCTGTGTGATCAGGCATTTTTGCATGAGGGTGAAAGGTTCCTCCTGC

Sac II

AGTTTATGCTTACATTTGAGTTGTTATTCTGAATTGCACAAATATCATCTTGGTTTGAAAGTGACTTAAGTGTGAGCA

Plasmid backbone

GTGTTTCACACATCACTACAATGTTAATAATATGCATTAATAATAATCAAAACTGTGACCATATCTTCACCCGCGGTG

GAGCTCCAGCTTTTGTTCCCTTTAGTGAGGGTTAATTGCGCGCTTGGCGTAATCATGGTCATAGCTGTTTCCTGTGTG

AAATTGTTATCCGCTCACAATTCCACACAACATACGAGCCGGAAGCATAAAGTGTAAAGCCTGGGGTGCCTAATGAGT

GAGCTAACTCACATTAATTGCGTTGCGCTCACTGCCCGCTTTCCAGTCGGGAAACCTGTCGTGCCAGCTGCATTAATG

AATCGGCCAACGCGCGGGGAGAGGCGGTTTGCGTATTGGGCGCTCTTCCGCTTCCTCGCTCACTGACTCGCTGCGCTC

GGTCGTTCGGCTGCGGCGAGCGGTATCAGCTCACTCAAAGGCGGTAATACGGTTATCCACAGAATCAGGGGATAACGC

AGGAAAGAACATGTGAGCAAAAGGCCAGCAAAAGGCCAGGAACCGTAAAAAGGCCGCGTTGCTGGCGTTTTTCCATAG

GCTCCGCCCCCCTGACGAGCATCACAAAAATCGACGCTCAAGTCAGAGGTGGCGAAACCCGACAGGACTATAAAGATA

CCAGGCGTTTCCCCCTGGAAGCTCCCTCGTGCGCTCTCCTGTTCCGACCCTGCCGCTTACCGGATACCTGTCCGCCTT

TCTCCCTTCGGGAAGCGTGGCGCTTTCTCATAGCTCACGCTGTAGGTATCTCAGTTCGGTGTAGGTCGTTCGCTCCAA

GCTGGGCTGTGTGCACGAACCCCCCGTTCAGCCCGACCGCTGCGCCTTATCCGGTAACTATCGTCTTGAGTCCAACCC

GGTAAGACACGACTTATCGCCACTGGCAGCAGCCACTGGTAACAGGATTAGCAGAGCGAGGTATGTAGGCGGTGCTAC

AGAGTTCTTGAAGTGGTGGCCTAACTACGGCTACACTAGAAGGACAGTATTTGGTATCTGCGCTCTGCTGAAGCCAGT

TACCTTCGGAAAAAGAGTTGGTAGCTCTTGATCCGGCAAACAAACCACCGCTGGTAGCGGTGGTTTTTTTGTTTGCAA

GCAGCAGATTACGCGCAGAAAAAAAGGATCTCAAGAAGATCCTTTGATCTTTTCTACGGGGTCTGACGCTCAGTGGAA

CGAAAACTCACGTTAAGGGATTTTGGTCATGAGATTATCAAAAAGGATCTTCACCTAGATCCTTTTAAATTAAAAATG

AAGTTTTAAATCAATCTAAAGTATATATGAGTAAACTTGGTCTGACAGTTACCAATGCTTAATCAGTGAGGCACCTAT

CTCAGCGATCTGTCTATTTCGTTCATCCATAGTTGCCTGACTCCCCGTCGTGTAGATAACTACGATACGGGAGGGCTT

ACCATCTGGCCCCAGTGCTGCAATGATACCGCGAGACCCACGCTCACCGGCTCCAGATTTATCAGCAATAAACCAGCC

AGCCGGAAGGGCCGAGCGCAGAAGTGGTCCTGCAACTTTATCCGCCTCCATCCAGTCTATTAATTGTTGCCGGGAAGC

TAGAGTAAGTAGTTCGCCAGTTAATAGTTTGCGCAACGTTGTTGCCATTGCTACAGGCATCGTGGTGTCACGCTCGTC

GTTTGGTATGGCTTCATTCAGCTCCGGTTCCCAACGATCAAGGCGAGTTACATGATCCCCCATGTTGTGCAAAAAAGC

GGTTAGCTCCTTCGGTCCTCCGATCGTTGTCAGAAGTAAGTTGGCCGCAGTGTTATCACTCATGGTTATGGCAGCACT

GCATAATTCTCTTACTGTCATGCCATCCGTAAGATGCTTTTCTGTGACTGGTGAGTACTCAACCAAGTCATTCTGAGA

ATAGTGTATGCGGCGACCGAGTTGCTCTTGCCCGGCGTCAATACGGGATAATACCGCGCCACATAGCAGAACTTTAAA
AGTGCTCATCATTGGAAAACGTTCTTCGGGGCGAAAACTCTCAAGGATCTTACCGCTGTTGAGATCCAGTTCGATGTA
ACCCACTCGTGCACCCAACTGATCTTCAGCATCTTTTACTTTCACCAGCGTTTCTGGGTGAGCAAAAACAGGAAGGCA
AAATGCCGCAAAAAAGGGAATAAGGGCGACACGGAAATGTTGAATACTCATACTCTTCCTTTTTCAATATTATTGAAG
CATTTATCAGGGTTATTGTCTCATGAGCGGATACATATTTGAATGTATTTAGAAAAATAAACAAATAGGGGTTCCGCG
CACATTTCCCCGAAAAGTGCCAC

Supplementary Text S1. Sequence of zebrafish *gfap* targeting construct *p2A-ChR2-EYFP*. The donor backbone is indicated with black lines, homology arms with purple lines, and *ChR2-EYFP* with red lines. The exon sequences of *gfap* gene are in bold and highlighted, and the gRNA1 and sgRNA3 targeting sites were replaced by in-frame silent mutations. The mutated sequences of the sgRNA2 targeting sites are marked in green.

Supplementary Text S2

…… CCAGCTGGCGAAAGGGGGATGTGCTGCAAGGCGATTAAGTTGGGTAACGCCAGGGTTTTCCCAGTCACGACGT

Plasmid backbone ***Kpn* I** **5' arm**

TGTAAAACGACGGCCAGTGAGCGCGCGTAATACGACTCACTATAGGGCGAATTGGGTACCGTGCTAAAATAAAAATAA
TCTGTAAATAAATCCTTGCTGCTATTTTCTTTAGTTCAAACAACTTAATATTTTAGTCAGTTTGGCGTAGGTAAATCG

***lefty2* 3rd intron sequence**

AGATGACTAGAAATTTCGTATAACTTAATTAATTAAAATAAGTAAACTAAACATCCAATACTTTAAAGAATAATTGAA
TAAATATTTAAAAATAAATAAATAAGTGGGTTCAAATACAGAATATATAAATAAAATATAAATATAAATATTAAAATA
ATAAATTATTTAAATTAAAATAATATTATTTTTTGAAGTATATTTAATTTTATTTTCCTTTTGTGCTAAAATAAAAAA
AATATCTGTAAATAAATTCTTGCTTCCTAAATTTTATTTAGCTGAATCAACTTAAATTTTCTAGTCACCTCAACTTAC
AGAAGTCAATCTGACTAAACATATTGTGTTAAACTTTGTAAAGTTGTAACTTGATTAACCAAATTAAAATAAGTAACC
TAAATATTAAACAATATTAATATTAAATACTATCAATAACATTTGTAGTCACGACTTTTTGTCTCACATTTTTCATGG
CACATAATGAAGTCTGTCACAAAACACAGTATAGTGTAGAAACCATTTAAACGTACTTGATTTAATGGCTCAAATGTT
ATTTTGTCTTGAAAAACATTAGAAACTCTGTAATAATTCCTATTTATTATTATCTTTATTATATGATTTGATGCATTT

***lefty2* 4rd exon sequence**

TTCAGTTATATAGGTTATATAAACTAACAAGTATCCTTTACTGTCTTATCAACAGATCCCAAGGCAACTGTAACTCCA
GCCCAAACAGCAGCAAATGTTGTCGGGAGGAGCATTTCATCAACTTCCGTGAGCTCACCTGGACTCAGTACTGGATCA
TTGAGCCGGCCGGGTACCAGGCGTTCAGGTGCGCTGGAGGATGCAAGCAGCCCAAACGTGGTTTCTATGGATATGGAC

PAM 1 **In-frame silent mutation**

AGAGGACATGTGCAGTGATGGAGAGCGCGCCGCTGCCCATGATGTACCTGGTG (V*) AAG (K*) AAA (K*) GGT (G*)
GAC (D*) TAT (Y*) ACTGAAATCGAGGTGGCAGAATTCCCTAATATGATTGTGGAGAAGTGTGGATGCTCCATGGACA

linker-Dendra2

AAATCCCTCCAGTCGGCGGAGGTTCCGGTGGAAGCGGAGGTAGCGGCGGAAACACCCCCGGCATCAACCTGATCAAGG
AAGATATGCGCGTCAAGGTGCACATGGAGGGCAACGTGAACGGCCACGCCTTCGTGATCGAGGGCGAGGGCAAGGGAA

V5

AGCCCTACGAGGGCACCCAGACCGCCAACCTGACCGTGAAGGAGGGAGCCCCGCTGCCCTTCAGCTACGATATCCTGA
CCACCGCCGTGCACTACGGCAACCGCGTGTTCACCAAGTACCCCGAGGATATCCCCGATTACTTCAAGCAGAGCTTCC
CCGAGGGCTACAGCTGGGAGCGCACCATGACCTTCGAGGATAAGGGCATCTGCACCATCCGCAGCGATATCAGCCTGG
AGGGCGATTGCTTCTTCCAGAACGTGCGCTTCAAGGGCACCAACTTCCCCCCCAACGGCCCCGTGATGCAGAAAAAGA
CCCTGAAGTGGGAGCCCAGCACCGAGAAGCTGCACGTGCGCGACGGCCTGCTGGTCGGCAACATCAACATGGCCCTGC
TGCTGGAGGGCGGAGGACACTACCTGTGCGATTTCAAGACCACCTACAAGGCCAAGAAGGTGGTCCAGCTGCCCGATG
CCCACTTCGTGGATCACCGCATCGAGATCCTGGGCAACGATAGCGATTACAACAAGGTGAAGCTGTACGAGCACGCCG
TGGCCCGCTACAGCCCCCTGCCCAGCCAGGTGTGGAAGCGGCGGGGCAAGCCGATCCCGAATCCACTGCTCGGACTGG

Stop codon **PAM 2**

ATAGCACCTAA^GTGTGGTGTCGAATAGTTTGCTCTCAGATGAGAGGTGACGTGTTTAATAATCACAGTTATGGGACT

Deletion of "TGACA"

CCCCAAATCCCCACTGAGCACTTGAAAAACAAAGTATTTATGAATTATTGCTTTATGTAATGTAAGCTTTAGAAATGG

3' arm

CTTGTTGAGAGATGTGATGTTTGAAAAATAAAGTAAGTAGCTTAATTTTATATCAATGAGGGTCGTCATTTTTTTATT
TCTAACATGATTCTTCATTCATTCATTTTCCTTCGGCTTAATCCCTTTATTCATTAGGGGTCACCACAGCGGAATGAA
CCGCCAACTTATCAAGCATATGTTTTACACAGCGCATATTCTTCTCGCTGCAACTTAGAACTAGGAAAACACCCATAC
ACTCTCACTTTCGCAGTCATACACTACGGCCAATTTAGTTTTGTTCCAGTGGTTCTCAAACTGTTTGTGTATTAGTGG
TACGCAGGCTTACTTCTAGGCTGTTCTCTAAAGCCTGCGACCAAAGTGCTGTGCTTAGAATGTTTACTTTATTGCATC
AGGTCATCAGCTGCTAAAAATACATGTAATTGTCGCAGAACTAGAGAAAGGTGCGTGGTGCTTGCAGAGCAGATAAGT
TATATTGGTACCAGGCGGCCCAAAAAATCCTTAATTATTTCGGTTTTGTTAATAATCTGAGTCTGAACGATCATTTAT
TTTGAAAAATCTTTTATTTTGAAAAATGACCGGATTCTCGTTACATCTCAGTCACTTGAATAACAATATTTAACCCAC
AGGCTAGCAAAATGAGTAAGCTCGGCGCGCTTGCCTATTCCAGGCGTACGCAGTTGAAAGCACCTTAACTTTTCAGAA
TGCTGCACCGCGAGTCACGCTACAAGAACCGACTGATCAGATTCATATTTTGTATGGAATATAATTTCGTTGAATGAA
TATGACCTTCTTGTTTTCGAACTTAGCAGTGCTGTAGCTGCTTAATTTGGCAAACTACGCTACTTTATTTCCGCGGTG
GAGCTCCAGCTTTTGTTCCCTTTAGTGAGGGTTAATTGCGCGCTTGGCGTAATCATGGTCATAGCTGTTTCCTG……

Supplementary Text S2. Sequence of zebrafish *lefty2* targeting construct *linker-Dendra2*. The donor backbone is indicated with black lines, homology arms with purple lines, and *linker-Dendra2* with red lines. The exon sequences of *lefty2* gene are in bold and highlighted. The mutated sequences of the sgRNA1 targeting sites are marked in green. The plasmid backbone sequences are the same as Gfap-p2A-ChR2-EYFP targeting donor showed in **Supplementary Text S1**.

Supplementary Text S3

Plasmid backbone

...... CCAGCTGGCGAAAGGGGGATGTGCTGCAAGGCGATTAAGTTGGGTAACGCCAGGGTTTTCCCAGTCACGACGT

Kpn I 5' arm

TGTAAAACGACGGCCAGTGAGCGCGCGTAATACGACTCACTATAGGGCGAATTGGGTACCTTTTTTCAGCTCACATAA

ndr2 1rd intron sequence *ndr* 2rd exon sequence

TAACCTTTTTAATTTATTTTTCTGTCTTCTCCCAGATGTGCTCAACAATGACAACCACTACGTGGCGATCTTTGACCT

GTCTCCGGTTTTGTCGGAACGTCAGATTCAGGCAGCGGAGCTGCGGATCCGCGTCCCTAGAGACCTGCACCCCGATGG

GCTGACATTGGAGCTCCAGCACCAGCAGGGGGCGCCGTGCCCTCGTCACCAGCCCTGCCCGAAGAGCCAGTCTCTGGG

CCTTTTGCCGGAGGAGTCCCTGCTGAGCGTAACACAGCACTGGAGGGTGTATAATGTCACAAATCTGCTTCTGCACTG

GCCCCGGCCCCGAACATCTCCCCGCAGCCGAGTAAAGACCAAGAGACCAGCAGCGGCCCCTGGGGGAAAGGAGATCCA

CAGTGGGCCTAGGAGACCAGCGGCAGCCCCTGGGGGACAGGAGCTCCAGAGAAGGCCCCGAAGACCAGCGGTGGCCCC

TGAGGAACAGGAGCTACCGAGCAGGCCCAGGAGACCAGCGGTGGCCCCTGGGGCACAGGAGCTCCAGAGTGGGCCTCA

GAGACCAGCAGCGGCCCCAGGGGGACAGAAGATCCAGAATAGACCCCGGAGACCATCAGCAGCCCCTAAAGGACAGGA

GGTCCAGAGCAGGCCCAGCAGACCAGCGACAGCTCCTAGGGGACAGGAGCTCCAGAGCGCACCCCAGCGGCCAGTAGT

GGCCCCTGGGGGACGGGAGATTCATAGCAGGCCCTGGTTGCCAGCAGCAACCCCTGGGGGACAGGAGATCCAGAATGG

GACCCAGAAACACAGAGCCATGCTGCTGCTGTTTTCAGAGCAGCAGGACGGGGCCAGTCTCCTGCACACTGCCGGGGC

Furin cleavage site PAM 1

CTCGAAGTTTCTGTTTTCTAGAAATAAGAAAGAAGTCAAGCGAGGACGGGCCCTCAGGAGCCGCAGGGGCCGCCGGGG

Dendra2

GCCAAACACCCCCGGCATCAACCTGATCAAGGAAGATATGCGCGTCAAGGTGCACATGGAGGGCAACGTGAACGGCCA

CGCCTTCGTGATCGAGGGCGAGGGCAAGGGAAAGCCCTACGAGGGCACCCAGACCGCCAACCTGACCGTGAAGGAGGG

AGCCCCGCTGCCCTTCAGCTACGATATCCTGACCACCGCCGTGCACTACGGCAACCGCGTGTTCACCAAGTACCCCGA

GGATATCCCCGATTACTTCAAGCAGAGCTTCCCCGAGGGCTACAGCTGGGAGCGCACCATGACCTTCGAGGATAAGGG

CATCTGCACCATCCGCAGCGATATCAGCCTGGAGGGCGATTGCTTCTTCCAGAACGTGCGCTTCAAGGGCACCAACTT

CCCCCCCAACGGCCCCGTGATGCAGAAAAAGACCCTGAAGTGGGAGCCCAGCACCGAGAAGCTGCACGTGCGCGACGG

CCTGCTGGTCGGCAACATCAACATGGCCCTGCTGCTGGAGGGCGGAGGACACTACCTGTGCGATTTCAAGACCACCTA

CAAGGCCAAGAAGGTGGTCCAGCTGCCCGATGCCCACTTCGTGGATCACCGCATCGAGATCCTGGGCAACGATAGCGA

TTACAACAAGGTGAAGCTGTACGAGCACGCCGTGGCCCGCTACAGCCCCCTGCCCAGCCAGGTGTGGAAGCGGCGGGG

Linker V5

CAAGCCGATCCCGAATCCACTGCTCGGACTGGATAGCACCGGCGGAGGTTCCGGTGGAAGCGGAGGTAGCGGCGGACC

In-frame silent mutation

A (P*) GTG (V*) CGC (R*) AGG (S*) CCT (P*) GAA (E*) CTC (L*) CAG (Q*) AGG (R*) ACC (T*) CCT (P*) CTC

PAM 2

(L*) CAT (H*) AAA (K*) TCC (S*) ACA (T*) ACA (T*) TGCAGGAGGGTGGACATGCATGTGGATTTTAACCAGATC

ndr 2rd exon sequence

GGATGGGGCTCCTGGATCGTGTTCCCTAAGAAGTACAATGCATACCGGTGCGAGGGGGCCTGCCCGAACCCACTGGGA

GAAGAGCTGCGGCCCACCAACCATGCATACATGCAGGTACTACGGTATATGTGTAAATATAACTTTAAATACATGGAT

AAATGTGTAAATGTTAATTATTTTAAATTAATCTGGCTTTCAATAAAATATAAGCTAATTAGCATTTTTAAAAGTAGA

TTTAAATAGTTTAAAATATTTATATTAAATATTTTGTAATGGCATAAAAATAGGTAGATAAGAATATAAATAATAATT

GTTTGAAATAAAAGTTGCTTTAATTTAAATTTATATTAATTGGCATTTTTAATTGATGTGAAAAATTAAAGCTCAAAA

ATATTTATAATAAAACAACATAAATAAATGTAGAAATATTAAATAAAATGTCATTTGAAATAAAACTAAAACTATAAA

ACTAAAACTAATACAAGATTGTGTTACTTTAATGTTATATAATTTATAGATATGCATACATTTATAAAATAAAAATTT

ATATGAAATAATTAAAAAATAAATAATATCTATTTATATATTTTTTATTTACTGAAATAACAACTTTGCATTTAATAA

ATCACATCAAATGCTCTGCAAAACATTGATTTTACTCCTGAAGTAAATGCAAAGTTAATGTGAAACTTTACTGAAATA

ATGAAATAACTCTGCTGTGTGTGTGTATGTGTGTGTGTGTGTGTGTGCTTGTGCTGCAGAGTTTGCTGAAATATCATC

3' arm *Sac* II

ACCCCAGTCGTGTTCCTGCATCCTGCTGCGCTCCGACCCGCACCAGCGCTCTCAGTATGCTGTACTACGAGACCGCGG

TGGAGCTCCAGCTTTTGTTCCCTTTAGTGAGGGTTAATTGCGCGCTTGGCGTAATCATGGTCATAGCTGTTTCCTGTG

Plasmid backbone

TGAAATTGTTATCCGCTCACAAT......

Supplementary Text S3. Sequence of zebrafish *ndr2* targeting construct *linker-Dendra2*. The donor backbone is indicated with black lines, homology arms with purple lines, and *linker-Dendra2* with red lines. The exon sequences of *gfap* gene are in bold and highlighted. The mutated sequences of the sgRNA2 targeting sites are marked in green. The plasmid backbone sequences are the same as Gfap-p2A-ChR2-EYFP targeting donor showed in **Supplementary Text S1**.

Supplementary Text S4

Plasmid backbone

5’ctaaattgtaagcgttaatattttgttaaaattcgcgttaaatttttgttaaatcagctcattttttaaccaataggccgaaatcggcaaaatcccttataaatcaaaagaatagac
cgagatagggttgagtgttgttccagtttggaacaagagtccactattaaagaacgtggactccaacgtcaaagggcgaaaaaccgtctatcagggcgatggcccactacg
tgaaccatcaccctaatcaagttttttggggtcgaggtgccgtaaagcactaaatcggaaccctaaagggagcccccgatttagagcttgacggggaaagccggcgaacg
tggcgagaaaggaagggaagaaagcgaaaggagcgggcgctagggcgctggcaagtgtagcggtcacgctgcgcgtaaccaccacacccgccgcgcttaatgcgc
cgctacagggcgcgtcccattcgccattcaggctgcgcaactgttgggaagggcgatcggtgcgggcctcttcgctattacgccagctggcgaaagggggatgtgctgc
aaggcgattaagttgggtaacgccagggttttcccagtcacgacgttgtaaaacgacggccagtgagcgcgcgtaatacgactcactatagggcgaattgggtaccAAG

3’ arm

CGAGCTCTTTGGTCTGAGAGCGTTTTATTTTAGGGCATTGAGTCGTAGGAGTAGCTGTTTCTTCGCTGATTGCAACCT
TTATTCAATAAAGACTTGGAAACAAGAGAATGTTGTCACTCTACAGCCACACAAACAGCCTTGTGCTTACAGCTGGCC
TTTCATTTTAACACCTGGTCCTGGTACCTTATAATATCCAGCGCAGGCCAGACCAGCAAATTGCTGAGTGGAAAATCC
CCCCCCAAAAAAGACTTGTTAGATGTTTCTAACAAACTCTTTTCTTCTGTCCTTCCAGAGGCTTTCGAGGCACTGTCC

5’ arm

AGCCTGAAAGGAAAAACAACGCAGCAGTTTTTCTTCAACCTTACCTCCATTCCTGGCGAGGAGCTGATCTCCGCTGCG
GAGCTGCGCATTTTCAGGGACCAAGTTCTCGGAGATGCCAGTACGAGTGGCTTCCACAGAATTAACATTTACGAGGTG
TTCAGGCCAGCTTTGGCCCCCTCCAAAGAGCCTCTAACCAGACTTCTGGACACCCGTCTGGTGCAGGACTCTCACACG
CGCTGGGAAAGCTTCGACGTGGGTTCAGCTGTGGCACGCTGGGCCCGCGAATCCCAGCACAACCATGGGCTCCTTGTA
GAGGTGCTCCATCCTAAGGAGTCAGAAGTATCCGAGGAGGCTGAGAGCAACCGGAGGAAGCACGTGAGGGTCAGTCGT
TCCCTTCACGCGGATGAGGACTCGTGGGCACAAGCCCGACCTCTGCTGGTAACCTACAGCCATGACGGTCAAGGCACA

sg1 with in-frame mutation **Linker**

GCCGTCTTGCATTCGAACCGAGAgAAaCGcCAaGCaGGCGGAGGTTCCGGTGGAAGCGGAGGTAGCGGCGGAAACACC

Dendra2

CCCGGCATCAACCTGATCAAGGAAGATATGCGCGTCAAGGTGCACATGGAGGGCAACGTGAACGGCCACGCCTTCGTG
ATCGAGGGCGAGGGCAAGGGAAAGCCCTACGAGGGCACCCAGACCGCCAACCTGACCGTGAAGGAGGGAGCCCCGCTG
CCCTTCAGCTACGATATCCTGACCACCGCCGTGCACTACGGCAACCGCGTGTTCACCAAGTACCCCGAGGATATCCCC
GATTACTTCAAGCAGAGCTTCCCCGAGGGCTACAGCTGGGAGCGCACCATGACCTTCGAGGATAAGGGCATCTGCACC
ATCCGCAGCGATATCAGCCTGGAGGGCGATTGCTTCTTCCAGAACGTGCGCTTCAAGGGCACCAACTTCCCCCCCAAC
GGCCCCGTGATGCAGAAAAAGACCCTGAAGTGGGAGCCCAGCACCGAGAAGCTGCACGTGCGCGACGGCCTGCTGGTC
GGCAACATCAACATGGCCCTGCTGCTGGAGGGCGGAGGACACTACCTGTGCGATTTCAAGACCACCTACAAGGCCAAG
AAGGTGGTCCAGCTGCCCGATGCCCACTTCGTGGATCACCGCATCGAGATCCTGGGCAACGATAGCGATTACAACAAG

Linker

GTGAAGCTGTACGAGCACGCCGTGGCCCGCTACAGCCCCCTGCCCAGCCAGGTGTGGGGCAAGCCGATCCCGAATCCA

V5

CTGCTCGGACTGGATAGCACCGGCGGAGGTTCCGGTGGAAGCGGAGGTAGCGGCGGACGACGAGGGCAAAAGCCGAGG

PAM 2 with mutation

AGAAAGCACCACCAGCGCTCGAACTGTAGGCGACATGCTCTCTATGTcGACTTCAGTGATGTCGGCTGGAACGAGTGG
ATCGTGGCACCGCCAGGCTATCATGCTTTCTACTGCCATGGCGAGTGTCCGTTCCCTCTGCCGGACCATCTAAACTCC
ACCAACCATGCCATTGTCCAGACGCTGGTGAACTCGGTCAACTCCAACATTCCCAAAGCCTGTTGCATCCCGACGGAG
CTCAGCCCTATCTCACTGCTGTACCTGGACGAGTACGAGAAGGTCATTCTTAAAAACTACCAGGACATGGTGGTGGAG
GGCTGCGGTTGCCGATGAGAACAATCTCCCCAATGAAGACTTTTATTTATACAAAAGAGCGAGCTATTTTGGAGGAAG
AAAAGAAATATATATGAATATATTTATGTTGAATGAACAAAACAAAAAAAAATGGGAAAATAAATATTTTAATGAGAGT
GCTTGCTTTTTCCAGTGGCTTCGAAGATGATTTTTTTTCTTGCCCCATTCCAAAACAAGTGCAATGGCACATGAAGTA
TAATGCTCAGATTTTTATGATGTATTTATTGCATAACCACTTTATTTGTAAATGTGTATTTATCATGAAAAAATATAT
GATCTTCATTCAGTGTGCATTTGGGAATACCTTATTTGATACCAAAAAAATAAACAATGGATGATGAAGTTCTCTTTA
AAAAGTCATGATATAACATGCTGAAGGCATATTAAAAATCTATGCATccgcggtggagctccagcttttgttccctttagtgagggttaattgc
gcgcttggcgtaatcatggtcatagctgtttcctgtgtgaaattgttatccgctcacaattccacacaacatacgagccggaagcataaagtgtaaagcctggggtgcctaatg
agtgagctaactcacattaattgcgttgcgctcactgcccgctttccagtcgggaaacctgtcgtgccagctgcattaatgaatcggccaacgcgcggggagaggcggttt
gcgtattgggcgctcttccgcttcctcgctcactgactcgctgcgctcggtcgttcggctgcggcgagcggtatcagctcactcaaaggcggtaatacggttatccacagaa

tcaggggataacgcaggaaagaacatgtgagcaaaaggccagcaaaaggccaggaaccgtaaaaaggccgcgttgctggcgtttttccataggctccgcccccctgac
gagcatcacaaaaatcgacgctcaagtcagaggtggcgaaacccgacaggactataaagataccaggcgtttccccctggaagctccctcgtgcgctctcctgttccgac
cctgccgcttaccggatacctgtccgcctttctcccttcgggaagcgtggcgctttctcatagctcacgctgtaggtatctcagttcggtgtaggtcgttcgctccaagctggg
ctgtgtgcacgaaccccccgttcagcccgaccgctgcgccttatccggtaactatcgtcttgagtccaacccggtaagacacgacttatcgccactggcagcagccactgg
taacaggattagcagagcgaggtatgtaggcggtgctacagagttcttgaagtggtggcctaactacggctacactagaaggacagtatttggtatctgcgctctgctgaag
ccagttaccttcggaaaaagagttggtagctcttgatccggcaaacaaaccaccgctggtagcggtggtttttttgtttgcaagcagcagattacgcgcagaaaaaaaggat
ctcaagaagatcctttgatcttttctacggggtctgacgctcagtggaacgaaaactcacgttaagggattttggtcatgagattatcaaaaaggatcttcacctagatccttttta
aattaaaaatgaagttttaaatcaatctaaagtatatatgagtaaacttggtctgacagttaccaatgcttaatcagtgaggcacctatctcagcgatctgtctatttcgttcatccat
agttgcctgactccccgtcgtgtagataactacgatacgggagggcttaccatctggccccagtgctgcaatgataccgcgagacccacgctcaccggctccagatttatc
agcaataaaccagccagccggaagggccgagcgcagaagtggtcctgcaactttatccgcctccatccagtctattaattgttgccgggaagctagagtaagtagttcgcc
agttaatagtttgcgcaacgttgttgccattgctacaggcatcgtggtgtcacgctcgtcgtttggtatggcttcattcagctccggttcccaacgatcaaggcgagttacatgat
cccccatgttgtgcaaaaaagcggttagctccttcggtcctccgatcgttgtcagaagtaagttggccgcagtgttatcactcatggttatggcagcactgcataattctcttact
gtcatgccatccgtaagatgcttttctgtgactggtgagtactcaaccaagtcattctgagaatagtgtatgcggcgaccgagttgctcttgcccggcgtcaatacgggataat
accgcgccacatagcagaactttaaaagtgctcatcattggaaaacgttcttcggggcgaaaactctcaaggatcttaccgctgttgagatccagttcgatgtaacccactcg
tgcacccaactgatcttcagcatcttttactttcaccagcgtttctgggtgagcaaaaacaggaaggcaaaatgccgcaaaaaagggaataagggcgacacggaaatgttg
aatactcatactcttcctttttcaatattattgaagcatttatcagggttattgtctcatgagcggatacatatttgaatgtatttagaaaaataaacaaataggggttccgcgcacatt
Plasmid backbone
tccccgaaaagtgccac-3’

Supplementary Text S4. Sequence of zebrafish *bmp2b* targeting construct *linker-Dendra2-linker*.

Supplementary Text S5

Plasmid backbone

…… CCAGCTGGCGAAAGGGGGATGTGCTGCAAGGCGATTAAGTTGGGTAACGCCAGGGTTTTCCCAGTCACGACGT

***Kpn* I** **5' arm**

TGTAAAACGACGGCCAGTGAGCGCGCGTAATACGACTCACTATAGGGCGAATTGGGTACCGGTGCAATATTTATTTAA

***gfap* 8rd intron sequence**

AAAAGTATGTTTATTGTAAGATTTTTATGAACATCTATTAACATAAACAATGAACAATATATGTATTATATTACTTTA
TAATCTTTGTCAAATGTTAGTTGAAATACAGCTATTCATTATTTAAGTTAGCTTCAATCCATTAAATAATATCAACAT
ACAGCAAAATCCTCTGAGCAAAATTTACTCACTTCAGAGAGTAAATGTATCTCTTTAAATAAGGGCAAACAGGTTGGT
AATAATTAAAGCTGCTGGTTTTGGAGTTGCTTAATGATCCTCTGCTGAGATTTCAAAAGAGATTCCATGTACTATATT
TCAGCTGATTTCATCTAAGGATGTGACTGATTTCAGTTTTAGTTTTGTGTTTCTCTTCTGTGTTTCTGTTTATTAACA
ACAACAATCAGTGCTCAATCATCCCCCAATAAATCCTTTCATTTTTTCATAAACATGAGTAACTTTAACACTTTAAAA
AGAACCAAATACTGTCTAAACTGAGTAAAATTTACTCAGAGGATTTTGCTGTGTAACTTTTTTACTTAATGATTTAGT
TCATATTAACTAATTTACTCTATATTCCAGTCAATAAATTTGTATTCCTGTCTACAATGTAATATTCTCTTTCACTGT
AAAAGAATTCAGGATCCCACACAACTCAAATAAATTAACTTTTTTACTAATTTTTTTAGGGTTTTTTCACCTTTATTT
GGTGTAGGGCAGTGGAGGTTACAGACTGGATAGTATTGGGAGCAGAGAGGGGGGAAGGGTTGGCAAAGGACCTTAAGC
CGAGAATCGAACTCGGATCACCATAAGAACCATGGTGCTATGTGTTGCGCACTTAACCACTAGGCTATAGGCGCCGAC
CGATTAAGTTAACTTCAATTAAAGTGGATTAAACATAAAACAATTAAGTTGTCCCCAAAAACATCTCAAGAATTGTGT

PAM 1 ***gfap* 9rd exon sequence**

TGATTCTGCTCATTCTTCATTTTACACATTCTCTCTTTCGCAGATCATTAAAGAGTCCACTA^CC(T*).GAA(E*).

In-frame silent mutation **P2A**

CGT(R*).AAA(K*).GAC(D*).CTA(L*).CCG(P*).GGATCCGGAGCTACTAATTTCTCCTTGCTTAAGCAAGC

NpHR3.0

TGGTGATGTTGAAGAAAATCCTGGTCCTATGACAGAGACCCTGCCTCCCGTGACCGAGAGTGCCGTGGCCCTTCAAGC
CGAGGTTACCCAAAGGGAGTTGTTCGAGTTCGTGCTGAACGACCCTTTGCTTGCAAGCAGTCTCTATATCAACATCGC
ACTTGCAGGACTGAGTATACTGCTGTTCGTTTTTATGACCCGAGGACTCGATGATCCACGGGCAAAACTTATTGCTGT
GTCAACCATCCTTGTGCCTGTCGTCAGCATTGCCTCCTACACTGGATTGGCGAGCGGCCTGACAATTTCCGTTCTTGA
AATGCCAGCGGGCCATTTTGCAGAAGGCAGCTCAGTGATGCTGGGAGGAGAAGAGGTAGATGGTGTAGTCACCATGTG
GGGACGGTATCTCACCTGGGCACTTTCCACGCCCATGATTCTCCTCGCTCTGGGTCTCCTGGCCGGAAGCAATGCTAC
AAAGCTCTTCACAGCTATCACTTTCGATATCGCTATGTGCGTGACTGGCCTTGCCGCGGCCCTGACTACCTCCTCCCA
CCTCATGAGATGGTTCTGGTACGCTATCAGTTGTGCATGCTTTCTGGTGGTCTTGTATATCCTGCTGGTGGAGTGGGC
ACAGGACGCCAAAGCCGCGGGAACCGCTGACATGTTCAATACCCTGAAGCTGTTGACAGTAGTGATGTGGCTGGGGTA
TCCAATTGTGTGGGCTCTTGGAGTCGAGGGTATCGCGGTGTTGCCCGTTGGGGTGACGAGCTGGGGATATTCTTTCCT
GGATATCGTGGCAAAGTACATTTTCGCATTCTTGCTCCTGAACTATCTGACGTCAAACGAATCTGTCGTGTCCGGCAG

TS

CATTTTGGATGTTCCATCTGCTTCTGGGACCCCGGCTGATGATGCGGCCGCCAAGAGCAGGATCACCAGCGAGGGCGA

EYFP

GTACATCCCCCTGGACCAGATCGACATCAACGTGGTGAGCAAGGGCGAGGAGCTGTTCACCGGGGTGGTGCCCATCCT
GGTCGAGCTGGACGGCGACGTAAACGGCCACAAGTTCAGCGTGTCCGGCGAGGGCGAGGGCGATGCCACCTACGGCAA
GCTGACCCTGAAGTTCATCTGCACCACCGGCAAGCTGCCCGTGCCCTGGCCCACCCTCGTGACCACCTTCGGCTACGG
CCTGCAGTGCTTCGCCCGCTACCCCGACCACATGAAGCAGCACGACTTCTTCAAGTCCGCCATGCCCGAAGGCTACGT
CCAGGAGCGCACCATCTTCTTCAAGGACGACGGCAACTACAAGACCCGCGCCGAGGTGAAGTTCGAGGGCGACACCCT
GGTGAACCGCATCGAGCTGAAGGGCATCGACTTCAAGGAGGACGGCAACATCCTGGGGCACAAGCTGGAGTACAACTA
CAACAGCCACAACGTCTATATCATGGCCGACAAGCAGAAGAACGGCATCAAGGTGAACTTCAAGATCCGCCACAACAT
CGAGGACGGCAGCGTGCAGCTCGCCGACCACTACCAGCAGAACACCCCCATCGGCGACGGCCCCGTGCTGCTGCCCGA
CAACCACTACCTGAGCTACCAGTCCGCCCTGAGCAAAGACCCCAACGAGAAGCGCGATCACATGGTCCTGCTGGAGTT

P2A

CGTGACCGCCGCCGGGATCACTCTCGGCATGGACGAGCTGTACAAGTTCTGCTACGAGAACGAGGTGGGCTCCGGAGC

hChR2

CACGAACTTCTCTCTGTTAAAGCAAGCAGGAGACGTGGAAGAAAACCCCGGTCCCATGGACTATGGCGGCGCTTTGTC
TGCCGTCGGACGCGAACTTTTGTTCGTTACTAATCCTGTGGTGGTGAACGGGTCCGTCCTGGTCCCTGAGGATCAATG

TTACTGTGCCGGATGGATTGAATCTCGCGGCACGAACGGCGCTCAGACCGCGTCAAATGTCCTGCAGTGGCTTGCAGC
AGGATTCAGCATTTTGCTGCTGATGTTCTATGCCTACCAAACCTGGAAATCTACATGCGGCTGGGAGGAGATCTATGT
GTGCGCCATTGAAATGGTTAAGGTGATTCTCGAGTTCTTTTTTGAGTTTAAGAATCCCTCTATGCTCTACCTTGCCAC
AGGACACCGGGTGCAGTGGCTGCGCTATGCAGAGTGGCTGCTCACTTGTCCTGTCATCCTTATCCGCCTGAGCAACCT
CACCGGCCTGAGCAACGACTACAGCAGGAGAACCATGGGACTCCTTGTCTCAGACATCGGGACTATCGTGTGGGGGGC
TACCAGCGCCATGGCAACCGGCTATGTTAAAGTCATCTTCTTTTGTCTTGGATTGTGCTATGGCGCGAACACATTTTT
TCACGCCGCCAAAGCATATATCGAGGGTTATCATACTGTGCCAAAGGGTCGGTGCCGCCAGGTCGTGACCGGCATGGC
ATGGCTGTTTTTCGTGAGCTGGGGTATGTTCCCAATTCTCTTCATTTTGGGGCCCGAAGGTTTTGGCGTCCTGAGCGT
CTATGGCTCCACCGTAGGTCACACGATTATTGATCTGATGAGTAAAAATTGTTGGGGGTTGTTGGGACACTACCTGCG
CGTCCTGATCCACGAGCACATATTGATTCACGGAGATATCCGCAAAACCACCAAACTGAACATCGGCGGAACGGAGAT
CGAGGTCGAGACTCTCGTCGAAGACGAAGCCGAGGCCGGAGCCGTGCCAGCGGCCGCCGTGAGCAAGGGCGAGGAGGA
mcherry
TAACATGGCCATCATCAAGGAGTTCATGCGCTTCAAGGTGCACATGGAGGGCTCCGTGAACGGCCACGAGTTCGAGAT
CGAGGGCGAGGGCGAGGGCCGCCCCTACGAGGGCACCCAGACCGCCAAGCTGAAGGTGACCAAGGGTGGCCCCCTGCC
CTTCGCCTGGGACATCCTGTCCCCTCAGTTCATGTACGGCTCCAAGGCCTACGTGAAGCACCCCGCCGACATCCCCGA
CTACTTGAAGCTGTCCTTCCCCGAGGGCTTCAAGTGGGAGCGCGTGATGAACTTCGAGGACGGCGGCGTGGTGACCGT
GACCCAGGACTCCTCCCTGCAGGACGGCGAGTTCATCTACAAGGTGAAGCTGCGCGGCACCAACTTCCCCTCCGACGG
CCCCGTAATGCAGAAGAAGACCATGGGCTGGGAGGCCTCCTCCGAGCGGATGTACCCCGAGGACGGCGCCCTGAAGGG
CGAGATCAAGCAGAGGCTGAAGCTGAAGGACGGCGGCCACTACGACGCTGAGGTCAAGACCACCTACAAGGCCAAGAA
GCCCGTGCAGCTGCCCGGCGCCTACAACGTCAACATCAAGTTGGACATCACCTCCCACAACGAGGACTACACCATCGT
GGAACAGTACGAACGCGCCGAGGGCCGCCACTCCACCGGCGGCATGGACGAGCTGTACAAGTAATGAGAATTCGCGCC
CCCCCCTAACGTTACTGGCCGAAGCCGCTTGGAATAAGGCCGGTGTGCGTTTGTCTATATGTTATTTTCCACCATATT
IRES
GCCGTCTTTTGGCAATGTGAGGGCCCGGAAACCTGGCCCTGTCTTCTTGACGAGCATTCCTAGGGGTCTTTCCCCTCT
CGCCAAAGGAATGCAAGGTCTGTTGAATGTCGTGAAGGAAGCAGTTCCTCTGGAAGCTTCTTGAAGACAAACAACGTC
TGTAGCGACCCTTTGCAGGCAGCGGAACCCCCCACCTGGCGACAGGTGCCTCTGCGGCCAAAAGCCACGTGTATAAGA
TACACCTGCAAAGGCGGCACAACCCCAGTGCCACGTTGTGAGTTGGATAGTTGTGGAAAGAGTCAAATGGCTCTCCTC
AAGCGTATTCAACAAGGGGCTGAAGGATGCCCAGAAGGTACCCCATTGTATGGGATCTGATCTGGGGCCTCGGTGCAC
ATGCTTTACATGTGTTTAGTCGAGGTTAAAAAACGTCTAGGCCCCCCGAACCACGGGGACGTGGTTTTCCTTTGAAAA
ACACGATGATAATATGGCCACAACCATGAGGAAGATGATGTCTACAATGGCATTGACCCTGGGAGCTGCCGTGTTTCT
WGA
CGCGTTCGCCGCCGCTACGGCACAGGCCCAGCGATGCGGGGAACAAGGGAGCAATATGGAGTGTCCCAATAATCTTTG
TTGCAGTCAGTATGGCTACTGCGGGATGGGTGGTGACTACTGTGGCAAGGGATGTCAGAACGGCGCTTGCTGGACATC
TAAACGCTGTGGCTCTCAGGCCGGAGGAGCTACCTGCCCAAACAACCATTGCTGCTCACAGTATGGTCACTGTGGCTT
TGGAGCAGAATATTGCGGTGCTGGCTGTCAGGGCGGGCCGTGTAGAGCAGACATCAAGTGTGGAAGCCAATCAGGTGG
GAAACTGTGTCCTAACAATCTGTGCTGCTCCCAGTGGGGTTTCTGTGGCCTGGGGAGCGAGTTCTGCGGTGGGGGCTG
CCAGAGTGGGGCCTGTAGCACCGATAAACCATGCGGAAAGGACGCCGGAGGGCGGGTCTGCACTAACAATTACTGCTG
TTCCAAATGGGGTTCCTGCGGGATTGGCCCCGGATATTGCGGCGCTGGATGCCAAAGTGGCGGTTGTGATATGCCCAA
GAAGAAGAGGAAGGTGTCCAATCTCCTGACTGTTCACCAGAACCTCCCTGCGCTGCCAGTAGATGCCACTAGCGATGA
Cre
GGTCAGGAAAAATCTCATGGATATGTTTAGGGATAGACAGGCGTTTTCTGAACACACCTGGAAAATGCTGCTTAGCGT
GTGCCGATCCTGGGCAGCCTGGTGTAAGCTGAACAATCGCAAATGGTTCCCCGCCGAGCCGGAGGACGTGCGCGATTA
CCTGCTGTATCTCCAGGCAAGAGGGCTGGCTGTCAAGACTATCCAGCAGCACTTGGGCCAACTGAATATGCTGCATCG
ACGCAGCGGGCTCCCCCGGCCTAGCGATTCAAACGCAGTCTCCCTTGTTATGAGGAGAATTAGAAAGGAAAACGTAGA
TGCGGGTGAGAGGGCTAAGCAGGCTCTCGCTTTTGAGCGGACTGATTTCGACCAGGTCAGATCCCTGATGGAGAACAG

CGATCGGTGCCAGGACATCAGGAACCTCGCATTTCTGGGAATTGCATATAACACACTTCTGCGCATAGCTGAGATCGC

CCGGATCAGAGTGAAAGACATCAGTCGAACGGACGGCGGCCGGATGCTTATTCATATTGGACGCACAAAGACATTGGT

CAGCACCGCTGGCGTTGAAAAGGCCTTGTCCCTGGGCGTAACGAAGCTGGTGGAAAGATGGATCTCAGTGTCCGGCGT

GGCTGACGACCCTAATAATTACTTGTTCTGTCGAGTGAGAAAAAACGGAGTCGCCGCGCCCTCTGCCACCAGCCAATT

GAGTACACGGGCCCTTGAAGGGATCTTTGAGGCAACCCACCGACTCATATACGGAGCCAAGGATGACAGTGGCCAGAG

GTATCTCGCCTGGTCAGGTCATTCTGCTAGGGTGGGGGCCGCACGAGACATGGCGCGGCAGGAGTCTCCATACCAGA

GATTATGCAAGCTGGAGGTTGGACAAATGTGAACATCGTTATGAACTATATCCGCAATCTTGACTCTGAAACCGGGGC

Stop codon **sgRNA2 target Mutation**

CATGGTGAGACTGCTCGAAGATGGTGAC**TG**TGAGGCTCAGACACTGGCTTGGCTGCAGAAGAAGATCTCCTTGTGATT

PAM 2

ACGTTCAGGAAGCTGTGTTTGTCAAACCTTGTATTTTCTCACCAACTTTCCCTGCTTCTCTCCTGGTATATATAGTTC

ATGGAAACTGTATTCACTGTTTTTAGCAACTACCTATATGTTTTGATCTCCTTTTCTTCCTGGGCTTCTCTTTTTATC

TGTCTGTTTCTTCTGTGGCAGAGTCCTTGCAAGATAGTAGACTAGTTTATCGCAGAATGTGCTATAAAAAGTCATGGT

TGGACAGATGAAAGTAAACAGTAGGACAAGATGTAGCTAGTGGTCAGAGCGGGTGGGAAATATAGTGTTTTTTTTTAT

CAAGCATAAATTGTAGAGAACAAGTTCTTGAGCTTTATTGTAGTCTTTAAGAATGCCAGAAGAAAAAAAGAGCAAGTC

TGGAGCTGGTAGTTTTTAAATCTGAACAGTAGGTGTTAAAGTAAGGCAGAAGGGAATTTTAACAGCAAGATGTTTGTA

AGAGTTTTAAAGGAATGATGGATTTGGTCAGCGGTGCTATTTGAACATTTTACTCCACATTTTTTTTGTCCTCCTCCA

CTGTGTGTGTGCGCAGTGTTTTTGCACAGTAGCTAACTCATCTCTTGGACCCAGGTTAAAATGCTTTAAATTGCTCGT

TGACACATTACTGACCTGTAGAAGACATTAAAATCTTAACATTAAGCATCTTACTGAATGAATTATGGTATAAATATC

TGACACAGTGTTCCCCATCATGTGACCAGATCATTTGATTCAGATTTATACTGATAGCTTTCAATTGTCACGCTACAT

TTCCTTGGTGAAATTGAGCAATTTTTCCATTTCCATCTGTGTGATCAGGCATTTTTGCATGAGGGTGAAAGGTTCCTC

3' arm

CTGCAGTTTATGCTTACATTTGAGTTGTTATTCTGAATTGCACAAATATCATCTTGGTTTGAAAGTGACTTAAGTGTG

AGCAGTGTTTCACACATCACTACAATGTTAATAATATGCATTAATAATAATCAAAACTGTGACCATATCTTCACCCGC

***Sac* II**

GGTGGAGCTCCAGCTTTTGTTCCCTTTAGTGAGGGTTAATTGCGCGCTTGGCGTAATCATGGTCATAGCTGTTTCCTG

Plasmid backbone

TGTGAAATTGTTATCCGCTCAC……

Supplementary Text S5. Sequence of zebrafish *gfap* targeting construct *p2AV1-NpHR3.0-EYFP-p2AV2-hChR2-mCherry-IRES-WGA-Cre*. The donor backbone is indicated with black lines, homology arms with purple lines, and insertion sequence with red lines. The exon sequences of *gfap* gene are in bold and highlighted, and the gRNA1 targeting sites were replaced by in-frame silent mutations. The mutated sequences of the sgRNA2 targeting sites are marked in green. The plasmid backbone sequences are the same as Gfap-p2A-ChR2-EYFP targeting donor showed in **Supplementary Text S1**.

Supplementary Text S6

5'...ctaaattgtaagcgttaatattttgttaaaattcgcgttaaatttttgttaaatcagctcattttttaaccaataggccgaaatcggcaaaatcccttataaatcaaaagaatag
Plasmid backbone
accgagatagggttgagtgttgttccagtttggaacaagagtccactattaaagaacgtggactccaacgtcaaagggcgaaaaaccgtctatcagggcgatggcccacta
cgtgaaccatcaccctaatcaagttttttggggtcgaggtgccgtaaagcactaaatcggaaccctaaagggagcccccgatttagagcttgacggggaaagccggcgaa
cgtggcgagaaaggaagggaagaaagcgaaaggagcgggcgctagggcgctggcaagtgtagcggtcacgctgcgcgtaaccaccacacccgccgcgcttaatgc
gccgctacagggcgcgtcccattcgccattcaggctgcgcaactgttgggaagggcgatcggtgcgggcctcttcgctattacgccagctggcgaaagggggatgtgct
gcaaggcgattaagttgggtaacgccagggttttcccagtcacgacgttgtaaaacgacggccagtgagcgcgcgtaatacgactcactatagggcgaattgggtaccA
5' arm
TGCTCTGTTCTTAGATTAGCTGTAGCTTATAGGTTAGATAGTCTCGTAGAAGGCTATGGCCTGGCAAAG
TCTGAGCTCAGTAGTCGTCTAGTGGGCTGGCAAAATGGTTCAGCAGCAAAGGCTGCAGGAGTAAAATT
GCTGGAACTTACACAGTAGAAAACTGACCCCTAAAAGTCACCTTCCGACCTCCACGTAGGCACCGCTCA
CATGCACCCACACATACGCACACCAGAATTAAGTACGATCTAAGCCTGGCAACTTGGGAGAATCCATG
TCTAAATGGGCAGCTGTGAGCATAGGAAGGAGGTGGGGCGTGAAAGCCATTTTCTTTCAATGTTATTTA
CTGTGCTCGCCTCTCCCACACACTGTGAGGAGTGAGCTCTTTCCTGTGCATCCTAACTCCACTTAAGACT
AATTCCAGGCCGGGCAGTGGTGGCACACGCCTTTAATCCCAGCACTTGGGAGGCAGAGGCAGGCGGAT
TTCTGAGTTTGAGGCCAGCCTGGTCTACAGAGTGAGTTCCAGGACAGCCAGGGCTACACAGAGAAACC
CTGTCTCAAAACGAACTAAAAAAAAAAAAAAAAGACCAATTCCAATTGTAAGGCATATTTCTATCTAATT
AATGATAGTTTATAGAGTGGTGAGGGATGGCTTTCGGTGGCTCTTGGCACTGGCGGGCACCATGGCTGA
GAGGTGGGATGTCTGCTCCCGGAGTGGCTCTGGGAAGCCTTGTCTGTTTAGTCAGTCATGCTGTGGCCA
GCAGGGTTCCGCCGTAGGACTGTTCCTTAAGTTCCTGTCACAACCACACTCTTCCTGCACCCGACGCTTG
TACTTCTCCACTCTGACATTTTCATCTGTATTAGAAGTTGGGAATTTCTTCTTTTAAAGGCTAGTGAGGC
TATTTCAACTTATTGTGAAATATCATTAAAATACTCTCAATGTTTTAATATTAATGTTGAAATGTCTTTTC
PAM1 CCC - CgC
AAACAAGACTTTGTAACACTGCACTTTTCTTCgCAACAGCGCATTATTAAAAGTATCACTCCTGAAACAC
PAM3 CCC - tCG p2A-chR2-EYFP
CAACGGAAATTCCGTGTGGGGACATtCGCATGAATGCTGTGGGATCCGGAGCTACTAATTTCTCCTTGCT
TAAGCAAGCTGGTGATGTTGAAGAAAATCCTGGTCCTATGGACTATGGCGGCGCTTTGTCTGCCGTCGG
ACGCGAACTTTTGTTCGTTACTAATCCTGTGGTGGTGAACGGGTCCGTCCTGGTCCCTGAGGATCAATGT
TACTGTGCCGGATGGATTGAATCTCGCGGCACGAACGGCGCTCAGACCGCGTCAAATGTCCTGCAGTGG
CTTGCAGCAGGATTCAGCATTTTGCTGCTGATGTTCTATGCCTACCAAACCTGGAAATCTACATGCGGCT
GGGAGGAGATCTATGTGTGCGCCATTGAAATGGTTAAGGTGATTCTCGAGTTCTTTTTTGAGTTTAAGA
ATCCCTCTATGCTCTACCTTGCCACAGGACACCGGGTGCAGTGGCTGCGCTATGCAGAGTGGCTGCTCA
CTTGTCCTGTCATCCTTATCCGCCTGAGCAACCTCACCGGCCTGAGCAACGACTACAGCAGGAGAACCA
TGGGACTCCTTGTCTCAGACATCGGGACTATCGTGTGGGGGGCTACCAGCGCCATGGCAACCGGCTATG
TTAAAGTCATCTTCTTTTGTCTTGGATTGTGCTATGGCGCGAACACATTTTTTCACGCCGCCAAAGCATA
TATCGAGGGTTATCATACTGTGCCAAAGGGTCGGTGCCGCCAGGTCGTGACCGGCATGGCATGGCTGTT
TTTCGTGAGCTGGGGTATGTTCCCAATTCTCTTCATTTTGGGGCCCGAAGGTTTTGGCGTCCTGAGCGTC
TATGGCTCCACCGTAGGTCACACGATTATTGATCTGATGAGTAAAAATTGTTGGGGGTTGTTGGGACAC
TACCTGCGCGTCCTGATCCACGAGCACATATTGATTCACGGAGATATCCGCAAAACCACCAAACTGAAC
ATCGGCGGAACGGAGATCGAGGTCGAGACTCTCGTCGAAGACGAAGCCGAGGCCGGAGCCGTGCCAG
CGGCCGCCACCATGGTGAGCAAGGGCGAGGAGCTGTTCACCGGGGTGGTGCCCATCCTGGTCGAGCTG
GACGGCGACGTAAACGGCCACAAGTTCAGCGTGTCCGGCGAGGGCGAGGGCGATGCCACCTACGGCAA
GCTGACCCTGAAGTTCATCTGCACCACCGGCAAGCTGCCCGTGCCCTGGCCCACCCTCGTGACCACCTT
CGGCTACGGCCTGCAGTGCTTCGCCCGCTACCCCGACCACATGAAGCAGCACGACTTCTTCAAGTCCGC

CATGCCCGAAGGCTACGTCCAGGAGCGCACCATCTTCTTCAAGGACGACGGCAACTACAAGACCCGCG
CCGAGGTGAAGTTCGAGGGCGACACCCTGGTGAACCGCATCGAGCTGAAGGGCATCGACTTCAAGGAG
GACGGCAACATCCTGGGGCACAAGCTGGAGTACAACTACAACAGCCACAACGTCTATATCATGGCCGA
CAAGCAGAAGAACGGCATCAAGGTGAACTTCAAGATCCGCCACAACATCGAGGACGGCAGCGTGCAG
CTCGCCGACCACTACCAGCAGAACACCCCCATCGGCGACGGCCCCGTGCTGCTGCCCGACAACCACTAC
CTGAGCTACCAGTCCGCCCTGAGCAAAGACCCCAACGAGAAGCGCGATCACATGGTCCTGCTGGAGTT
Stop PAM2
CGTGACCGCCGCCGGGATCACTCTCGGCATGGACGAGCTGTACAAGTAATAGCACACTGAGGGAGAGG
ACATGGCCTCCCAGCCCCCGACTCCTCATCTCTGAAAAGCCCCACCTTGACTCCTCCCCTCTAAGCCAA
GCTGATGATGTAAGGTCTTTCTCCATGGAGTCACAGTCCTAAAGACTATGGTGCCCAGACTCTTGTGGG
TTCCAACCACTTCTTTCCATGAACTCTCTTGGACTTACTGCCACATTAGCTGGTGACACGGCTGAGCTGA
CTTGGATATGTGAGGAGAGGAAGGAGGGGATGAACGCCACCCAGTCATCAGCTAGCTTCAGGTTTAGA
TTAGGTCTGTGAACGTCTGTATCATGTTCTGGGTATGATCATATTGCCCTGCATCTGTTTGCTTCTAAAG
CCTTCAGTGTTCATGAATACATAAACCACCTAAGAGAAAACAGGGATGTCTTGCTAGCCATATATATTT
TCTCAGTAGCATAGAACTCTGTAGCCGGAATCTACTAGAACCCTGTAACCCACGTGCTGCTGTGAGGTT
AAGAAAGGAAGACGTAAAGACGCTACACTGAAAACTGATATATATGTGTGAGCTCTTGTGTCTGTCCAT
TGTTGTCTGTGTCCCCTCAATTCCAACACTCCGGGCCCATTACAAACTATATAAATGGCCTCTAATTTTT
CTTACATTAAACAGATTCTACCTACTCAATTGGGTTTGTGTTATTAGAAGCATGCTGGGTTACCATCTCC
TTTCCCGGTGTCTCTTCCACCACCCACCTCCCTGTTAGGCATTCAGGATAAAGGTCCTTGGGCAAACCTG
GCCTGTTCTATCAGTCACTATGGCCACTGCTTTCCAGATGGTTGTAGCCAGATGCAACTTCCACGTGCAG
TTGGGTATCTGGTTTAAATGGCTGAGGGTTTGACTATTCAGGTTATTGGTCCTGTGGAGCCAGGGCAAA
CCTGACTTCTACTTAGCTCTGGGGTACATAGGTCTAATTCTATGAGCTAAAAGGCAGATCTTCTCCACCC
3’ arm Plasmid backbone
GGGAGCAATCGCAGAATCGGACTccgcggtggagctccagcttttgttccctttagtgagggttaattgcgcgcttggcgtaatcatggtcatagctgtt
tcctgtgtgaaattgttatccgctcacaattccacacaacatacgagccggaagcataaagtgtaaagcctggggtgcctaatgagtgagctaactcacattaattgcgttgc
gctcactgcccgctttccagtcgggaaacctgtcgtgccagctgcattaatgaatcggccaacgcgcggggagaggcggtttgcgtattgggcgctcttccgcttcctcgct
cactgactcgctgcgctcggtcgttcggctgcggcgagcggtatcagctcactcaaaggcggtaatacggttatccacagaatcaggggataacgcaggaaagaacatgt
gagcaaaaggccagcaaaaggccaggaaccgtaaaaaggccgcgttgctggcgtttttccataggctccgcccccctgacgagcatcacaaaaatcgacgctcaagtc
agaggtggcgaaacccgacaggactataaagataccaggcgtttccccctggaagctccctcgtgcgctctcctgttccgaccctgccgcttaccggatacctgtccgcct
ttctcccttcgggaagcgtggcgctttctcatagctcacgctgtaggtatctcagttcggtgtaggtcgttcgctccaagctgggctgtgtgcacgaaccccccgttcagccc
gaccgctgcgccttatccggtaactatcgtcttgagtccaacccggtaagacacgacttatcgccactggcagcagccactggtaacaggattagcagagcgaggtatgta
ggcggtgctacagagttcttgaagtggtggcctaactacggctacactagaaggacagtatttggtatctgcgctctgctgaagccagttaccttcggaaaaagagttggta
gctcttgatccggcaaacaaaccaccgctggtagcggtggtttttttgtttgcaagcagcagattacgcgcagaaaaaaaggatctcaagaagatcctttgatcttttctacgg
ggtctgacgctcagtggaacgaaaactcacgttaagggattttggtcatgagattatcaaaaaggatcttcacctagatccttttaaattaaaaatgaagttttaaatcaatctaa
agtatatatgagtaaacttggtctgacagttaccaatgcttaatcagtgaggcacctatctcagcgatctgtctatttcgttcatccatagttgcctgactccccgtcgtgtagata
actacgatacgggagggcttaccatctggccccagtgctgcaatgataccgcgagacccacgctcaccggctccagatttatcagcaataaaccagccagccggaaggg
ccgagcgcagaagtggtcctgcaactttatccgcctccatccagtctattaattgttgccgggaagctagagtaagtagttcgccagttaatagtttgcgcaacgttgttgccat
tgctacaggcatcgtggtgtcacgctcgtcgtttggtatggcttcattcagctccggttcccaacgatcaaggcgagttacatgatcccccatgttgtgcaaaaaagcggttag
ctccttcggtcctccgatcgttgtcagaagtaagttggccgcagtgttatcactcatggttatggcagcactgcataattctcttactgtcatgccatccgtaagatgcttttctgtg
actggtgagtactcaaccaagtcattctgagaatagtgtatgcggcgaccgagttgctcttgcccggcgtcaatacgggataataccgcgccacatagcagaactttaaaag
tgctcatcattggaaaacgttcttcggggcgaaaactctcaaggatcttaccgctgttgagatccagttcgatgtaacccactcgtgcacccaactgatcttcagcatcttttact
ttcaccagcgtttctgggtgagcaaaaacaggaaggcaaaatgccgcaaaaaagggaataagggcgacacggaaatgttgaatactcatactcttcctttttcaatattattg
aagcatttatcagggttattgtctcatgagcggatacatatttgaatgtatttagaaaaataaacaaataggggttccgcgcacatttccccgaaaagtgccac-3’

Supplementary Text S6. Sequence of mouse Slc6a4-p2A-ChR2-EYFP donor plasmid.

Supplementary Text S7

5’...ctaaattgtaagcgttaatattttgttaaaattcgcgttaaatttttgttaaatcagctcattttttaaccaataggccgaaatcggcaaaatcccttataaatcaaaagaatag
Plasmid backbone
accgagatagggttgagtgttgttccagtttggaacaagagtccactattaaagaacgtggactccaacgtcaaagggcgaaaaaccgtctatcagggcgatggcccacta
cgtgaaccatcaccctaatcaagttttttggggtcgaggtgccgtaaagcactaaatcggaaccctaaagggagcccccgatttagagcttgacggggaaagccggcgaa
cgtggcgagaaaggaagggaagaaagcgaaaggagcgggcgctagggcgctggcaagtgtagcggtcacgctgcgcgtaaccaccacacccgccgcgcttaatgc
gccgctacagggcgcgtcccattcgccattcaggctgcgcaactgttgggaagggcgatcggtgcgggcctcttcgctattacgccagctggcgaaagggggatgtgct
gcaaggcgattaagttgggtaacgccagggttttcccagtcacgacgttgtaaaacgacggccagtgagcgcgcgtaatacgactcactatagggcgaattggCTCC
5’ arm
CCCAACACCAGGACGTTTGGCAAAGCTGCAAGACTTTTTTTTTTTTTTTTTTTTTAATTGTGCTTCCAG
TAAAATAGGGAGTTGCTAAAGTCATAGCAAGAGATTTGCAGCTATCCCTCACGGGACCTGAAAGGTTCT
CGGTAAAGTCCCTTAAAAATAGGAGGTGCTTGGGAAATGTGCTTTGCTTTGGGTGTGTCTGAAGCCTCA
TTAAATCTTAGGTAAGAATTGGCAAGGATACCATATCCTGGTACATGGTAATTTTCTCACCTGTGCCCTA
ACCCTGTTCTGCCTTTCTGGGAGAAGGGAAGATGGTGTCTGGATCTGATTCTTACTTTCTTCCCTTTCCA
ACTTGGTATTTGGATAGCATCGGTCAAATCCTATGTATAGCGTCCGGGATTCAGGAGGCGTGGCTAACT
GTGATCTTCCACTTCCTCCCTTACAGAAGAAGAGCAAGAAGATGAGGAAGAAATTGATGTGGTGTCTGT
GGAGAAGAGGCAAACCCCTGCCAAGAGGTCGGAGTCGGGCTCATCTCCATCCCGAGGCCACAGCAAAC
CTCCGCACAGCCCACTGGTCCTCAAGAGGTGCCACGTCTCCACTCACCAGCACAACTACGCCGCACCCC
CCTCCACAAGGAAGGACTATCCAGCTGCCAAGAGGGCCAAGTTGGACAGTGGCAGGGTCCTGAAGCAG
ATCAGCAACAACCGCAAGTGCTCCAGCCCCAGGTCCTCAGACACGGAGGAAAACGACAAGAGGCGGA
CACACAACGTCTTGGAACGTCAGAGGAGGAACGAGCTGAAGCGCAGCTTTTTTGCCCTGCGTGACCAG
ATCCCTGAATTGGAAAACAACGAAAAGGCCCCCAAGGTAGTGATCCTCAAAAAAGCCACCGCCTACAT
PAM1
CCTGTCCATTCAAGCAGACGAGCACAAGCTCACCTCTGAAAAGGACTTATTGAGGAAACGACGAGAAC
PAM2 TGG - atG Stop linker-sfGFP
AGTTGAAACACAAACTCGAACAGCTTCGAAACTCatGTGCA~~(TAA)~~GGCGGAGGTTCCGGTGGAAGCGGA
GGTAGCGGCGGAATGCGTAAAGGCGAGGAGCTGTTCACCGGCGTGGTGCCCATCCTGGTGGAGCTGGA
TGGCGACGTGAACGGCCACAAGTTCAGCGTGCGCGGCGAGGGCGAGGGCGACGCCACCAACGGCAAG
CTGACCCTGAAGTTCATCTGCACCACCGGCAAGCTGCCCGTGCCCTGGCCCACCCTGGTGACCACCCTG
ACCTACGGCGTGCAGTGCTTCAGCCGCTACCCCGATCACATGAAGCAGCACGATTTCTTCAAGAGCGCC
ATGCCCGAGGGCTACGTGCAGGAGCGCACCATCAGCTTCAAGGATGACGGCACCTACAAGACCCGCGC
CGAGGTGAAGTTCGAGGGCGATACCCTGGTGAACCGCATCGAGCTGAAGGGCATCGATTTCAAGGAGG
ATGGCAACATCCTGGGCCACAAGCTGGAGTACAACTTCAACAGCCACAACGTGTACATCACCGCCGAT
AAGCAGAAGAACGGCATCAAGGCCAACTTCAAGATCCGCCACAATGTGGAGGATGGCTCCGTGCAGCT
GGCCGATCACTACCAGCAGAACACCCCCATCGGCGACGGCCCAGTGCTGCTGCCCGATAACCACTACCT
GAGCACCCAGAGCGTGCTGTCCAAGGACCCCAACGAGAAGCGCGATCACATGGTGCTGCTGGAGTTCG
Stop 3’ arm
TGACCGCCGCCGGCATCACCCTGGGTATGGATGAACTGTACAAATGATGAACTGACCTAACTCGAGGA
GGAGCTGGAATCTCTCGTGAGAGTAAGGAGAACGGTTCCTTCTGACAGAACTGATGCGCTGGAATTAA
AATGCATGCTCAAAGCCTAACCTCACAACCTTGGCTGGGGCTTTGGGACTGTAAGCTTCAGCCATAATT
TTAACTGCCTCAAACTTAAATAGTATAAAAGAACTTTTTTTTATGCTTCCCATCTTTTTTCTTTTTCCTTTT
AACAGATTTGTATTTAATTGTTTTTTTAAAAAAATCTTAAAATCTATCCAATTTTCCCATGTAAATAGGG
CCTTGAAATGTAAATAACTTTAATAAAACGTTTATAACAGTTACAAAAGATTTTAAGACATGTACCATA
ATTTTTTTTATTTAAAGACATTTTCATTTTTAAAGTTGATTTTTTTCTATTGTTTTTAGAAAAAAATAAAA
TAATTGGAAAAAATACAATTGGGCCAACTTGTGTTTTCTTTTTCCTCTTCCTCAAACTTCCTTTCCTCAAT
TACAGATTAAAAGAATTTGACCATTTTCACAGGGTAGGTTTACAAATATGGGAAGGGGTTATCATTGTT

AAAATGGGGCTGGGGGTCCTCAGGATTTCTAAGTTGTCTACAGGATGCTTTCTGTGGATAGTAATAAAA
ACCAGAGCTGTTAGTTAGGAATGGGCAAAAGGCAAGTGAGAAGGCTAGATGCAGGGAAGGGAAAAGC
AAGAGGTTAAAGATAACAGCTAAATATACAGGAGGAAGAGATGGCAGAATCTCCTACAGTTAACCGAA
GCCATTCCCTGGTTCACCTCAACCCAAGGACTCTGCCCTGCCAAAGAACTGGTGAGGGGAGGGAGAGA
GAACCACCGTTTGTTCCTTGCCTCTTGCTCCCAGGTGATAGTCCCTTCACATCAGTATCTCCTATGCTTCT
GAAAAAAACAGAGGAAGAGCATTACCACTGCTAAGTTGATCCTGGTTTTCCAAACAAGGACATACAAA
GGTTCAGAGGGTcagcttttgttccctttagtgagggttaattgcgcgcttggcgtaatcatggtcatagctgtttcctgtgtgaaattgttatccgctcacaattccac
acaacatacgagccggaagcataaagtgtaaagcctggggtgcctaatgagtgagctaactcacattaattgcgttgcgctcactgcccgctttccagtcgggaaacctgtc
gtgccagctgcattaatgaatcggccaacgcgcggggagaggcggtttgcgtattgggcgctcttccgcttcctcgctcactgactcgctgcgctcggtcgttcggctgcg
gcgagcggtatcagctcactcaaaggcggtaatacggttatccacagaatcaggggataacgcaggaaagaacatgtgagcaaaaggccagcaaaaggccaggaacc
gtaaaaaggccgcgttgctggcgtttttccataggctccgcccccctgacgagcatcacaaaaatcgacgctcaagtcagaggtggcgaaacccgacaggactataaag
ataccaggcgtttccccctggaagctccctcgtgcgctctcctgttccgaccctgccgcttaccggatacctgtccgcctttctcccttcgggaagcgtggcgctttctcatag
ctcacgctgtaggtatctcagttcggtgtaggtcgttcgctccaagctgggctgtgtgcacgaaccccccgttcagcccgaccgctgcgccttatccggtaactatcgtcttg
agtccaacccggtaagacacgacttatcgccactggcagcagccactggtaacaggattagcagagcgaggtatgtaggcggtgctacagagttcttgaagtggtggcct
aactacggctacactagaaggacagtatttggtatctgcgctctgctgaagccagttaccttcggaaaaagagttggtagctcttgatccggcaaacaaaccaccgctggta
gcggtggtttttttgtttgcaagcagcagattacgcgcagaaaaaaaggatctcaagaagatcctttgatcttttctacggggtctgacgctcagtggaacgaaaactcacgtt
aagggattttggtcatgagattatcaaaaaggatcttcacctagatccttttaaattaaaaatgaagttttaaatcaatctaaagtatatatgagtaaacttggtctgacagttacca
atgcttaatcagtgaggcacctatctcagcgatctgtctatttcgttcatccatagttgcctgactccccgtcgtgtagataactacgatacgggagggcttaccatctggcccc
agtgctgcaatgataccgcgagacccacgctcaccggctccagatttatcagcaataaaccagccagccggaagggccgagcgcagaagtggtcctgcaactttatccg
cctccatccagtctattaattgttgccgggaagctagagtaagtagttcgccagttaatagtttgcgcaacgttgttgccattgctacaggcatcgtggtgtcacgctcgtcgttt
ggtatggcttcattcagctccggttcccaacgatcaaggcgagttacatgatcccccatgttgtgcaaaaaagcggttagctccttcggtcctccgatcgttgtcagaagtaag
ttggccgcagtgttatcactcatggttatggcagcactgcataattctcttactgtcatgccatccgtaagatgcttttctgtgactggtgagtactcaaccaagtcattctgagaa
tagtgtatgcggcgaccgagttgctcttgcccggcgtcaatacgggataataccgcgccacatagcagaactttaaaagtgctcatcattggaaaacgttcttcggggcgaa
aactctcaaggatcttaccgctgttgagatccagttcgatgtaacccactcgtgcacccaactgatcttcagcatcttttactttcaccagcgtttctgggtgagcaaaaacagg
aaggcaaaatgccgcaaaaaagggaataagggcgacacggaaatgttgaatactcatactcttcctttttcaatattattgaagcatttatcagggttattgtctcatgagcgga
Plasmid backbone
tacatatttgaatgtatttagaaaaataaacaaataggggttccgcgcacatttccccgaaaagtgccac-3’

Supplementary Text S7 Sequence of mouse Myc-linker-sfGFP donor plasmid.

Supplementary Text S8

5'...ctaaattgtaagcgttaatattttgttaaaattcgcgttaaatttttgttaaatcagctcattttttaaccaataggccgaaatcggcaaaatcccttataaatcaaaagaatag
Plasmid backbone
accgagatagggttgagtgttgttccagtttggaacaagagtccactattaaagaacgtggactccaacgtcaaagggcgaaaaaccgtctatcagggcgatggcccacta
cgtgaaccatcaccctaatcaagttttttggggtcgaggtgccgtaaagcactaaatcggaaccctaaagggagcccccgatttagagcttgacggggaaagccggcgaa
cgtggcgagaaaggaagggaagaaagcgaaaggagcgggcgctagggcgctggcaagtgtagcggtcacgctgcgcgtaaccaccacacccgccgcgcttaatgc
gccgctacagggcgcgtcccattcgccattcaggctgcgcaactgttgggaagggcgatcggtgcgggcctcttcgctattacgccagctggcgaaagggggatgtgct
gcaaggcgattaagttgggtaacgccagggttttcccagtcacgacgttgtaaaacgacggccagtgagcgcgcgtaatacgactcactatagggcgaattggGTCC
5' arm
CCAGAGTTCTATTGCTTCATTCAGTGCTGACTCAGCCCAGAGGGTTCCCCCTGGATGGCTGCTCTTGTAG
TGAATAAACTCTAGGTTCCCTGCTCTTCGTTTTACATATTAGTGAGTGGCGACACGGTATATTTTCCTCT
CTAAGGGGGTTGGATCAAATTACGTTTTACTGGTATGTGGCCCTTAAATGATTCTTAGATATGGGGGAA
GATTCCATTTAGGAACATTGGCCGTGCATATTGCAGGGTGTAATTGAGCTTTAGAAAGTTAACCATGCT
GGCCAGGCAAAGGTGCCAAATACCAGGCAGTCAAAGGTCACTAGAGCCCAGATAAGTGACAGGGAAA
TAAGAATGGGGAAAGGTCCCGTGCGTATTAACCACTAATCTGCACCACGTGGTATACGTCAGTGAGTTG
ACACCTCGCCATGTCCCAGGGAAGTAGTCACTATGAGCCCATTTTGTAAGAGAGGAAACTGAGGCTCCT
TCCTTGGTGCAGCCCTTGGCTGTCTCTCAGCCAGGTGCCAGAACAGAAGGCAGAGCGGGCTACAGGAC
AGCAGCAGGGTGTGCCCAGCGCTGGGGGACAGATGGGAGTCTCCAGTCACTTCGGAAAAGTCATGCTT
TTCGCTGCCTAGCTAGCTAGCCCCGTCCGTATTCACGATCCTGGGTGCATGATGGGGAACTTGGGTGCA
GGGCAGGATGTCTAGTGTCCCAAGAGCCCACGGAGACCTCCTCTCCTCGTACCTGCAGAAACCAGCCTG
GACACCAAATCTGTGTCAGAAGGCCACCTCAAGAGGAACATCGTGGTAAAGACGGTGGAGATGCGGGA
TGGCGAGGTGAGGAAACGTTCCAGTGGCCCCGGGGAGTTCTTGAGGCTGTACTGGAGAAAGCCTGGTA
CTAGCTCACTGAAGGTCCTTATTAACTACCAGGAGAGCTCTGGTAGAAGGGATGGGCCTTGAATGTAAT
TCTCCTTTTCCAGCTCTTTCATGGGTGGCGTGCTAAGGGCAACCTGTATATGGAGGCCACCATCTTTCTG
GAACACTTGGTGGAGGGAGGCTTTCAAATTTTACTCAGAAGAACTTTCCATAGGGGAATTGGAAGAGG
GGCTAGACATAGAATGGCCGTCTTCATTTGTTGGGATGTTAGCAGCAATGGTTGCCCTTACGTCATAGT
CCTCTACAAGTGGCATTTCATATCTCAATGTGTGATGCAATCACAGCGATAGCTTCATAAGACACATCC
PAM1
AGGGCAGTCAGGGGCTCGCCTCGGGGATGCTCAGCCTAGCAAACTGGATCTGGGATTCTGGAGCCCCT
AACTCTGTGCTGTGCATCTTCCCTCTCCCTGCAGGTCATTAAGGAGTCGAAGCAGGAGCACAAGGATGT
p2A-EYFP
GATGGGATCCGGAGCTACTAATTTCTCCTTGCTTAAGCAAGCTGGTGATGTTGAAGAAAATCCTGGTCC
TATGGACTATGGCGGCGCTTTGTCTGCCGTCGGACGCGAACTTTTGTTCGTTACTAATCCTGTGGTGGTG
AACGGGTCCGTCCTGGTCCCTGAGGATCAATGTTACTGTGCCGGATGGATTGAATCTCGCGGCACGAAC
GGCGCTCAGACCGCGTCAAATGTCCTGCAGTGGCTTGCAGCAGGATTCAGCATTTTGCTGCTGATGTTC
TATGCCTACCAAACCTGGAAATCTACATGCGGCTGGGAGGAGATCTATGTGTGCGCCATTGAAATGGTT
AAGGTGATTCTCGAGTTCTTTTTTGAGTTTAAGAATCCCTCTATGCTCTACCTTGCCACAGGACACCGGG
TGCAGTGGCTGCGCTATGCAGAGTGGCTGCTCACTTGTCCTGTCATCCTTATCCGCCTGAGCAACCTCAC
CGGCCTGAGCAACGACTACAGCAGGAGAACCATGGGACTCCTTGTCTCAGACATCGGGACTATCGTGT
GGGGGGCTACCAGCGCCATGGCAACCGGCTATGTTAAAGTCATCTTCTTTTGTCTTGGATTGTGCTATG
GCGCGAACACATTTTTTCACGCCGCCAAAGCATATATCGAGGGTTATCATACTGTGCCAAAGGGTCGGT
GCCGCCAGGTCGTGACCGGCATGGCATGGCTGTTTTTCGTGAGCTGGGGTATGTTCCCAATTCTCTTCAT
TTTGGGGCCCGAAGGTTTTGGCGTCCTGAGCGTCTATGGCTCCACCGTAGGTCACACGATTATTGATCT
GATGAGTAAAAATTGTTGGGGGTTGTTGGGACACTACCTGCGCGTCCTGATCCACGAGCACATATTGAT
TCACGGAGATATCCGCAAAACCACCAAACTGAACATCGGCGGAACGGAGATCGAGGTCGAGACTCTCG

TCGAAGACGAAGCCGAGGCCGGAGCCGTGCCAGCGGCCGCCACCATGGTGAGCAAGGGCGAGGAGCT
GTTCACCGGGGTGGTGCCCATCCTGGTCGAGCTGGACGGCGACGTAAACGGCCACAAGTTCAGCGTGT
CCGGCGAGGGCGAGGGCGATGCCACCTACGGCAAGCTGACCCTGAAGTTCATCTGCACCACCGGCAAG
CTGCCCGTGCCCTGGCCCACCCTCGTGACCACCTTCGGCTACGGCCTGCAGTGCTTCGCCCGCTACCCCG
ACCACATGAAGCAGCACGACTTCTTCAAGTCCGCCATGCCCGAAGGCTACGTCCAGGAGCGCACCATCT
TCTTCAAGGACGACGGCAACTACAAGACCCGCGCCGAGGTGAAGTTCGAGGGCGACACCCTGGTGAAC
CGCATCGAGCTGAAGGGCATCGACTTCAAGGAGGACGGCAACATCCTGGGGCACAAGCTGGAGTACAA
CTACAACAGCCACAACGTCTATATCATGGCCGACAAGCAGAAGAACGGCATCAAGGTGAACTTCAAGA
TCCGCCACAACATCGAGGACGGCAGCGTGCAGCTCGCCGACCACTACCAGCAGAACACCCCCATCGGC
GACGGCCCCGTGCTGCTGCCCGACAACCACTACCTGAGCTACCAGTCCGCCCTGAGCAAAGACCCCAA
CGAGAAGCGCGATCACATGGTCCTGCTGGAGTTCGTGACCGCCGCCGGGATCACTCTCGGCATGGACG
Stop Stop PAM2 3’ arm
AGCTGTACAAGTAAATGTGAGGTGTGCCCAGCTGGCAGCCCTTGCCATACAGTGTGAGGGCCTAAAGC
TCCCTCCTCAGATAGTCTTGTTTGCTAGGCCCAATTCCCATCCACACCAGTGCTCCCCTTCCTTCTGTTTT
TATGCCCACGGCTCGGTCAGTGCGGAGTCTCATGGACGGCACAGACCACCCTGCATCTCCAACTAACAG
GATACTCACCCCAAAGGGGCAATCAGGAGGGGAGGACCCCCCTCCCCCCAGCTGGGTTAGAACTGGAA
GAAAGAGGAAAGACAGGGGCAGGGAGACTTAACAAATCCCTTCCTTCATCCTTGTTGTTATGGAAACC
GTTGCCAGAGCTGGAGGTCTCTGGGAACTGGACTTTGAGTTTTCATAGGCTGCTGGAGCAAGACAAACA
TTCAGACAGAAAGGAAAAGATCCCGAGGCAAAGAATCTCTAGCCAGAGGCCTAGGCATCTGGAAGAA
CTATTCAGATGTAGGAGTGGGTAGGGCAGACTTGCTACCTGGAATGGCCACTAAGGCAGTCCTGAAGG
GCCCCCCTCCGGAGGGATGACCCTCGTGTATCGGCCCCACTGAGCAGCCCTGCAGGTTGATGCCCCACG
AGCGTGTGAAAACTTGGTTCTTGGCATGTGGCAGGCTCTATAGCATAAGTGGAGAGGGAAGGTGTACT
GGAGGGTATAGAGGAGGGCTCTCTGGCCCCTAAGTATGGATGCGGAGAGGGGGGAGCCCAGGAAGGC
TACCCCGCTCAGGCTGCAGGGGTGCCATGGCGGAGGAACCGGTGGAGATAACTTGGACAATGGAGTTG
GAAGTTGTAGGCAACTAGTTACACTTGGCTCTGAATCCTTGGAATCAAGGAAATGACCTGTTCTCTCAA
AGACACTGAAACAGGAGAGAGGGACTTCCATCCACTGGGCAGGGTACAGGCGCGTCTCAGTTGTGAAG
GTCTATTCCTGGTTGCTCAGTCCCCAACTGCGCATCACCCTGGGCTTCTCAACCTGGAAGAGTCCACAA
CCATCCTTCTGAGGCCCTCCATCCCCACAACCACTAGCTGTTGTTCTCCAAGCCAAGGGCCCCATTCCCT
TTCTTATGCATGTACGGAGTATCGCCTAGACTTTAAGCGTCCATCCTGTTTGAAAGTTTGGGAAACTGAC
ACACGTTGTGTTCAAGCAGCCTGGTGTGGAGTGCCTTCGTATTAGTGTACCCTCTCGGAAGCTGGTTGG
TGGGCAGGTGAGGAAGAAATGGAGCTGAAAGTGTCCCCTCAGTTGTCCTTTCCTCCCCCTCTAAGGTCC
CTCCCTTTTCCCAGGACATCGTACACTCCCCCCCTTGTCACCTCTGCTAACCTTCAGAGCAGTACTGTCA
CCTTTACTCACTGGGCAGAAATAAAGACAGTGTCAGAGGCTTCCACAGAGTGGATCTGATTTGTTCAGA
AGGTGGGGTGAGGACAGACAAGACCCAACTTGCTCGTTATCACCACCGAATGTCTAGCGAGCCTAAAC
Plasmid backbone
CCTCCACAACAAACCCTcagcttttgttccctttagtgagggttaattgcgcgcttggcgtaatcatggtcatagctgtttcctgtgtgaaattgttatccgctcac
aattccacacaacatacgagccggaagcataaagtgtaaagcctggggtgcctaatgagtgagctaactcacattaattgcgttgcgctcactgcccgctttccagtcggga
aacctgtcgtgccagctgcattaatgaatcggccaacgcgcggggagaggcggtttgcgtattgggcgctcttccgcttcctcgctcactgactcgctgcgctcggtcgttc
ggctgcggcgagcggtatcagctcactcaaaggcggtaatacggttatccacagaatcaggggataacgcaggaaagaacatgtgagcaaaaggccagcaaaaggcc
aggaaccgtaaaaaggccgcgttgctggcgtttttccataggctccgcccccctgacgagcatcacaaaaatcgacgctcaagtcagaggtggcgaaacccgacaggac
tataaagataccaggcgtttccccctggaagctccctcgtgcgctctcctgttccgaccctgccgcttaccggatacctgtccgcctttctcccttcgggaagcgtggcgcttt
ctcatagctcacgctgtaggtatctcagttcggtgtaggtcgttcgctccaagctgggctgtgtgcacgaaccccccgttcagcccgaccgctgcgccttatccggtaactat
cgtcttgagtccaacccggtaagacacgacttatcgccactggcagcagccactggtaacaggattagcagagcgaggtatgtaggcggtgctacagagttcttgaagtg

gtggcctaactacggctacactagaaggacagtatttggtatctgcgctctgctgaagccagttaccttcggaaaaagagttggtagctcttgatccggcaaacaaaccacc
gctggtagcggtggtttttttgtttgcaagcagcagattacgcgcagaaaaaaaggatctcaagaagatcctttgatcttttctacggggtctgacgctcagtggaacgaaaac
tcacgttaagggattttggtcatgagattatcaaaaaggatcttcacctagatccttttaaattaaaaatgaagttttaaatcaatctaaagtatatatgagtaaacttggtctgaca
gttaccaatgcttaatcagtgaggcacctatctcagcgatctgtctatttcgttcatccatagttgcctgactccccgtcgtgtagataactacgatacgggagggcttaccatct
ggccccagtgctgcaatgataccgcgagacccacgctcaccggctccagatttatcagcaataaaccagccagccggaagggccgagcgcagaagtggtcctgcaact
ttatccgcctccatccagtctattaattgttgccgggaagctagagtaagtagttcgccagttaatagtttgcgcaacgttgttgccattgctacaggcatcgtggtgtcacgctc
gtcgtttggtatggcttcattcagctccggttcccaacgatcaaggcgagttacatgatcccccatgttgtgcaaaaaagcggttagctccttcggtcctccgatcgttgtcaga
agtaagttggccgcagtgttatcactcatggttatggcagcactgcataattctcttactgtcatgccatccgtaagatgcttttctgtgactggtgagtactcaaccaagtcattc
tgagaatagtgtatgcggcgaccgagttgctcttgcccggcgtcaatacgggataataccgcgccacatagcagaactttaaaagtgctcatcattggaaaacgttcttcgg
ggcgaaaactctcaaggatcttaccgctgttgagatccagttcgatgtaacccactcgtgcacccaactgatcttcagcatcttttactttcaccagcgtttctgggtgagcaaa
aacaggaaggcaaaatgccgcaaaaaagggaataagggcgacacggaaatgttgaatactcatactcttcctttttcaatattattgaagcatttatcagggttattgtctcatg
agcggatacatatttgaatgtatttagaaaaataaacaaataggggttccgcgcacatttccccgaaaagtgccac-3’

Supplementary Text S8 Sequence of rat *GFAP* targeting construct *p2A-mEYFP*.

Supplementary Text S9

5'...ctaaattgtaagcgttaatattttgttaaaattcgcgttaaatttttgttaaatcagctcattttttaaccaataggccgaaatcggcaaaatcccttataaatcaaaagaatag
Plasmid backbone
accgagatagggttgagtgttgttccagtttggaacaagagtccactattaaagaacgtggactccaacgtcaaagggcgaaaaaccgtctatcagggcgatggcccacta
cgtgaaccatcaccctaatcaagttttttggggtcgaggtgccgtaaagcactaaatcggaaccctaaagggagcccccgatttagagcttgacggggaaagccggcgaa
cgtggcgagaaaggaagggaagaaagcgaaaggagcgggcgctagggcgctggcaagtgtagcggtcacgctgcgcgtaaccaccacacccgccgcgcttaatgc
gccgctacagggcgcgtcccattcgccattcaggctgcgcaactgttgggaagggcgatcggtgcgggcctcttcgctattacgccagctggcgaaagggggatgtgct
gcaaggcgattaagttgggtaacgccagggttttcccagtcacgacgttgtaaaacgacggccagtgagcgcgcgtaatacgactcactatagggcgaattggGTCC
5' arm
ATTCTGAACCTCTGCGTGATCAGCGTGGACAGGTACTGGGCTATCTCCAGCCCTTTCCAGTATGAGAGG
AAGATGACCCCCAAAGCAGCCTTCATCCTGATTAGCGTAGCATGGACTCTGTCTGTCCTTATATCCTTCA
TCCCAGTACAGCTAAGCTGGCACAAGGCAAAGCCCACATGGCCCTTGGATGGCAATTTTACCTCCCTGG
AGGACACCGAGGATGACAACTGTGACACAAGGTTGAGCAGGACGTATGCCATTTCATCGTCCCTCATC
AGCTTTTACATCCCCGTAGCCATTATGATCGTCACCTACACCAGTATCTACAGGATTGCCCAGAAGCAA
ATCCGGCGCATCTCAGCCTTGGAGAGGGCAGCAGTCCATGCCAAGAATTGCCAGACCACCGCAGGTAA
CGGGAACCCCGTCGAATGCGCCCAGTCTGAAAGTTCCTTTAAGATGTCCTTCAAGAGGGAGACGAAAG
TTCTAAAGACGCTGTCTGTGATCATGGGGGTGTTTGTGTGCTGCTGGCTCCCTTTCTTCATCTCGAACTG
TATGGTGCCCTTCTGTGGCTCTGAGGAGACCCAGCCATTCTGCATCGATTCCATCACCTTCGATGTGTTT
GTGTGGTTTGGGTGGGCGAATTCTTCCCTGAACCCCATTATTTATGCTTTTAATGCTGACTTCCAGAAGG
CGTTCTCAACCCTCTTAGGATGCTACAGACTCTGCCCTACTACGAATAATGCCATAGAGACGGTGAGCA
TTAACAACAATGGGGCTGTGGTGTTTTCCAGCCACCATGAGCCCCGAGGCTCCATCTCCAAGGACTGTA
ATCTGGTTTACCTGATCCCTCATGCCGTGGGCTCCTCTGAGGACCTGAAGAAGGAAGAGGCTGGTGGAA
TAGCTAAGCCACTGGAGAAGCTGTCCCCAGCCTTATCGGTCATATTGGACTATGACACCGATGTCTCTC
PAM1 Stop p2A-ChR2::EYFP
TAGAAAAGATCCAACCTGTCACACACAGTGGACAGCATTCCACT(~~TGA~~)GGATCCGGAGCTACTAATTTC
TCCTTGCTTAAGCAAGCTGGTGATGTTGAAGAAAATCCTGGTCCTATGGACTATGGCGGCGCTTTGTCT
GCCGTCGGACGCGAACTTTTGTTCGTTACTAATCCTGTGGTGGTGAACGGGTCCGTCCTGGTCCCTGAG
GATCAATGTTACTGTGCCGGATGGATTGAATCTCGCGGCACGAACGGCGCTCAGACCGCGTCAAATGTC
CTGCAGTGGCTTGCAGCAGGATTCAGCATTTTGCTGCTGATGTTCTATGCCTACCAAACCTGGAAATCT
ACATGCGGCTGGGAGGAGATCTATGTGTGCGCCATTGAAATGGTTAAGGTGATTCTCGAGTTCTTTTTT
GAGTTTAAGAATCCCTCTATGCTCTACCTTGCCACAGGACACCGGGTGCAGTGGCTGCGCTATGCAGAG
TGGCTGCTCACTTGTCCTGTCATCCTTATCCGCCTGAGCAACCTCACCGGCCTGAGCAACGACTACAGC
AGGAGAACCATGGGACTCCTTGTCTCAGACATCGGGACTATCGTGTGGGGGGCTACCAGCGCCATGGC
AACCGGCTATGTTAAAGTCATCTTCTTTTGTCTTGGATTGTGCTATGGCGCGAACACATTTTTTCACGCC
GCCAAAGCATATATCGAGGGTTATCATACTGTGCCAAAGGGTCGGTGCCGCCAGGTCGTGACCGGCAT
GGCATGGCTGTTTTTCGTGAGCTGGGGTATGTTCCCAATTCTCTTCATTTTGGGGCCCGAAGGTTTTGGC
GTCCTGAGCGTCTATGGCTCCACCGTAGGTCACACGATTATTGATCTGATGAGTAAAAATTGTTGGGGG
TTGTTGGGACACTACCTGCGCGTCCTGATCCACGAGCACATATTGATTCACGGAGATATCCGCAAAACC
ACCAAACTGAACATCGGCGGAACGGAGATCGAGGTCGAGACTCTCGTCGAAGACGAAGCCGAGGCCG
GAGCCGTGCCAGCGGCCGCCACCATGGTGAGCAAGGGCGAGGAGCTGTTCACCGGGGTGGTGCCCATC
CTGGTCGAGCTGGACGGCGACGTAAACGGCCACAAGTTCAGCGTGTCCGGCGAGGGCGAGGGCGATGC
CACCTACGGCAAGCTGACCCTGAAGTTCATCTGCACCACCGGCAAGCTGCCCGTGCCCTGGCCCACCCT
CGTGACCACCTTCGGCTACGGCCTGCAGTGCTTCGCCCGCTACCCCGACCACATGAAGCAGCACGACTT
CTTCAAGTCCGCCATGCCCGAAGGCTACGTCCAGGAGCGCACCATCTTCTTCAAGGACGACGGCAACTA

CAAGACCCGCGCCGAGGTGAAGTTCGAGGGCGACACCCTGGTGAACCGCATCGAGCTGAAGGGCATCG
ACTTCAAGGAGGACGGCAACATCCTGGGGCACAAGCTGGAGTACAACTACAACAGCCACAACGTCTAT
ATCATGGCCGACAAGCAGAAGAACGGCATCAAGGTGAACTTCAAGATCCGCCACAACATCGAGGACGG
CAGCGTGCAGCTCGCCGACCACTACCAGCAGAACACCCCCATCGGCGACGGCCCCGTGCTGCTGCCCG
ACAACCACTACCTGAGCTACCAGTCCGCCCTGAGCAAAGACCCCAACGAGAAGCGCGATCACATGGTC
Stop
CTGCTGGAGTTCGTGACCGCCGCCGGGATCACTCTCGGCATGGACGAGCTGTACAAGTAAATATTGGGT
PAM2
CCTCATCTCTGAGGCCACGAGTTCCCTTGGGCTTGCTGTTAAGGAATTAACAGGAGATCCCTCTGCTGCT
TTTGGACAATTACGAAGCTTCTCAAACTCACTGATTCCAGTGTATTCTCTAGCTTCAAGGGAAATGACTT
CGGCTCTGAAATCAGTTTGGGAGTATTATCTTAGGACATTATAAAACAACAACAAACAAACAAACAAA
CAAACAAATAGGGCCAAGAGTCAACTGTAAACAGCTTCACTTAAAAATCGAACTTTCCAGAAAGGAAG
GGTAGGAGTTGAGTTTGCTGTCCAAACAGGTGCTAAAACTGTCCGAGCAGTTTTCAGATTGGAAAGGTA
GGTGCATGCCTTTGTTAATTAACTTCTCCAATAATAATTGAGCCTTACAGCAGGAGTGGGATTCCTTTTT
CTCAGAATTGACAGATGCATTGTTGATGACGGTTTTATTTATTTATTTATTGTACTATATGAATATTTTA
AATTTATCATAGTGAATCTATATTTAACATATTTAACAGAGCAAACCAATGTGTTATCTGAGACTGACC
TCTCCATTTGTACTAGCACTTTATGAGCCAATGAAACATACGCGTAGACTCTGAGATTCTGAATTGTGA
GTTACTTCTGGGAACACAGCAAAGACTGATGTGGTGGCTCCTTAACTCGACAAGGACACAAAGAAACG
CAAGAGGAGAAGTGACTAATGCCACCAATGCTCCCCCTAAAAAGATTTTGAAAAGATTAGTTTTTTTTT
TTTTTAAAAGAAGCTACTATTGTGTTCTGAATGTTTTAAATGGCAGAGGCTTTCCCCGGGGCGAATTTGC
ACTTCTGTAAATATCTATGTAAGAACCAGCTCAAGAGGAATACAACTTTATATTTCCGCTTTTGGATGG
CGAGGAAGAGCATATGCCACTTTGTATTTATGTAAAATAATTGGCCCTCCTTGTCATTTCTCATTTCATG
CTTGAAATAGCTTTCTGAAACAAACAAATGACTGTCCAGGCTGGAGATCTGCAGGGTGGAGAATGAGT
TGTAAATTCACAGGTCACAGCAGCCCCTCCGATAGCTGGGCTCATCATTGGTCCTTTATCTGCCCAGGTC
TAACCAAGTCGGCTGCTTAAGGGGCTACTTTTGTAGTGCTTTAATCCGAATTTAGTATCCTCTCTTTAAA
AAAAAAAAGCTCTTTAATGTTAGTGGTAAACTAGCTAATGAACGGTACCTCATCGCTGCATAATACACT
TCTGTTGGTGGGGGCGTAGACGAGCCCCTTCCCGGTGCGAGCACCACAAAGCCATCTGCATAGCTAGTC
ACAAATGCTGTTTTTCTTTCTCTGTGGGTTTGAATCTAGTTTCCTTGTTATCATAGCCTGGACTGCAAAA
3' arm Plasmid backbone
AGAACCATCGAGTCCCCTCTTGTGGGGGCATTGCAACAGTGTcagcttttgttccctttagtgagggttaattgcgcgcttggcgtaat
catggtcatagctgtttcctgtgtgaaattgttatccgctcacaattccacacaacatacgagccggaagcataaagtgtaaagcctggggtgcctaatgagtgagctaactc
acattaattgcgttgcgctcactgcccgctttccagtcgggaaacctgtcgtgccagctgcattaatgaatcggccaacgcgcggggagaggcggtttgcgtattgggcgct
cttccgcttcctcgctcactgactcgctgcgctcggtcgttcggctgcggcgagcggtatcagctcactcaaaggcggtaatacggttatccacagaatcaggggataacg
caggaaagaacatgtgagcaaaaggccagcaaaaggccaggaaccgtaaaaaggccgcgttgctggcgtttttccataggctccgcccccctgacgagcatcacaaaa
atcgacgctcaagtcagaggtggcgaaacccgacaggactataaagataccaggcgtttccccctggaagctccctcgtgcgctctcctgttccgaccctgccgcttaccg
gatacctgtccgcctttctcccttcgggaagcgtggcgctttctcatagctcacgctgtaggtatctcagttcggtgtaggtcgttcgctccaagctgggctgtgtgcacgaac
cccccgttcagcccgaccgctgcgccttatccggtaactatcgtcttgagtccaacccggtaagacacgacttatcgccactggcagcagccactggtaacaggattagca
gagcgaggtatgtaggcggtgctacagagttcttgaagtggtggcctaactacggctacactagaaggacagtatttggtatctgcgctctgctgaagccagttaccttcgg
aaaaagagttggtagctcttgatccggcaaacaaaccaccgctggtagcggtggtttttttgtttgcaagcagcagattacgcgcagaaaaaaaggatctcaagaagatcct
ttgatcttttctacggggtctgacgctcagtggaacgaaaactcacgttaagggattttggtcatgagattatcaaaaaggatcttcacctagatccttttaaattaaaaatgaagt
tttaaatcaatctaaagtatatatgagtaaacttggtctgacagttaccaatgcttaatcagtgaggcacctatctcagcgatctgtctatttcgttcatccatagttgcctgactcc
ccgtcgtgtagataactacgatacgggagggcttaccatctggccccagtgctgcaatgataccgcgagacccacgctcaccggctccagatttatcagcaataaaccag
ccagccggaagggccgagcgcagaagtggtcctgcaactttatccgcctccatccagtctattaattgttgccgggaagctagagtaagtagttcgccagttaatagtttgc
gcaacgttgttgccattgctacaggcatcgtggtgtcacgctcgtcgtttggtatggcttcattcagctccggttcccaacgatcaaggcgagttacatgatcccccatgttgtg

caaaaaagcggttagctccttcggtcctccgatcgttgtcagaagtaagttggccgcagtgttatcactcatggttatggcagcactgcataattctcttactgtcatgccatcc
gtaagatgcttttctgtgactggtgagtactcaaccaagtcattctgagaatagtgtatgcggcgaccgagttgctcttgcccggcgtcaatacgggataataccgcgccaca
tagcagaactttaaaagtgctcatcattggaaaacgttcttcggggcgaaaactctcaaggatcttaccgctgttgagatccagttcgatgtaacccactcgtgcacccaactg
atcttcagcatcttttactttcaccagcgtttctgggtgagcaaaaacaggaaggcaaaatgccgcaaaaaagggaataagggcgacacggaaatgttgaatactcatactc
ttcctttttcaatattattgaagcatttatcagggttattgtctcatgagcggatacatatttgaatgtatttagaaaaataaacaaataggggttccgcgcacatttccccgaaaagt
gccac-3’

Supplementary Text S9 Sequence of rat *Drd1* targeting construct *p2A-ChR2-EYFP*.

Supplementary Text S10

5’...ctaaattgtaagcgttaatattttgttaaaattcgcgttaaatttttgttaaatcagctcattttttaaccaataggccgaaatcggcaaaatcccttataaatcaaaagaatag
Plasmid backbone
accgagatagggttgagtgttgttccagtttggaacaagagtccactattaaagaacgtggactccaacgtcaaagggcgaaaaaccgtctatcagggcgatggcccacta
cgtgaaccatcaccctaatcaagttttttggggtcgaggtgccgtaaagcactaaatcggaaccctaaagggagcccccgatttagagcttgacggggaaagccggcgaa
cgtggcgagaaaggaagggaagaaagcgaaaggagcgggcgctagggcgctggcaagtgtagcggtcacgctgcgcgtaaccaccacacccgccgcgcttaatgc
gccgctacagggcgcgtcccattcgccattcaggctgcgcaactgttgggaagggcgatcggtgcgggcctcttcgctattacgccagctggcgaaagggggatgtgct
gcaaggcgattaagttgggtaacgccagggttttcccagtcacgacgttgtaaaacgacggccagtgagcgcgcgtaatacgactcactatagggcgaattggCATA
5’ arm
TCTGAGGTGTGGCAGGATGGGTTTGGAGCAAGCTTCATGTGACAGTCTACCAGTTCTACCCTTGTTACT
CCAAGACACTGCTCTGTCCTTAAACATCCCCTATAACTGCACACTGAGTATCTGACTGGAGCAAGTAGT
CTTGAAGCCTGCTTTTGCAAGAGGGCGTGAACTCCAGGTAGGACCAACAGAGGGTACGTCTACCAATA
CTGAGCAATCTTAGGAAAAGTTACAAGGGCTTTGACACTTCAGGGGTCTAGACAGATGGGAATCAACT
ATCATTTGTGTCAGTCAAGAGCAAAGTGTCTTGGGGACATTGTGGAGTCAATAAGTATAGTTTTCCAAT
GAATTCTAGGAGTCACCATGAAAACTAAGTACGACGGGTATTTGGTTTTTTCCTAAATTTTATGCCCCAC
ACTGCCGAGAGCAAATTCCATCCAATGTCATCAGTTTGAAGAAAATATTGCCCACAGTGGTGACTGGCA
AGTAGTTGGTGTGCCTATTTGCAGAGACCAACTGGAGTCAGATGGCTGGCATTTCCCATTTGTTTCTCAA
GCCACTTCCTTCTTTAGCAGGTGGATGTCATGCCTGTTGAGCCAATGAGGAAAATGAGGCTGAGGGAGA
TGCAGGGAAGATGTGTGCTAAAAAAGCACATGTTTAAATCTGAGAGCAGGGCTCCCTTTGCTGCAACA
CTGGGTTACCACCAGCAGTCAGCAGTCTCAGTGCCCCAGAGAAGCCTGTGGCAGAACAGAGAGATGGA
TGGGGAGTGGGACCCACCTAGTCCAGTCTTTCCTTCGCCTGCCGCCAGCCTGACCCTGCCCTTTTCTCAG
GTGTGTTCATCATCTGCTGGCTGCCCTTCTTCATCACGCACATCCTGAATATACACTGTGATTGCAACAT
CCCACCAGTCCTCTACAGCGCCTTCACATGGCTGGGCTATGTCAACAGTGCCGTCAACCCCATCATCTA
PAM1 CCT-CtT p2A-ChR2::EYFP
CACCACCTTCAACATCGAGTTCCGCAAaGCtTTtATGAAaATacTcCAtTGtGGATCCGGAGCTACTAATTTC
TCCTTGCTTAAGCAAGCTGGTGATGTTGAAGAAAATCCTGGTCCTATGGACTATGGCGGCGCTTTGTCT
GCCGTCGGACGCGAACTTTTGTTCGTTACTAATCCTGTGGTGGTGAACGGGTCCGTCCTGGTCCCTGAG
GATCAATGTTACTGTGCCGGATGGATTGAATCTCGCGGCACGAACGGCGCTCAGACCGCGTCAAATGTC
CTGCAGTGGCTTGCAGCAGGATTCAGCATTTTGCTGCTGATGTTCTATGCCTACCAAACCTGGAAATCT
ACATGCGGCTGGGAGGAGATCTATGTGTGCGCCATTGAAATGGTTAAGGTGATTCTCGAGTTCTTTTTT
GAGTTTAAGAATCCCTCTATGCTCTACCTTGCCACAGGACACCGGGTGCAGTGGCTGCGCTATGCAGAG
TGGCTGCTCACTTGTCCTGTCATCCTTATCCGCCTGAGCAACCTCACCGGCCTGAGCAACGACTACAGC
AGGAGAACCATGGGACTCCTTGTCTCAGACATCGGGACTATCGTGTGGGGGGCTACCAGCGCCATGGC
AACCGGCTATGTTAAAGTCATCTTCTTTTGTCTTGGATTGTGCTATGGCGCGAACACATTTTTTCACGCC
GCCAAAGCATATATCGAGGGTTATCATACTGTGCCAAAGGGTCGGTGCCGCCAGGTCGTGACCGGCAT
GGCATGGCTGTTTTTCGTGAGCTGGGGTATGTTCCCAATTCTCTTCATTTTGGGGCCCGAAGGTTTTGGC
GTCCTGAGCGTCTATGGCTCCACCGTAGGTCACACGATTATTGATCTGATGAGTAAAAATTGTTGGGGG
TTGTTGGGACACTACCTGCGCGTCCTGATCCACGAGCACATATTGATTCACGGAGATATCCGCAAAACC
ACCAAACTGAACATCGGCGGAACGGAGATCGAGGTCGAGACTCTCGTCGAAGACGAAGCCGAGGCCG
GAGCCGTGCCAGCGGCCGCCACCATGGTGAGCAAGGGCGAGGAGCTGTTCACCGGGGTGGTGCCCATC
CTGGTCGAGCTGGACGGCGACGTAAACGGCCACAAGTTCAGCGTGTCCGGCGAGGGCGAGGGCGATGC
CACCTACGGCAAGCTGACCCTGAAGTTCATCTGCACCACCGGCAAGCTGCCCGTGCCCTGGCCCACCCT
CGTGACCACCTTCGGCTACGGCCTGCAGTGCTTCGCCCGCTACCCCGACCACATGAAGCAGCACGACTT
CTTCAAGTCCGCCATGCCCGAAGGCTACGTCCAGGAGCGCACCATCTTCTTCAAGGACGACGGCAACTA

CAAGACCCGCGCCGAGGTGAAGTTCGAGGGCGACACCCTGGTGAACCGCATCGAGCTGAAGGGCATCG
ACTTCAAGGAGGACGGCAACATCCTGGGGCACAAGCTGGAGTACAACTACAACAGCCACAACGTCTAT
ATCATGGCCGACAAGCAGAAGAACGGCATCAAGGTGAACTTCAAGATCCGCCACAACATCGAGGACGG
CAGCGTGCAGCTCGCCGACCACTACCAGCAGAACACCCCCATCGGCGACGGCCCCGTGCTGCTGCCCG
ACAACCACTACCTGAGCTACCAGTCCGCCCTGAGCAAAGACCCCAACGAGAAGCGCGATCACATGGTC
Stop
CTGCTGGAGTTCGTGACCGCCGCCGGGATCACTCTCGGCATGGACGAGCTGTACAAGTAGGTCTGCCCC
3’ arm PAM2
TTGCCTGCACAGCAGCTGCTTCCCACCTCCCTGCCTATGCAGGCCAGACCTCATCCCTGCAAGCTGTGG
GCAGAAAGGCCCAGATGAACTTGGCCTTCTCTCGACCCTGCAGGCCCTGCAGTGTTAGCTTGGCTCGAT
GCCCCTCTCTGCCCACACACCCTCATCCTGCCAGGGTAGGGCCAGGGAGACTGGTATCTTACCAGCTCT
GGGGTTGGACCCATGGCTCAGGGCAGCTCACAGAGTGCCCCTCTCATATCCAGACCCTGTCTCCTTGGC
ACCAAAGATGCAGCGGCCTTCCTTGACCTTCCTCTTGGGCACAGAAACTAGCTCAGTGGTCGAGCACAC
CCTGATCGCTGGCTTGGCCTGGCCCTTGCTTGCCTGTGCCGGATCAGGTGGTGGGAGGGAGCGACAGTT
CTTACTTTATAGGAACCACATAGGAAAGCAGGGAACACGCCAAGTCCTCCAGGCAACATCAGTGTCAG
GAGACACACATAAACACCAGGTAGCTCCATGGACCCCAGAGAAACTGAGGCTGAAAAATCTGTTTTCC
ACTCCAACTCTAGTGTGAGTCCCTACTTTTCATAGCCATGGGTATTACTATGTCCTACCTTGTTATAGTA
TCCCATGGGGTTTCTGTACCATTTGGGGGAAAACAACTCTAATCCTCAAGGGCCCCAAGAGAATCTGTA
AGGAGAAAAATAGCTGATCTCCCTCTACTCTCCAATCCACTCCACCACTTCTTGATATACCTTGGATGTA
TCCATTCCTCACAGCAAATGCTGGCCAGTCAGGCCTTGGACCAGTGTTGGAGTTGAAGCTGGATGTGGT
AACTTGGGGCTCTTTGGGGCTGGGGGGGTTGTTAACATCGTCTCTCTTCCATATCTCTTCCTTCCCAGTG
CCTCTGCCTTAGAAGAGGCTGTGGATGGGGTGCTGGGACTGCTGATACCATTGGGCCTGGCCCTGAATG
AGGAGGGGAAGCTGCAGTTTGcagcttttgttccctttagtgagggttaattgcgcgcttggcgtaatcatggtcatagctgtttcctgtgtgaaattgttatc
cgctcacaattccacacaacatacgagccggaagcataaagtgtaaagcctggggtgcctaatgagtgagctaactcacattaattgcgttgcgctcactgcccgctttcca
gtcgggaaacctgtcgtgccagctgcattaatgaatcggccaacgcgcggggagaggcggtttgcgtattgggcgctcttccgcttcctcgctcactgactcgctgcgctc
ggtcgttcggctgcggcgagcggtatcagctcactcaaaggcggtaatacggttatccacagaatcaggggataacgcaggaaagaacatgtgagcaaaaggccagca
aaaggccaggaaccgtaaaaaggccgcgttgctggcgtttttccataggctccgcccccctgacgagcatcacaaaaatcgacgctcaagtcagaggtggcgaaacccg
acaggactataaagataccaggcgtttccccctggaagctccctcgtgcgctctcctgttccgaccctgccgcttaccggatacctgtccgcctttctcccttcgggaagcgt
ggcgctttctcatagctcacgctgtaggtatctcagttcggtgtaggtcgttcgctccaagctgggctgtgtgcacgaaccccccgttcagcccgaccgctgcgccttatccg
gtaactatcgtcttgagtccaacccggtaagacacgacttatcgccactggcagcagccactggtaacaggattagcagagcgaggtatgtaggcggtgctacagagttct
tgaagtggtggcctaactacggctacactagaaggacagtatttggtatctgcgctctgctgaagccagttaccttcggaaaaagagttggtagctcttgatccggcaaacaa
accaccgctggtagcggtggtttttttgtttgcaagcagcagattacgcgcagaaaaaaaggatctcaagaagatcctttgatcttttctacggggtctgacgctcagtggaac
gaaaactcacgttaagggattttggtcatgagattatcaaaaaggatcttcacctagatccttttaaattaaaaatgaagttttaaatcaatctaaagtatatatgagtaaacttggt
ctgacagttaccaatgcttaatcagtgaggcacctatctcagcgatctgtctatttcgttcatccatagttgcctgactccccgtcgtgtagataactacgatacgggagggctt
accatctggccccagtgctgcaatgataccgcgagacccacgctcaccggctccagatttatcagcaataaaccagccagccggaagggccgagcgcagaagtggtcc
tgcaactttatccgcctccatccagtctattaattgttgccgggaagctagagtaagtagttcgccagttaatagtttgcgcaacgttgttgccattgctacaggcatcgtggtgt
cacgctcgtcgtttggtatggcttcattcagctccggttcccaacgatcaaggcgagttacatgatcccccatgttgtgcaaaaaagcggttagctccttcggtcctccgatcg
ttgtcagaagtaagttggccgcagtgttatcactcatggttatggcagcactgcataattctcttactgtcatgccatccgtaagatgcttttctgtgactggtgagtactcaacca
agtcattctgagaatagtgtatgcggcgaccgagttgctcttgcccggcgtcaatacgggataataccgcgccacatagcagaactttaaaagtgctcatcattggaaaacg
ttcttcggggcgaaaactctcaaggatcttaccgctgttgagatccagttcgatgtaacccactcgtgcacccaactgatcttcagcatcttttactttcaccagcgtttctgggt
gagcaaaaacaggaaggcaaaatgccgcaaaaaagggaataagggcgacacggaaatgttgaatactcatactcttcctttttcaatattattgaagcatttatcagggttatt
gtctcatgagcggatacatatttgaatgtatttagaaaaataaacaaataggggttccgcgcacatttccccgaaaagtgccac-3’

Supplementary Text S10 Sequence of rat *Drd2* targeting construct *p2A-ChR2-EYFP*.

Supplementary Text S11

5’...ctaaattgtaagcgttaatattttgttaaaattcgcgttaaatttttgttaaatcagctcattttttaaccaataggccgaaatcggcaaaatcccttataaatcaaaagaatag
Plasmid backbone
accgagatagggttgagtgttgttccagtttggaacaagagtccactattaaagaacgtggactccaacgtcaaagggcgaaaaaccgtctatcagggcgatggcccacta
cgtgaaccatcaccctaatcaagttttttggggtcgaggtgccgtaaagcactaaatcggaaccctaaagggagcccccgatttagagcttgacggggaaagccggcgaa
cgtggcgagaaaggaagggaagaaagcgaaaggagcgggcgctagggcgctggcaagtgtagcggtcacgctgcgcgtaaccaccacacccgccgcgcttaatgc
gccgctacagggcgcgtcccattcgccattcaggctgcgcaactgttgggaagggcgatcggtgcgggcctcttcgctattacgccagctggcgaaagggggatgtgct
gcaaggcgattaagttgggtaacgccagggttttcccagtcacgacgttgtaaaacgacggccagtgagcgcgcgtaatacgactcactatagggcgaattggGGTG
5’ arm
AGCTGTGCCCAGAACTCTCACAGCAATCTTTCCCTGTATCACCTGCACTCGCCTCCCCACCCCCAACCCC
GTGTGCACATTCTCACGATCCCCCTGGAGCACCCAGTAGGCTGCAGACCTCCTACCAGAAAATGAACCT
GGGCCAGTGAGGGCCCAGGAAACTATCCTTGGTGAGCACTGATGAGCAAGGACCAGAACCTTGTCTGT
TCTTTTCTTTCCTTTTCTTTCTTTCTGTTTGTTTGATTGATTGATTATTTATTTATTTATTTATTTATT
TATTTATTTATTTATTTTTGAGAGAGGGTCTTACTGTGTAGCCTTGTCTGGCCTGGAACTCAATGTGTAG
ACCAGGCTAGCCTTGACCTCCCAGAGGTCCATCTGCCTCTAACTGTAGAATGCTAGGATTAAAGGCAGG
GGACATGATGCCCATCTGAACTGCAGCTCCTTCCTTCCTGCCTGCCTGCCTGCCTGCCTTTTCCTTCCCCT
TCCCCTTCCCCTTCCCTCCTCTCCCCTGCCCCTCATCTCTTCTCCTTTCTCTTCCCTTTCGACTTCTTTATCT
CCTGCTTCCCTTCTCCTCTTGTTCTTGTCTTGCTATTTTTATTTCTCCAGCCCCCACCTTCTTCTACCTCAC
AGGCTACCTGTGTTCAGGCTCCCTCTCCCCCTCCTACCCCGAACAACCAAGCATAGTGTTGTTCGTAATG
CCGCTGCTTCCTTTCCTGTAGGGGCGAAGACTGGAGCC**AGGCCTGGGGGGACCCCAGGGGCTCCTG**
CTGGTCAGCCAGCGGCAGAAGGAGAGAGTGTATTTTCTAAGATCCTCCCTGGTGGGGCAGCAGA
GCAAGCCGGAAAGCTGACAGAAGGTATTACTGCCTGGGGTCAGGTCCACACTGGGTGGATGCCTCTC
TGGTAGAGGCTGGGGACCGGGTGGTTCCAGGTCCAGTGACCAGGACACAGACCACCTGCTGGTACCCA
PAM1
GGGTTTCTCTGTACTCCTTATCCCTTGAGCCTTACCGTGACCTATGGCCCTGACTAGGAGTCTGACCCAG
p2A
TTTCTGACATCTCTTTCTGTTTTCTATGTCACA**GCTGTCTCTGCTTTTGGCAAAAAATTTTCC**GGATCC
NpHR
GGAGCTACTAATTTCTCCTTGCTTAAGCAAGCTGGTGATGTTGAAGAAAATCCTGGTCCTATGACAGAG
ACCCTGCCTCCCGTGACCGAGAGTGCCGTGGCCCTTCAAGCCGAGGTTACCCAAAGGGAGTTGTTCGAG
TTCGTGCTGAACGACCCTTTGCTTGCAAGCAGTCTCTATATCAACATCGCACTTGCAGGACTGAGTATA
CTGCTGTTCGTTTTTATGACCCGAGGACTCGATGATCCACGGGCAAAACTTATTGCTGTGTCAACCATCC
TTGTGCCTGTCGTCAGCATTGCCTCCTACACTGGATTGGCGAGCGGCCTGACAATTTCCGTTCTTGAAAT
GCCAGCGGGCCATTTTGCAGAAGGCAGCTCAGTGATGCTGGGAGGAGAAGAGGTAGATGGTGTAGTCA
CCATGTGGGGACGGTATCTCACCTGGGCACTTTCCACGCCCATGATTCTCCTCGCTCTGGGTCTCCTGGC
CGGAAGCAATGCTACAAAGCTCTTCACAGCTATCACTTTCGATATCGCTATGTGCGTGACTGGCCTTGC
CGCGGCCCTGACTACCTCCTCCCACCTCATGAGATGGTTCTGGTACGCTATCAGTTGTGCATGCTTTCTG
GTGGTCTTGTATATCCTGCTGGTGGAGTGGGCACAGGACGCCAAAGCCGCGGGAACCGCTGACATGTTC
AATACCCTGAAGCTGTTGACAGTAGTGATGTGGCTGGGGTATCCAATTGTGTGGGCTCTTGGAGTCGAG
GGTATCGCGGTGTTGCCCGTTGGGGTGACGAGCTGGGGATATTCTTTCCTGGATATCGTGGCAAAGTAC
ATTTTCGCATTCTTGCTCCTGAACTATCTGACGTCAAACGAATCTGTCGTGTCCGGCAGCATTTTGGATG
Linker-TS
TTCCATCTGCTTCTGGGACCCCGGCTGATGATGCGGCCGCCAAGAGCAGGATCACCAGCGAGGGCGAG
EYFP
TACATCCCCCTGGACCAGATCGACATCAACGTGGTGAGCAAGGGCGAGGAGCTGTTCACCGGGGTGGT
GCCCATCCTGGTCGAGCTGGACGGCGACGTAAACGGCCACAAGTTCAGCGTGTCCGGCGAGGGCGAGG
GCGATGCCACCTACGGCAAGCTGACCCTGAAGTTCATCTGCACCACCGGCAAGCTGCCCGTGCCCTGGC
CCACCCTCGTGACCACCTTCGGCTACGGCCTGCAGTGCTTCGCCCGCTACCCCGACCACATGAAGCAGC

ACGACTTCTTCAAGTCCGCCATGCCCGAAGGCTACGTCCAGGAGCGCACCATCTTCTTCAAGGACGACG

WGA

GCAACTACAAGACCCGCGCCGAGGTGAAGTTCGAGGGCGACACCCTGGTGAACCGCATCGAGCTGAAG

GGCATCGACTTCAAGGAGGACGGCAACATCCTGGGGCACAAGCTGGAGTACAACTACAACAGCCACAA

CGTCTATATCATGGCCGACAAGCAGAAGAACGGCATCAAGGTGAACTTCAAGATCCGCCACAACATCG

AGGACGGCAGCGTGCAGCTCGCCGACCACTACCAGCAGAACACCCCCATCGGCGACGGCCCCGTGCTG

CTGCCCGACAACCACTACCTGAGCTACCAGTCCGCCCTGAGCAAAGACCCCAACGAGAAGCGCGATCA

CATGGTCCTGCTGGAGTTCGTGACCGCCGCCGGGATCACTCTCGGCATGGACGAGCTGTACAAGTTCTG

linker p2A

CTACGAGAACGAGGTGGGCTCCGGAGCCACGAACTTCTCTCTGTTAAAGCAAGCAGGAGACGTGGAAG

chR2-mCherry

AAAACCCCGGTCCCATGGACTATGGCGGCGCTTTGTCTGCCGTCGGACGCGAACTTTTGTTCGTTACTA

ATCCTGTGGTGGTGAACGGGTCCGTCCTGGTCCCTGAGGATCAATGTTACTGTGCCGGATGGATTGAAT

CTCGCGGCACGAACGGCGCTCAGACCGCGTCAAATGTCCTGCAGTGGCTTGCAGCAGGATTCAGCATTT

TGCTGCTGATGTTCTATGCCTACCAAACCTGGAAATCTACATGCGGCTGGGAGGAGATCTATGTGTGCG

CCATTGAAATGGTTAAGGTGATTCTCGAGTTCTTTTTTGAGTTTAAGAATCCCTCTATGCTCTACCTTGC

CACAGGACACCGGGTGCAGTGGCTGCGCTATGCAGAGTGGCTGCTCACTTGTCCTGTCATCCTTATCCG

CCTGAGCAACCTCACCGGCCTGAGCAACGACTACAGCAGGAGAACCATGGGACTCCTTGTCTCAGACA

TCGGGACTATCGTGTGGGGGGCTACCAGCGCCATGGCAACCGGCTATGTTAAAGTCATCTTCTTTTGTC

TTGGATTGTGCTATGGCGCGAACACATTTTTTCACGCCGCCAAAGCATATATCGAGGGTTATCATACTG

TGCCAAAGGGTCGGTGCCGCCAGGTCGTGACCGGCATGGCATGGCTGTTTTTCGTGAGCTGGGGTATGT

TCCCAATTCTCTTCATTTTGGGGCCCGAAGGTTTTGGCGTCCTGAGCGTCTATGGCTCCACCGTAGGTCA

CACGATTATTGATCTGATGAGTAAAAATTGTTGGGGGTTGTTGGGACACTACCTGCGCGTCCTGATCCA

CGAGCACATATTGATTCACGGAGATATCCGCAAAACCACCAAACTGAACATCGGCGGAACGGAGATCG

AGGTCGAGACTCTCGTCGAAGACGAAGCCGAGGCCGGAGCCGTGCCAGCGGCCGCCGTGAGCAAGGG

CGAGGAGGATAACATGGCCATCATCAAGGAGTTCATGCGCTTCAAGGTGCACATGGAGGGCTCCGTGA

ACGGCCACGAGTTCGAGATCGAGGGCGAGGGCGAGGGCCGCCCCTACGAGGGCACCCAGACCGCCAA

GCTGAAGGTGACCAAGGGTGGCCCCCTGCCCTTCGCCTGGGACATCCTGTCCCCTCAGTTCATGTACGG

CTCCAAGGCCTACGTGAAGCACCCCGCCGACATCCCCGACTACTTGAAGCTGTCCTTCCCCGAGGGCTT

CAAGTGGGAGCGCGTGATGAACTTCGAGGACGGCGGCGTGGTGACCGTGACCCAGGACTCCTCCCTGC

AGGACGGCGAGTTCATCTACAAGGTGAAGCTGCGCGGCACCAACTTCCCCTCCGACGGCCCCGTAATG

CAGAAGAAGACCATGGGCTGGGAGGCCTCCTCCGAGCGGATGTACCCCGAGGACGGCGCCCTGAAGGG

CGAGATCAAGCAGAGGCTGAAGCTGAAGGACGGCGGCCACTACGACGCTGAGGTCAAGACCACCTAC

AAGGCCAAGAAGCCCGTGCAGCTGCCCGGCGCCTACAACGTCAACATCAAGTTGGACATCACCTCCCA

CAACGAGGACTACACCATCGTGGAACAGTACGAACGCGCCGAGGGCCGCCACTCCACCGGCGGCATGG

IRES

ACGAGCTGTACAAGTAATGAGAATTCGCGCCCCCCCCTAACGTTACTGGCCGAAGCCGCTTGGAATAA

GGCCGGTGTGCGTTTGTCTATATGTTATTTTCCACCATATTGCCGTCTTTTGGCAATGTGAGGGCCCGGA

AACCTGGCCCTGTCTTCTTGACGAGCATTCCTAGGGGTCTTTCCCCTCTCGCCAAAGGAATGCAAGGTCT

GTTGAATGTCGTGAAGGAAGCAGTTCCTCTGGAAGCTTCTTGAAGACAAACAACGTCTGTAGCGACCCT

TTGCAGGCAGCGGAACCCCCCACCTGGCGACAGGTGCCTCTGCGGCCAAAAGCCACGTGTATAAGATA

CACCTGCAAAGGCGGCACAACCCCAGTGCCACGTTGTGAGTTGGATAGTTGTGGAAAGAGTCAAATGG

CTCTCCTCAAGCGTATTCAACAAGGGGCTGAAGGATGCCCAGAAGGTACCCCATTGTATGGGATCTGAT

CTGGGGCCTCGGTGCACATGCTTTACATGTGTTTAGTCGAGGTTAAAAAACGTCTAGGCCCCCCGAACC

ACGGGGACGTGGTTTCCTTTGAAAAACACGATGATAATATGGCCACAACCATGAGGAAGATGATGTC

TACAATGGCATTGACCCTGGGAGCTGCCGTGTTTCTCGCGTTCGCCGCCGCTACGGCACAGGCCCAGCG
ATGCGGGGAACAAGGGAGCAATATGGAGTGTCCCAATAATCTTTGTTGCAGTCAGTATGGCTACTGCG
GGATGGGTGGTGACTACTGTGGCAAGGGATGTCAGAACGGCGCTTGCTGGACATCTAAACGCTGTGGC
TCTCAGGCCGGAGGAGCTACCTGCCCAAACAACCATTGCTGCTCACAGTATGGTCACTGTGGCTTTGGA
GCAGAATATTGCGGTGCTGGCTGTCAGGGCGGGCCGTGTAGAGCAGACATCAAGTGTGGAAGCCAATC
AGGTGGGAAACTGTGTCCTAACAATCTGTGCTGCTCCCAGTGGGGTTTCTGTGGCCTGGGGAGCGAGTT
CTGCGGTGGGGGCTGCCAGAGTGGGGCCTGTAGCACCGATAAACCATGCGGAAAGGACGCCGGAGGG
CGGGTCTGCACTAACAATTACTGCTGTTCCAAATGGGGTTCCTGCGGGATTGGCCCCGGATATTGCGGC
GCTGGATGCCAAAGTGGCGGTTGTGATATGCCCAAGAAGAAGAGGAAGGTGTCCAATCTCCTGACTGT
Cre
TCACCAGAACCTCCCTGCGCTGCCAGTAGATGCCACTAGCGATGAGGTCAGGAAAAATCTCATGGATAT
GTTTAGGGATAGACAGGCGTTTTCTGAACACACCTGGAAAATGCTGCTTAGCGTGTGCCGATCCTGGGC
AGCCTGGTGTAAGCTGAACAATCGCAAATGGTTCCCCGCCGAGCCGGAGGACGTGCGCGATTACCTGC
TGTATCTCCAGGCAAGAGGGCTGGCTGTCAAGACTATCCAGCAGCACTTGGGCCAACTGAATATGCTGC
ATCGACGCAGCGGGCTCCCCCGGCCTAGCGATTCAAACGCAGTCTCCCTTGTTATGAGGAGAATTAGAA
AGGAAAACGTAGATGCGGGTGAGAGGGCTAAGCAGGCTCTCGCTTTTGAGCGGACTGATTTCGACCAG
GTCAGATCCCTGATGGAGAACAGCGATCGGTGCCAGGACATCAGGAACCTCGCATTTCTGGGAATTGC
ATATAACACACTTCTGCGCATAGCTGAGATCGCCCGGATCAGAGTGAAAGACATCAGTCGAACGGACG
GCGGCCGGATGCTTATTCATATTGGACGCACAAAGACATTGGTCAGCACCGCTGGCGTTGAAAAGGCCT
TGTCCCTGGGCGTAACGAAGCTGGTGGAAAGATGGATCTCAGTGTCCGGCGTGGCTGACGACCCTAAT
AATTACTTGTTCTGTCGAGTGAGAAAAAACGGAGTCGCCGCGCCCTCTGCCACCAGCCAATTGAGTACA
CGGGCCCTTGAAGGGATCTTTGAGGCAACCCACCGACTCATATACGGAGCCAAGGATGACAGTGGCCA
GAGGTATCTCGCCTGGTCAGGTCATTCTGCTAGGGTGGGGGCCGCACGAGACATGGCGCGGGCAGGAG
TCTCCATACCAGAGATTATGCAAGCTGGAGGTTGGACAAATGTGAACATCGTTATGAACTATATCCGCA
ATCTTGACTCTGAAACCGGGGCCATGGTGAGACTGCTCGAAGATGGTGACTGATTCTGGTGATTGTGTC
Stop PAM2
CAGAAGGGTGAGTACCTGTGCCTGCCATATTGGGAGAGGCCTGACTTACTCGTAGTTGGTGGAAGTCCT
3' arm
CTGGGAAGGGTCCAGTCTTCAGTAGCTGGCACAGTGAGAGCTACAGCGTATTCTCCTGGCTGTGTGAGA
GAAAGAGCGAGGGCACTCCACAGAGCCTAAGACATGGGCCTGCTCAGGAGAGCAGTCACTGTGTAGAC
CAGGCCAGCTTTGAACTCACAGAGATCCAGGCATGGGCCACATTGTCCCCAGTAAAAGAAAGCCAGGC
AGGGCTGGTGCCCCTCTTGCCTCTCTCTCCACCCCAGTCTTCCGCCATGCTCTCAAAGTGATAGGGCATC
TTGGCTTAGAGGCAGGGGCAGTGCTGCAGCGACTCATGCCCCTTCTGCAACGCTCTGCAGGCTCCTGAA
GACCTGAAGAAAGACAATCTCATTGCTGTGGAAAAATCTCTGGAGTCAGAGGCCTGGGTGCAGCTCTG
GACTCTGCGTATAACGGTGAGTGTCAGGACCAGGTTAAGATGAACCAGAGACGTGGGTGAAGGGGTCC
TGTTCCTGGAAGTGTGAACTAAAAGGATGCTTCTTTCAGTGGGATGGGCCTAGCAGCCCCCCTCAGAGG
CAGTGTGCTGGCTGGCATGACGGTAAATGAGTCAGCTATCCTTGGGAAAGGGCCCAGTGAAGCCAATT
GTACCCTTACAACTTCAGGTCTGTATTCTGGACACTGAGTTCAAAGTCATCCTGTCTCTAGGACTGTGGC
GCTCTTTGGAGACATTGGTGTCTGGTGCCGGTGTTTCCAAACCCAGCAGAACGTAGAGCTTTGGTCATG
AAGTGAAGAGAGTCTTGGATTCACAGGTTGTGAATCCAAAAGGCAAAAGGGTGAACTAGGGAGAGAG
GCATACAGAAAGGGGACACAGGAAAAGCAGTCACTGGTCAGCTTGTCTCAGCCCATTTCAGGGCAGCA
CGGTGAGAGGACAGGACGGTACATGATAGGACCCTTCTCTGCGTAGATTGTAGTGGCCAAAGAAGATG
AGATGCTCCCTCTCGGGGCCCACCCTGTCCTGGCCTTGACCTTGCCTCTGTGGACAGAAGAGGGCACAC
CTCATGTAGAATTGGGGAGAAGCCTAGTTGTTTAAGCTGTCCCTTGCACcagcttttgttccctttagtgagggttaattgcgc

gcttggcgtaatcatggtcatagctgtttcctgtgtgaaattgttatccgctcacaattccacacaacatacgagccggaagcataaagtgtaaagcctggggtgcctaatgag
tgagctaactcacattaattgcgttgcgctcactgcccgctttccagtcgggaaacctgtcgtgccagctgcattaatgaatcggccaacgcgcggggagaggcggtttgc
gtattgggcgctcttccgcttcctcgctcactgactcgctgcgctcggtcgttcggctgcggcgagcggtatcagctcactcaaaggcggtaatacggttatccacagaatc
aggggataacgcaggaaagaacatgtgagcaaaaggccagcaaaaggccaggaaccgtaaaaaggccgcgttgctggcgtttttccataggctccgcccccctgacg
agcatcacaaaaatcgacgctcaagtcagaggtggcgaaacccgacaggactataaagataccaggcgtttccccctggaagctccctcgtgcgctctcctgttccgacc
ctgccgcttaccggatacctgtccgcctttctcccttcgggaagcgtggcgctttctcatagctcacgctgtaggtatctcagttcggtgtaggtcgttcgctccaagctgggct
gtgtgcacgaaccccccgttcagcccgaccgctgcgccttatccggtaactatcgtcttgagtccaacccggtaagacacgacttatcgccactggcagcagccactggta
acaggattagcagagcgaggtatgtaggcggtgctacagagttcttgaagtggtggcctaactacggctacactagaaggacagtatttggtatctgcgctctgctgaagcc
agttaccttcggaaaaagagttggtagctcttgatccggcaaacaaaccaccgctggtagcggtggtttttttgtttgcaagcagcagattacgcgcagaaaaaaaggatctc
aagaagatcctttgatcttttctacggggtctgacgctcagtggaacgaaaactcacgttaagggattttggtcatgagattatcaaaaaggatcttcacctagatccttttaaatt
aaaaatgaagttttaaatcaatctaaagtatatatgagtaaacttggtctgacagttaccaatgcttaatcagtgaggcacctatctcagcgatctgtctatttcgttcatccatagt
tgcctgactccccgtcgtgtagataactacgatacgggagggcttaccatctggccccagtgctgcaatgataccgcgagacccacgctcaccggctccagatttatcagc
aataaaccagccagccggaagggccgagcgcagaagtggtcctgcaactttatccgcctccatccagtctattaattgttgccgggaagctagagtaagtagttcgccagtt
aatagtttgcgcaacgttgttgccattgctacaggcatcgtggtgtcacgctcgtcgtttggtatggcttcattcagctccggttcccaacgatcaaggcgagttacatgatccc
ccatgttgtgcaaaaaagcggttagctccttcggtcctccgatcgttgtcagaagtaagttggccgcagtgttatcactcatggttatggcagcactgcataattctcttactgtc
atgccatccgtaagatgcttttctgtgactggtgagtactcaaccaagtcattctgagaatagtgtatgcggcgaccgagttgctcttgcccggcgtcaatacgggataatacc
gcgccacatagcagaactttaaaagtgctcatcattggaaaacgttcttcggggcgaaaactctcaaggatcttaccgctgttgagatccagttcgatgtaacccactcgtgc
acccaactgatcttcagcatcttttactttcaccagcgtttctgggtgagcaaaaacaggaaggcaaaatgccgcaaaaaagggaataagggcgacacggaaatgttgaat
Plasmid backbone
actcatactcttcctttttcaatattattgaagcatttatcagggttattgtctcatgagcggatacatatttgaatgtatttagaaaaataaacaaataggggttccgcgcacatttcc
ccgaaaagtgccac-3’

Supplementary Text S11 Sequence of rat *Bassoon* targeting construct p2A-NpHR-EYFP-p2A-ChR2-mCherry-IRES-WGA-Cre donor plasmid (*Bassoon*-5.5 kb)

Supplementary Text S12

5'...ctaaattgtaagcgttaatattttgttaaaattcgcgttaaatttttgttaaatcagctcattttttaaccaataggccgaaatcggcaaaatcccttataaatcaaaagaatag
Plasmid backbone
accgagatagggttgagtgttgttccagtttggaacaagagtccactattaaagaacgtggactccaacgtcaaagggcgaaaaaccgtctatcagggcgatggcccacta
cgtgaaccatcaccctaatcaagttttttggggtcgaggtgccgtaaagcactaaatcggaaccctaaagggagcccccgatttagagcttgacggggaaagccggcgaa
cgtggcgagaaaggaagggaagaaagcgaaaggagcgggcgctagggcgctggcaagtgtagcggtcacgctgcgcgtaaccaccacacccgccgcgcttaatgc
gccgctacagggcgcgtcccattcgccattcaggctgcgcaactgttgggaagggcgatcggtgcgggcctcttcgctattacgccagctggcgaaagggggatgtgct
gcaaggcgattaagttgggtaacgccagggttttcccagtcacgacgttgtaaaacgacggccagtgagcgcgcgtaatacgactcactatagggcgaattggTGTG
5' arm
CAGGATACCCGCTTTAGGTGGGACAAGACATTCTATTCGCCACAGTCCCCATCACTCCCTATCGCCTCCT
GACCCAGAGGCAAAGGGTCAGTTGTCATTTAGCATCTTATACCCAGCTCAGCCCCCGTTGTTTATGTGC
CTGCCTGGTACCTGGGGAGATCCGGATTTGAGACTCTGCTCAGAGCGTATCTGGTTCTATAATCCTCTA
GGCTTATTCCATGTGGCTAATAGTCATGATTCTAACATTCCATTTTCCCTTGTTTGATGAATTCTGCATGT
CTGAACTCCACTGTATCTAAGCTGGGATGTGATGTCCCCATCATCTAGAAGTTCTACCTTGATGCCAGGT
AGCTGAGCCCTGCCACAGAGGCCCCCTCCCTACAAATGGGGCAGACAGTGTGCTTCCATTGAGAGGGC
TGCTGGGAGTGGAGGAAGTGAAGAAAGTGAGACAGGAGGCCCCCGGTTGCATCTCCTCTGCCCCTCCC
ATCCCCGTCTCCAGTGTGGTCCCGGAACAGCAAGCCCGTGCACACCACCATCCTGAATCCCCACATCCA
CCTGATGGGCGACGAGTCAGCCTGCATCGCCTACATCCGCATCACGCAGTACCTGGACGCCGGCGGCAT
CCCACGCACCGCCCAGTCGGAGGAGACCCGTGTCTGGCACCGCCGGGACGGCAAATGGCAGATCGTCC
ACTTCCACAGATCTGGGGCGCCCTCCGTCCTGCCCCAGTAAGAATCCCTCCTTCCCACCTCCCAGGGTA
ATGTAAAGGATGGGAGTGTTGCCCTGGAGAGGGTTACGTCAGGGAAGCTGCTGCCTTCAGCTCTGAAA
GGCTGCAGAGTGTAGTGGGAAGACCCCAAGAAGAGGGGGCAAGGCCTGCAGGTCTGAGGGAAGAGCT
GGGCAGGAGCCACCGGGGACCGCTTGGACTCCATCTAAAGCGGCAGGGTGGGTGGGTTGCCCAAG
PAM1 CCT-CaT p2A-ChR2::EYFP
CAGGAAACACCATGCCaTGAGtAcGtgctCGaGTGaGaCTTACAGCGGATCCGGAGCTACTAATTTCTCCTTG
CTTAAGCAAGCTGGTGATGTTGAAGAAAATCCTGGTCCTATGGACTATGGCGGCGCTTTGTCTGCCGTC
GGACGCGAACTTTTGTTCGTTACTAATCCTGTGGTGGTGAACGGGTCCGTCCTGGTCCCTGAGGATCAA
TGTTACTGTGCCGGATGGATTGAATCTCGCGGCACGAACGGCGCTCAGACCGCGTCAAATGTCCTGCAG
TGGCTTGCAGCAGGATTCAGCATTTTGCTGCTGATGTTCTATGCCTACCAAACCTGGAAATCTACATGC
GGCTGGGAGGAGATCTATGTGTGCGCCATTGAAATGGTTAAGGTGATTCTCGAGTTCTTTTTTGAGTTTA
AGAATCCCTCTATGCTCTACCTTGCCACAGGACACCGGGTGCAGTGGCTGCGCTATGCAGAGTGGCTGC
TCACTTGTCCTGTCATCCTTATCCGCCTGAGCAACCTCACCGGCCTGAGCAACGACTACAGCAGGAGAA
CCATGGGACTCCTTGTCTCAGACATCGGGACTATCGTGTGGGGGGCTACCAGCGCCATGGCAACCGGCT
ATGTTAAAGTCATCTTCTTTTGTCTTGGATTGTGCTATGGCGCGAACACATTTTTTCACGCCGCCAAAGC
ATATATCGAGGGTTATCATACTGTGCCAAAGGGTCGGTGCCGCCAGGTCGTGACCGGCATGGCATGGCT
GTTTTTCGTGAGCTGGGGTATGTTCCCAATTCTCTTCATTTTGGGGCCCGAAGGTTTTGGCGTCCTGAGC
GTCTATGGCTCCACCGTAGGTCACACGATTATTGATCTGATGAGTAAAAATTGTTGGGGGTTGTTGGGA
CACTACCTGCGCGTCCTGATCCACGAGCACATATTGATTCACGGAGATATCCGCAAAACCACCAAACTG
AACATCGGCGGAACGGAGATCGAGGTCGAGACTCTCGTCGAAGACGAAGCCGAGGCCGGAGCCGTGC
CAGCGGCCGCCACCATGGTGAGCAAGGGCGAGGAGCTGTTCACCGGGGTGGTGCCCATCCTGGTCGAG
CTGGACGGCGACGTAAACGGCCACAAGTTCAGCGTGTCCGGCGAGGGCGAGGGCGATGCCACCTACGG
CAAGCTGACCCTGAAGTTCATCTGCACCACCGGCAAGCTGCCCGTGCCCTGGCCCACCCTCGTGACCAC
CTTCGGCTACGGCCTGCAGTGCTTCGCCCGCTACCCCGACCACATGAAGCAGCACGACTTCTTCAAGTC
CGCCATGCCCGAAGGCTACGTCCAGGAGCGCACCATCTTCTTCAAGGACGACGGCAACTACAAGACCC

GCGCCGAGGTGAAGTTCGAGGGCGACACCCTGGTGAACCGCATCGAGCTGAAGGGCATCGACTTCAAG
GAGGACGGCAACATCCTGGGGCACAAGCTGGAGTACAACTACAACAGCCACAACGTCTATATCATGGC
CGACAAGCAGAAGAACGGCATCAAGGTGAACTTCAAGATCCGCCACAACATCGAGGACGGCAGCGTG
CAGCTCGCCGACCACTACCAGCAGAACACCCCCATCGGCGACGGCCCCGTGCTGCTGCCCGACAACCA
CTACCTGAGCTACCAGTCCGCCCTGAGCAAAGACCCCAACGAGAAGCGCGATCACATGGTCCTGCTGG
Stop
AGTTCGTGACCGCCGCCGGGATCACTCTCGGCATGGACGAGCTGTACAAGTAATGAGGGACCAGGCCA
3’ arm PAM2
GGGTCACTGCGTTGCTGTGCCGCAGAGATCCACTCTGTCCGTGCAATGGAGCTGCTGGTTCTCCCAGGT
GGATTTTGCTGGAATTCTCCCATCTCATCATCCCACCACCGTCACTTCTGTACCTGCATCAAGAAAACTT
GCTTGTTCACAAAAGTCATCGCAACTTCAGAGCGAATAGCCACATCTCCCCACCTCTCACCCCCACCCT
CTCTCCTGCCAGGCTGGGGCTTCCTCAGGCATGGGTGCCCACGGCACTGGCCCCCTCTCCCCAGCCTCG
GCTGCTGTCCGCCTGGCCTGTCTTGGGCTGTAGGCTAGAATGCCTGGGCTGTGTGCCCACTAGTGGCTG
GGGAGAAGGAGGGGTGGCATGGTGAGGAAGGCAGCATCTGCCCATCCCTCTCCCAGACCTTTCCTATTC
CAGTGTCCCCAGGGAAGGGCAGATGACACTCCCTTCCCTCAAGCCAACCGCCCTGAAGGAGGGGGGAG
AAGAGCATATGCCAGGAGCCTCCTGCCTCAAAGTGCTCCCCTAAGTCTTCTCTTTCCTGTGCTGACCTCA
GGGTGGTCTGACCCTTCCCTCAGTGTGGGGGACGTGGCCCTCTCAGGTGCCCCTACTTGCTTTCTGCTTC
CTTCTGGTGAAGTCCACCTCCAACATTAACCCGCCCACCCCGCTCCACCGTTAACCCTGGAGAATTCCA
GCTTCGTCATATCTCAGAGAGGGAATCTAATTGTTTTTGGGGGGCAAACGAAAGCAACGTTTAGTATCA
CTTTCTACTTGGACCGCATGCCTTTTTATAGCCAAATTTCTGTGTATTTCGTAAATGGATTTCGCGTTAAT
GGATATTTATGTAATAACTAAACTTCTCAGATTATCGTGAGAAAGGGTCAGGTTGGAAGGGGTGTAGG
AAGGGGGTGAGGGGTAGTTTTTTTCTGTTCTAGTTTTTTTTTTTTTTTCTTTGTCATCTCTGAGGTGGAC
CTTGTCACCTGTGGTTATTGGGGCcagcttttgttccctttagtgagggttaattgcgcgcttggcgtaatcatggtcatagctgtttcctgtgtgaaattgt
tatccgctcacaattccacacaacatacgagccggaagcataaagtgtaaagcctggggtgcctaatgagtgagctaactcacattaattgcgttgcgctcactgcccgcttt
ccagtcgggaaacctgtcgtgccagctgcattaatgaatcggccaacgcgcggggagaggcggtttgcgtattgggcgctcttccgcttcctcgctcactgactcgctgcg
ctcggtcgttcggctgcggcgagcggtatcagctcactcaaaggcggtaatacggttatccacagaatcaggggataacgcaggaaagaacatgtgagcaaaaggcca
gcaaaaggccaggaaccgtaaaaaggccgcgttgctggcgtttttccataggctccgcccccctgacgagcatcacaaaaatcgacgctcaagtcagaggtggcgaaac
ccgacaggactataaagataccaggcgtttccccctggaagctccctcgtgcgctctcctgttccgaccctgccgcttaccggatacctgtccgcctttctcccttcgggaag
cgtggcgctttctcatagctcacgctgtaggtatctcagttcggtgtaggtcgttcgctccaagctgggctgtgtgcacgaaccccccgttcagcccgaccgctgcgccttat
ccggtaactatcgtcttgagtccaacccggtaagacacgacttatcgccactggcagcagccactggtaacaggattagcagagcgaggtatgtaggcggtgctacagag
ttcttgaagtggtggcctaactacggctacactagaaggacagtatttggtatctgcgctctgctgaagccagttaccttcggaaaaagagttggtagctcttgatccggcaaa
caaaccaccgctggtagcggtggtttttttgtttgcaagcagcagattacgcgcagaaaaaaaggatctcaagaagatcctttgatcttttctacggggtctgacgctcagtgg
aacgaaaactcacgttaagggattttggtcatgagattatcaaaaaggatcttcacctagatccttttaaattaaaaatgaagttttaaatcaatctaaagtatatatgagtaaactt
ggtctgacagttaccaatgcttaatcagtgaggcacctatctcagcgatctgtctatttcgttcatccatagttgcctgactccccgtcgtgtagataactacgatacgggaggg
cttaccatctggccccagtgctgcaatgataccgcgagacccacgctcaccggctccagatttatcagcaataaaccagccagccggaagggccgagcgcagaagtggt
cctgcaactttatccgcctccatccagtctattaattgttgccgggaagctagagtaagtagttcgccagttaatagtttgcgcaacgttgttgccattgctacaggcatcgtggt
gtcacgctcgtcgtttggtatggcttcattcagctccggttcccaacgatcaaggcgagttacatgatcccccatgttgtgcaaaaaagcggttagctccttcggtcctccgat
cgttgtcagaagtaagttggccgcagtgttatcactcatggttatggcagcactgcataattctcttactgtcatgccatccgtaagatgcttttctgtgactggtgagtactcaac
caagtcattctgagaatagtgtatgcggcgaccgagttgctcttgcccggcgtcaatacgggataataccgcgccacatagcagaactttaaaagtgctcatcattggaaaa
cgttcttcggggcgaaaactctcaaggatcttaccgctgttgagatccagttcgatgtaacccactcgtgcacccaactgatcttcagcatcttttactttcaccagcgtttctgg
gtgagcaaaaacaggaaggcaaaatgccgcaaaaaagggaataagggcgacacggaaatgttgaatactcatactcttcctttttcaatattattgaagcatttatcagggtt
Plasmid backbone
attgtctcatgagcggatacatatttgaatgtatttagaaaaataaacaaataggggttccgcgcacatttccccgaaaagtgccac-3’

Supplementary Text S12 Sequence of monkey *CAMK2A* targeting construct p2A-ChR2::EYFP donor plasmid.

Supplementary Text S13

5’...ctaaattgtaagcgttaatattttgttaaaattcgcgttaaatttttgttaaatcagctcattttttaaccaataggccgaaatcggcaaaatcccttataaatcaaaagaatag
Plasmid backbone
accgagatagggttgagtgttgttccagtttggaacaagagtccactattaaagaacgtggactccaacgtcaaagggcgaaaaaccgtctatcagggcgatggcccacta
cgtgaaccatcaccctaatcaagttttttggggtcgaggtgccgtaaagcactaaatcggaaccctaaagggagcccccgatttagagcttgacggggaaagccggcgaa
cgtggcgagaaaggaagggaagaaagcgaaaggagcgggcgctagggcgctggcaagtgtagcggtcacgctgcgcgtaaccaccacacccgccgcgcttaatgc
gccgctacagggcgcgtcccattcgccattcaggctgcgcaactgttgggaagggcgatcggtgcgggcctcttcgctattacgccagctggcgaaagggggatgtgct
gcaaggcgattaagttgggtaacgccagggttttcccagtcacgacgttgtaaaacgacggccagtgagcgcgcgtaatacgactcactatagggcgaattggGATG
5’ arm
GGGTGGCTGGATTTGGCCAGTATCTGGATGGGAATGCCTAGGGTTCTGGATGGGTCAGGGGGAGGGCA
TCAGGGAGCAGCTGGCCAGTGTGCTTATGGCTGTTGATGCATTGAGGGATAGCTCCACACACACATTCA
ATAAATTTGAGGAGCTGAGAGGGTGACTGGCTCCCCGTGAAGGCACAGTGCCAGAGGTCTTTGGAGAG
GGGGTGAAGCACCTGGGTTCCTGAAGAACATGGTGGTGTAGGAGTGATTCCAGACAGCTGGGATGTGC
AGAGCCCGAGAGAGTGCCAGGGCGCAGGCTGGGAGTTGAAAGTTGGGTGTGGTGGCTCACGCCTTTAA
TCCTGACATGGGAGGCAGAGGCGGGAGGATCGCTTGAGGACAGGAATTCAAGACCAGCCTGGGTAACA
TAGCAAGACCCCATCTCTACTAAAAATAAAATCAACAGGTCGAGGTTGTCCAAGCCTGTAGTCCCAGCC
ACTCAGGAGGCTGGGGCAGAAGGATTGCTTCGGCCCAGTAGATCGAGGCTGCAATGAGCCAGCATTGT
ACCCCACTGCACTCCAGTCTGGGCAACAAAATAAGACCCTGTCTTAAAAAAAAAAGGTTTCGGTGGGG
GACCTGTGCCGAGGTCCTGGAGGGGCGCCCAGTTGTGTCTCCGGTTGTCCCCTTCCACAGACACCACTG
CCACCACCATTAGGCAAACATCCTTCGCCTCAGTTTCTCCCCCCCACCTCCCTCTCCTCCACCCATCCAG
GGGGCGGGGCCAGAGGTCAAGGCTAGTGGGTGGGACTGGGGAGGGAGAGAGGGGTTGAGTAGTCCCT
PAM1 EGFP
TAGCAAGCCCTCATTTCACCAGGCCCCCGGCTTGGGGTGCCTTCCTTCCCCATGGTGAGCAAGGGCGAG
GAGCTGTTCACCGGGGTGGTGCCCATCCTGGTCGAGCTGGACGGCGACGTAAACGGCCACAAGTTCAG
CGTGTCCGGCGAGGGCGAGGGCGATGCCACCTACGGCAAGCTGACCCTGAAGTTCATCTGCACCACCG
GCAAGCTGCCCGTGCCCTGGCCCACCCTCGTGACCACCCTGACCTACGGCGTGCAGTGCTTCAGCCGCT
ACCCCGACCACATGAAGCAGCACGACTTCTTCAAGTCCGCCATGCCCGAAGGCTACGTCCAGGAGCGC
ACCATCTTCTTCAAGGACGACGGCAACTACAAGACCCGCGCCGAGGTGAAGTTCGAGGGCGACACCCT
GGTGAACCGCATCGAGCTGAAGGGCATCGACTTCAAGGAGGACGGCAACATCCTGGGGCACAAGCTGG
AGTACAACTACAACAGCCACAACGTCTATATCATGGCCGACAAGCAGAAGAACGGCATCAAGGTGAAC
TTCAAGATCCGCCACAACATCGAGGACGGCAGCGTGCAGCTCGCCGACCACTACCAGCAGAACACCCC
CATCGGCGACGGCCCCGTGCTGCTGCCCGACAACCACTACCTGAGCACCCAGTCCGCCCTGAGCAAAG
ACCCCAACGAGAAGCGCGATCACATGGTCCTGCTGGAGTTCGTGACCGCCGCCGGGATCACTCTCGGC
p2A
ATGGACGAGCTGTACAAGGGATCCGGAGCTACTAATTTCTCCTTGCTTAAGCAAGCTGGTGATGTTGAA
NeoR
GAAAATCCTGGTCCTATGATTGAACAAGATGGATTGCACGCAGGTTCTCCGGCCGCTTGGGTGGAGAG
GCTATTCGGCTATGACTGGGCACAACAGACAATCGGCTGCTCTGATGCCGCCGTGTTCCGGCTGTCAGC
GCAGGGGCGCCCGGTTCTTTTTGTCAAGACCGACCTGTCCGGTGCCCTGAATGAACTGCAAGACGAGGC
AGCGCGGCTATCGTGGCTGGCCACGACGGGCGTTCCTTGCGCAGCTGTGCTCGACGTTGTCACTGAAGC
GGGAAGGGACTGGCTGCTATTGGGCGAAGTGCCGGGGCAGGATCTCCTGTCATCTCACCTTGCTCCTGC
CGAGAAAGTATCCATCATGGCTGATGCAATGCGGCGGCTGCATACGCTTGATCCGGCTACCTGCCCATT
CGACCACCAAGCGAAACATCGCATCGAGCGAGCACGTACTCGGATGGAAGCCGGTCTTGTCGATCAGG
ATGATCTGGACGAAGAGCATCAGGGGCTCGCGCCAGCCGAACTGTTCGCCAGGCTCAAGGCGAGCATG
CCCGACGGCGAGGATCTCGTCGTGACCCATGGCGATGCCTGCTTGCCGAATATCATGGTGGAAAATGGC
CGCTTTTCTGGATTCATCGACTGTGGCCGGCTGGGTGTGGCGGACCGCTATCAGGACATAGCGTTGGCT

ACCCGTGATATTGCTGAAGAGCTTGGCGGCGAATGGGCTGACCGCTTCCTCGTGCTTTACGGTATCGCC
p2A
GCTCCCGATTCGCAGCGCATCGCCTTCTATCGCCTTCTTGACGAGTTCTTCGGCTCCGGAGCCACGAACT
TCTCTCTGTTAAAGCAAGCAGGAGACGTGGAGGAGAACCCCGGACCCATGGCAGGTCATTTAGCAAGT
PAM2 3’ arm
GAATTTGCATTTAGTCCTCCTCCAGGAGGTGGAGGTGATGGGCCAGGGGGGCCGGAGCCGGGCTGGGT
TGATCCTCGGACCTGGCTAAGCTTCCAAGGCCCTCCTGGAGGGCCAGGAATCGGGCCGGGGGTTGGGC
CAGGCTCCGAGGTGTGGGGGATTCCCCCGTGCCCCCCGCCATATGAGTTCTGTGGGGGGATGGCGTACT
GTGGGCCTCAGGTTGGAGTGGGGCTAGTGCCCCAAGGTGGCTTGGAGACCTCTCAGCCTGAGGGCGAA
GCAGGAGCCGGGGTGGAGAGCAACTCCGATGGGGCCTCCCCAGAGCCCTGCACCGTCCCCACTGGTGC
CGTGAAGCTGGAGAAGGAGAAGCTGGAGCAAAACCCGGAGGAGGCAAGTGAGCTTCAATGGGGTTGG
GGTGTGGGGAGGTGGTCATGACAGGGCAGCCTGATGGGGAAGTGGTCACCTGCAGCTGCCCATACCTG
GCACCCAGGAGAGGAGCGGGCAGGGGCAGCTGTCCTGGCCTGGGAGGGGTGTGTATCGACTGTAGGTG
AGGGCAGAGAGGCCTGGGAGAGTCGGAAGTTGGCCCAGGCTGGCCTTTGCTCTGGCCCAGCCCTTGTC
AGGTTCTCTCACATTTCATAGGCCTGCCCAGGGTCAGGTCATTCATTACCGGCCCAGCACCAGACTCAG
CTTGGGGTTGGTTTCAGCTCCTTTTCCCACCCTTAGTCCTGCTTGACCCTTATCAGACCCAAGATCTTGG
CCTTAGGGTTAAGCAGAGCCTGGGGATAAAAACAGTGCTTATCCCAGGATTATTGTTTCTGAAAGTCTA
GGGTGTGGCTCGTTTCTGATAGGGGCTCCTGTTTGGGCTGTGTGTGTGCACGTGTGGAGCTGGGTTTACC
TGCAGTCAAGTATAGGGCTTGTCTTCCCTTGACCTGTGCCTCAGGCCAGTGACTTGCCACTGTGTTAAGG
TGCACACCCTGGCACCCCTTGTAGAGAGCTGGATTTTGATTGACTTCAGCCTCGGTTCCAAAGTTGTAA
ACAAGAAAAATGGTGAGAGATTTCTCCAGGCCATTCCCCAAATGTAGAGCTGCTGAGGGATTGAAGGC
ATCCAGCCCTGCTGAGGACTATTAAAGATGTATCTTCCAGTCCTTCAAGGCcagcttttgttccctttagtgagggttaattg
cgcgcttggcgtaatcatggtcatagctgtttcctgtgtgaaattgttatccgctcacaattccacacaacatacgagccggaagcataaagtgtaaagcctggggtgcctaat
gagtgagctaactcacattaattgcgttgcgctcactgcccgctttccagtcgggaaacctgtcgtgccagctgcattaatgaatcggccaacgcgcggggagaggcggtt
tgcgtattgggcgctcttccgcttcctcgctcactgactcgctgcgctcggtcgttcggctgcggcgagcggtatcagctcactcaaaggcggtaatacggttatccacaga
atcaggggataacgcaggaaagaacatgtgagcaaaaggccagcaaaaggccaggaaccgtaaaaaggccgcgttgctggcgtttttccataggctccgcccccctga
cgagcatcacaaaaatcgacgctcaagtcagaggtggcgaaacccgacaggactataaagataccaggcgtttccccctggaagctccctcgtgcgctctcctgttccga
ccctgccgcttaccggatacctgtccgcctttctcccttcgggaagcgtggcgctttctcatagctcacgctgtaggtatctcagttcggtgtaggtcgttcgctccaagctgg
gctgtgtgcacgaaccccccgttcagcccgaccgctgcgccttatccggtaactatcgtcttgagtccaacccggtaagacacgacttatcgccactggcagcagccactg
gtaacaggattagcagagcgaggtatgtaggcggtgctacagagttcttgaagtggtggcctaactacggctacactagaaggacagtatttggtatctgcgctctgctgaa
gccagttaccttcggaaaaagagttggtagctcttgatccggcaaacaaaccaccgctggtagcggtggtttttttgtttgcaagcagcagattacgcgcagaaaaaaagga
tctcaagaagatcctttgatcttttctacggggtctgacgctcagtggaacgaaaactcacgttaagggattttggtcatgagattatcaaaaaggatcttcacctagatccttttа
aattaaaaatgaagttttaaatcaatctaaagtatatatgagtaaacttggtctgacagttaccaatgcttaatcagtgaggcacctatctcagcgatctgtctatttcgttcatccat
agttgcctgactccccgtcgtgtagataactacgatacgggagggcttaccatctggccccagtgctgcaatgataccgcgagacccacgctcaccggctccagatttatc
agcaataaaccagccagccggaagggccgagcgcagaagtggtcctgcaactttatccgcctccatccagtctattaattgttgccgggaagctagagtaagtagttcgcc
agttaatagtttgcgcaacgttgttgccattgctacaggcatcgtggtgtcacgctcgtcgtttggtatggcttcattcagctccggttcccaacgatcaaggcgagttacatgat
cccccatgttgtgcaaaaaagcggttagctccttcggtcctccgatcgttgtcagaagtaagttggccgcagtgttatcactcatggttatggcagcactgcataattctcttact
gtcatgccatccgtaagatgcttttctgtgactggtgagtactcaaccaagtcattctgagaatagtgtatgcggcgaccgagttgctcttgcccggcgtcaatacgggataat
accgcgccacatagcagaactttaaaagtgctcatcattggaaaacgttcttcggggcgaaaactctcaaggatcttaccgctgttgagatccagttcgatgtaacccactcg
tgcacccaactgatcttcagcatcttttactttcaccagcgtttctgggtgagcaaaaacaggaaggcaaaatgccgcaaaaaagggaataagggcgacacggaaatgttg
aatactcatactcttcctttttcaatattattgaagcatttatcagggttattgtctcatgagcggatacatatttgaatgtatttagaaaataaacaaataggggttccgcgcacatt
Plasmid backbone
tccccgaaaagtgccac-3’

Supplementary Text S13 Sequence of monkey *Oct4* targeting construct EGFP-p2A-NeoR-p2A donor plasmid.

Supplementary Text S14

5'...ttacgccagctggcgaaagggggatgtgctgcaaggcgattaagttgggtaacgccagggttttcccagtcacgacgttgtaaaacgacggccagtgagcgcgcgt

Plasmid backbone   5' arm

aatacgactcactatagggcgaattgGGTACCCCAAATACTCGGTCTTCCGAAAGAACTAACTCAACCTACCCTTCTAC
AAGAGGGTCCGAAAACCACTGTTACGCCCATTGGGTAGCCCCGCCCTTGGGGGGGGCAAAGGGCGTGA
AAGCGGAAGTGACGACACCCGGCGCTCCATTAAATAGCCGTAGACGGAACTTCGCCTTTCTCTCGGCCT
TAGCGCCATTTTTTTGGGTGAGTGTTTTTTGGTTCCTGCGTTGGGATTCCGTGTACAATCCATAGACATC
TGACCTCGGCACTTAGCATCATCACAGCAAACTAACTGTAGCCTTTCTCTCTTTCCCTGTAGAAACCTCT
GCGCCATGAGAGCCAAGGTGAGCGGTTCCTGGTAGTAAGCTTGGGAGGTAGGAGTTGGCGAGTAGTAG
CGAGGAGACGAAGGCAAGTCCGCCATACCTCCTGAACTACTGGGTTTCAAGGGTGCCCAAGAGCTGGT
GGGAGAGAGAAGGTAGTTTGTGAGAGAGCTAGCGGTTAAGTGCTATGGGTAGAGAGGGTGGGCTTAGA
AAAGGGTGGAATTCTGATCTTATGTTGGGAGGGTGTCCAAGTTACTGATGTAGTTGTTACGACCAATCT
TTCATACTTCTTGGTTAAGAATCTGTCCGGTTCTAAAGAGTGCATTTCATATCCTTGCTAAGCCTACTAA
TAAGCTTCATCCCTTTTTTTTTTTTTTTTTTTAATTATCTGCTGCTTGTGATCGGTTGCTAGTGGAGGAA
GAAGCGAATGCGCAGGTACGTTGAGACTTTGCCAGCCCAGGAGGAGGGAAGTTCCTTGGACAAAACTT
AGGAGAAACATTTGGTTTGGAAATCTTAAAAGATCTTTAGGAGAAAAACGTTTGAGTGTTTTCTCCCTG
GAGCCAGGATTTAACAGAACAGAGAACGATAGAACCGTAGTGCTTGTTCATTTTACCACCTCATTCTTT

sgRNA1 target Mutation

ATGTGGACGTTTGATTTAATGTGGGAGGGAAAGGCAACTCTGGTTTGAGGTGTATTCCATTTTTATGTCT

IRES

GCTTTTCAGGCTGAAGCGCAAAAGAAGAAAGATGAGGCAGAGGTCCAAGTAACCCCCCCCTAACGTTA
CTGGCCGAAGCCGCTTGGAATAAGGCCGGTGTGCGTTTGTCTATATGTTATTTTCCACCATATTGCCGTC
TTTTGGCAATGTGAGGGCCCGGAAACCTGGCCCTGTCTTCTTGACGAGCATTCCTAGGGGTCTTTCCCCT
CTCGCCAAAGGAATGCAAGGTCTGTTGAATGTCGTGAAGGAAGCAGTTCCTCTGGATGCTTCTTGAAGA
CAAACAACGTCTGTAGCGACCCTTTGCAGGCAGCGGAA[illegible]CCACCTGGCGACAGGTGCCTCTGCGG (3' arm)
CCAAAAGCCACGTGTATAAGATACACCTGCAAAGGCGGCACAACCCCAGTGCCACGTTGTGAGTTGGA
TAGTTGTGGAAAGAGTCAAATGGCTCTCCTCAAGCGTATTCAACAAGGGGCTGAAGGATGCCCAGAAG
GTACCCCATTGTATGGGATCTGATCTGGGGCCTCGGTGCACATGCTTTACATGTGTTTAGTCGAGGTTAA
AAAACGTCTAGGCCCCCCGAACCACGGGGACGTGGTTTTCCTTTGAAAAACACGATGATAATATGGCC

Mito

ACAACCATGTCCGTCCTGACGCCGCTGCTGCTGCGGGGCTTGACAGGCTCGGCCCGGCGGCTCCCAGTG

EGFP

CCGCGCGCCAAGATCCATTCGTTGGTGAGCAAGGGCGAGGAGCTGTTCACCGGGGTGGTGCCCATCCT
GGTCGAGCTGGACGGCGACGTAAACGGCCACAAGTTCAGCGTGTCCGGCGAGGGCGAGGGCGATGCCA
CCTACGGCAAGCTGACCCTGAAGTTCATCTGCACCACCGGCAAGCTGCCCGTGCCCTGGCCCACCCTCG
TGACCACCCTGACCTACGGCGTGCAGTGCTTCAGCCGCTACCCCGACCACATGAAGCAGCACGACTTCT
TCAAGTCCGCCATGCCCGAAGGCTACGTCCAGGAGCGCACCATCTTCTTCAAGGACGACGGCAACTAC
AAGACCCGCGCCGAGGTGAAGTTCGAGGGCGACACCCTGGTGAACCGCATCGAGCTGAAGGGCATCGA
CTTCAAGGAGGACGGCAACATCCTGGGGCACAAGCTGGAGTACAACTACAACAGCCACAACGTCTATA
TCATGGCCGACAAGCAGAAGAACGGCATCAAGGTGAACTTCAAGATCCGCCACAACATCGAGGACGGC
AGCGTGCAGCTCGCCGACCACTACCAGCAGAACACCCCCATCGGCGACGGCCCCGTGCTGCTGCCCGA
CAACCACTACCTGAGCACCCAGTCCGCCCTGAGCAAAGACCCCAACGAGAAGCGCGATCACATGGTCC

3' arm

TGCTGGAGTTCGTGACCGCCGCCGGGATCACTCTCGGCATGGACGAGCTGTACAAGTAAACCGCTAGCT

sgRNA2 target Mutation

TGTTGCACCGTCGAGGCCACAGGAGCAGAAACATGGAATGCCAGACGCTGGGGATGCTGGTACAAGTT
GTGGGACTGCATGCTACTGTCTAGAGCTTGTCTCAATGGATCTAGAACTTCATCGCCCTCTGATCGCCG
ATCACCTCTGAGACCCACCTTGCTCATAAACAAAATGCCCATGTTGGTCCTCTGCCCTGGACCTGTGAC

ATTCTGGACTATTTCTGTGTTTATTTGTGGCCGAGTGTAACAACCATATAATAAATCACCTCTTCCGCTG
TTTTAGCTGAAGAATTAAATCATCTTGTCTATTATGTTTTTTATGGTTCCATCGGGTGGGGGTTTTCTGTC
ATTAGAGTTTGCCCTGTCACTACCTGTGCTATGGAGGGTATCAAAGCTATAAAGGCAACAGCCCGGGTT
ACGTGGTGTGGTTTGCATCTCGCTGGAGTTGGATGGGATATGGTACGTCTCTGGAGGCAGGTTTTAGGT
TTCGCGGGTTATTAGGGGCCAATGCTGGTACCGCCCCTTTCCAGGAGGGCTGCCTTGGCGACTACCGCT
TAGACCATGTCGTGCCTTTGGGTTGGGTTGCCATAGTGACACGCAAAGCGTGCTGGGAACGCCCTGCGG
CATGTCGCACGCGTACTTCATACACCATAGAGTATGGTTCCGGGTCTGCTGGCTAGAGCGTCACTTCTTC
CGCAGGAAGCGGAAGAGCGATCGGGTAGGCGGCTCTTTGTCGAAGCTAGAGGACCGGCAGGCGGCAG
CAGCAACTACGGCGGCGGCGGCAGGTGAGGGAGGCGGGAGACTTAGGTGGAGGCCGCCCGCGGtggagct
ccagcttttgttccctttagtgagggttaattgcgcgcttggcgtaatcatggtcatagctgtttcctgtgtgaaattgttatccgctcac……-3’

Supplementary Text S14 Sequence of Human Rpl41 targeting construct hRpl41-IRES-mito-EGFP.

Supplementary Text S15

5’...ttacgccagctggcgaaagggggatgtgctgcaaggcgattaagttgggtaacgccagggttttcccagtcacgacgttgtaaaacgacggccagtgagcgcgcgt

Plasmid backbone 5’ arm

aatacgactcactatagggcgaattgTGAGATGTTCCACAAGAGCCTGGAGAGGGCCGAGGCCGGAGATAACCTCGGG
GCCCTGGTCCGAGGCTTGAAGCGGGAGGACTTGCGGCGGGGCCTGGTCATGGTCAAGCCAGGTTCCAT
CAAGCCCCACCAGAAGGTGGAGGCCCAGGTGAGGGCTCCAGGTGACGGTGGGCAGGGTTGAGCCAAG
CTCTCCCCAGCCTCCAGCCAAGCCCAGCTCACCGTCATTGCTTGCTCTCCTCCAGGTTTACATCCTCAGC
AAGGAGGAAGGTGGCCGCCACAAGCCCTTTGTGTCCCACTTCATGCCTGTCATGTTCTCCCTGACTTGG
GACATGGCCTGTCGGATTATCCTGCCCCCAGAGAAGGTACGGTGGGTGGGAGGAATGTAGGGTGGAGG
GGGGACTTTTTATATTACCTTTGCTTCACTCATAGTTCCAAAGATACACTGTGACAACCCGAGTGGCTTT
ATTTCCTTAGGTGGAAAGGGGCTGCTTGCCGCTGCTAGCCAACAGAAGGGGCATGGCCCATGGACTTA
GGGGCGGGAGTCCTCATCCAGTGTGCCCTAAGCTGAAGCAGGTTAAAAGTTTAGCTCTGCCAGCATGTA
CCCACAGAAAGATTTCCTAGAAAAGGTCTCTTCTTTTTGAGCGTTTCACTATATATGAAGGCTTAAGAA
GTTCTGTGACCAAGAAACCTGCATAACCCACAACTTCCTAGACATTTTTTTCTCAAGAACATACCTTAG
GAACACTCATCACTGGTCTCTTTTATGTTTTCCTGTCTCAATATGCTGTTTGCATCTGCCTGACAGCCTGG
GATGGATGAAGCTGTCCCCAGGCAGAACCATTCCCCTGCTGCATTCTCCCATGACTCTTAACCTCCGAG
GTAGCCAAAAAAGACTTAAGGAATGAAGGCACCCTGGAGGCCACGCATGATCCTTCTCTTCCTACTCTA
GGAGCTTGCCATGCCCGGGGAGGACCTGAAGTTCAACCTAATCTTGCGGCAGCCAATGATCTTAGAGA

sgRNA1 target Mutation

AAGGCCAGCGTTTCACaCTGCGAGATGGCAACCGGACTATTGGCACCGGTCTAGTCACCAACACGCTGG

IRES

CCATGACTGAGGAGGAGAAGAATATCAAATGGGGTTAACCCCCCCCTAACGTTACTGGCCGAAGCCGC
TTGGAATAAGGCCGGTGTGCGTTTGTCTATATGTTATTTTCCACCATATTGCCGTCTTTTGGCAATGTGA
GGGCCCGGAAACCTGGCCCTGTCTTCTTGACGAGCATTCCTAGGGGTCTTTCCCCTCTCGCCAAAGGAA
TGCAAGGTCTGTTGAATGTCGTGAAGGAAGCAGTTCCTCTGGATGCTTCTTGAAGACAAACAACGTCTG
TAGCGACCCTTTGCAGGCAGCGGAACCCCCCACCTGGCGACAGGTGCCTCTGCGGCCAAAAGCCACGT
GTATAAGATACACCTGCAAAGGCGGCACAACCCCAGTGCCACGTTGTGAGTTGGATAGTTGTGGAAAG
AGTCAAATGGCTCTCCTCAAGCGTATTCAACAAGGGGCTGAAGGATGCCCAGAAGGTACCCCATTGTAT
GGGATCTGATCTGGGGCCTCGGTGCACATGCTTTACATGTGTTTAGTCGAGGTTAAAAAACGTCTAGGC
CCCCCGAACCACGGGGACGTGGTTTTCCTTTGAAAAACACGATGATAATATGGCCACAACCATGCTGAG

Mito **mCherry**

CCTGCGCCAGAGCATCCGCTTCTTCAAGCGCAGCGGCATCGTGAGCAAGGGCGAGGAGGATAACATGG
CCATCATCAAGGAGTTCATGCGCTTCAAGGTGCACATGGAGGGCTCCGTGAACGGCCACGAGTTCGAG
ATCGAGGGCGAGGGCGAGGGCCGCCCCTACGAGGGCACCCAGACCGCCAAGCTGAAGGTGACCAAGG
GTGGCCCCCTGCCCTTCGCCTGGGACATCCTGTCCCCTCAGTTCATGTACGGCTCCAAGGCCTACGTGA
AGCACCCCGCCGACATCCCCGACTACTTGAAGCTGTCCTTCCCCGAGGGCTTCAAGTGGGAGCGCGTGA
TGAACTTCGAGGACGGCGGCGTGGTGACCGTGACCCAGGACTCCTCCCTGCAGGACGGCGAGTTCATCT
ACAAGGTGAAGCTGCGCGGCACCAACTTCCCCTCCGACGGCCCCGTAATGCAGAAGAAGACCATGGGC
TGGGAGGCCTCCTCCGAGCGGATGTACCCCGAGGACGGCGCCCTGAAGGGCGAGATCAAGCAGAGGCT
GAAGCTGAAGGACGGCGGCCACTACGACGCTGAGGTCAAGACCACCTACAAGGCCAAGAAGCCCGTG
CAGCTGCCCGGCGCCTACAACGTCAACATCAAGTTGGACATCACCTCCCACAACGAGGACTACACCATC
GTGGAACAGTACGAACGCGCCGAGGGCCGCCACTCCACCGGCGGCATGGACGAGCTGTACAAGTAATG

sgRNA2 target Mutation

AGTGTGCAGATCTCTGCTCAGCTTCCCTTGCGTTTAAGGCCTGCCCTAGCCAacGCTCCCTCCTGCTTCCA
GTACCCTCTCATGGCATAGGCTGCAACCCAGCAGAGGGCAGCTAGATGGACATTTCCCCTGCTCGGAAG
GGTTGGCCTGCCTGGCTGGGGAGGTCAGTAAACTTTGAATAGTAAGCCAGCCTGTGTCCTGTGTCCTTT

GTGCAAATTGGAGAGGATAGGGGAGGACAAAAAGGGTGTTGAACCCCAGACAGATGGGGAGCTTGTG
AGATCCCAGAGCAGTTTTAACGGAGAAATCAAGTCCAGTGAGCTTAGTGATTGCCTAGACACAGTAGG
ATGCCGTTATTCAGCCTCCTGGGAGATAGTGGGAAAGAATGTTCTATCGCTCTGGAGCCAGCATGGCTT
CAGCTCAGGTGCCAAACCCATCTTGTTTCCTCGGAATACTAAGTGAATGTCTAGCATCCATTCACATTCT
GTGGGAACAGGGACTGGACAGGCATACCTCAGATGCTGTAGGTTTGCTTCCAGACTACCACAATAAAG
CAGGTCACAAATTTTTTGATTTCTCAGTGAAGTTATGTTTATATTATAGTTGACCCTTGAACAATGCAGG
GGTTAGGGGTGTCAGCCCTCCTCCATGCAGCCACAAATCCACATATAACTTGTTTTTGAGATGGGGCCA
GCTTTGTGCAGTGGCAGTATCATAGCCAATGACTCCATGATGACTTGAAATATAATCAGCATTGGCAGT
TTTCAACGGTCTCTATGGGGAGGCTGAACAAAGGGAAAAAAAGAGATATAGGGTCTCACTGTCACCCA
GGCTGGGTGCGGTGGCACCATCCTAGCTCACTGCAGCCTTGGACTCCTGGGCTCAACCTTGGTCTCCCC
AACTGCTGGGATTAGAGGTGTAAGCTACCGTGTCTGGCTCCTCACATATAACTTGACTCCCCCAAAATG
3’ arm
TAACTAGTAACCTGCTGCTGGCCAGAAGCCTCGCTGATAGCATACAAACACATACtggagctccagcttttgttccctttagtgagggttaattgcgcgcttggcgtaatcatggtcatagctgtttcctgtgtgaaattgttatccgctcac……-3’

Supplementary Text S15 Sequence of Human TUFm targeting construct hTUFm-IRES-mito-mCherry.

Supplementary Text S16:

>pSP73-Sp6- **vertebrate kozak**-NLS-hSpD10ACas9n-NLS-stop HindIII
TACACATACGATTTAGGTGACACTATAGAACCAGACCGCCACCATGGTACCAAAAAAGAAGAGAAAG
GTAGCGTCCGACAAGAAGTACAGCATCGGCCTGGCCATCGGCACCAACTCTGTGGGCTGGGCCGTGAT
CACCGACGAGTACAAGGTGCCCAGCAAGAAATTCAAGGTGCTGGGCAACACCGACCGGCACAGCATCA
AGAAGAACCTGATCGGAGCCCTGCTGTTCGACAGCGGCGAAACAGCCGAGGCCACCCGGCTGAAGAGA
ACCGCCAGAAGAAGATACACCAGACGGAAGAACCGGATCTGCTATCTGCAAGAGATCTTCAGCAACGA
GATGGCCAAGGTGGACGACAGCTTCTTCCACAGACTGGAAGAGTCCTTCCTGGTGGAAGAGGATAAGA
AGCACGAGCGGCACCCCATCTTCGGCAACATCGTGGACGAGGTGGCCTACCACGAGAAGTACCCCACC
ATCTACCACCTGAGAAAGAAACTGGTGGACAGCACCGACAAGGCCGACCTGCGGCTGATCTATCTGGC
CCTGGCCCACATGATCAAGTTCCGGGGCCACTTCCTGATCGAGGGCGACCTGAACCCCGACAACAGCG
ACGTGGACAAGCTGTTCATCCAGCTGGTGCAGACCTACAACCAGCTGTTCGAGGAAAACCCCATCAAC
GCCAGCGGCGTGGACGCCAAGGCCATCCTGTCTGCCAGACTGAGCAAGAGCAGACGGCTGGAAAATCT
GATCGCCCAGCTGCCCGGCGAGAAGAAGAATGGCCTGTTCGGCAACCTGATTGCCCTGAGCCTGGGCC
TGACCCCCAACTTCAAGAGCAACTTCGACCTGGCCGAGGATGCCAAACTGCAGCTGAGCAAGGACACC
TACGACGACGACCTGGACAACCTGCTGGCCCAGATCGGCGACCAGTACGCCGACCTGTTTCTGGCCGCC
AAGAACCTGTCCGACGCCATCCTGCTGAGCGACATCCTGAGAGTGAACACCGAGATCACCAAGGCCCC
CCTGAGCGCCTCTATGATCAAGAGATACGACGAGCACCACCAGGACCTGACCCTGCTGAAAGCTCTCGT
GCGGCAGCAGCTGCCTGAGAAGTACAAAGAGATTTTCTTCGACCAGAGCAAGAACGGCTACGCCGGCT
ACATTGACGGCGGAGCCAGCCAGGAAGAGTTCTACAAGTTCATCAAGCCCATCCTGGAAAAGATGGAC
GGCACCGAGGAACTGCTCGTGAAGCTGAACAGAGAGGACCTGCTGCGGAAGCAGCGGACCTTCGACAA
CGGCAGCATCCCCCACCAGATCCACCTGGGAGAGCTGCACGCCATTCTGCGGCGGCAGGAAGATTTTTA
CCCATTCCTGAAGGACAACCGGGAAAAGATCGAGAAGATCCTGACCTTCCGCATCCCCTACTACGTGG
GCCCTCTGGCCAGGGGAAACAGCAGATTCGCCTGGATGACCAGAAAGAGCGAGGAAACCATCACCCCC
TGGAACTTCGAGGAAGTGGTGGACAAGGGCGCTTCCGCCCAGAGCTTCATCGAGCGGATGACCAACTT
CGATAAGAACCTGCCCAACGAGAAGGTGCTGCCCAAGCACAGCCTGCTGTACGAGTACTTCACCGTGT
ATAACGAGCTGACCAAAGTGAAATACGTGACCGAGGGAATGAGAAAGCCCGCCTTCCTGAGCGGCGAG
CAGAAAAAGGCCATCGTGGACCTGCTGTTCAAGACCAACCGGAAAGTGACCGTGAAGCAGCTGAAAGA
GGACTACTTCAAGAAAATCGAGTGCTTCGACTCCGTGGAAATCTCCGGCGTGGAAGATCGGTTCAACGC
CTCCCTGGGCACATACCACGATCTGCTGAAAATTATCAAGGACAAGGACTTCCTGGACAATGAGGAAA
ACGAGGACATTCTGGAAGATATCGTGCTGACCCTGACACTGTTTGAGGACAGAGAGATGATCGAGGAA
CGGCTGAAAACCTATGCCCACCTGTTCGACGACAAAGTGATGAAGCAGCTGAAGCGGCGGAGATACAC
CGGCTGGGGCAGGCTGAGCCGGAAGCTGATCAACGGCATCCGGGACAAGCAGTCCGGCAAGACAATCC
TGGATTTCCTGAAGTCCGACGGCTTCGCCAACAGAAACTTCATGCAGCTGATCCACGACGACAGCCTGA
CCTTTAAAGAGGACATCCAGAAAGCCCAGGTGTCCGGCCAGGGCGATAGCCTGCACGAGCACATTGCC
AATCTGGCCGGCAGCCCCGCCATTAAGAAGGGCATCCTGCAGACAGTGAAGGTGGTGGACGAGCTCGT
GAAAGTGATGGGCCGGCACAAGCCCGAGAACATCGTGATCGAAATGGCCAGAGAGAACCAGACCACC
CAGAAGGGACAGAAGAACAGCCGCGAGAGAATGAAGCGGATCGAAGAGGGCATCAAAGAGCTGGGC
AGCCAGATCCTGAAAGAACACCCCGTGGAAAACACCCAGCTGCAGAACGAGAAGCTGTACCTGTACTA
CCTGCAGAATGGGCGGGATATGTACGTGGACCAGGAACTGGACATCAACCGGCTGTCCGACTACGATG
TGGACCATATCGTGCCTCAGAGCTTTCTGAAGGACGACTCCATCGACAACAAGGTGCTGACCAGAAGC
GACAAGAACCGGGGCAAGAGCGACAACGTGCCCTCCGAAGAGGTCGTGAAGAAGATGAAGAACTACT
GGCGGCAGCTGCTGAACGCCAAGCTGATTACCCAGAGAAAGTTCGACAATCTGACCAAGGCCGAGAGA
GGCGGCCTGAGCGAACTGGATAAGGCCGGCTTCATCAAGAGACAGCTGGTGGAAACCCGGCAGATCAC
AAAGCACGTGGCACAGATCCTGGACTCCCGGATGAACACTAAGTACGACGAGAATGACAAGCTGATCC
GGGAAGTGAAAGTGATCACCCTGAAGTCCAAGCTGGTGTCCGATTTCCGGAAGGATTTCCAGTTTTACA
AAGTGCGCGAGATCAACAACTACCACCACGCCCACGACGCCTACCTGAACGCCGTCGTGGGAACCGCC
CTGATCAAAAAGTACCCTAAGCTGGAAAGCGAGTTCGTGTACGGCGACTACAAGGTGTACGACGTGCG
GAAGATGATCGCCAAGAGCGAGCAGGAAATCGGCAAGGCTACCGCCAAGTACTTCTTCTACAGCAACA
TCATGAACTTTTTCAAGACCGAGATTACCCTGGCCAACGGCGAGATCCGGAAGCGGCCTCTGATCGAGA
CAAACGGCGAAACCGGGGAGATCGTGTGGGATAAGGGCCGGGATTTTGCCACCGTGCGGAAAGTGCTG
AGCATGCCCCAAGTGAATATCGTGAAAAAGACCGAGGTGCAGACAGGCGGCTTCAGCAAAGAGTCTAT
CCTGCCCAAGAGGAACAGCGATAAGCTGATCGCCAGAAAGAAGGACTGGGACCCTAAGAAGTACGGC
GGCTTCGACAGCCCCACCGTGGCCTATTCTGTGCTGGTGGTGGCCAAAGTGGAAAAGGGCAAGTCCAA
GAAACTGAAGAGTGTGAAAGAGCTGCTGGGGATCACCATCATGGAAAGAAGCAGCTTCGAGAAGAAT
CCCATCGACTTTCTGGAAGCCAAGGGCTACAAAGAAGTGAAAAAGGACCTGATCATCAAGCTGCCTAA
GTACTCCCTGTTCGAGCTGGAAAACGGCCGGAAGAGAATGCTGGCCTCTGCCGGCGAACTGCAGAAGG
GAAACGAACTGGCCCTGCCCTCCAAATATGTGAACTTCCTGTACCTGGCCAGCCACTATGAGAAGCTGA
AGGGCTCCCCCGAGGATAATGAGCAGAAACAGCTGTTTGTGGAACAGCACAAGCACTACCTGGACGAG
ATCATCGAGCAGATCAGCGAGTTCTCCAAGAGAGTGATCCTGGCCGACGCTAATCTGGACAAAGTGCT

GTCCGCCTACAACAAGCACCGGGATAAGCCCATCAGAGAGCAGGCCGAGAATATCATCCACCTGTTTA
CCCTGACCAATCTGGGAGCCCCTGCCGCCTTCAAGTACTTTGACACCACCATCGACCGGAAGAGGTACA
CCAGCACCAAAGAGGTGCTGGACGCCACCCTGATCCACCAGAGCATCACCGGCCTGTACGAGACACGG
ATCGACCTGTCTCAGCTGGGAGGCGACAGCCCCAAGAAGAAGAGAAAGGTGGAGGCCAGCTAATGAA
TTCCTAGAGCTCGCCGAGACCGGCGCGCCGGATCCACTAGTCCAGTGTGGTGGAATTCTGCAGATATCC
AGCACAGTGGCGGCCGCTCGAGTCTAGAGGGCCCGTTTAAACCCGCTGATCAGCCTCGACTGTGCCTTC
TAGTTGCCAGCCATCTGTTGTTTGCCCCTCCCCCGTGCCTTCCTTGACCCTGGAAGGTGCCACTCCCACT
GTCCTTTCCTAATAAAATGAGGAAATTGCATCGCATTGTCTGAGTAGGTGTCATTCTATTCTGGGGGGT
GGGGTGGGGCAGGACAGCAAGGGGGAGGATTGGGAAGACAATAGCAGGCATGCTGGGGATGCGGTGG
GCTCTATGGCTTCTGAGGCGGAAAGAACCAGCTGGGGCTCTAGGGGGTATCCCCACGCGCCCTGTAGC
GGCGCATTAAGCGCGGCGGGTGTGGTGGTTACGCGCAGCGTGACCGCTACACTTGCCAGCGCCCTAGC
GCCCGCTCCTTTCGCTTTCTTCCCTTCCTTTCTCGCCACGTTCGCCGGCTTTCCCCGTCAAGCTCTAAATC
GGGGGCTCCCTTTAGGGTTCCGATTTAGTGCTTTACGGCACCTCGACCCCAAAAAACTTGATTAGGGTG
ATGGTTCACGTAGTGGGCCATCGCCCTGATAGACGGTTTTTCGCCCTTTGACGTTGGAGTCCACGTTCTT
TAATAGTGGACTCTTGTTCCAAACTGGAACAACACGAATTCGAGCTCGGTACCCGGGGATCCTCTAGAG
TCGACCTGCAGGCATGCAAGCTTCAGCTGCTCGAGGCCGGTCTCCCTATAGTGAGTCGTATTAATTTCG
ATAAGCCAGGTTAACCTGCATTAATGAATCGGCCAACGCGCGGGGAGAGGCGGTTTGCGTATTGGGCG
CTCTTCCGCTTCCTCGCTCACTGACTCGCTGCGCTCGGTCGTTCGGCTGCGGCGAGCGGTATCAGCTCAC
TCAAAGGCGGTAATACGGTTATCCACAGAATCAGGGGATAACGCAGGAAAGAACATGTGAGCAAAAG
GCCAGCAAAAGGCCAGGAACCGTAAAAAGGCCGCGTTGCTGGCGTTTTTCCATAGGCTCCGCCCCCCTG
ACGAGCATCACAAAAATCGACGCTCAAGTCAGAGGTGGCGAAACCCGACAGGACTATAAAGATACCA
GGCGTTTCCCCCTGGAAGCTCCCTCGTGCGCTCTCCTGTTCCGACCCTGCCGCTTACCGGATACCTGTCC
GCCTTTCTCCCTTCGGGAAGCGTGGCGCTTTCTCATAGCTCACGCTGTAGGTATCTCAGTTCGGTGTAGG
TCGTTCGCTCCAAGCTGGGCTGTGTGCACGAACCCCCCGTTCAGCCCGACCGCTGCGCCTTATCCGGTA
ACTATCGTCTTGAGTCCAACCCGGTAAGACACGACTTATCGCCACTGGCAGCAGCCACTGGTAACAGGA
TTAGCAGAGCGAGGTATGTAGGCGGTGCTACAGAGTTCTTGAAGTGGTGGCCTAACTACGGCTACACTA
GAAGAACAGTATTTGGTATCTGCGCTCTGCTGAAGCCAGTTACCTTCGGAAAAAGAGTTGGTAGCTCTT
GATCCGGCAAACAAACCACCGCTGGTAGCGGTGGTTTTTTTGTTTGCAAGCAGCAGATTACGCGCAGAA
AAAAAGGATCTCAAGAAGATCCTTTGATCTTTTCTACGGGGTCTGACGCTCAGTGGAACGAAAACTCAC
GTTAAGGGATTTTGGTCATGAGATTATCAAAAAGGATCTTCACCTAGATCCTTTTAAATTAAAAATGAA
GTTTTAAATCAATCTAAAGTATATATGAGTAAACTTGGTCTGACAGTTACCAATGCTTAATCAGTGAGG
CACCTATCTCAGCGATCTGTCTATTTCGTTCATCCATAGTTGCCTGACTCCCCGTCGTGTAGATAACTAC
GATACGGGAGGGCTTACCATCTGGCCCCAGTGCTGCAATGATACCGCGAGACCCACGCTCACCGGCTCC
AGATTTATCAGCAATAAACCAGCCAGCCGGAAGGGCCGAGCGCAGAAGTGGTCCTGCAACTTTATCCG
CCTCCATCCAGTCTATTAATTGTTGCCGGGAAGCTAGAGTAAGTAGTTCGCCAGTTAATAGTTTGCGCA
ACGTTGTTGCCATTGCTACAGGCATCGTGGTGTCACGCTCGTCGTTTGGTATGGCTTCATTCAGCTCCGG
TTCCCAACGATCAAGGCGAGTTACATGATCCCCCATGTTGTGCAAAAAAGCGGTTAGCTCCTTCGGTCC
TCCGATCGTTGTCAGAAGTAAGTTGGCCGCAGTGTTATCACTCATGGTTATGGCAGCACTGCATAATTC
TCTTACTGTCATGCCATCCGTAAGATGCTTTTCTGTGACTGGTGAGTACTCAACCAAGTCATTCTGAGAA
TAGTGTATGCGGCGACCGAGTTGCTCTTGCCCGGCGTCAATACGGGATAATACCGCGCCACATAGCAGA
ACTTTAAAAGTGCTCATCATTGGAAAACGTTCTTCGGGGCGAAAACTCTCAAGGATCTTACCGCTGTTG
AGATCCAGTTCGATGTAACCCACTCGTGCACCCAACTGATCTTCAGCATCTTTTACTTTCACCAGCGTTT
CTGGGTGAGCAAAAACAGGAAGGCAAAATGCCGCAAAAAAGGGAATAAGGGCGACACGGAAATGTTG
AATACTCATACTCTTCCTTTTTCAATATTATTGAAGCATTTATCAGGGTTATTGTCTCATGAGCGGATAC
ATATTTGAATGTATTTAGAAAAATAAACAAATAGGGGTTCCGCGCACATTTCCCCGAAAAGTGCCACCT
GACGTCTAAGAAACCATTATTATCATGACATTAACCTATAAAAATAGGCGTATCACGAGGCCCTTTCGT
CTCGCGCGTTTCGGTGATGACGGTGAAAACCTCTGACACATGCAGCTCCCGGAGACGGTCACAGCTTGT
CTGTAAGCGGATGCCGGGAGCAGACAAGCCCGTCAGGGCGCGTCAGCGGGTGTTGGCGGGTGTCGGGG
CTGGCTTAACTATGCGGCATCAGAGCAGATTGTACTGAGAGTGCACCATATGGACATATTGTCGTTAGA
ACGCGGCTACAATTAATACATAACCTTATGTATCATACACATACGATTTAGGTGACA

Supplementary Text S16 Sequence of Cas9n transcript template.

Supplementary Sequence S17:

>pSP73-Sp6- vertebrate kozak-NLS-hSpCas9-NLS-stop HindIII
TACACATACGATTTAGGTGACACTATAGAACCAGACCGCCACCATGGTACCAAAAAAGAAGAGAAAG
GTAGCAGCCGACAAGAAGTACAGCATCGGCCTGGACATCGGCACCAACTCTGTGGGCTGGGCCG
TGATCACCGACGAGTACAAGGTGCCCAGCAAGAAATTCAAGGTGCTGGGCAACACCGACCGGCA
CAGCATCAAGAAGAACCTGATCGGAGCCCTGCTGTTCGACAGCGGCGAAACAGCCGAGGCCACC
CGGCTGAAGAGAACCGCCAGAAGAAGATACACCAGACGGAAGAACCGGATCTGCTATCTGCAAG
AGATCTTCAGCAACGAGATGGCCAAGGTGGACGACAGCTTCTTCCACAGACTGGAAGAGTCCTTC
CTGGTGGAAGAGGATAAGAAGCACGAGCGGCACCCCATCTTCGGCAACATCGTGGACGAGGTGG
CCTACCACGAGAAGTACCCCACCATCTACCACCTGAGAAAGAAACTGGTGGACAGCACCGACAA
GGCCGACCTGCGGCTGATCTATCTGGCCCTGGCCCACATGATCAAGTTCCGGGGCCACTTCCTGA
TCGAGGGCGACCTGAACCCCGACAACAGCGACGTGGACAAGCTGTTCATCCAGCTGGTGCAGAC
CTACAACCAGCTGTTCGAGGaaAACCCCATCAACGCCAGCGGCGTGGACGCCAAGGCCATCCTGT
CTGCCAGACTGAGCAAGAGCAGACGGCTGGAAAATCTGATCGCCCAGCTGCCCGGCGAGAAGAA
GAATGGCCTGTTCGGAAACCTGATTGCCCTGAGCCTGGGCCTGACCCCCAACTTCAAGAGCAACT
TCGACCTGGCCGAGGATGCCAAACTGCAGCTGAGCAAGGACACCTACGACGACGACCTGGACAA
CCTGCTGGCCCAGATCGGCGACCAGTACGCCGACCTGTTTCTGGCCGCCAAGAACCTGTCCGAC
GCCATCCTGCTGAGCGACATCCTGAGAGTGAACACCGAGATCACCAAGGCCCCCCTGAGCGCCT
CTATGATCAAGAGATACGACGAGCACCACCAGGACCTGACCCTGCTGAAAGCTCTCGTGCGGCA
GCAGCTGCCTGAGAAGTACAAAGAGATTTTCTTCGACCAGAGCAAGAACGGCTACGCCGGCTAC
ATTGACGGCGGAGCCAGCCAGGAAGAGTTCTACAAGTTCATCAAGCCCATCCTGGAAAAGATGG
ACGGCACCGAGGAACTGCTCGTGAAGCTGAACAGAGAGGACCTGCTGCGGAAGCAGCGGACCTT
CGACAACGGCAGCATCCCCCACCAGATCCACCTGGGAGAGCTGCACGCCATTCTGCGGCGGCAG
GAAGATTTTTACCCATTCCTGAAGGACAACCGGGAAAAGATCGAGAAGATCCTGACCTTCCGCAT
CCCCTACTACGTGGGCCCTCTGGCCAGGGGAAACAGCAGATTCGCCTGGATGACCAGAAAGAGC
GAGGAAACCATCACCCCCTGGAACTTCGAGGAAGTGGTGGACAAGGGCGCTTCCGCCCAGAGCT
TCATCGAGCGGATGACCAACTTCGATAAGAACCTGCCCAACGAGAAGGTGCTGCCCAAGCACAG
CCTGCTGTACGAGTACTTCACCGTGTATAACGAGCTGACCAAAGTGAAATACGTGACCGAGGGAA
TGAGAAAGCCCGCCTTCCTGAGCGGCGAGCAGAAAAAGGCCATCGTGGACCTGCTGTTCAAGAC
CAACCGGAAAGTGACCGTGAAGCAGCTGAAAGAGGACTACTTCAAGAAAATCGAGTGCTTCGAC
TCCGTGGAAATCTCCGGCGTGGAAGATCGGTTCAACGCCTCCCTGGGCACATACCACGATCTGCT
GAAAATTATCAAGGACAAGGACTTCCTGGACAATGAGGAAAACGAGGACATTCTGGAAGATATCG
TGCTGACCCTGACACTGTTTGAGGACAGAGAGATGATCGAGGAACGGCTGAAAACCTATGCCCA
CCTGTTCGACGACAAAGTGATGAAGCAGCTGAAGCGGCGGAGATACACCGGCTGGGGCAGGCTG
AGCCGGAAGCTGATCAACGGCATCCGGGACAAGCAGTCCGGCAAGACAATCCTGGATTTCCTGA
AGTCCGACGGCTTCGCCAACAGAAACTTCATGCAGCTGATCCACGACGACAGCCTGACCTTTAAA
GAGGACATCCAGAAAGCCCAGGTGTCCGGCCAGGGCGATAGCCTGCACGAGCACATTGCCAATC
TGGCCGGCAGCCCCGCCATTAAGAAGGGCATCCTGCAGACAGTGAAGGTGGTGGACGAGCTCGT
GAAAGTGATGGGCCGGCACAAGCCCGAGAACATCGTGATCGAAATGGCCAGAGAGAACCAGACC
ACCCAGAAGGGACAGAAGAACAGCCGCGAGAGAATGAAGCGGATCGAAGAGGGCATCAAAGAG
CTGGGCAGCCAGATCCTGAAAGAACACCCCGTGGAAAACACCCAGCTGCAGAACGAGAAGCTGT
ACCTGTACTACCTGCAGAATGGGCGGGATATGTACGTGGACCAGGAACTGGACATCAACCGGCT
GTCCGACTACGATGTGGACCATATCGTGCCTCAGAGCTTTCTGAAGGACGACTCCATCGACAACA
AGGTGCTGACCAGAAGCGACAAGAACCGGGGCAAGAGCGACAACGTGCCCTCCGAAGAGGTCGT
GAAGAAGATGAAGAACTACTGGCGGCAGCTGCTGAACGCCAAGCTGATTACCCAGAGAAAGTTC
GACAATCTGACCAAGGCCGAGAGAGGCGGCCTGAGCGAACTGGATAAGGCCGGCTTCATCAAGA
GACAGCTGGTGGAAACCCGGCAGATCACAAAGCACGTGGCACAGATCCTGGACTCCCGGATGAA
CACTAAGTACGACGAGAATGACAAGCTGATCCGGGAAGTGAAAGTGATCACCCTGAAGTCCAAG
CTGGTGTCCGATTTCCGGAAGGATTTCCAGTTTTACAAAGTGCGCGAGATCAACAACTACCACCA
CGCCCACGACGCCTACCTGAACGCCGTCGTGGGAACCGCCCTGATCAAAAAGTACCCTAAGCTG
GAAAGCGAGTTCGTGTACGGCGACTACAAGGTGTACGACGTGCGGAAGATGATCGCCAAGAGCG
AGCAGGAAATCGGCAAGGCTACCGCCAAGTACTTCTTCTACAGCAACATCATGAACTTTTTCAAG
ACCGAGATTACCCTGGCCAACGGCGAGATCCGGAAGCGGCCTCTGATCGAGACAAACGGCGAAA
CCGGGGAGATCGTGTGGGATAAGGGCCGGGATTTTGCCACCGTGCGGAAAGTGCTGAGCATGCC
CCAAGTGAATATCGTGAAAAAGACCGAGGTGCAGACAGGCGGCTTCAGCAAAGAGTCTATCCTG
CCCAAGAGGAACAGCGATAAGCTGATCGCCAGAAAGAAGGACTGGGACCCTAAGAAGTACGGCG
GCTTCGACAGCCCCACCGTGGCCTATTCTGTGCTGGTGGTGGCCAAAGTGGAAAAGGGCAAGTC
CAAGAAACTGAAGAGTGTGAAAGAGCTGCTGGGGATCACCATCATGGAAAGAAGCAGCTTCGAG
AAGAATCCCATCGACTTTCTGGAAGCCAAGGGCTACAAAGAAGTGAAAAAGGACCTGATCATCAA
GCTGCCTAAGTACTCCCTGTTCGAGCTGGAAAACGGCCGGAAGAGAATGCTGGCCTCTGCCGGC

GAACTGCAGAAGGGAAACGAACTGGCCCTGCCCTCCAAATATGTGAACTTCCTGTACCTGGCCAG
CCACTATGAGAAGCTGAAGGGCTCCCCCGAGGATAATGAGCAGAAACAGCTGTTTGTGGAACAG
CACAAGCACTACCTGGACGAGATCATCGAGCAGATCAGCGAGTTCTCCAAGAGAGTGATCCTGG
CCGACGCTAATCTGGACAAAGTGCTGTCCGCCTACAACAAGCACCGGGATAAGCCCATCAGAGA
GCAGGCCGAGAATATCATCCACCTGTTTACCCTGACCAATCTGGGAGCCCCTGCCGCCTTCAAGT
ACTTTGACACCACCATCGACCGGAAGAGGTACACCAGCACCAAAGAGGTGCTGGACGCCACCCT
GATCCACCAGAGCATCACCGGCCTGTACGAGACACGGATCGACCTGTCTCAGCTGGGAGGCGAC
AAAAGGCCGGCGGCCACGAAAAAGGCCGGCCAGGCAAAAAAGAAAAAGTAATGAATTCCTAGAGC
TCGCCGAGACCGGCGCGCCGGATCCACTAGTCCAGTGTGGTGGAATTCTGCAGATATCCAGCACAGTG
GCGGCCGCTCGAGTCTAGAGGGCCCGTTTAAACCCGCTGATCAGCCTCGACTGTGCCTTCTAGTTGCCA
GCCATCTGTTGTTTGCCCCTCCCCCGTGCCTTCCTTGACCCTGGAAGGTGCCACTCCCACTGTCCTTTCCT
AATAAAATGAGGAAATTGCATCGCATTGTCTGAGTAGGTGTCATTCTATTCTGGGGGGTGGGGTGGGGC
AGGACAGCAAGGGGGAGGATTGGGAAGACAATAGCAGGCATGCTGGGGATGCGGTGGGCTCTATGGC
TTCTGAGGCGGAAAGAACCAGCTGGGGCTCTAGGGGGTATCCCCACGCGCCCTGTAGCGGCGCATTAA
GCGCGGCGGGTGTGGTGGTTACGCGCAGCGTGACCGCTACACTTGCCAGCGCCCTAGCGCCCGCTCCTT
TCGCTTTCTTCCCTTCCTTTCTCGCCACGTTCGCCGGCTTTCCCCGTCAAGCTCTAAATCGGGGGCTCCCT
TTAGGGTTCCGATTTAGTGCTTTACGGCACCTCGACCCCAAAAAACTTGATTAGGGTGATGGTTCACGT
AGTGGGCCATCGCCCTGATAGACGGTTTTTCGCCCTTTGACGTTGGAGTCCACGTTCTTTAATAGTGGAC
TCTTGTTCCAAACTGGAACAACACGAATTCGAGCTCGGTACCCGGGGATCCTCTAGAGTCGACCTGCAG
GCATGCAAGCTTCAGCTGCTCGAGGCCGGTCTCCCTATAGTGAGTCGTATTAATTTCGATAAGCCAGGT
TAACCTGCATTAATGAATCGGCCAACGCGCGGGGAGAGGCGGTTTGCGTATTGGGCGCTCTTCCGCTTC
CTCGCTCACTGACTCGCTGCGCTCGGTCGTTCGGCTGCGGCGAGCGGTATCAGCTCACTCAAAGGCGGT
AATACGGTTATCCACAGAATCAGGGGATAACGCAGGAAAGAACATGTGAGCAAAAGGCCAGCAAAAG
GCCAGGAACCGTAAAAAGGCCGCGTTGCTGGCGTTTTTCCATAGGCTCCGCCCCCCTGACGAGCATCAC
AAAAATCGACGCTCAAGTCAGAGGTGGCGAAACCCGACAGGACTATAAAGATACCAGGCGTTTCCCCC
TGGAAGCTCCCTCGTGCGCTCTCCTGTTCCGACCCTGCCGCTTACCGGATACCTGTCCGCCTTTCTCCCT
TCGGGAAGCGTGGCGCTTTCTCATAGCTCACGCTGTAGGTATCTCAGTTCGGTGTAGGTCGTTCGCTCCA
AGCTGGGCTGTGTGCACGAACCCCCCGTTCAGCCCGACCGCTGCGCCTTATCCGGTAACTATCGTCTTG
AGTCCAACCCGGTAAGACACGACTTATCGCCACTGGCAGCAGCCACTGGTAACAGGATTAGCAGAGCG
AGGTATGTAGGCGGTGCTACAGAGTTCTTGAAGTGGTGGCCTAACTACGGCTACACTAGAAGAACAGT
ATTTGGTATCTGCGCTCTGCTGAAGCCAGTTACCTTCGGAAAAAGAGTTGGTAGCTCTTGATCCGGCAA
ACAAACCACCGCTGGTAGCGGTGGTTTTTTTGTTTGCAAGCAGCAGATTACGCGCAGAAAAAAAGGAT
CTCAAGAAGATCCTTTGATCTTTTCTACGGGGTCTGACGCTCAGTGGAACGAAAACTCACGTTAAGGGA
TTTTGGTCATGAGATTATCAAAAAGGATCTTCACCTAGATCCTTTTAAATTAAAAATGAAGTTTTAAATC
AATCTAAAGTATATATGAGTAAACTTGGTCTGACAGTTACCAATGCTTAATCAGTGAGGCACCTATCTC
AGCGATCTGTCTATTTCGTTCATCCATAGTTGCCTGACTCCCCGTCGTGTAGATAACTACGATACGGGAG
GGCTTACCATCTGGCCCCAGTGCTGCAATGATACCGCGAGACCCACGCTCACCGGCTCCAGATTTATCA
GCAATAAACCAGCCAGCCGGAAGGGCCGAGCGCAGAAGTGGTCCTGCAACTTTATCCGCCTCCATCCA
GTCTATTAATTGTTGCCGGGAAGCTAGAGTAAGTAGTTCGCCAGTTAATAGTTTGCGCAACGTTGTTGC
CATTGCTACAGGCATCGTGGTGTCACGCTCGTCGTTTGGTATGGCTTCATTCAGCTCCGGTTCCCAACGA
TCAAGGCGAGTTACATGATCCCCCATGTTGTGCAAAAAAGCGGTTAGCTCCTTCGGTCCTCCGATCGTT
GTCAGAAGTAAGTTGGCCGCAGTGTTATCACTCATGGTTATGGCAGCACTGCATAATTCTCTTACTGTC
ATGCCATCCGTAAGATGCTTTTCTGTGACTGGTGAGTACTCAACCAAGTCATTCTGAGAATAGTGTATG
CGGCGACCGAGTTGCTCTTGCCCGGCGTCAATACGGGATAATACCGCGCCACATAGCAGAACTTTAAA
AGTGCTCATCATTGGAAAACGTTCTTCGGGGCGAAAACTCTCAAGGATCTTACCGCTGTTGAGATCCAG
TTCGATGTAACCCACTCGTGCACCCAACTGATCTTCAGCATCTTTTACTTTCACCAGCGTTTCTGGGTGA
GCAAAAACAGGAAGGCAAAATGCCGCAAAAAAGGGAATAAGGGCGACACGGAAATGTTGAATACTCA
TACTCTTCCTTTTTCAATATTATTGAAGCATTTATCAGGGTTATTGTCTCATGAGCGGATACATATTTGA
ATGTATTTAGAAAAATAAACAAATAGGGGTTCCGCGCACATTTCCCCGAAAAGTGCCACCTGACGTCTA
AGAAACCATTATTATCATGACATTAACCTATAAAAATAGGCGTATCACGAGGCCCTTTCGTCTCGCGCG
TTTCGGTGATGACGGTGAAAACCTCTGACACATGCAGCTCCCGGAGACGGTCACAGCTTGTCTGTAAGC
GGATGCCGGGAGCAGACAAGCCCGTCAGGGCGCGTCAGCGGGTGTTGGCGGGTGTCGGGGCTGGCTTA
ACTATGCGGCATCAGAGCAGATTGTACTGAGAGTGCACCATATGGACATATTGTCGTTAGAACGCGGCT
ACAATTAATACATAACCTTATGTATCATACACATACGATTTAGGTGACA

Supplementary Sequence S17 Sequence of Cas9 transcript template.

Supplementary Text S18:

>EcoRV-SP6-vertebrate kozak-nls-RecA-nls-stop-EcoRV

GATATCATACGATTTAGGTGACACTATAGAACCGCCACCATGGTCCCAAAAAAGAAAAGGAAGGTGGCTATTGAC
GAGAACAAACAGAAAGCACTGGCAGCAGCACTGGGGCAGATTGAGAAACAGTTCGGGAAGGGATCCATTATGAGG
CTGGGGGAAGACCGCTCAATGGATGTGGAGACTATCAGCACCGGATCTCTGAGTCTGGACATTGCTCTGGGAGCAG
GAGGACTGCCAATGGGACGAATCGTGGAAATCTACGGACCTGAGAGCTCCGGCAAGACCACACTGACCCTGCAGG
TCATCGCCGCTGCACAGAGAGAGGGCAAAACATGCGCCTTCATCGACGCCGAACACGCTCTGGATCCAATCTACGC
TAGGAAGCTGGGCGTGGACATCGATAACCTGCTGTGCTCTCAGCCCGACACTGGGGAACAGGCCCTGGAGATTTGT
GATGCACTGGCTAGGTCCGGAGCTGTGGACGTGATCGTGGTCGATTCTGTGGCCGCTCTGACCCCCAAAGCCGAGA
TCGAAGGGGAGATTGGAGACAGTCATATGGGCCTGGCAGCCCGAATGATGTCACAGGCAATGCGGAAGCTGGCCG
GCAACCTGAAACAGAGCAATACACTGCTGATCTTCATTAACCAGATCCGCATGAAGATTGGCGTGATGTTTGGGAA
CCCCGAGACTACCACAGGAGGCAATGCTCTGAAGTTCTACGCAAGCGTGCGCCTGGACATCCGGAGAATTGGGGCC
GTCAAAGAAGGAGAGAACGTGGTCGGCTCCGAGACCCGAGTGAAGGTGGTCAAGAACAAGATCGCTGCACCTTTC
AAACAGGCCGAATTTCAGATCCTGTACGGCGAGGGCATCAACTTCTACGGGGAACTGGTGGACCTGGGAGTCAAGG
AAAAACTGATTGAGAAGGCAGGAGCCTGGTACTCTTATAAGGGCGAGAAAATCGGACAGGGCAAAGCTAACGCAA
CAGCCTGGCTGAAGGATAATCCTGAGACTGCCAAAGAAATCGAGAAGAAAGTGAGAGAACTGCTGCTGAGTAACC
CAAATTCAACCCCCGATTTTTCAGTGGATGATTCCGAAGGCGTCGCAGAGACTAATGAGGATTTTAGCCCCAAGAA
GAAGAGAAAGGTGGAGGCCAGCTGATAAGATATC

Supplementary Text S18 Sequence of RecA transcript template.

Supplementary Text S19:
>EcoRV-SP6-vertebrate kozak-nls-RecF-nls-stop-EcoRV
GATATCATACGATTTAGGTGACACTATAGAACCGCCACCATGGTGCCCAAGAAAAAACGCAAGGTGTCCCTGACTA
GACTGCTGATTAGAGACTTTAGGAACATTGAAACTGCCGATCTGGCCCTGTCTCCAGGGTTCAACTTTCTGGTGGGA
GCCAATGGATCTGGCAAGACCAGTGTCCTGGAGGCCATCTACACACTGGGGCACGGACGGGCTTTCAGAAGTCTGC
AGATCGGGAGAGTGATCCGCCACGAGCAGGAAGCATTTGTCCTGCATGGACGCCTGCAGGGCGAGGAACGAGAGA
CTGCCATCGGCCTGACCAAGGACAAACAGGGGGATTCTAAGGTGCGGATTGACGGCACAGATGGGCACAAAGTCG
CTGAACTGGCACATCTGATGCCCATGCAGCTGATTACACCTGAGGGCTTCACTCTGCTGAACGGCGGGCCCAAGTAT
CGGAGAGCTTTCCTGGATTGGGGATGCTTTCATAATGAACCTGGCTTCTTTACTGCATGGAGCAACCTGAAGAGACT
GCTGAAACAGAGGAATGCCGCTCTGCGACAGGTGACCCGGTACGAGCAGCTGCGGCCATGGGACAAAGAACTGAT
CCCCCTGGCCGAGCAGATTTCCACCTGGAGAGCTGAATATTCTGCAGGAATCGCAGCCGACATGGCTGATACTTGT
AAGCAGTTCCTGCCCGAGTTTTCACTGACCTTCAGCTTTCAGAGGGGCTGGGAGAAAGAAACAGAGTACGCCGAAG
TGCTGGAGCGCAACTTCGAAAGAGATAGGCAGCTGACCTATACAGCTCACGGCCCTCATAAGGCAGACCTGCGCAT
TCGAGCCGATGGCGCTCCAGTCGAGGACACACTGTCCAGGGGGCAGCTGAAACTGCTGATGTGCGCACTGCGACTG
GCTCAGGGAGAATTCCTGACTAGAGAGAGCGGAAGGCGCTGTCTGTACCTGATCGACGATTTTGCCTCCGAACTGG
ACGATGAGCGACGAGGACTGCTGGCAAGTCGCCTGAAGGCAACCCAGTCACAGGTGTTTGTCAGCGCAATTTCCGC
CGAGCACGTGATTGACATGAGCGACGAGAATAGCAAAATGTTCACTGTGGAGAAAGGAAAGATTACCGATAGCCC
CAAGAAGAAGAGAAAGGTGGAGGCCAGCTGATAAGATATC

Supplementary Text S19 Sequence for RecF transcript template.

Supplementary Text S20:

>EcoRV-SP6-vertebrate kozak-nls-RecO-nls-stop-EcoRV

GATATCATACGATTTAGGTGACACTATAGAACCGCCACCATGGTCCCCAAGAAGAAGAGAAAGGTGGAAGGATGG
CAGAGAGCATTTGTGCTGCATTCACGCCCCTGGAGCGAAACTAGCCTGATGCTGGACGTGTTCACCGAGGAAAGCG
GAAGGGTGCGCCTGGTCGCTAAGGGAGCAAGAAGCAAAAGGTCCACTCTGAAGGGGGCACTGCAGCCATTCACCC
CACTGCTGCTGCGATTTGGAGGAAGAGGCGAGGTGAAAACACTGAGATCTGCAGAAGCCGTCAGTCTGGCTCTGCC
CCTGTCAGGGATCACTCTGTACAGCGGACTGTATATTAACGAGCTGCTGTCCAGGGTGCTGGAGTACGAAACCCGC
TTTTCTGAACTGTTCTTTGACTATCTGCACTGCATCCAGTCTCTGGCAGGAGTGACCGGAACACCAGAGCCTGCACT
GCGGAGATTCGAACTGGCCCTGCTGGGACACCTGGGATACGGAGTGAACTTCACCCATTGTGCAGGAAGTGGAGAG
CCAGTCGACGATACTATGACCTACCGGTACCGGGAGGAAAAAGGATTCATCGCTTCCGTGGTCATTGATAACAAGA
CCTTCACCGGCCGACAGCTGAAGGCCCTGAATGCTCGCGAGTTCCCTGACGCTGATACCCTGCGGGCCGCTAAGCG
GTTCACCCGAATGGCACTGAAACCTTATCTGGGAGGCAAGCCACTGAAGTCAAGAGAGCTGTTCAGACAGTTTATG
CCCAAGAGGACCGTCAAGACCCATTACGAGAGCCCCAAGAAGAAGAGAAAGGTGGAGGCCAGCTGATAAGATATC

Supplementary Text S20 Sequence for RecO transcript template.

Supplementary Text S21:

>EcoRV-SP6-vertebrate kozak-nls-RecR-nls-stop-EcoRV

GATATCATACGATTTAGGTGACACTATAGAACCGCCACCATGGTGCCCAAAAAGAAGAGAAAAGTCCAGACAAGC
CCCCTGCTGACCCAGCTGATGGAAGCCCTGAGGTGCCTGCCCGGCGTCGGCCCCAAGTCTGCTCAGCGCATGGCATT
CACCCTGCTGCAGAGGGACCGCAGTGGAGGAATGAGACTGGCACAGGCTCTGACAAGGGCCATGTCAGAGATCGG
CCACTGCGCTGATTGTCGAACCTTTACAGAGCAGGAAGTGTGCAACATCTGTAGCAATCCACGGAGACAGGAGAAC
GGGCAGATTTGCGTGGTCGAATCCCCCGCCGACATCTACGCTATTGAACAGACCGGCCAGTTCAGCGGGAGGTATT
TTGTCCTGATGGGACATCTGTCCCCCCTGGACGGGATCGGACCTGACGATATTGGACTGGATCGACTGGAGCAGCG
GCTGGCAGAGGAAAAAATCACAGAAGTGATTCTGGCCACTAACCCTACCGTCGAGGGGGAAGCAACTGCCAATTA
CATCGCAGAGCTGTGCGCCCAGTATGATGTGGAAGCTAGTCGGATTGCACACGGAGTGCCAGTCGGAGGCGAGCTG
GAGATGGTGGATGGAACAACACTGTCACATTCACTGGCAGGGAGGCACAAGATTCGGTTTAGCCCCAAGAAGAAG
AGAAAGGTGGAGGCCAGCTGATAAGATATC

Supplementary Text S21 Sequence for RecR transcript template.

Supplementary Text S22:

> SP6-vertebrate kozak-TagBFP -stop

CATACGATTTAGGTGACACTATAGAACCGCCACCATGAGCGAGCTGATTAAGGAGAACATGCACATGAAGCTGTAC
ATGGAGGGCACCGTGGACAACCATCACTTCAAGTGCACATCCGAGGGCGAAGGCAAGCCCTACGAGGGCACCCAG
ACCATGAGAATCAAGGTGGTCGAGGGCGGCCCTCTCCCCTTCGCCTTCGACATCCTGGCTACTAGCTTCCTCTACGG
CAGCAAGACCTTCATCAACCACACCCAGGGCATCCCCGACTTCTTCAAGCAGTCCTTCCCTGAGGGCTTCACATGGG
AGAGAGTCACCACATACGAAGACGGGGGCGTGCTGACCGCTACCCAGGACACCAGCCTCCAGGACGGCTGCCTCAT
CTACAACGTCAAGATCAGAGGGGTGAACTTCACATCCAACGGCCCTGTGATGCAGAAGAAAACACTCGGCTGGGA
GGCCTTCACCGAGACGCTGTACCCCGCTGACGGCGGCCTGGAAGGCAGAAACGACATGGCCCTGAAGCTCGTGGGC
GGGAGCCATCTGATCGCAAACATCAAGACCACATATAGATCCAAGAAACCCGCTAAGAACCTCAAGATGCCTGGCG
TCTACTATGTGGACTACAGACTGGAAAGAATCAAGGAGGCCAACAACGAGACCTACGTCGAGCAGCACGAGGTGG
CAGTGGCCAGATACTGCGACCTCCCTAGCAAACTGGGGCACAAGCTTAATTAA

Supplementary Text S22 Sequence for TagBFP transcript template.

Supplementary Excel S1. *GFAP* On-target deep-sequencing.
Supplementary Excel S2. *Slc6a4* On-target deep-sequencing.

Supplementary Movie S1:

Representative heart beating movie clip of Lefty2-linker-Dendra2 positive fish at 36 h. Lefty2-linker-Dendra2 positive F2 fish was dechorionated and embedded in 0.2% agrose. Movie of 36 h zebrafish heart that was originally taken at 4 frames per second by a Leica SP5 confocal microscopy was transformed to a 15s movie clip. Note that lefty2-linker-Dendra2 was able to label certain cell types in the fish heart.
